# Suppression of insulin-induced gene 1 (INSIG1) function promotes hepatic lipid remodelling and restrains NASH progression

**DOI:** 10.1016/j.molmet.2021.101210

**Published:** 2021-03-17

**Authors:** Vian Azzu, Michele Vacca, Ioannis Kamzolas, Zoe Hall, Jack Leslie, Stefania Carobbio, Samuel Virtue, Susan E. Davies, Agnes Lukasik, Martin Dale, Mohammad Bohlooly-Y, Animesh Acharjee, Daniel Lindén, Guillaume Bidault, Evangelia Petsalaki, Julian L. Griffin, Fiona Oakley, Michael E.D. Allison, Antonio Vidal-Puig

**Affiliations:** 1 Wellcome Trust/MRC Institute of Metabolic Science, Metabolic Research Laboratories, University of Cambridge, Cambridge, UK; 2 Liver Unit, Cambridge NIHR Biomedical Research Centre, Cambridge University Hospitals NHS Foundation Trust, Cambridge, UK; 3 Department of Gastroenterology and Hepatology, Norfolk and Norwich University Hospitals, Norwich, UK; 4 Department of Biochemistry and Cambridge Systems Biology Centre, University of Cambridge, Cambridge, UK; 5 Clinica Medica Cesare Frugoni, Department of Interdisciplinary Medicine, University of Bari Aldo Moro, Bari, Italy; 6 European Molecular Biology Laboratory, European Bioinformatics Institute (EMBL-EBI), Wellcome Genome Campus, Hinxton, UK; 7 Biomolecular Medicine, Systems Medicine, Department of Metabolism, Digestion and Reproduction, Imperial College London, London, UK; 8 Newcastle Fibrosis Research Group, Biosciences Institute, Faculty of Medical Sciences, 5 Newcastle University, Newcastle upon Tyne, UK; 9 Department of Pathology, Cambridge University Hospitals, Cambridge, UK; 10 Translational Genomics, Discovery Sciences, BioPharmaceuticals R&D, AstraZeneca, Gothenburg, Sweden; 11 College of Medical and Dental Sciences, Institute of Cancer and Genomic Sciences, Centre for Computational Biology, University of Birmingham, UK; 12 Bioscience Metabolism, Research and Early Development Cardiovascular, Renal and Metabolism (CVRM), BioPharmaceuticals R&D, AstraZeneca, Gothenburg, Sweden; 13 Division of Endocrinology, Department of Neuroscience and Physiology, Sahlgrenska Academy, University of Gothenburg, Sweden; 14 Wellcome Trust Sanger Institute, Hinxton, UK; 15 Cambridge University Nanjing Centre of Technology and Innovation, Jiangbei, Nanjing, China

**Keywords:** Non-alcoholic fatty liver disease (NAFLD), De novo lipogenesis (DNL), Lipid remodelling, Western diet, Carbon tetrachloride (CCl_4_), Liver regeneration

## Abstract

**Objective:**

Non-alcoholic fatty liver disease (NAFLD) is a silent pandemic associated with obesity and the metabolic syndrome, and also increases cardiovascular- and cirrhosis-related morbidity and mortality. A complete understanding of adaptive compensatory metabolic programmes that modulate non-alcoholic steatohepatitis (NASH) progression is lacking.

**Methods and results:**

Transcriptomic analysis of liver biopsies in patients with NASH revealed that NASH progression is associated with rewiring of metabolic pathways, including upregulation of *de novo* lipid/cholesterol synthesis and fatty acid remodelling. The modulation of these metabolic programmes was achieved by activating sterol regulatory element-binding protein (SREBP) transcriptional networks; however, it is still debated whether, in the context of NASH, activation of SREBPs acts as a pathogenic driver of lipotoxicity, or rather promotes the biosynthesis of protective lipids that buffer excessive lipid accumulation, preventing inflammation and fibrosis. To elucidate the pathophysiological role of SCAP/SREBP in NASH and wound-healing response, we used an *Insig1* deficient (with hyper-efficient SREBPs) murine model challenged with a NASH-inducing diet. Despite enhanced lipid and cholesterol biosynthesis, *Insig1* KO mice had similar systemic metabolism and insulin sensitivity to Het/WT littermates. Moreover, activating SREBPs resulted in remodelling the lipidome, decreased hepatocellular damage, and improved wound-healing responses.

**Conclusions:**

Our study provides actionable knowledge about the pathways and mechanisms involved in NAFLD pathogenesis, which may prove useful for developing new therapeutic strategies. Our results also suggest that the SCAP/SREBP/INSIG1 trio governs transcriptional programmes aimed at protecting the liver from lipotoxic insults in NASH.

## Highlights

• Human NASH biopsies’ transcriptomics analysis features metabolic pathway rewiring.
• SCAP/SREBP/INSIG1 modulation promotes lipid/cholesterol synthesis/remodelling in NASH.
• Loss of Insig1 promotes lipid remodelling, preventing hepatic lipotoxicity in NASH.
• Loss of Insig1 improves liver damage and wound healing and restrains NASH progression.

## 1 Introduction

Non-alcoholic fatty liver disease (NAFLD) encompasses a spectrum of liver disease affecting up to one-third of Western populations [1], characterised by hepatic steatosis, leading to lipotoxicity, inflammation (non-alcoholic steatohepatitis, NASH), and fibrosis, potentially culminating in advanced liver disease [2] and conferring increased cardiovascular risk/mortality [3]. NAFLD is associated with obesity and/or adipose tissue dysfunction, insulin resistance (IR), and multiple features of the metabolic syndrome [4]. Moreover, the exponential increase in obesity and NAFLD means this condition is presently responsible for an enormous economic and health burden [5].

Despite recent advances in understanding NASH pathophysiology, important information is lacking regarding specific factors driving NAFL development and the mechanistic links between systemic metabolic stress and disease progression [6]. An increase in *de novo* lipogenesis (DNL) and lipid remodelling has been reported in multiple studies of subjects with NAFLD [[7], [8], [9]] and shown to be a significant factor in NASH pathophysiology [10]. Dysregulation of DNL and cholesterol synthesis in liver metabolism is a feature of NASH [11,12] and fibrogenesis [13].

In our cohort of biopsy-proven NASH patients, transcriptomic analysis suggested that the transcriptional complex of sterol regulatory element-binding protein 1 and 2 (SREBP1/SREBF1 and SREBP2/SREBF2) and SREBP cleavage-activating protein (SCAP) were strongly activated; this was associated with enhanced metabolic programmes governing cholesterol/lipid synthesis and remodelling. However, observational “omics” were unable to explain 1) whether these transcriptional regulators induced a maladaptive response or their activation might represent a protective mechanism aimed at maintaining the liver's functionality when metabolically challenged by an excess nutrient/calorie load, and 2) the consequences, in terms of compensatory mechanisms, of activating these pathways on connected metabolic programmes (for example, synthesis of complex lipids, lipid catabolism, and lipoprotein handling) [14].

To study the biological consequences of these processes, we used a murine model characterised by unrestrained SREBP1 and SREBP2 activity resulting from the absence of insulin-induced gene 1 (INSIG1) along with a NASH-inducing diet. INSIG1 is part of negative regulatory feedback, acting as a brake on SREBP transcriptional function [[15], [16], [17]]. INSIG1 also has a secondary function mediating sterol-accelerated proteolytic degradation of HMG CoA reductase, thus impacting the mevalonate pathway [18]. In this report, we show that, in a NASH-like metabolic context with acute oxidative damage and hyper-efficient SREBP activation, despite the collateral cost of increased hepatic lipid deposition, INSIG1 deficiency leads to a beneficial outcome in NASH driven by an increase in desaturase activity and lipid remodelling as well as a multifaceted regulation of multiple metabolic pathways preventing hepatic lipotoxicity.

## 2 Methods

### 2.1 Study population (patients with NAFLD)

Our BioNASH cohort consisted of 58 consecutive patients recruited by the NAFLD service at Cambridge University Hospital's NHS Foundation Trust. This study was approved by the Research Ethics Committee (ref: 06/Q0106/70). All of the patients provided informed consent for the use of data (biochemistry and clinical history) and samples for research purposes; the principles of the Declaration of Helsinki were followed. All of the patients had a clinical diagnosis of NAFLD (with alternate diagnoses and aetiologies excluded), histology scored by an expert pathologist according to the NASH Clinical Research Network scoring system [19,20], and snap-frozen tissue for research purposes. For this study, histology was grouped into NAFL (fatty liver without inflammation and/or ballooning) and NASH (fatty liver with inflammation and ballooning) [21] with fibrosis scores of 1–2 (moderate) or 3–4 (advanced). The study population is described in Table S1.

### 2.2 Animals

All of the data were from male mice. *Insig1* KO mice were generated by Cre-mediated deletion of three *Insig1* exons (exons 2–4) as previously described [15] on a C57Bl6/J background and compared to wild-type and heterozygous littermates. The mice were maintained in a room with a controlled temperature (21 °C; 12 h light/dark cycle) with free access to food and water (ad libitum); the pathogen-free facilities were compliant with FELASA guidelines [22]. The UK Home Office and USC Bioethics Committee of the Universities of Cambridge and Newcastle approved all of the animal procedures. The mice were fed a chow diet (Safe Diets, Code ds-105) until they were enrolled in specific procedures.

### 2.3 Western diet and sugar water studies

At the age of 8 weeks, the mice were fed either low-fat control (Teklad, TD08485) or Western diets (Teklad, TD88137) plus drinking water supplemented with fructose and glucose (SW, 23.1 g/L of d-fructose +18.9 g/L of d-glucose) ad libitum starting from week 8; the total duration of the experimental challenge was 12 weeks. Fat/lean mass was calculated by time-domain nuclear magnetic resonance (TD-NMR) using a Minispec LF50 Live Mice Analyser (Bruker) the day before culling. Serum and tissue were collected in a fed condition the day of the culling.

### 2.4 Carbon tetrachloride studies

The 12-week-old *Insig1* KO, Het, or WT mice underwent intraperitoneal (IP) injection of 2 μl/BW (g) of CCl_4_: virgin olive oil [1:1 (v/v)] mix or virgin olive oil alone. Fed mice were humanely culled under terminal isoflurane anaesthesia four days after the IP injection by cutting the heart and inferior vena cava. Blood was collected from the chest cavity. These experiments were approved by the Animal Welfare and Ethical Review Board and carried out at Newcastle University Comparative Biology Centre under a UK Home office license.

### 2.5 Data availability

All of the raw data (FASTQ files) and relevant metadata are available in the Array Express database (www.ebi.ac.uk/arrayexpress) under accession numbers E-MTAB-9815 and E-MTAB-9864.

#### 2.5.1 Additional methods provided in supplementary files

Biochemical serum analyses, murine tissue collection and processing, histology, immunohistochemistry, tissue imaging, quantification, and scoring, RNA extraction and RNA integrity, reverse transcription (RT) polymerase chain reaction (PCR), whole-transcriptome amplification and RNA sequencing, hepatic lipid extraction and lipidomics, quantification and statistical analysis, NGS data processing, and bioinformatics functional analyses.

## 3 Results

### 3.1 Progressive human NASH was characterised by increasing lipid and cholesterol biosynthesis transcriptional programmes

We first recruited 58 patients with biopsy-proven NAFLD clustered into three groups (NAFL, moderate NASH, and advanced NASH) according to the NASH Clinical Research Network's scoring system [[19], [20], [21]]. In addition to the expected differences in the histology, we also found a progressive increase in body mass index (BMI) and worsening systemic insulin resistance (HOMA-IR) in NASH (Table S1). No other significant changes among the groups were recorded with regards to metabolic biochemistry and transaminases.

**Figure 1:**
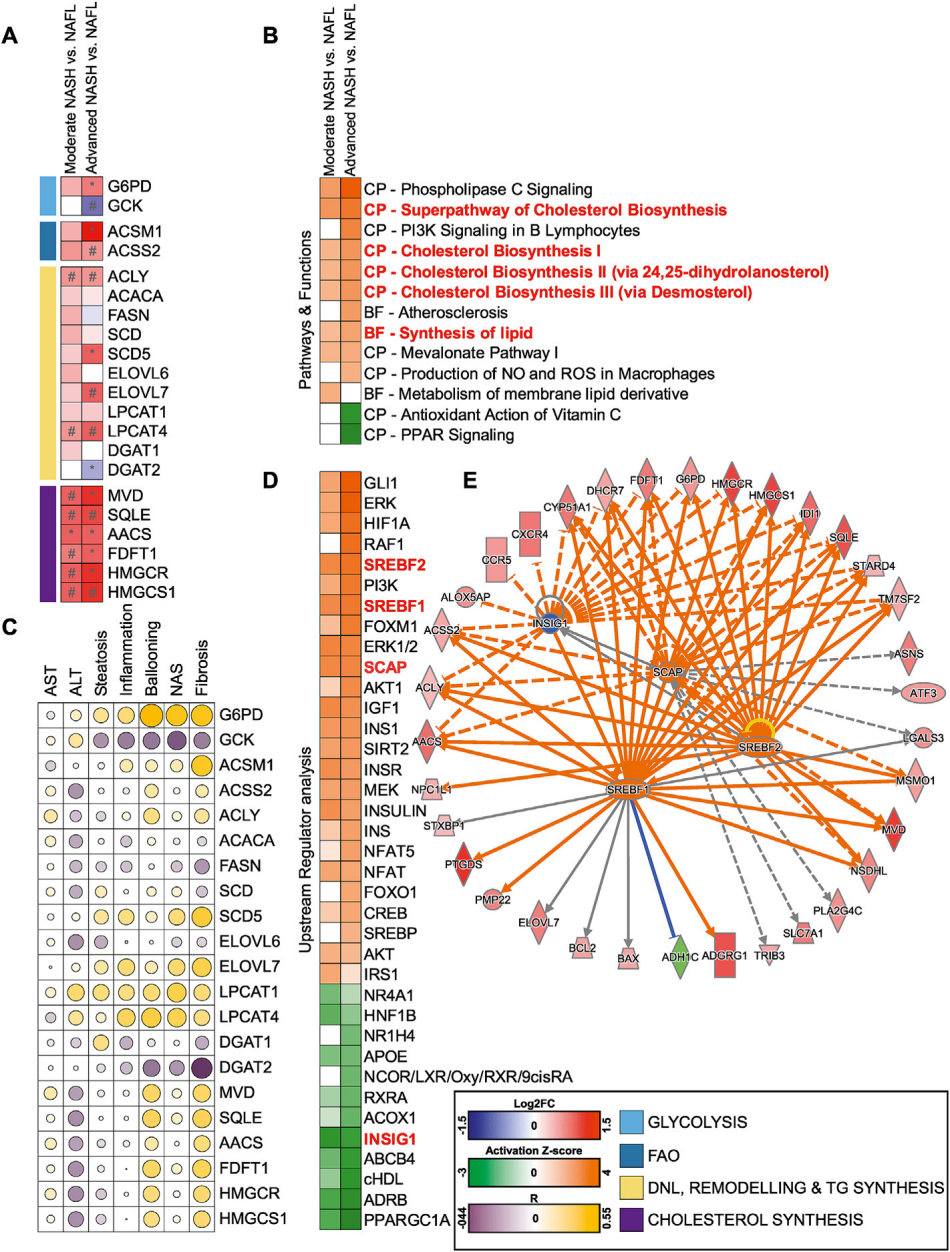
**NGS analysis predicts a strong regulation of metabolic pathways in biopsy-proven NASH clustered against the disease stage.** (A) Gene expression profiled by NGS and analysed with the Wald test (sample size defined in Table S1; #p < 0.05 and ∗p _BH_ < 0.05) of key metabolic enzymes involved in glucose and lipid metabolism. (B) Selection of canonical pathways (CP) and bio-function (BF) predicted to be significantly (p < 0.05) enriched and activated (orange) or inhibited (green) according to the Ingenuity Pathway Analysis (IPA; −2 < Z score >2 in at least one comparison); expanded list in Table S2. (C) Correlation of genes with hallmarks of NASH progression (histology and transaminases). (D) Selection of upstream regulators predicted to be significantly (p < 0.05) enriched and activated (orange) or inhibited (green) according to the IPA (−2 < Z score >2 in at least one comparison); expanded list in Table S2. (E) Graphical representation in network format of SCAP/SREBF1 and 2 and INSIG1 transcriptional activity based on the regulation of their targets was differentially modulated (green: downregulated; red: upregulated), leading to the predicted inhibition (blue)/activation (orange). Data are shown in a heat map matrix format; details are in the graphical legend. (For interpretation of the references to color in this figure legend, the reader is referred to the Web version of this article.)

Using next-generation sequencing (NGS), we studied transcriptomic changes associated with NASH progression. We first focused on changes in the transcripts of individual genes involved in metabolic programmes and specific mechanisms of disease. We validated the upregulation of known drivers of disease progression involved in apoptosis, inflammation, hepatic stellate cell (HSC) activation and fibrosis, and compensatory hepatocyte proliferation (Figure S1A). Of relevance, we also found that the progression of NASH was associated with a coordinated and robust rewiring of metabolic pathways. Specifically, we found the upregulation of many transcripts involved in DNL, fatty acid remodelling, and triglyceride and cholesterol biosynthesis (Figure 1A) following the same trend of modulation observed for hyperinsulinaemia. The activation of these lipid metabolic transcriptional programmes with disease progression was further confirmed by the canonical pathway (CP) and biological functions (BF) of the Ingenuity Pathway Analysis (IPA; Figure 1B; a detailed list of significant hits is in Table S2). Many of these lipid-related genes correlated with hallmarks of metabolic impairment (Figure S1B), with the expression of critical drivers of disease progression (Figure S1C), elevated transaminases, and NASH histological features including steatosis, ballooning degeneration (cell death), inflammation, and fibrosis (Figure 1C).

To better understand the network of transcriptional regulators that might govern the metabolic adaptation to disease progression, we further refined the NGS analysis with an IPA “upstream regulator” analysis (URA), which predicts the degree of activation of transcription factors and signalling regulators accounting for changes in their downstream targets (Figure 1D; details in Table S2). The URA confirmed a strong activation of multiple downstream effectors of insulin signalling. Activation of insulin signalling was coupled with suppression of multiple lipid-sensing nuclear receptors and their coactivators, including farnesoid X receptor (FXR/*NR1H4*), liver X receptor (LXR), retinoid X receptor alpha (RXRα), and peroxisome proliferator-activated receptor gamma coactivator 1 alpha (PGC1α/*PPARGC1A*) (Figure 1D). Of note, some of these hits are currently considered candidate targets for the pharmacological treatment of NASH [14].

Among the differentially activated upstream regulators, IPA predicted the activation of the two master transcription factors controlling DNL and cholesterol synthesis namely SREBP1/SREBF1*,* SREBP2/SREBF2, and their interacting partner SCAP; as well as suppressed activity of INSIG1 (Figure 1D,E), a target gene of SREBP1 and an inhibitor of SREBP1/2, functioning as a crucial negative feedback loop controlling SREBP1/2 cleavage and activity [15,17,23,24].

Altogether, these data suggested that the co-regulation of INSIG/SREBPs is tightly associated with the pathophysiology of NASH progression. The pathobiological consequences of DNL activation in a NASH context are under debate and yet to be fully understood. One view is that activation of SREBPs drives lipotoxicity [13,[25], [26], [27]]; an alternative view is that SREBPs also co-regulate desaturases and other lipid remodelling enzymes, potentially protecting from insulin resistance and lipotoxicity [28,29]. To determine whether SREBPs are protective or deleterious, we used an *Insig1* KO mouse characterised by hyper-functional SREBP transcriptional activity that was subjected to a dietary challenge to model NAFLD.

### 3.2 Absence of *Insig1* resulted in a more benign hepatic lipidome and decreased hepatic damage induced by a NASH-inducing challenge

To study the contribution of decreased INSIG1 in the early stages of NASH, we exposed *Insig1* KO mice and their wild-type (WT) and heterozygous (Het) littermates to a Western diet (high in saturated fat, cholesterol, and refined carbohydrates) supplemented with sugar water (WDSW) ad libitum, thus promoting activation of SREBP1 and ChREBP transcriptional programmes [30].

In line with our previous observations where we defined that *Insig1* KO mice were smaller/shorter (but not leaner) than their WT littermates [15], *Insig1* KO mice body weights (Figure S2A) were reduced compared to the other genotypes without genotype-associated differences in fat mass (Figure S2B), liver weight to body weight ratio (Figure S2C), metabolic biochemistry (Figsure S2D–G: glucose, TG, cholesterol, and free fatty acid), or markers of systemic and peripheral insulin resistance (Figures. S2H–K). *Insig1* KO mice were therefore indistinguishable from their WT/Het littermates with regards to the systemic metabolic phenotype as previously described by our group and others [15,17].

**Figure 2:**
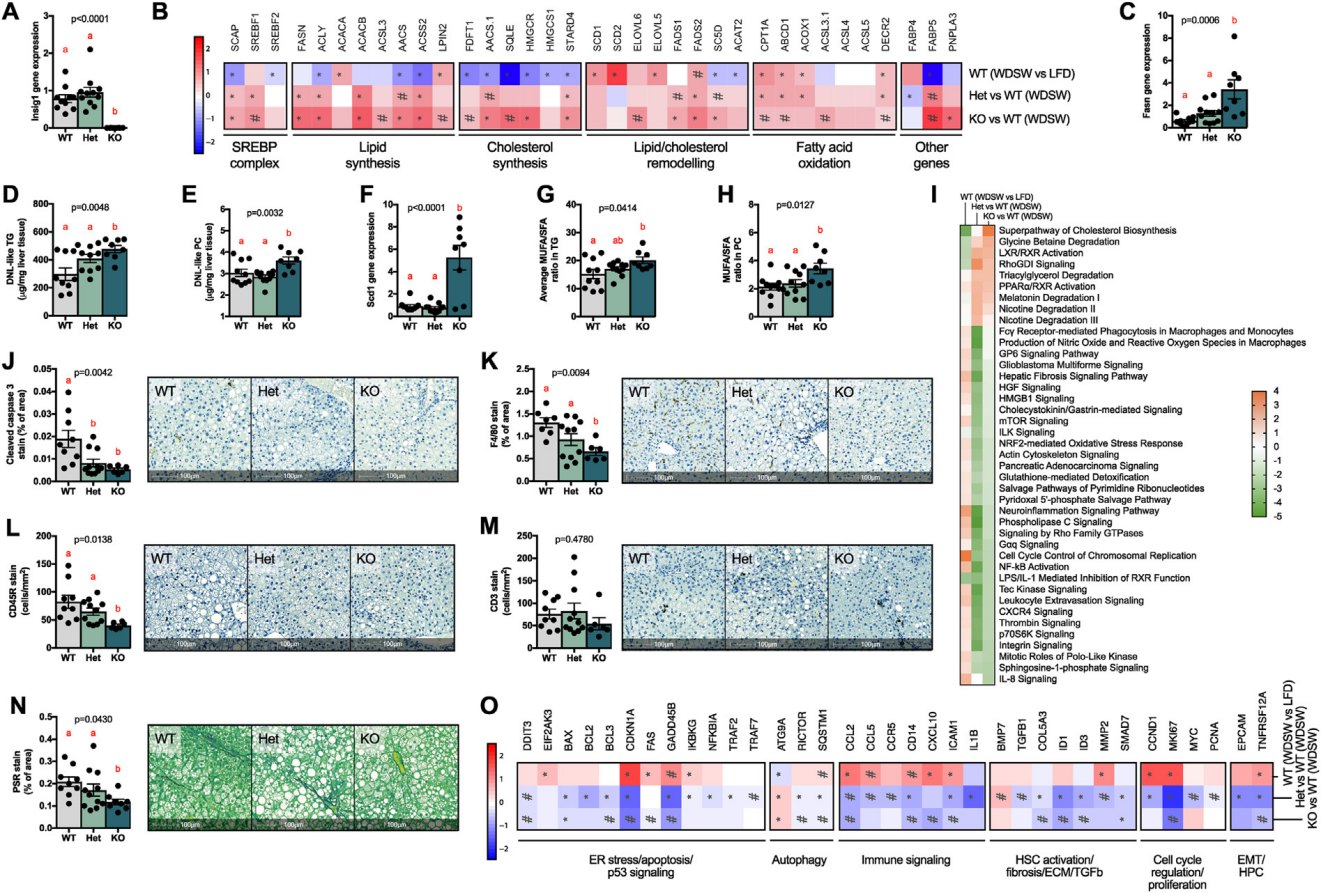
***Insig1* ablation improved the lipidome and reduced cellular damage, inflammation, and extracellular matrix deposition in a Western diet supplemented with sugar water.** (A) Relative *Insig1* mRNA expression measured by RTqPCR. (B) NGS showing upregulation of key enzymes in lipid/cholesterol synthesis, remodelling, and oxidation. (C) Relative *Fasn* mRNA expression measured by RTqPCR. DNL-like triglycerides (D) and phosphatidylcholine (E) as measured by LC-MS. (F) Relative *Scd1* mRNA expression as measured by RTqPCR. Monounsaturated fatty acid-containing triglycerides (G) and phosphatidylcholines (H). (I) Prediction of canonical pathways significantly enriched and predicted as activated (orange) or inhibited (green) according to IPA; data are shown as a heat map matrix format representing the prediction of activation (Z score). HALO imaging software analysis on scanned slides of whole tissue of: (J) caspase 3 IHC, (K) F4/80 IHC, (L) CD45R IHC, (M) CD3 IHC, and (N) Picrosirius Red. Representative tissues are shown. (O) Gene expression of selected genes involved in apoptosis, autophagy, compensatory hepatocyte proliferation, inflammation, hepatic stellate cell (HSC) activation and fibrosis, and epithelial–mesenchymal transition (EMT) as profiled by NGS. Data were analysed by ANOVA (p values < 0.05 are considered significant) with Tukey's post hoc test (a, reference group; groups with different letters are statistically different per post hoc comparison; differences between groups with the same letter are statistically not significant per post hoc comparison). NGS data were analysed with the Wald test (#p < 0.05 and ∗p_BH_ < 0.05). (For interpretation of the references to color in this figure legend, the reader is referred to the Web version of this article.)

We previously described that INSIG1 in adipose tissue promotes a negative feedback loop on SREBP1 activation, ensuring SREBP1 function in the context of IR (when the transcriptional drive of both is reduced) [15]. Absence of *Insig1* in the liver (Figure 2A) resulted in a marked upregulation of SREBP1 and SREBP2 target genes, leading to enhanced lipid/cholesterol synthesis and remodelling (Figure 2B). The total hepatic lipid and triglyceride (TG) composition was only marginally affected, with a mild upward trend in the KO mice without reaching statistical significance (Figures. S2L–M) despite an increase in *Fasn* (qPCR; Figure 2C) and “DNL-like” [8] lipids (Figure 2D–E); this was probably the consequence of the upregulation of genes encoding key beta-oxidation enzymes (Figure 2B) [31]. More broadly, the upregulation of lipid remodelling genes in the KO mice was reflected by an overall change in the triglyceride composition (elongation and desaturation) in the liver (Figure S2N). Furthermore, the upregulation of SREBP1 target *Scd1* and of other desaturases (Figure 2B,F) led to qualitative alterations in the repertoire of fatty acid (FA) desaturation in TGs and phosphatidylcholines (PCs) as shown by the increased ratio of monounsaturated fatty acid (MUFA) to saturated fatty acid (SFA; Figure 2G–H). We did not detect differences in cholesterol ester (CE) species between the genotypes (Figure S2O), possibly due to feedback inhibition of cholesterol synthesis from the diet's cholesterol. Overall, an enhanced remodelling of the lipidome toward desaturated lipids is expected to reduce lipotoxicity in murine models of NASH [28]; accordingly, the *Insig1* KO mice showed reduced hepatocyte damage with ALT values below the upper limit of normal (Figure S2P) which, although “clinically” significant, did not reach statistical significance. We therefore evaluated by NGS (Figure 2I) and histology (Figure 2J–N and Figure S2Q) whether the livers of the *Insig1* KO mice exhibited a milder NASH phenotype compared to the WT and Het littermates with regards to hepatocellular damage, inflammation and fibrosis. Figure 2I shows than in *Insig1* KO livers, there was downregulation of oxidative pathways and a concordant decrease in oxidative stress response. Consequently, we observed reduced apoptosis (gene expression in Figure 2I; cleaved caspase 3 IHC in Figure 2J), inflammation (gene expression in Figure 2I; inflammation markers' IHC in Figure 2K–M and Figure S2Q) and, despite this being a model of very mild NASH, extracellular matrix (ECM) deposition (picrosirius red, Figure 2N). Our study also revealed that the KO animals had decreased liver infiltration of lymphocytes (CD3^+^ T cells and CD45R^+^ B cells, Figure 2L–M) and a trend toward decreased myeloid cells (Ly6C/G^+^; monocytes/neutrophils, Figure S2Q), in keeping with the extensive literature detailing the involvement of these cell types in NAFLD progression [[32], [33], [34]]. The analysis of the hepatic transcriptome (Figure 2I and O; details in Table S3) and IPA confirmed that the *Insig1* KO mice were protected from ER stress (for example, *Ddit3*), oxidative damage, and apoptosis (for example, *Bax* and *Fasl*), featuring a substantial suppression of pathways involved in the wound-healing response (inflammation, compensatory hepatocyte proliferation, and ECM remodelling).

These results indicated that INSIG1 inactivation in the context of NAFLD facilitated the hyper-efficient function of SREBP transcriptional programmes that prevented WDSW-induced hepatic lipotoxicity, leading to a milder NASH phenotype.

### 3.3 Absence of *Insig1* protected the liver from carbon tetrachloride-induced acute hepatocellular damage

**Figure 3:**
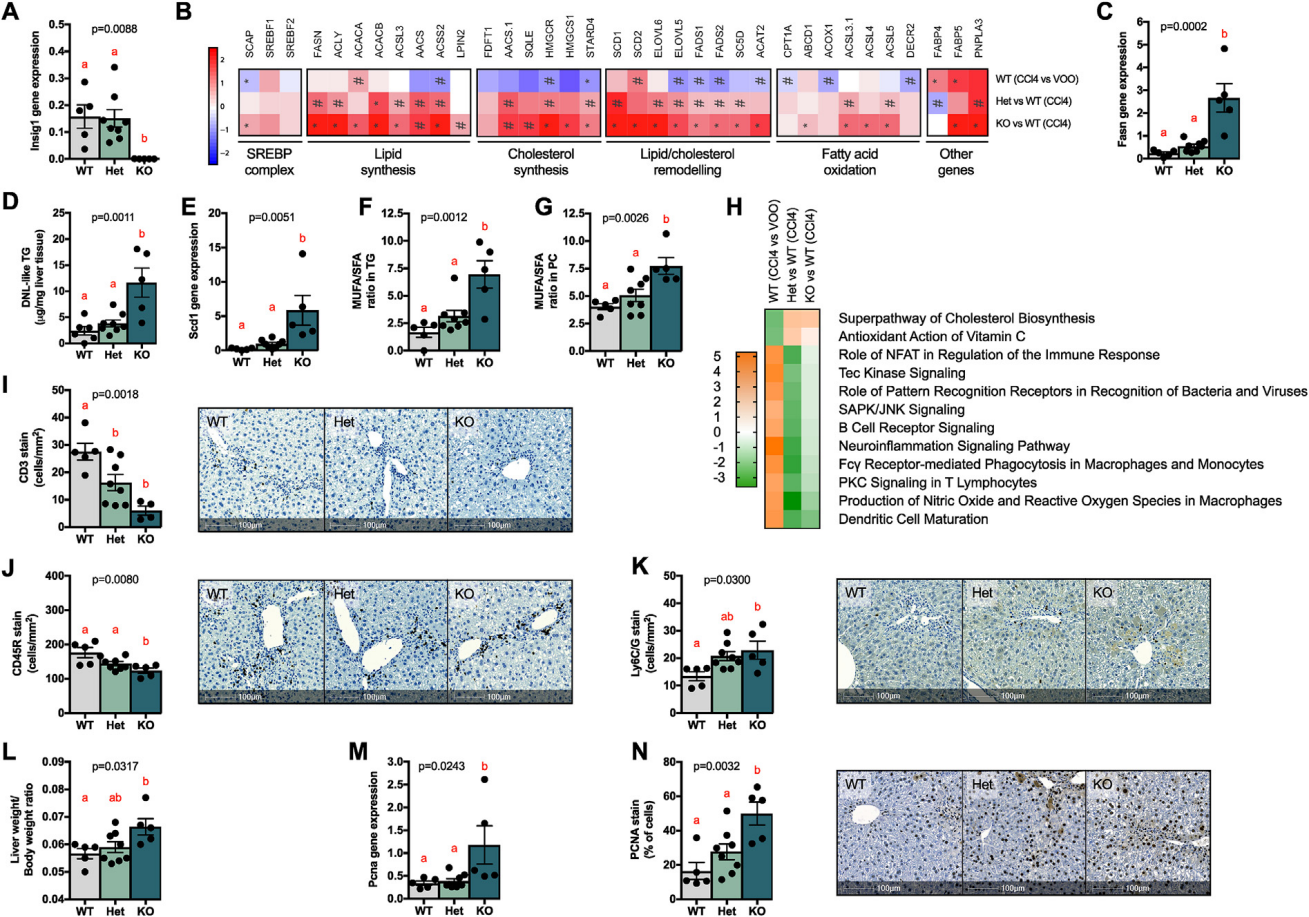
***Insig1* ablation improved the lipidome and reduced cellular damage in animals treated with CCl**_**4**_**.** (A) Relative *Insig1* mRNA expression measured by RTqPCR. (B) NGS showing upregulation of key enzymes in lipid/cholesterol synthesis, remodelling, and oxidation. C) Relative *Fasn* mRNA expression measured by RTqPCR. (D) DNL-like triglycerides measured by LC-MS. (E) Relative *Scd1* mRNA expression measured by RTqPCR, monounsaturated fatty acid-containing triglycerides (F), and phosphatidylcholines (G). (H) Prediction of canonical pathways was significantly enriched and predicted as activated (orange) or inhibited (green) according to the IPA; data are shown as a heat map matrix format representing the activation Z score prediction by the IPA. HALO imaging software analysis on scanned slides of whole tissue of (I) CD3 IHC, (J) CD45R IHC, and (K) Ly6C/G IHC. Representative tissues are shown. (L) Liver weight/body weight ratio. (M) Relative *Pcna* mRNA expression measured by RTqPCR. (N) PCNA IHC analysed by HALO software. Data were analysed by ANOVA (p values < 0.05 are considered significant) with Tukey's post hoc test (a, reference group; groups with different letters are statistically different per post hoc comparison; differences between groups with the same letter are statistically not significant per post hoc comparison). NGS data were analysed with the Wald test (#p < 0.05 and ∗p_BH_ < 0.05). (For interpretation of the references to color in this figure legend, the reader is referred to the Web version of this article.)

We next posited that the less harmful composition of the hepatic lipidome in the *Insig1* KO mice, leading to reduced hepatocellular damage in the obesity-specific WDSW challenge, might also be protective in the context of other forms of liver damage. Following the transcriptomic changes in the WDSW model pointing to protection from oxidative damage, we investigated this paradigm in the acute carbon tetrachloride (CCl_4_) model that induces substantial reactive oxygen species-mediated hepatotoxicity. One day after CCl_4_ injection, the *Insig1* Het and KO mice showed a trend of reduced hepatotoxicity (ALT, Figure S3A) compared to their WT littermates and this trend flattened on day four after the challenge (ALT, Figure S3B). We chose day four post-CCl_4_ injection as a time point to study the hepatic phenotype in the context of the wound-healing response (which peaks at 3–5 days) [35]. In line with the WDSW model and our previous report [15], the KO mice were smaller than their WT and Het littermates (BW, Figure S3C), but there were no genotype-associated differences in the systemic metabolism (TG, cholesterol, glucose, insulin, and HOMA-IR, Figures. S3D–H). However, as a result of the genetic deletion of *Insig1* (Figure 3A), we found a statistically significant upregulation of SREBP1 and SREBP2 targets involved in lipid synthesis and remodelling (Figure 3B), reflected by an overall change in the triglyceride composition (elongation and desaturation) in the liver (Figure S3I). As in the WDSW model, *Fasn* upregulation (Figure 3C) led to the accumulation of “DNL-like” TG (Figure 3D) and preferential *Scd1* upregulation (Fig. 3E) led to the accumulation of MUFA over SFA species in TGs and PCs (Figure 3F–G). We also observed increased cholesterol ester species in the KO animals (Figure S3J) in line with activation of cholesterol synthesis pathways (Figure 3B). Total hepatic lipid content remained unchanged (Figure S3K-L), possibly due to a mild upregulation in fatty acid oxidation pathways (Figure 3B). The NGS pathway analysis (Figure 3H; details in Table S4) confirmed that the KO livers exhibited increased antioxidant defences in the face of the CCl_4_ challenge (ascorbate pathway, *G6pd* upregulation in the KO), reduced reactive oxygen species production, and with regards to the inflammatory component of the wound-healing response, reduced signalling associated with lymphocyte infiltration, which was confirmed by IHC (reduced CD3+ T cells in Figure 3I and Cd45r+ B cells in Figure 3J). In this model, we did not detect any difference in F4/80 macrophages (Figure S3M) or αSMA-positive cells (that is, activated stellate cells, Figure S3N). Intriguingly, Ly6C/G^+^ cells (monocytes and neutrophils) increased in the livers of the KO mice (Figure 3K). We are tempted to speculate that this may be reflective of a more appropriate inflammatory response [36] to cytotoxic CCl_4_-induced damage and will be the focus of future studies aimed at ascertaining the biological mechanisms with cell-specific approaches.

Another critical aspect of the CCl_4_ model was that it allowed us to study the hepatocytes' compensatory regenerative programmes as part of the wound-healing responses activated by acute liver injury. This was an essential component of the *Insig1* KO mice phenotype to characterise since 1) the *Insig1* KO mice had increased LW to BW ratios (Figure 3L) in the CCl_4_ model, 2) hepatocyte proliferation was primed by pro-inflammatory pathways led by myeloid cells (increased in the *Insig1* KO mice) [37,38], 3) SREBP1 has been proposed as a direct regulator of cell cycle and proliferation [39,40], and 4) we and others have shown that cholesterol synthesis and MUFA PC production are key metabolic processes for hepatocyte proliferation [35,38]. Analysis of proliferating cell nuclear antigen (*Pcna* mRNA by qPCR in Figure 3M and PCNA protein by IHC in Figure 3N) showed that *Insig1* KO livers featured enhanced hepatocyte compensatory proliferation compared to the other genotypes. This phenotype was also confirmed by the IPA upstream regulator analysis of the KO vs WT mice, suggesting a strong activation of the proliferative programmes (CMYC, CCNE1, and CCDD1) supported by a dense and coherent activation of WNT and Hippo signalling pathways (β-CATENIN/CTNNBIP, FOXM1, JNK, YAP1, MTOR/MTORC1, CMYC, CCND1, TBX2, and TP73), growth factor signalling cascades (EGF, HGF, VEGF, and CSF2), STAT3 and STAT6 activation, and many other mediators of hepatocyte proliferation/survival and de-differentiation. Coherent tumour suppressors and cell cycle checkpoints (TP53 and PTEN) were suppressed (Figure S4; details in Table S5).

Overall, these data indicated that protection from hepatocellular damage in the *Insig1* KO mice was replicated in a model of acute hepatic damage (CCl_4_), whereby the KO mice featured reduced adaptive immune cell infiltration paralleled by enhanced compensatory hepatocyte proliferation, resulting in hepatoprotection from CCl_4_. Therefore, we speculate that inhibiting INSIG1 function might not only be an efficient strategy to reduce hepatic cytotoxicity, but also to favour more efficient liver regeneration in the context of acute hepatocellular damage.

### 3.4 NGS analysis confirmed that *Insig1* absence and increased DNL/lipid remodelling resulted in a favourable metabolic and inflammatory milieu in WDSW and CCl_4_ models

**Figure 4:**
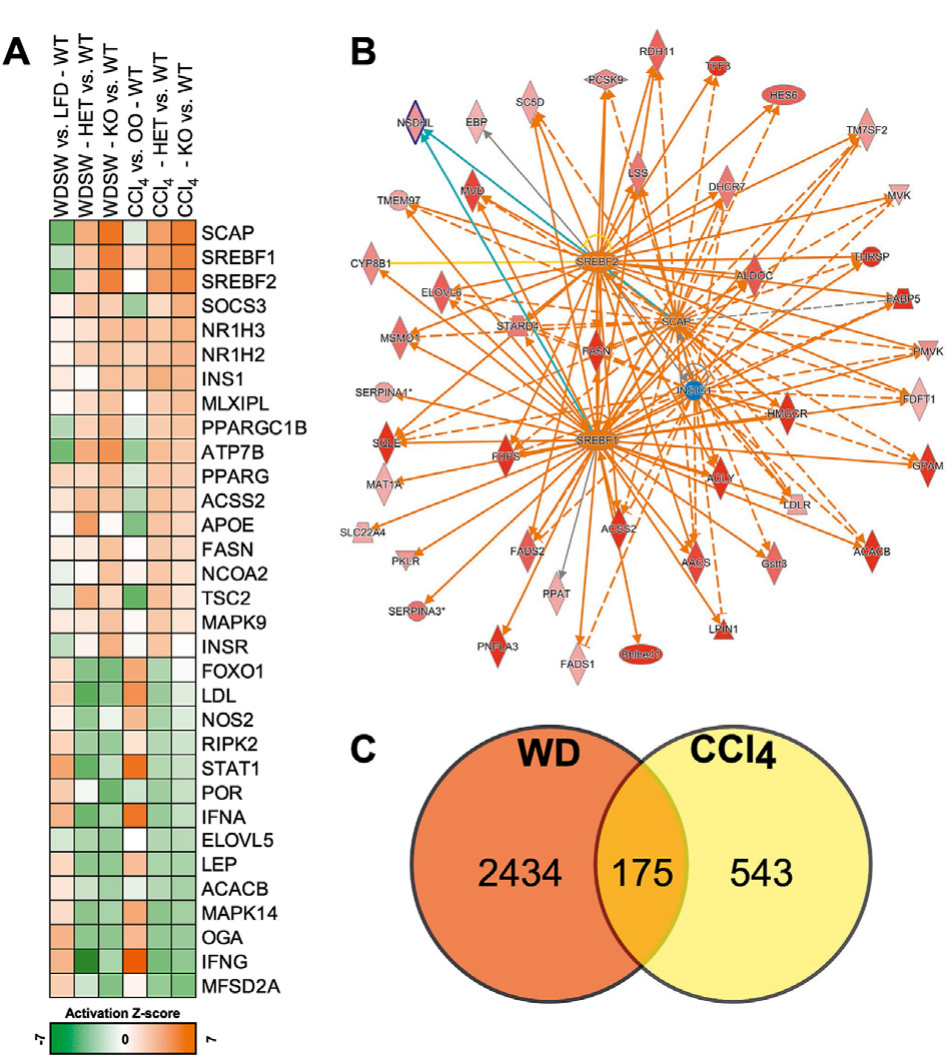
**NGS analysis confirmed *Insig1* ablation impacted metabolic and inflammatory pathways in WDSW and CCl**_**4**_**models.** (A) Prediction of upstream regulators significantly enriched and predicted (Z score) activated (orange) or inhibited (green) according to the IPA/URA in the Het and KO (vs WT) in the WDSW and CCl4 models (full list in Table S5). (B) Graphical representation in networks of differentially modulated genes (green, downregulated; red, upregulated) leading to the predicted activation (orange)/inhibition (blue) of the upstream regulators according to the IPA; data from the WDSW model. (C) Comparison of the genes differentially modulated in the treated *Insig1* KO mice (vs WT), a subset of 175 genes were differentially modulated the WDSW and CCl_4_ models. (p < 0.05; −0.378 ≤ log2 [fold change] ≥ 0.378; full list in Table S6). (For interpretation of the references to color in this figure legend, the reader is referred to the Web version of this article.)

To interpret the complex mechanisms that characterise the deletion of *Insig1* in liver damage, we performed the upstream regulator analysis (URA) in the IPA to highlight those adaptive changes that were associated with the absence of *Insig1* independently from the challenge. In addition to the expected activation of SREBF1/2 (Figure 4A–B; details in Table S6), the *Insig1* KO mice exhibited a pleiotropic activation of multiple regulators of hepatic metabolism that might have contributed to the more benign response to liver damage. Specifically, we found strong activation of lipid-sensing transcriptional programmes such as the liver X receptors (NR1H2/3), the peroxisome proliferator-activated receptor gamma (PPARG) and PPARG coactivator 1 beta (PGC1β/PPARGC1B). These transcriptional regulators contribute to lipid and cholesterol metabolism, fatty acid oxidation, lipoprotein secretion, and negative regulation of inflammation and fibrogenesis [14,39,[41], [42], [43]] potentially contributing to protecting the *Insig1* KO mice from liver damage. IPA also predicted an improvement of other metabolic pathways and insulin sensitivity (activation of INS, INSR, and MAPK9), reduced ER stress (MAPK14 and FOXO1), and suppression of inflammatory modulators (NOS2, RIPK2, STAT1, IFNA, and IFNG). All of these upstream regulators have been shown to be involved in NASH progression, lending weight to the hypothesis that *Insig1* downregulation might directly/indirectly lead to a pleiotropic protective set of mechanisms that attenuate inflammation and liver damage in NAFLD [41,42].

We also retrieved a list of 175 genes that are significantly modulated in the same direction in dietary and chemical models as a result of *Insig1* deletion (Figure 4C; details in Table S6). These genes are not only limited to SREBP-mediated pathways (for example, *Acacb*, *Fasn*, *Elovl6*, *Hmgcr*, and *Pnpla3*), but also carbohydrate metabolism (*Pfkfb1* and *Pklr*), anti-oxidant pathways (*Thrsp*, *Slc23a1*, and *Cyb5r3*), inflammation (*Cd14*, *Il22ra*, and *Plin5*), ECM remodelling (*Col5a3* and *Itga5*), and proliferation (*Blm*), further confirming the predicted regulation of the previously described pathways and upstream regulators.

## 4 Discussion and conclusion

Increased hepatic DNL is associated with NAFLD development, especially the development of steatosis [[7], [8], [9], [10],^43^,^44^]. Although multiple reports have associated DNL with NASH progression [45,46], these anecdotal associations in observational studies have not been substantiated by translational studies demonstrating that DNL is unequivocally a direct causal event in NASH progression and fibrosis. Metabolic programmes governing DNL also substantially reshape lipid remodelling: DNL and lipid remodelling co-regulation could also be a biomarker of allostatic protective responses aimed at producing specific types of lipids required for membranes/organelle homeostasis and safe storage of lipids in the form of triglycerides, maintaining the liver's functionality [47,48]. In this study, we used a transcriptomic analysis of liver biopsy samples from 58 patients with biopsy-proven NAFLD to show that progression to NASH and fibrosis is associated with a rewiring of metabolic pathways, most notably those associated with DNL, lipid desaturation, fatty acid remodelling, and cholesterol synthesis (Figure 1). These findings confirm previous reports investigating circulating lipid species, suggesting that complex lipids enriched in DNL products could act as biomarkers of the disease [8,49,50]. The IPA upstream regulator analysis predicted the activation of the SREBF1/SREBF2/SCAP network and the suppression of INSIG1 as critical integrated events associated with increasing NASH/fibrosis stage. Our group previously suggested that INSIG1, a direct target of SREBP1, acts as a brake, tuning SCAP/SREBP functional activation. Thus, INSIG1 should be considered the keystone protein in a negative feedback loop, ensuring the maintenance of qualitative aspects of lipid desaturation and cholesterol composition. This allostatic adaptation's proposed goal is to maintain required biophysical properties and functionality of cellular membranes when confronted with the challenges of obesity, nutritional and metabolic changes [14,15], and cell proliferation [38], even at the expense of accumulating more lipids.

The literature focussing on INSIG1 function in metabolic homeostasis has been inconclusive, probably due to the pathway's complexity. The direct involvement of *INSIG1* polymorphisms in regulating circulating TG and glucose levels is under debate [[51], [52], [53]], and previous studies suggested that the hepatocyte-specific deletion of *Insig1* or *Insig2* alone does not result in noticeable metabolic changes in the liver of healthy mice, requiring a double KO hit to observe metabolic dysfunction in the healthy liver [17]. Therefore, the specific role of INSIG1 in regulating hepatocellular metabolism and damage in the context of obesity, lipotoxic insults, and NASH was yet to be clearly determined. To mechanistically investigate whether deleting *Insig1* promotes liver damage or protection as well as its effect on other metabolic parameters, we used a whole-body Insig1 deficient animal model. Our lab previously showed that in murine obesity, *Insig1* is downregulated in adipose tissue. We posited that this response was part of an adaptive response promoting SREBP1 maturation, providing appropriate levels of fatty acid unsaturation and partially compensating the lipolytic and catabolic effect associated with insulin resistance [15] at the expense of facilitating the production and accumulation of lipids.

We challenged the *Insig1* KO mice with a high cholesterol/fat/refined sugar (Western) diet supplemented with sugar water for 12 weeks to maximally drive the hepatic lipogenic programme by activating both SREBP1C and ChREBP [54]. In the context of overnutrition, the *Insig1* KO mice showed a hyper-efficient activation of DNL and lipid remodelling programmes (Figure 2, Figure 4A). Despite this and in keeping with previous work from our laboratory and others [15,17], the *Insig1* KO mice had no differences in systemic metabolism or insulin resistance compared to their Het/WT littermates. However, the *Insig1* KO livers were protected from ER stress, oxidative damage and inflammation and exhibited reduced fibrogenesis (Figure 2I–O). Based on the transcriptional and lipidomic changes that we observed, we speculate that *Insig1* KO-mediated “lipo-protection” occurs via optimisation of lipid composition via enhanced production of MUFAs [55,56], other pleiotropic mechanisms that may include improved signalling of PPARs and their co-activator PGC1β [39,57], which were predicted as being upregulated in the *Insig1* KO mice (Figure 2, Figure 3, Figure 4). In our experimental setting, a significant role seemed to be played by the SREBP1-mediated upregulation of *Scd1* and other remodelling enzymes. Hepatic SCD1 plays a crucial role in preventing steatohepatitis by partitioning excess lipid into MUFA that can be safely stored in TGs and lipid droplets [56]. Furthermore, *Scd1* induction by ChREBP overexpression in the murine liver in conjunction with high-fat diet results in elevated MUFA, improved glucose tolerance, and insulin sensitivity despite the paradoxical increased hepatic steatosis; of relevance, this effect disappears upon SCD1 inhibition [28]. In contrast, *Scd1* deletion results in lowering of MUFA, induction of ER stress, acute inflammation, and macrophage recruitment [55]. Our data confirm that SREBP1 in the liver preferentially induces *Scd1*, thus resulting in accumulation of unsaturated fatty acids and providing similar protection from hepatic damage in the context of Western diet-like feeding. Intriguingly, we observed similar results when the *Insig1* WT/Het/KO mice were acutely challenged with CCl_4_, suggesting that the hepato-protection associated with INSIG1 suppression might not be limited to NASH and applicable to other acute and chronic liver diseases. To translate these findings back to human disease, we speculate that SREBPs activation in progressive human NASH likely reflects an attempt to metabolically adapt to nutritional surplus. The negative feedback loop induced by INSIG1 physiologically acts as a rheostat of the pathway; when excessively induced in the context of caloric excess, this feedback loop might restrain SREBPs and contribute to the failure of these equilibrium mechanisms [58].

This study has some limitations, such as the inability to conduct more comprehensive metabolically meaningful studies featuring advanced NASH: mice have a growth defect that becomes more prominent with age until adulthood, and as a result, in KO mice, length and BW progressively diverge with age. We also used a whole-body *Insig1* KO mouse that does not restrict the phenotype mechanistically to the role of INSIG1 in hepatocytes or other cell types. Thus, the observed phenotype may also be influenced by the effects of *Insig1* deletion in adipose tissue [15], immune cells [[59], [60], [61]], and hepatic stellate cells [62]. However, recapitulating a whole-body defect of DNL related to metabolic stress is a valid initial strategy to identify the potential relevance of this regulatory loop. It justifies and de-risks future studies focussed on the dissection of cell-specific mechanistic specificities. In this respect, these results represent a proof of concept that compensatory regulatory loops aiming to maintain lipid homeostasis provide a reasonable intellectual frame to devise combinatorial therapeutic strategies to inhibit effector mechanisms and promote resilience through potentiation of protective allostatic responses.

In conclusion, our study shows that activation and remodelling of lipid pathways in humans and animals in metabolic liver disease could be interpreted as a protective mechanism to limit liver damage and fibrosis. Our study provides actionable knowledge about the pathways and mechanisms involved in NAFLD pathogenesis, which may prove useful for developing new therapeutic strategies.

## Author contributions

VA, MV, AVP, and MEDA conceived and designed the experiments, analysed and interpreted the results, prepared the figures, and wrote the manuscript. MEDA, MV, and SED provided human clinical and histologic data and tissues. VA, MV, SV, SC, JL, and FO conducted the animal experiments. MV, SV, MD, IK, AA, and EP processed and analysed the human/murine NGS data. MV carried out the IPA analyses. ZH and JG processed and analysed the lipidomics data. VA, MV, and AL conducted the histology analyses with HALO. All of the authors provided useful criticism and revised and approved the manuscript.
