# Supplementary material for: Suppression of insulin-induced gene 1 (INSIG1) function promotes hepatic lipid remodelling and restrains NASH progression
Source: Mol Metab. 2021 Mar 17;48:101210. doi: 10.1016/j.molmet.2021.101210 (PMC8094910; doi:10.1016/j.molmet.2021.101210)
Supplement: Multimedia component 1 [file mmc1.pdf]

Suppression of insulin-induced gene 1 (INSIG1) function promotes hepatic lipid remodelling and restrains NASH progression

Vian Azzu [1,2,3,16], Michele Vacca [1,4,5,16], Ioannis Kamzolas [1,6], Zoe Hall [4,7], Jack Leslie [8], Stefania Carobbio [1], Sam Virtue [1], Susan E Davies [9], Agnes Lukasik [1], Martin Dale [1], Mohammad Bohlooly-Y [10], Animesh Acharjee [4,11], Daniel Lindén [12,13], Guillaume Bidault[1], Evangelia Petsalaki [6], Julian L. Griffin [4,7], Fiona Oakley [8], Michael ED Allison [$ 2], Antonio Vidal-Puig [$,1,14,15]

Institutions

[1] Wellcome Trust/MRC Institute of Metabolic Science, Metabolic Research Laboratories, University of Cambridge, Cambridge, UK

[2] Liver Unit, Cambridge NIHR Biomedical Research Centre, Cambridge University Hospitals NHS Foundation Trust, Cambridge, UK

[3] Department of Gastroenterology and Hepatology, Norfolk and Norwich University Hospitals, Norwich, UK

[4] Department of Biochemistry and Cambridge Systems Biology Centre, University of Cambridge, Cambridge, UK

[5] Clinica Medica Cesare Frugoni, Department of Interdisciplinary Medicine, University of Bari Aldo Moro, Bari, Italy

[6] European Molecular Biology Laboratory, European Bioinformatics Institute (EMBL-EBI), Wellcome Genome Campus, Hinxton, UK

[7] Biomolecular Medicine, Systems Medicine, Department of Metabolism, Digestion and Reproduction, Imperial College London, London, UK

[8] Newcastle Fibrosis Research Group, Biosciences Institute, Faculty of Medical Sciences, 5 Newcastle University, Newcastle upon Tyne, UK

[9] Department of Pathology, Cambridge University Hospitals, Cambridge, UK

[10] Translational Genomics, Discovery Sciences, BioPharmaceuticals R&D, AstraZeneca, Gothenburg, Sweden

[11] College of Medical and Dental Sciences, Institute of Cancer and Genomic Sciences, Centre for Computational Biology, University of Birmingham, UK

[12] Bioscience Metabolism, Research and Early Development Cardiovascular, Renal and Metabolism (CVRM), BioPharmaceuticals R&D, AstraZeneca, Gothenburg, Sweden

[13] Division of Endocrinology, Department of Neuroscience and Physiology, Sahlgrenska Academy, University of Gothenburg, Sweden

[14] Wellcome Trust Sanger Institute, Hinxton, UK

[15] Cambridge University Nanjing Centre of Technology and Innovation, Jiangbei, Nanjing, China

[*] Corresponding author. TVPlab, MRC Metabolic Diseases Unit, Metabolic Research Laboratories, University of Cambridge, Level 4WT/MRC Institute of Metabolic Science, Box 289, Addenbrooke Hospital, Hills Road, Cambridge, B2 0QQ, UK.

[**] Corresponding author. The Liver Unit, Cambridge University Hospitals, Box 210, CB2 0QQ, Cambridge, UK.

[16] Joint first authors.

SUPPLEMENTARY METHODS (continues from Methods)

S2.1 Biochemical serum analyses

Transaminases and metabolic biochemistry were measured using the Siemens Healthcare Dimension RXL analyser, or by Perkin Elmer DELFIA using reagents and calibrators purchased from Siemens. Free fatty acids were measured using the Roche Free Fatty Acid Kit (Code: 11383175001) modified to run in MicroTitre plate format. Insulin was measured using electrochemical luminescence immunoassay on the MesoScale Discovery immunoassay platform. Assays were run in duplicate. A minimum of two quality-control samples were run in each assay. All these measurements were performed by the Biochemistry Assay Lab (CBAL) of the Metabolic Research Laboratories, University of Cambridge.

S2.2 Murine tissue collection and processing

Murine tissues for protein or RNA extraction were frozen at the time of collection and stored at -80. Samples for histology were placed in 10% buffered formalin overnight before transfer to 70% ethanol and were embedded in paraffin 24h later (FFPE). Serial 4 μm sections were obtained from FFPE blocks to perform histology and immunohistochemistry (IHC).

S2.3 Histology

Sections were dried overnight at 37°C, dewaxed with xylene and 100% ethanol, and washed in running water for a minimum of 4 minutes. The sections were stained with standard haematoxylin and eosin (H&E) or Picrosirius red (PSR) histochemical stains. For PSR, picrosirius red stain (Pioneer Research Chemicals, PRC/R/109) was applied (1h); sections were rinsed in 1% acetic acid (in dH_2O) to "waterproof" the red staining and to remove excess. Fast Green 1% in dH_2O (Sigma, F7252-25G) was then incubated for 15s; excess stain was rinsed in 1% acetic acid in dH_2O (Fisher Scientific, A/0360/PB17) to remove excess stain. Sections were then dehydrated in alcohol, cleared in xylene, and mounted.

S2.4 Immunohistochemistry

After incubation overnight at 37°C, sections were dewaxed with xylene and industrial methylated spirits, washed in running water for a minimum 4 minutes, and kept hydrated in TBST. The sections then underwent the following steps: 1) (Optional) 25 minutes antigen

retrieval at 97.5°C (pH 6.0 - Vector H-3300, or pH 9.0 - Vector H-3301); 2) Washes in TBST; 3) Blocking step for endogenous peroxidase (5 minutes in DAKO Real Peroxidase Blocking solution, Cat S2023); 4) Multiple washes in TBST; 5) Blocking serum (Animal-Free Protein Block, Vector SP-5030) for 20-30 minutes; 6) Primary antibody incubation for 60 minutes at RT or ON at 4°C (PCNA, Abcam Cat: ab18197 – diluted 1:6000; αSMA, SIGMA Cat: A2547 – diluted 1: 100; CD3 [CD3-12], Abcam, Cat: ab11089 – diluted 1:800; CD45R [RA3-6B2], Abcam, Cat: ab64100 – diluted 1:1000; Ly6C/G, Abcam, Cat: ab2557 – diluted 1:100; F4/80 [CI:A3-1] Rt α Mse mAb, Bio-Rad, Cat: MCA497, diluted 1:20) all diluted in antibody diluent DAKO, Cat. S3022; 7) Multiple washes in TBST (5 minutes each); 8) Incubation for 30 minutes with polymers: MOM ImmPress Polymer Reagent (VECTOR, Cat: MP-2400) or ImmPRESS HRP Polymer (Vector, MP-7451); 9) Multiple washes in TBST; 10) DAB (5-10 minutes; VECTOR - PEROXIDASE SUBSTRATE KIT DAB, Cat: SK-5100; or, ImmPACT DAB, Vector SK-4105) prepared following the manufacturer's instruction; 11) Washes in TBST; 12) 1 minute incubation with DAKO REAL Haematoxylin (CAT: S2020). The sections where then washed in tap water, dehydrated in graded alcohols, cleared in xylene, and mounted.

S2.5 Tissue imaging, quantification and scoring

The tissue slides were scanned using a Zeiss AxioScan Z1 and analysed using HALO AI software (Indica Labs, Corrales, NM). The "Cytoplasmic & Nuclear IHC quantification" module was used to quantify nuclear stains (% of total nuclei stained with DAB), and inflammatory cells; nuclear roundness/area thresholds were used to distinguish the nuclei of the hepatocytes from the other cell types when needed. F4/80, αSMA, Cleaved caspase 3, and PSR staining (% stained area) were quantified using the "Area Quantification" module (vascular staining was excluded). The analyses were performed in the whole scanned section to avoid selection bias; tissue edges and vessels were excluded using appropriate tissue annotation using the "AI tissue classifier module" that utilises a state-of-the-art "artificial intelligence" learning algorithm to identify tissue types based on tissue texture and contextual features. HALO was "trained-by-example" on randomly selected images, and then the analysis was extended on the whole batch of sections with HALO's fully automated and unbiased pipeline. Images were checked for accuracy of annotation.

S2.6 RNA extraction and RNA integrity

Murine RNA was isolated using miRNAeasy Mini Kits (Qiagen), according to the manufacturer's instructions. Human biopsies' RNA was isolated using STAT-60 (AMS biotechnology, CS-502) according to the following procedure: 1) biopsies were homogenised in STAT-60 (1 ml) using a tissue homogeniser, mixed (vortexing) and centrifuged at 13,000g for 5 minutes at RT; 2) the supernatant was mixed (vortexing) with 200 μl chloroform (Sigma, Cat 650471) and centrifuged (12,000g) for 15 minutes at 4°C; 3) the supernatant was then mixed with 500 μl isopropanol (Sigma, cat 33539) and centrifuged at 10,000g for 10 minutes at 4°C to pellet the RNA; 4) the pellet was washed with 75% ethanol (1 ml) and allowed to dry until evaporation; 5) RNA was re-suspended in RNAse free water (Thermo Fisher Scientific, Delaware USA). All reagents, plastic ware, and supplies used were nuclease-free, sterile, and of molecular biology grade. RNA purity (A260/A280>1.80) and concentration were determined using the Nanodrop spectrophotometer (Thermo Fisher Scientific, Delaware USA). RNA integrity was studied using the 2100 Bioanalyzer (Agilent) and RNA 6000 Nano Kits (Agilent, Santa Clara, California, USA). RNA Integrity Number (RIN) of 7 or 8 was considered the lowest cut-off for RTqPCR and RNA sequencing, respectively.

S2.6 Reverse transcription (RT-) polymerase chain reaction (PCR)

cDNA was generated using the manufacturer's protocol (Reverse Transcriptase System, Promega). Briefly, 1000 ng RNA in 10 μl of RNAse free water was heated to 65°C for 5 minutes and placed on ice. 10 μl of RT mix [4 μl of M-MLV RT buffer (Promega M351A); 2 μl of 25mM $MgCl_2$ (Promega A351B); 2.5 μl nucleotide triphosphate (dNTP, Promega U151B); 0.5 μl (100 mg/ml) of random hexamers (Promega C118A); 1.0 μl of reverse transcriptase (Promega M170b)] was added and samples were incubated at 37°C for 1 hour. RT and RNA negative controls were also used, as well as a titration curve from a pool of cDNAs. Each sample was diluted 1:10 or 1:40 with RNAse-free water. A PCR reaction mix of 13 μl, composed of cDNA and primers, which were designed using Primer Express v3.0 and available upon request. RT PCR was performed using TaqMan or SYBR green (Thermo) in the following conditions: denaturation at 95°C for 10 min, followed by 40 cycles at 95°C for 15 seconds, then at 60°C for 60 seconds. Reactions were run in duplicate for each sample and quantified in the ABI QuantStudio 7 detection system (Applied Biosystems). Data were expressed as arbitrary units

and expression of target genes corrected to the geometric average of 3 housekeeping genes: 18S, 36b4 and Tbp (murine data) or 18S, CYCA, GUSB (human data).

S2.7 Whole-transcriptome amplification and RNA sequencing

RNA from tissues (1 μg RNA) were used to generate barcoded sequencing libraries using Illumina TruSeq® Stranded mRNA Library Preparation Kit (Illumina) following manufacturer's instructions. The sequencing libraries were normalised for concentration and combined into pools of 96-plex. The pooled libraries were sequenced on 3 lanes of an Illumina HiSeq 4000/6000 instrument at single-end 50bp (SE50), yielding an average of >15 million reads per sample. Library preparation was performed by the Genomics and Transcriptomic Core at the Institute of Metabolic Science. The sequencing was performed at the Genomics Core, Cancer Research UK Cambridge Institute.

S2.8 Hepatic lipid extraction and lipidomics

Thirty mg tissue was homogenised in chloroform/ methanol (2:1, 1 ml) using a TissueLyser (Qiagen Ltd., Manchester, UK). Deionised water (400 μl) was added before thoroughly mixing. The organic and aqueous layers were separated following centrifugation at 13,000g. The organic layer was dried under nitrogen and reconstituted in 2:1:1 isopropanol: acetonitrile: water for analysis by liquid chromatography mass spectrometry (LC-MS). LC-MS was performed on an Accela Autosampler coupled to LTQ Orbitrap Elite™ (Thermo Fisher Scientific, Hemel Hempstead, UK). Lipids were separated on an Acquity C18 BEH column (Waters Ltd., Wilmslow, UK) at 55°C. Mobile phase A was 60:40 acetonitrile: water and mobile phase B was 90:10 isopropanol: acetonitrile, each with 10 mM ammonium formate (positive ion mode) or 10 mM ammonium acetate (negative ion mode). A gradient flow at 0.5 ml/minutes was used, starting with 40 % B, to 99 % B over 8 min, held at 99 % B for 0.5 minutes and then return to starting conditions. The electrospray ionisation source was heated to 375˚C, desolvation temperature and gas flow were 380˚C and 40 arbitrary units, respectively. The analysis was performed in positive and negative ion modes (m/z 200 – 2000). Data were converted to mzML format and features picked using XCMS [1]. Peak areas were normalised to the appropriate internal standard of a mix of isotopically-labelled lipids covering the main lipid classes, and tissue weight. Lipid identification was performed by accurate mass matching using LIPID MAPS® database [2]. Abundance permitting, fatty acyl chain composition of

complex lipids was confirmed using collision induced dissociation and tandem mass spectrometry (fragmentation patterns are shown in Table S7).

For analyses, lipid species were classified as follows: SFAs were lipids with no double bonds, MUFAs were species with a maximum of 1 double bond per chain, PUFAs were species with 2 or more double bonds per chain.

DNL-like species contained SFA- or MUFA-containing fatty acyl chains of 16 and/or 18 carbons as previously described [3], thereby excluding any essential fatty acids. In WDSW these were TG(46:0), TG(46:1), TG(46:2) [12:0/16:1/18:1 & 14:0/16:1/16:1], TG(48:0), TG(48:1) [16:0/16:0/16:1], TG(48:2) [16:0/16:1/16:1 & 14:0/16:1/18:1], TG(48:3) [16:1/16:1/16:1TG], TG(49:0), TG(49:1), TG(49:2), TG(50:0), TG(50:1) [16:0/16:0/18:1], TG(50:2) [16:0/16:1/18:1], TG(50:3) [16:1/16:1/18:1], TG(51:0), TG(51:1), TG(51:2) [16:0/17:1/18:1 & 15:0/18:1/18:1], TG(52:0), TG(52:1) [16:0/18:0/18:1], TG(52:2) [16:0/18:1/18:1], TG(52:3) [16:1/18:1/18:1], TG(53:1), TG(53:2), TG(54:1) [18:0/18:0/18:1 & 16:0/18:1/20:0], and PC(32:0), PC(32:1), PC(34:1), PC(34:2), PC(36:1), PC(36:2). In CCl_4 these were TG(48:0), TG(48:1), TG(48:2), TG(50:1), TG(50:2), TG(50:3), TG(52:1), TG(52:2) and PC(32:1)/(32:0).

MUFA/SFA ratios were calculated for species with matching chain lengths then averaged. In WDSW these were TG[(46:1)+(46:2)]/(46:0), TG[(48:1)+(48:2)+(48:3)]/(48:0), TG(49:1)/(46:0), TG[(50:1)+(50:2)+(50:3)]/(50:0), TG[(51:1)+(51:2)]/(51:0), TG[(52:1)+(52:2)]/(52:0) and PC(32:1)/(32:0). In CCl_4 this was TG[(48:1)+(48:2)]/(48:0) and PC(32:1)/(32:0).

S2.9 Quantification and statistical analysis

All data are expressed as mean ± SEM. All analyses were performed using Graphpad Prism Statistical Package. P values ≤ 0.05 were considered significant. Pairwise comparison was by Student's T-test, and comparisons of 3+ groups was by analysis of variance (ANOVA) with Tukey's multiple comparison test or by non-parametric Kruskal-Wallis test with Dunn's multiple-comparison where appropriate [4].

S2.10 NGS data processing

RNA sequencing reads were aligned to the mouse GRCm38 genome or human GRCh38 genome respectively, using hisat2 (v 2.1.0) [5] (with the default parameters) and genes were counted using HTseq-count (v 0.11.1) [6]. The R package DESeq2 (v 1.26.0) [7] was then used to perform differential gene expression analysis (Wald Test) of the raw gene-level counts [7],

and the Benjamini-Hochberg method was applied to adjust the raw p values and control the False Discovery Rate (FDR).

S2.11 Bioinformatics functional analyses

After statistical analysis, differentially expressed genes within groups were studied using the Ingenuity Pathway Analysis (Qiagen). The whole transcriptome was uploaded In IPA; with the purpose of the enrichment analyses, genes were filtered for statistical significance ($p<0.05$) and fold change ($-0.3785 < Log_2FC > 0.3785$). “Upstream Regulators”, “Canonical Pathways” and “Biological Function” networks showing relationships and interactions, experimentally confirmed between differentially expressed genes and others that functionally interact with them, were generated and ranked in terms of the significance of participating genes and activation status (Z-score). A comparison analysis was then performed to focus only on those pathways significantly enriched in all the datasets of the same series (see figure legends for details). We considered “biologically relevant” only those genes which are statistically significant ($p<0.05$), with $-0.378 < Log_2FC > 0.378$, and enriched in “significantly modulated” pathways in the comparative analysis, and/or those genes with a $p_{bh}<0.05$

SUPPLEMENTARY REFERERENCES

[1] Smith, C.A., Want, E.J., O'Maille, G., Abagyan, R., Siuzdak, G., 2006. XCMS: processing mass spectrometry data for metabolite profiling using nonlinear peak alignment, matching, and identification. Anal Chem 78(3):779-787.

[2] Sud, M., Fahy, E., Cotter, D., Brown, A., Dennis, E.A., Glass, C.K., et al., 2007. LMSD: LIPID MAPS structure database. Nucleic Acids Res 35(Database issue):D527-532.

[3] Sanders, F.W.B., Acharjee, A., Walker, C., Marney, L., Roberts, L.D., Imamura, F., et al., 2018. Hepatic steatosis risk is partly driven by increased de novo lipogenesis following carbohydrate consumption. Genome Biol 19(1):79.

[4] Gibson-Corley, K.N., Olivier, A.K., Meyerholz, D.K., 2013. Principles for valid histopathologic scoring in research. Vet Pathol 50(6):1007-1015.

[5] Kim, D., Langmead, B., Salzberg, S.L., 2015. HISAT: a fast spliced aligner with low memory requirements. Nat Methods 12(4):357-360.

[6] Anders, S., Pyl, P.T., Huber, W., 2015. HTSeq--a Python framework to work with high-throughput sequencing data. Bioinformatics 31(2):166-169.

[7] Love, M.I., Huber, W., Anders, S., 2014. Moderated estimation of fold change and dispersion for RNA-seq data with DESeq2. Genome Biol 15(12):550.

SUPPLEMENTARY LEGENDS

Figure S1. NGS analysis predicts a strong regulation of metabolic pathways in patients with biopsy-proven NASH clustered against disease stage (continues from Figure 1). A) Gene expression profiled by NGS, analyzed with the Wald-Test (sample size defined in Table S1; # $p<0.05$; * $p_{BH}<0.05$), of key genes involved liver disease-associated pathways. B) Correlation of metabolic genes with hallmarks of systemic metabolic impairment. C) Correlation of metabolic vs disease-associated genes. Data are shown in a heat-map matrix format; details in the graphical legend.

Figure S2. *Insig1* ablation improves the lipidome and reduces cellular damage, inflammation and extracellular matrix deposition in 12 weeks of western diet supplemented with sugar water (continues from Figure 2). All data refer to *Insig1* WT/Het/KO animals treated with 12 weeks of WDSW. A) Body weight. B) Body fat composition as measured by TD-NMR. C) Liver weight/body weight ratio. Serum levels of glucose (D); TG (E); total cholesterol (F); free fatty acids (G). Calculation of peripheral/HOMA IR (H) and adipose tissue IR (I). Glucose tolerance test measured by time (J) and calculation of their respective area under the curve (AUC; K). L) % lipid content of livers as measured by Folch lipid extraction. M) Total hepatic TG content measured by LC-MS. N) Statistically significantly increased TG species in *Insig1* KO and Het versus WT as represented by carbon chain length and desaturation. O) Subclasses of lipids measured by LC-MS (FFA, free fatty acids; DG, diglycerides; TG, triglycerides; PC, phosphatidylcholines; LPC, lysophosphatidylcholines; PE, phosphoethanolamines; PI, phosphatidylinositols; Cer, ceramides; SM, sphingolmeylins; CE, cholesterol esters). P) Serum ALT levels of animals at 12 weeks of WDSW diet. Q) HALO imaging software analysis on scanned slides of whole-tissue of Ly6C/G IHC.

Figure S3. *Insig1* ablation improves the lipidome and reduces cellular damage and in animals treated with acute CCl_4 (continues from Figure 3). Serum ALT levels measured at 24h (A) and 96h (B) after challenge with virgin olive oil (VOO) or CCl_4. C) Body weight. Serum levels of TG (D); total cholesterol (E); glucose (F); insulin (G). Calculation of peripheral/HOMA IR (H). I) Statistically significantly increased TG species in *Insig1* KO versus WT as represented by carbon chain length and desaturation (there were no statistically significant Het vs WT hits).

J) Subclasses of lipids measured by LC-MS (FFA, free fatty acids; PC, phosphatidylcholines; LPC, lysophosphatidylcholines; PE, phosphoethanolamines; PI, phosphatidylinositols; Cer, ceramides; CE, cholesterol esters; SM, sphingolmeylins). K) % lipid content of livers as measured by Folch lipid extraction. L) Total hepatic TG content measured by LC-MS. HALO imaging software analysis on scanned slides of whole-tissue F4/80 stain (M) and αSMA IHC (N) of livers, with representative images shown. Data were analyzed by ANOVA (p-values < 0.05 are considered significant) with Tukey's post hoc test (a, reference group; groups with different letters are statistically different per post hoc comparison; differences between groups with the same letter are statistically not significant per post hoc comparison).

Figure S4. IPA Upstream regulator analysis (URA) of the transcriptome of the *Insig1* KO vs WT mice 4 days after CCl_4 challenge. Analysis of significantly ($P < 0.05$) enriched upstream regulators (UR) predicted to be activated/inhibited by IPA (on the basis of their Z-Score) was performed on differentially expressed genes. "Network-like" graphical representation of the interaction between a selection of upstream regulators (blue, down-regulated; orange, up-regulated) and their annotation into "Canonical Pathways (CP)" and "Disease and Biofunctions (D&BF)". Full list of modulated URs in Supporting File S5.

Figure S1

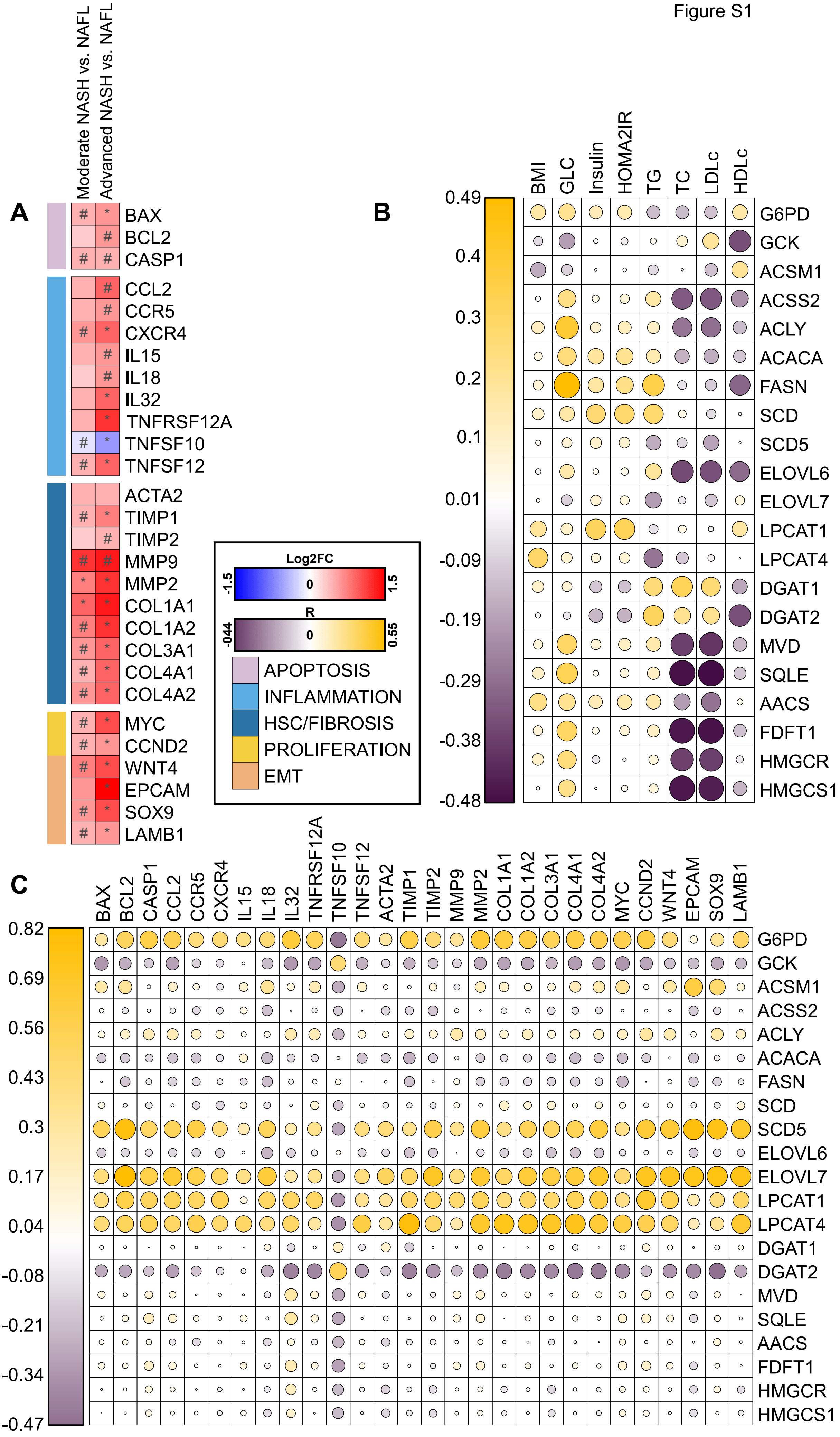
A
Moderate NASH vs. NAFL
Advanced NASH vs. NAFL
BAX
BCL2
CASP1
CCL2
CCR5
CXCR4
IL15
IL18
IL32
TNFRSF12A
TNFSF10
TNFSF12
ACTA2
TIMP1
TIMP2
MMP9
MMP2
COL1A1
COL1A2
COL3A1
COL4A1
COL4A2
MYC
CCND2
WNT4
EPCAM
SOX9
LAMB1
Log2FC
-1.5
0
1.5
R
-044
0
0.55
APOPTOSIS
INFLAMMATION
HSC/FIBROSIS
PROLIFERATION
EMT
B
BMI
GLC
Insulin
HOMA2IR
TG
TC
LDLc
HDLc
0.49
0.4
0.3
0.2
0.1
0.01
-0.09
-0.19
-0.29
-0.38
-0.48
G6PD
GCK
ACSM1
ACSS2
ACLY
ACACA
FASN
SCD
SCD5
ELOVL6
ELOVL7
LPCAT1
LPCAT4
DGAT1
DGAT2
MVD
SQLE
AACS
FDFT1
HMGCR
HMGCS1
C
0.82
0.69
0.56
0.43
0.3
0.17
0.04
-0.08
-0.21
-0.34
-0.47

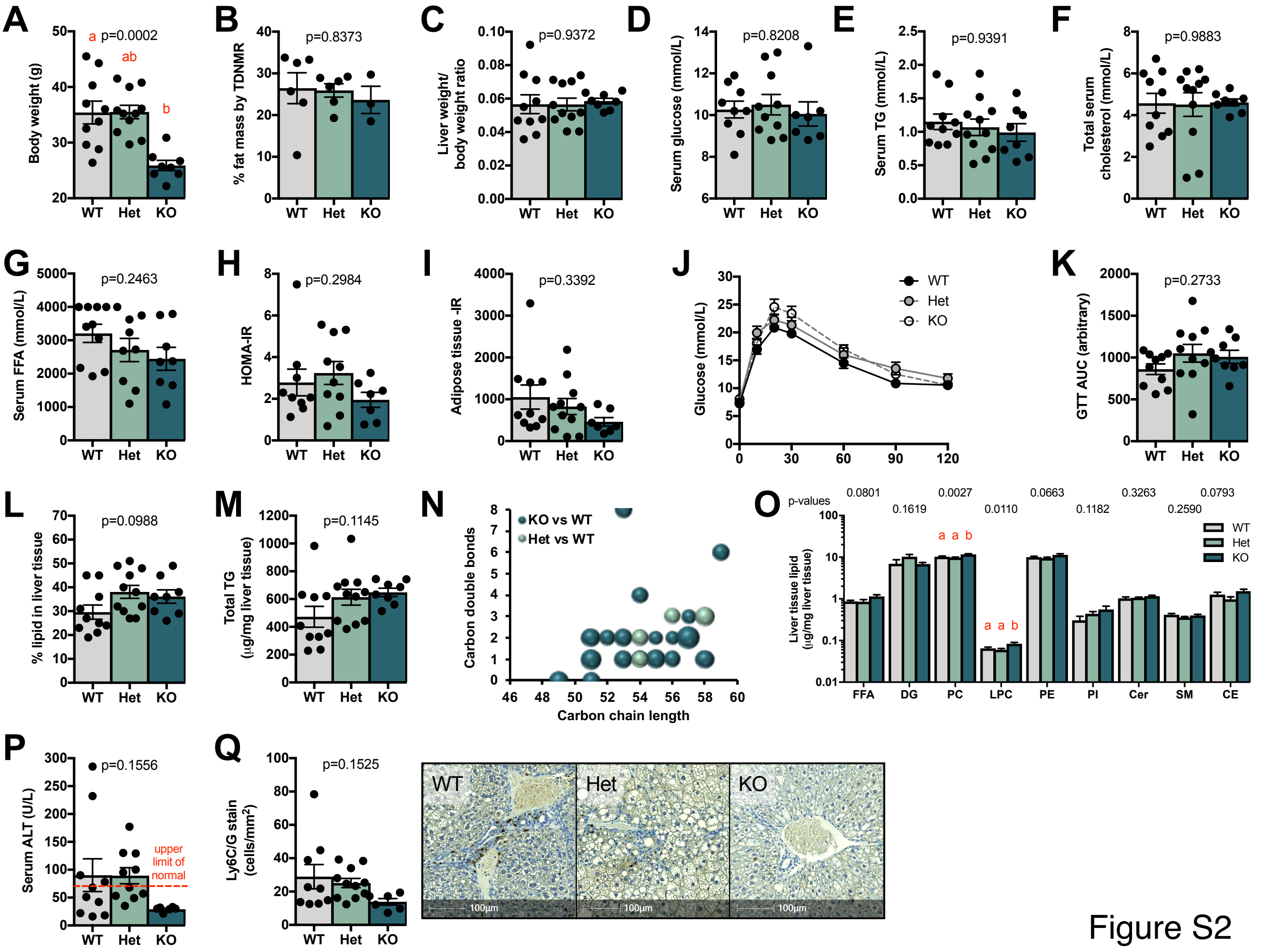

A
Body weight (g)
a p=0.0002
ab
b
B
% fat mass by TDNMR
p=0.8373
C
Liver weight/ body weight ratio
p=0.9372
D
Serum glucose (mmol/L)
p=0.8208
E
Serum TG (mmol/L)
p=0.9391
F
Total serum cholesterol (mmol/L)
p=0.9883
G
Serum FFA (mmol/L)
p=0.2463
H
HOMA-IR
p=0.2984
I
Adipose tissue -IR
p=0.3392
J
Glucose (mmol/L)
WT
Het
KO
K
GTT AUC (arbitrary)
p=0.2733
L
% lipid in liver tissue
p=0.0988
M
Total TG (µg/mg liver tissue)
p=0.1145
N
Carbon double bonds
KO vs WT
Het vs WT
Carbon chain length
O
p-values
0.0801
0.1619
0.0027
0.0110
0.0663
0.1182
0.3263
0.2590
0.0793
a a b
a a b
Liver tissue lipid (µg/mg liver tissue)
FFA
DG
PC
LPC
PE
PI
Cer
SM
CE
P
Serum ALT (U/L)
p=0.1556
upper limit of normal
Q
Ly6C/G stain (cells/mm²)
p=0.1525
WT
Het
KO
100µm

Figure S2

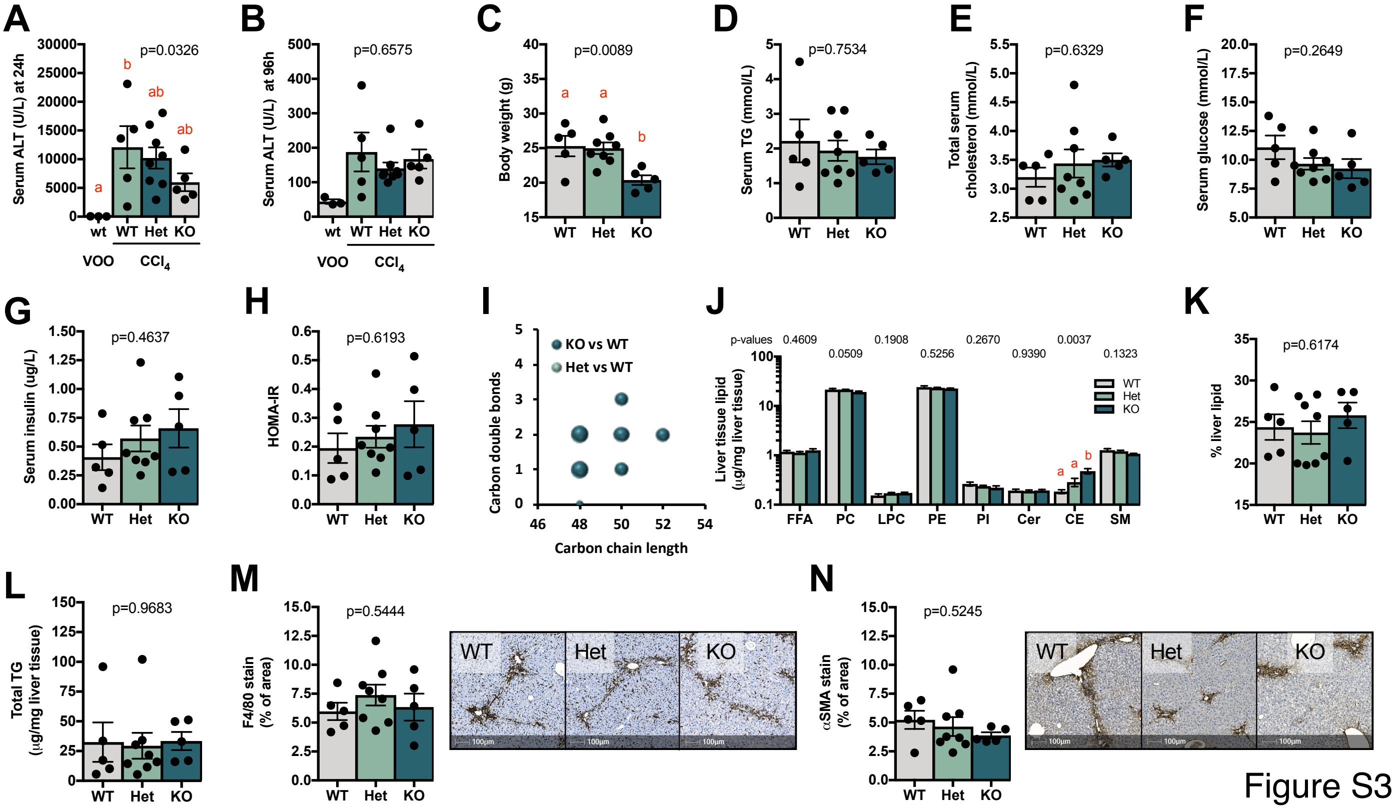
A
Serum ALT (U/L) at 24h
p=0.0326
wt WT Het KO
VOO CCl4
B
Serum ALT (U/L) at 96h
p=0.6575
C
Body weight (g)
p=0.0089
D
Serum TG (mmol/L)
p=0.7534
E
Total serum cholesterol (mmol/L)
p=0.6329
F
Serum glucose (mmol/L)
p=0.2649
G
Serum insulin (ug/L)
p=0.4637
H
HOMA-IR
p=0.6193
I
KO vs WT
Het vs WT
Carbon double bonds
Carbon chain length
J
p-values 0.4609 0.0509 0.1908 0.5256 0.2670 0.9390 0.0037 0.1323
Liver tissue lipid (µg/mg liver tissue)
FFA PC LPC PE PI Cer CE SM
K
% liver lipid
p=0.6174
L
Total TG (µg/mg liver tissue)
p=0.9683
M
F4/80 stain (% of area)
p=0.5444
N
αSMA stain (% of area)
p=0.5245

Figure S3

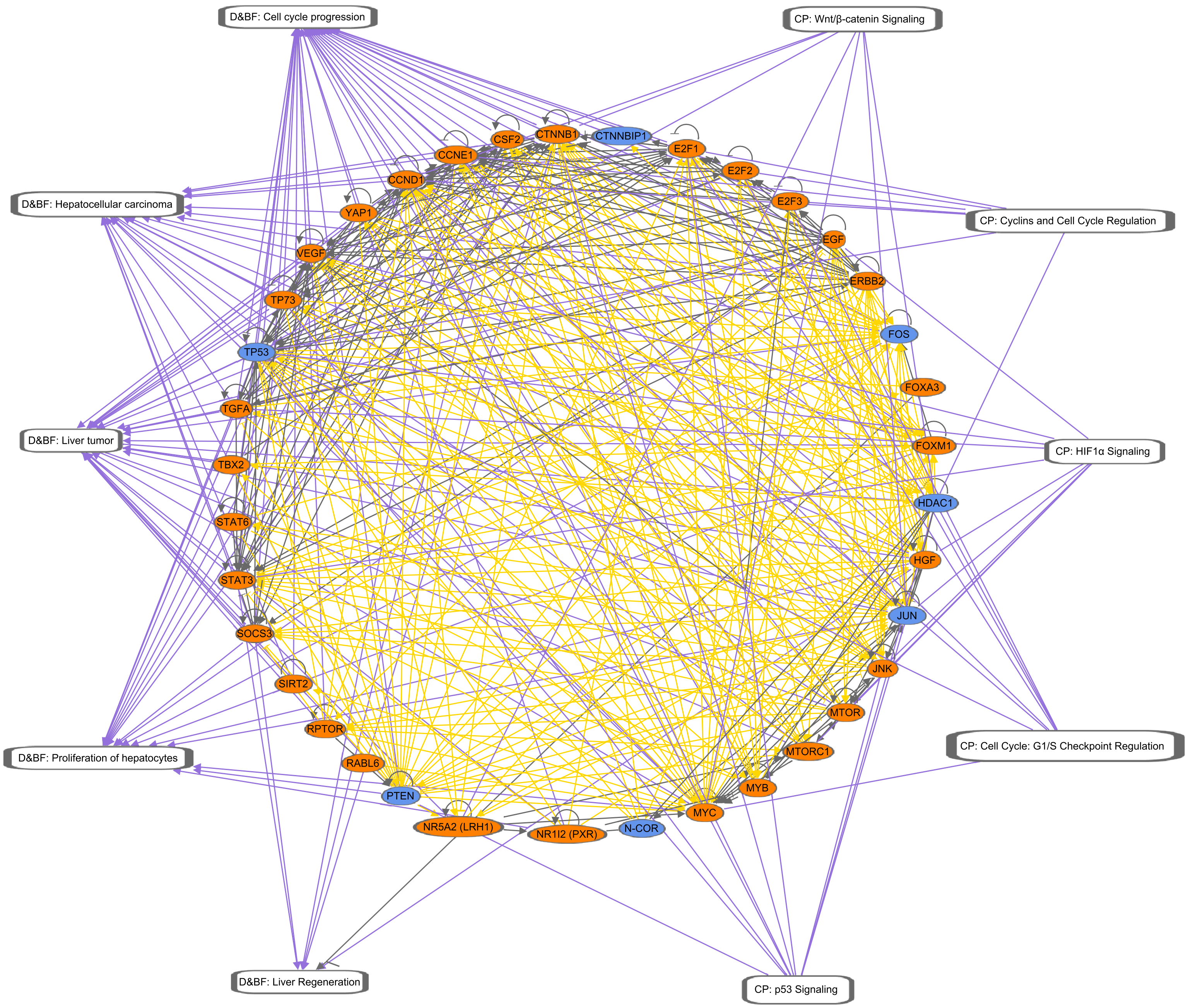

D&BF: Cell cycle progression
CP: Wnt/β-catenin Signaling
D&BF: Hepatocellular carcinoma
CP: Cyclins and Cell Cycle Regulation
D&BF: Liver tumor
CP: HIF1α Signaling
CP: Cell Cycle: G1/S Checkpoint Regulation
D&BF: Proliferation of hepatocytes
D&BF: Liver Regeneration
CP: p53 Signaling
CSF2
CTNNB1
CTNNBIP1
CCNE1
E2F1
E2F2
CCND1
E2F3
YAP1
EGF
VEGF
ERBB2
TP73
FOS
TP53
FOXA3
TGFA
FOXM1
TBX2
HDAC1
STAT6
HGF
STAT3
JUN
SOCS3
JNK
SIRT2
MTOR
RPTOR
MTORC1
RABL6
MYB
PTEN
MYC
NR5A2 (LRH1)
NR1I2 (PXR)
N-COR

Figure S4

Table S1: Patients clinical characteristics

	NAFL	NASH F0-2	NASH F3-4	P-Value (ANOVA)
**Patients (M/F)**	19 (12/7)	24 (14/10)	15 (11/4)	
**Age (years)**	54.42 ± 2.81	53.88 ± 2.34	56.73 ± 2.45	0.732
**BMI (Kg/m^2)**	32.73 ± 0.97[a]	36.74 ± 1.08[b]	32.34 ± 1.56[a]	**0.012**
**Glucose (mmol/L)**	6.85 ± 0.8	7.02 ± 0.53	7.6 ± 0.73	0.744
**Insulin (pmol/L)**	96.47 ± 9.92[a]	177.95 ± 29.55[b]	134.8 ± 19.56[a,b]	**0.041**
**HOMA2-IR**	1.91 ± 0.19[a]	3.43 ± 0.57[b]	2.63 ± 0.37[a,b]	**0.049**
**TG (mmol/L)**	1.82 ± 0.22	1.75 ± 0.11	1.78 ± 0.25	0.955
**Tot Cholesterol (mmol/L)**	4.53 ± 0.25	4.37 ± 0.23	4.42 ± 0.2	0.869
**LDL Cholesterol (mmol/L)**	2.68 ± 0.23	2.6 ± 0.2	2.52 ± 0.25	0.899
**HDL Cholesterol (mmol/L)**	1.05 ± 0.08	1 ± 0.05	1.05 ± 0.06	0.815
**AST (IU/L)**	41.25 ± 6.53	43.05 ± 3.56	61.83 ± 8.57	0.051
**ALT (IU/L)**	63.63 ± 6.62	73.83 ± 7.25	74.2 ± 8.52	0.529
**Steatosis**	1.21 ± 0.21[a]	2.04 ± 0.14[b]	1.8 ± 0.14 [a,b]	**0.002**
**Inflammation**	1 ± 0.17[a]	1.42 ± 0.1 [a,b]	1.67 ± 0.16[b]	**0.009**
**Ballooning**	0.21 ± 0.12[a]	1.12 ± 0.07[b]	1.33 ± 0.13[b]	**5.38e-10**
**NAS**	2.42 ± 0.3[a]	4.58 ± 0.22[b]	4.8 ± 0.26[b]	**2.49e-08**
**Fibrosis**	0.68 ± 0.13[a]	1.54 ± 0.1[b]	3.33 ± 0.13[c]	**<2e-16**

Statistical significance is assessed by ANOVA. Superscript letters indicate post-hoc analysis significance: "a" means reference group; when groups show different letters, they should be considered statistically different at the post-hoc comparison; groups showing the same letter are statistically not-significant at the post-hoc comparison.

Table S2. Canonical Pathways (CP), Upstream Regulators (UR) and Desearse and Functions (DF) significantly enriched in patients with NASH clustered against disease progression

A - Canonical Pathways	Moderate NASH VS NAFL	Advanced NASH VS NAFL
Dendritic Cell Maturation	2.530	4.583
Phospholipase C Signaling	2.646	4.491
CD28 Signaling in T Helper Cells	2.236	3.873
PKCθ Signaling in T Lymphocytes	2.000	3.742
Superpathway of Cholesterol Biosynthesis	2.828	3.606
IL-8 Signaling	0.000	3.578
GP6 Signaling Pathway	3.742	3.530
IL-6 Signaling	0.000	3.464
PDGF Signaling	0.000	3.464
Signaling by Rho Family GTPases	1.134	3.357
HOTAIR Regulatory Pathway	2.449	3.317
iCOS-iCOSL Signaling in T Helper Cells	2.000	3.317
PI3K Signaling in B Lymphocytes	0.000	3.317
B Cell Receptor Signaling	0.000	3.317
RANK Signaling in Osteoclasts	0.000	3.317
Integrin Signaling	2.000	3.207
STAT3 Pathway	2.000	3.162
PAK Signaling	2.000	3.162
Tec Kinase Signaling	2.000	3.051
Sperm Motility	0.000	3.051
HMGB1 Signaling	0.000	3.000
ILK Signaling	0.000	2.887
LPS-stimulated MAPK Signaling	0.000	2.887
NF-κB Signaling	0.000	2.840
GNRH Signaling	0.000	2.840
Cholesterol Biosynthesis I	2.000	2.828
Cholesterol Biosynthesis II (via 24,25-dihydrolanosterol)	2.000	2.828
Cholesterol Biosynthesis III (via Desmosterol)	2.000	2.828
NF-κB Activation by Viruses	0.000	2.714
Fc Epsilon RI Signaling	0.000	2.714
VEGF Family Ligand-Receptor Interactions	0.000	2.714
Gαq Signaling	0.000	2.714
14-3-3-mediated Signaling	0.000	2.714
Endothelin-1 Signaling	2.000	2.711
P2Y Purigenic Receptor Signaling Pathway	0.000	2.673
Role of NFAT in Regulation of the Immune Response	2.236	2.668
Systemic Lupus Erythematosus In B Cell Signaling Pathway	1.667	2.646
IL-1 Signaling	0.000	2.646
EGF Signaling	0.000	2.646
IL-2 Signaling	0.000	2.646
Estrogen-Dependent Breast Cancer Signaling	0.000	2.646
SPINK1 General Cancer Pathway	0.000	2.646
Th1 Pathway	0.000	2.646
B Cell Activating Factor Signaling	0.000	2.646
Paxillin Signaling	0.000	2.646
MIF Regulation of Innate Immunity	0.000	2.646
Cholecystokinin/Gastrin-mediated Signaling	0.000	2.530
Acute Phase Response Signaling	0.000	2.530
p70S6K Signaling	0.000	2.530
Chemokine Signaling	0.000	2.530
ErbB Signaling	0.000	2.530
Apelin Cardiomyocyte Signaling Pathway	0.000	2.530
HGF Signaling	0.000	2.530
CCR3 Signaling in Eosinophils	0.000	2.530
CXCR4 Signaling	0.000	2.496
IL-7 Signaling Pathway	0.000	2.496
Renin-Angiotensin Signaling	0.000	2.496
Apelin Liver Signaling Pathway	2.236	2.449
iNOS Signaling	0.000	2.449
Opioid Signaling Pathway	1.000	2.400
Neuroinflammation Signaling Pathway	1.000	2.353
Prolactin Signaling	0.000	2.333
Thrombopoietin Signaling	0.000	2.333
CCR5 Signaling in Macrophages	0.000	2.333
UVC-Induced MAPK Signaling	0.000	2.333
UVB-Induced MAPK Signaling	0.000	2.333
Thrombin Signaling	0.000	2.324
Th2 Pathway	0.000	2.309
Leukocyte Extravasation Signaling	2.333	2.236
Intrinsic Prothrombin Activation Pathway	2.236	2.236
Superpathway of Geranylgeranyldiphosphate Biosynthesis I (via Mevalonate)	2.000	2.236
Mevalonate Pathway I	2.000	2.236
Agrin Interactions at Neuromuscular Junction	0.000	2.236
Zymosterol Biosynthesis	0.000	2.236
Production of Nitric Oxide and Reactive Oxygen Species in Macrophages	0.000	2.138
Apelin Endothelial Signaling Pathway	0.000	2.138
Melanocyte Development and Pigmentation Signaling	0.000	2.121
April Mediated Signaling	0.000	2.121
Role of Pattern Recognition Receptors in Recognition of Bacteria and Viruses	0.000	2.121

Table S2. Canonical Pathways (CP), Upstream Regulators (UR) and Desearse and Functions (DF) significantly enriched in patients with NASH clustered against disease progression

A - Canonical Pathways	Moderate NASH VS NAFL	Advanced NASH VS NAFL
IL-3 Signaling	0.000	2.121
Calcium-induced T Lymphocyte Apoptosis	0.000	2.121
Sphingosine-1-phosphate Signaling	0.000	2.111
Role of NFAT in Cardiac Hypertrophy	0.000	2.065
IL-9 Signaling	0.000	2.000
4-1BB Signaling in T Lymphocytes	0.000	2.000
UVA-Induced MAPK Signaling	0.000	2.000
FcγRIIB Signaling in B Lymphocytes	0.000	2.000
IL-17A Signaling in Gastric Cells	0.000	2.000
Antioxidant Action of Vitamin C	0.000	-2.828
PPAR Signaling	0.000	-3.162

Table S2. Canonical Pathways (CP), Upstream Regulators (UR) and Desearse and Functions (DF) significantly enriched in patients with NASH clustered against disease progression

B-Upstream Regulators	Moderate NASH VS NAFL	Advanced NASH VS NAFL
TGFB1	4.990	6.785
Tgf beta	3.150	5.273
IL1B	2.472	5.265
SMARCA4	1.809	5.062
AGT	3.538	4.990
F2	3.512	4.914
CSF2	1.934	4.871
CTNNB1	3.029	4.810
IL6	2.329	4.763
CSF1	3.613	4.714
TNF	2.647	4.713
Vegf	3.330	4.650
CD44	3.000	4.611
IL5	1.255	4.557
MAP2K1	2.766	4.480
CG	2.816	4.426
GLI1	2.375	4.334
ERBB2	2.015	4.296
SP1	2.262	4.286
ERK	2.309	4.280
TCF7L2	0.000	4.248
TGFB3	2.727	4.205
CCR2	3.359	4.167
P38 MAPK	3.130	4.166
BRD4	3.281	4.164
NFkB (complex)	2.238	4.146
Interferon alpha	0.000	4.139
NORAD	2.828	4.123
SMAD3	1.658	4.098
STAT3	1.252	4.062
PRL	2.072	4.037
ETS1	1.342	4.015
EDN1	3.028	4.009
CD40LG	2.787	4.007
EGF	2.233	3.946
EGR2	0.896	3.944
SYVN1	0.000	3.873
Brd4	2.985	3.860
IFNG	-0.391	3.768
PGR	2.262	3.765
HIF1A	2.259	3.694
RAF1	0.000	3.687
TNFSF11	2.139	3.685
IL2	2.374	3.684
IL3	0.000	3.652
SREBF2	3.097	3.610
CD38	1.265	3.561
HGF	1.418	3.560
AR	2.198	3.551
Ige	0.000	3.516
EGR1	3.076	3.504
WNT1	1.937	3.501
MKNK1	2.781	3.484
NRG1	2.435	3.474
SH3TC2	3.000	3.464
PI3K (complex)	2.198	3.461
SREBF1	3.056	3.448
FOXM1	1.703	3.404
TCF4	1.709	3.394
VEGFA	2.038	3.388
CHUK	2.218	3.362
CCL2	2.208	3.349
RETNLB	2.121	3.317
FEV	0.000	3.317
F3	2.162	3.269
PRKCD	1.756	3.257
F2R	2.178	3.253
Tnf (family)	2.575	3.245
MTPN	2.400	3.208
ERK1/2	3.208	3.162
TET2	0.000	3.153
SOX4	0.000	3.144
SCAP	3.104	3.140
ROCK1	2.646	3.138
MITF	0.000	3.133
MYD88	0.000	3.128
OSM	1.102	3.114
SRC	1.758	3.102
SMAD2	1.701	3.088

B-Upstream Regulators	Moderate NASH VS NAFL	Advanced NASH VS NAFL
MAP3K1	2.005	3.087
Jnk	2.758	3.040
YAP1	1.987	3.035
BMP2	2.639	3.030
FGF2	2.890	3.025
PTH	2.403	3.018
AKT1	1.201	3.004
BMP4	0.000	3.003
STAT1	0.000	2.999
PLAG1	0.000	2.985
Collagen type I (complex)	2.219	2.970
LEP	1.890	2.969
TGFBR1	2.219	2.942
TP53	0.796	2.935
RAC1	2.586	2.933
MAP2K1/2	1.964	2.932
IL13	1.812	2.928
NFKB1	1.180	2.914
IL1A	2.074	2.894
ERG	2.192	2.892
RELA	0.864	2.890
IGF1	3.105	2.882
CD40	1.440	2.860
WNT3A	1.112	2.836
BCR (complex)	0.892	2.820
Ins1	2.649	2.816
KLF4	2.472	2.816
Calcineurin A	1.000	2.813
SEMA7A	0.000	2.813
TLR4	0.000	2.808
CAMP	0.000	2.805
TAZ	1.980	2.795
SOX11	1.710	2.784
NEDD9	0.000	2.781
JAK2	0.000	2.781
MEF2D	-0.218	2.778
IL18	0.000	2.777
ATF4	0.000	2.768
RELB	0.000	2.759
NFKB2	0.000	2.755
HTT	2.348	2.751
CCN1	1.982	2.742
PDGFB	2.200	2.731
IL1	2.045	2.729
SRF	1.593	2.720
IRF7	0.000	2.701
MAPK3	1.151	2.694
SIRT2	2.449	2.646
C4BP	2.236	2.646
SGK1	0.000	2.646
POU2F2	0.000	2.630
INSR	2.937	2.624
FGF10	0.000	2.621
USF1	0.000	2.621
CCN2	1.945	2.607
IL17A	2.542	2.597
Ap1	1.980	2.595
ITGB3	1.307	2.593
IL6R	0.000	2.588
FN1	1.672	2.586
PDGF BB	2.380	2.585
C5	0.000	2.578
EPHB4	0.000	2.570
GDF2	0.756	2.562
Mek	2.385	2.551
NTRK2	1.387	2.547
ATP7B	1.667	2.530
SMAD4	0.985	2.523
Insulin	2.921	2.507
ADCYAP1	1.461	2.498
INS	1.406	2.474
NFAT5	0.659	2.458
SMARCB1	1.326	2.456
TLR7/8	0.000	2.449
ATF2	0.000	2.435
IKBKB	2.092	2.426
EPAS1	-0.284	2.424
TGM2	2.348	2.423
IL4	1.805	2.416

Table S2. Canonical Pathways (CP), Upstream Regulators (UR) and Desearse and Functions (DF) significantly enriched in patients with NASH clustered against disease progression

B-Upstream Regulators	Moderate NASH VS NAFL	Advanced NASH VS NAFL
LCK	0.000	2.412
MAP2K4	0.000	2.407
PRKD1	0.000	2.400
PDGFC	0.000	2.394
AVP	0.000	2.392
VTN	1.400	2.391
SHH	0.074	2.387
REL	0.000	2.385
FGFR1	1.446	2.377
TLR9	0.000	2.367
TRAF3IP2	1.982	2.360
EGFR	1.522	2.360
TGFA	0.000	2.354
MRTFB	2.219	2.350
STAT5a/b	0.000	2.345
ANGPT2	1.304	2.343
NOTCH1	1.408	2.314
Nfat (family)	2.333	2.309
FOXO1	-0.021	2.302
Creb	1.390	2.300
ITGAV	0.000	2.282
RUNX2	1.185	2.265
TNC	0.000	2.259
IGFBP2	2.580	2.236
SMARCD3	2.236	2.236
TBXT	2.236	2.236
VGLL3	2.000	2.236
HRG	0.000	2.236
EDNRA	0.000	2.236
SKIL	0.000	2.236
SELPLG	0.000	2.236
MYOC	0.000	2.236
JUN	1.622	2.231
IRF1	0.000	2.223
MUC1	0.000	2.221
HBEGF	0.000	2.219
Il8r	0.000	2.219
TCF7	0.000	2.216
MIF	0.503	2.215
Smad2/3-Smad4	1.980	2.213
TP53COR1	0.000	2.213
Complement	0.000	2.213
CCL5	2.000	2.208
TWIST1	1.414	2.208
GNAQ	0.000	2.208
CSF2RB	0.000	2.207
POU2AF1	0.000	2.201
FOXL2	0.849	2.200
ETV4	0.000	2.194
IPMK	0.000	2.191
LTA	0.000	2.183
CYP27A1	0.000	2.183
MAP3K8	0.000	2.180
S1PR2	0.000	2.180
MMP1	0.000	2.178
EIF2AK2	0.000	2.177
CAMK4	0.000	2.173
APP	0.859	2.168
CCR1	0.000	2.138
TGFB2	1.307	2.136
AGER	1.913	2.132
JAG1	0.842	2.132
TEAD1	0.000	2.121
TEAD3	0.000	2.121
PARPBP	0.000	2.121
TEAD2	0.000	2.121
RPTOR	3.138	2.114
SPP1	1.474	2.090
CXCL8	0.000	2.073
POU5F1	2.562	2.065
TP63	0.282	2.061
LDL	1.994	2.043
NOTCH3	0.507	2.041
BTK	0.000	2.035
NOTCH2	1.724	2.034
CEBPB	1.216	2.026
MYB	0.924	2.024
PLG	0.000	2.020
IL2RG	0.000	2.016

Table S2. Canonical Pathways (CP), Upstream Regulators (UR) and Desearse and Functions (DF) significantly enriched in patients with NASH clustered against disease progression

B-Upstream Regulators	Moderate NASH VS NAFL	Advanced NASH VS NAFL
INHBA	1.398	2.006
POMC	0.666	2.003
HOXD3	2.000	2.000
CPXM1	2.000	2.000
Srebp	0.000	2.000
NSD2	0.000	2.000
RAPGEF4	0.000	2.000
ETV1	0.000	2.000
NPPC	0.000	2.000
USP22	0.000	2.000
RAPGEF3	0.000	2.000
CDC42	0.000	2.000
Pdgf (complex)	2.544	1.927
Akt	2.047	1.864
CXCL12	2.177	1.796
PTGS2	2.189	1.625
KLF5	2.111	1.362
IRS1	2.297	0.854
let-7	-2.734	0.000
TLR7	2.407	0.000
IL1RN	-2.236	0.000
PRMT5	2.176	0.000
BRCA1	2.220	-0.100
NKX2-3	2.224	-0.101
YY1	2.177	-0.717
FBXW7	-2.364	-0.730
MYC	-2.849	-0.762
NR4A1	-2.093	-1.108
DICER1	-2.720	-1.263
IKZF3	-2.228	-1.425
PPIF	-2.449	-1.667
HNF1B	-2.449	-1.713
MFSD2A	-2.000	-1.913
DSP	-1.982	-2.000
CTNNBIP1	0.000	-2.000
miR-103-3p (and other miRNAs w/seed GCAGCAU)	0.000	-2.000
miR-21-5p (and other miRNAs w/seed AGCUUAU)	0.000	-2.004
ZFP36	0.000	-2.007
mir-21	0.307	-2.015
SP110	-2.121	-2.111
NR1H4	0.000	-2.112
APOE	-1.981	-2.141
GFI1	0.000	-2.159
MDM4	0.000	-2.190
mir-30	0.000	-2.200
mir-150	0.000	-2.200
FBN1	-2.207	-2.204
NCOR-LXR-Oxysterol-RXR-9 cis RA	0.000	-2.219
miR-182-5p (and other miRNAs w/seed UUGGCAA)	0.000	-2.219
ADAMTS12	0.000	-2.236
EFNA5	0.000	-2.236
ARHGDIG	0.000	-2.236
CORT	0.000	-2.236
EFNA3	0.000	-2.236
SPTLC2	0.000	-2.236
EFNA4	0.000	-2.236
CYP51A1	0.000	-2.236
IFNB1	-0.732	-2.295
RXRA	-1.383	-2.325
ACOX1	-0.816	-2.333
CIP2A	-1.980	-2.359
MYCN	-2.829	-2.376
mir-29	-1.203	-2.377
miR-145-5p (and other miRNAs w/seed UCCAGUU)	0.000	-2.394
miR-200b-3p (and other miRNAs w/seed AAUACUG)	0.000	-2.409
mir-185	0.000	-2.433
ZNF217	0.000	-2.433
GLIS2	0.000	-2.449
CUL3	0.000	-2.449
TSC2	-1.633	-2.490
NPPB	-2.619	-2.573
MEOX2	0.000	-2.598
mir-19	0.000	-2.599
miR-34a-5p (and other miRNAs w/seed GGCAGUG)	-1.944	-2.602
IFT88	-1.368	-2.613
miR-17-5p (and other miRNAs w/seed AAAGUGC)	0.000	-2.615
CAT	0.000	-2.621
LMNB1	0.000	-2.630
PAX5	0.000	-2.630

B-Upstream Regulators	Moderate NASH VS NAFL	Advanced NASH VS NAFL
AURK	0.000	-2.646
ANLN	0.000	-2.646
AHR	-3.094	-2.650
COL18A1	-1.478	-2.745
TAF4	-1.982	-2.784
FAS	-2.846	-2.786
miR-141-3p (and other miRNAs w/seed AACACUG)	0.000	-2.789
mir-133	0.000	-2.813
mir-122	-1.980	-2.815
TFRC	-2.449	-2.874
KIF3A	-2.592	-2.945
miR-199a-5p (and other miRNAs w/seed CCAGUGU)	-1.387	-2.950
CDK19	-2.000	-2.985
INSIG1	-3.257	-3.034
EGLN	0.000	-3.053
miR-1-3p (and other miRNAs w/seed GGAAUGU)	0.000	-3.122
ABCB4	-2.219	-3.138
HDL-cholesterol	-1.633	-3.317
RUNX3	-1.937	-3.391
SPDEF	-2.828	-3.413
CR1L	-3.162	-3.426
POR	-2.218	-3.494
miR-29b-3p (and other miRNAs w/seed AGCACCA)	-2.462	-3.519
ADRB	-2.795	-3.578
Nr1h	-0.707	-3.842
let-7a-5p (and other miRNAs w/seed GAGGUAG)	-2.414	-3.873
PPARGC1A	-2.670	-3.901
SMAD7	-2.777	-3.977
Alpha catenin	-2.945	-4.398
IKZF1	-2.732	-4.522

Table S2. Canonical Pathways (CP), Upstream Regulators (UR) and Desearse and Functions (DF) significantly enriched in patients with NASH clustered against disease progression

C-Diseases and Bio Functions	Moderate NASH VS NAFL	Advanced NASH VS NAFL
Cell survival	2.685	7.298
Cell viability	2.271	7.072
Size of body	4.450	6.052
Cell movement	3.161	6.001
Migration of cells	2.816	5.552
Quantity of mononuclear leukocytes	1.764	5.171
Quantity of lymphatic system cells	1.472	5.081
Quantity of cells	2.110	5.037
Quantity of lymphocytes	1.683	4.991
Organization of cytoplasm	2.301	4.938
Organization of cytoskeleton	2.301	4.938
Migration of mononuclear leukocytes	3.107	4.917
Migration of tumor cell lines	2.029	4.853
Fibrogenesis	0.000	4.813
Microtubule dynamics	2.124	4.785
Immune response of leukocytes	2.354	4.780
Immune response of cells	2.520	4.745
Activation of cells	2.575	4.689
Formation of filaments	2.795	4.633
Lymphocyte migration	3.049	4.632
Cell movement of tumor cell lines	2.455	4.611
Migration of lymphatic system cells	2.851	4.603
Formation of cellular protrusions	0.000	4.579
Binding of leukocytes	0.000	4.522
Binding of mononuclear leukocytes	0.000	4.502
Binding of blood cells	0.000	4.484
Adhesion of blood cells	0.000	4.461
Mobilization of Ca2+	0.000	4.396
Adhesion of mononuclear leukocytes	0.000	4.366
Chemotaxis	2.376	4.354
Adhesion of immune cells	0.000	4.304
Homing of cells	1.973	4.281
Quantity of leukocytes	1.869	4.280
Invasion of tumor cell lines	1.614	4.268
Cell-cell contact	2.444	4.250
Cell movement of blood cells	0.000	4.238
Growth of tumor	1.474	4.220
Formation of cytoskeleton	2.868	4.187
Lymphopoiesis	0.000	4.172
Cell movement of mononuclear leukocytes	2.505	4.063
Leukocyte migration	4.152	4.057
Recruitment of cells	3.694	3.999
Development of cytoplasm	2.280	3.971
Invasion of cells	1.398	3.971
Cell movement of leukocytes	3.426	3.959
Formation of muscle	2.191	3.901
Hematopoiesis of mononuclear leukocytes	0.000	3.898
Leukopoiesis	1.173	3.882
Quantity of blood cells	1.326	3.850
Formation of actin filaments	0.000	3.838
Cell movement of lymphatic system cells	2.218	3.831
Cell spreading	0.000	3.823
Vasculogenesis	2.025	3.821
Recruitment of blood cells	0.000	3.799
Angiogenesis	1.690	3.779
Development of vasculature	1.690	3.779
Aggregation of cells	0.000	3.770
Cell movement of lymphocytes	2.270	3.739
Cellular homeostasis	0.000	3.708
Recruitment of leukocytes	3.289	3.636
Phosphorylation of protein	3.275	3.550
Proliferation of lymphatic system cells	0.000	3.452
Cell movement of breast cancer cell lines	0.000	3.449
Activation of blood cells	2.228	3.267
Growth of smooth muscle	0.966	3.254
Proliferation of mononuclear leukocytes	0.000	3.250
Growth of lymphoid organ	0.000	3.229
Proliferation of lymphocytes	0.000	3.201
Stimulation of cells	0.000	3.157
Quantity of metal ion	0.000	3.100
Cell movement of phagocytes	2.077	3.047
Proliferation of immune cells	0.000	3.030
Cell movement of myeloid cells	2.602	3.024
Proliferation of neuronal cells	0.000	2.972
Proliferation of blood cells	0.000	2.968
Quantity of metal	0.000	2.937
Cell proliferation of T lymphocytes	0.000	2.933
Cell movement of endothelial cells	-0.357	2.858
Quantity of Ca2+	2.518	2.857

Table S2. Canonical Pathways (CP), Upstream Regulators (UR) and Desearse and Functions (DF) significantly enriched in patients with NASH clustered against disease progression

C-Diseases and Bio Functions	Moderate NASH VS NAFL	Advanced NASH VS NAFL
Development of body trunk	0.000	2.793
Outgrowth of cells	0.000	2.781
Quantity of hematopoietic progenitor cells	0.896	2.742
Cell proliferation of tumor cell lines	1.949	2.727
Migration of endothelial cells	0.000	2.719
Endothelial cell development	1.805	2.703
Blood clot	1.829	2.702
Development of neurons	0.000	2.696
Cell movement of antigen presenting cells	0.332	2.683
Arteriosclerosis	0.000	2.657
Growth of malignant tumor	0.000	2.624
Proliferation of tumor cells	0.000	2.615
Cell movement of smooth muscle cells	1.658	2.607
Growth of connective tissue	0.969	2.571
Thrombus	1.604	2.552
Development of endothelial tissue	1.798	2.507
Development of epithelial tissue	1.567	2.475
Proliferation of cancer cells	0.000	2.474
Synthesis of lipid	1.843	2.470
Branching of cells	0.000	2.427
Cell movement of muscle cells	1.658	2.415
Leukemia	0.728	2.412
Neuritogenesis	0.000	2.399
Morphogenesis of neurons	0.000	2.399
Binding of connective tissue cells	0.000	2.325
Vaso-occlusion	0.000	2.244
Myeloid neoplasm	0.000	2.242
Myeloid neoplasm	0.000	2.242
Cell movement of macrophages	0.471	2.240
Benign oral disorder	0.000	2.207
Malignant myeloid neoplasm	0.000	2.207
Acute myeloid leukemia	0.000	2.207
Benign oral disorder	0.000	2.207
Malignant myeloid neoplasm	0.000	2.207
Acute myeloid leukemia	0.000	2.207
Proliferation of endothelial cells	1.510	2.190
Liquid tumor	1.091	2.176
Occlusion of artery	0.000	2.173
Occlusion of artery	0.000	2.173
Development of genital tumor	0.762	2.159
Sprouting	0.000	2.147
Proliferation of connective tissue cells	0.688	2.001
Growth Failure	-3.910	0.000
Growth failure or short stature	-3.910	0.000
Engulfment of cells	3.595	0.000
Endocytosis	3.588	0.000
Recruitment of phagocytes	3.534	0.000
Endocytosis by eukaryotic cells	3.304	0.000
Recruitment of myeloid cells	3.303	0.000
Hypoplasia	-3.233	0.000
Aplasia or hypoplasia	-3.233	0.000
Attachment of cells	3.206	0.000
Perinatal death	-2.695	0.000
Differentiation of nervous system	2.573	0.000
Growth of liver tumor	2.572	0.000
Migration of phagocytes	2.536	0.000
T cell migration	2.463	0.000
Limb defect	-2.436	0.000
Organ Degeneration	-2.356	0.000
Recruitment of granulocytes	2.307	0.000
Congenital anomaly of digit	-2.300	0.000
Recruitment of neutrophils	2.296	0.000
Cell movement of T lymphocytes	2.291	0.000
Response of myeloid leukocytes	2.271	0.000
Allergy	2.260	0.000
Experimentally induced inflammation	2.219	0.000
Hereditary myopathy	-2.211	0.000
Tensile strength of skin	2.200	0.000
Immediate hypersensitivity	2.194	0.000
Metabolism of membrane lipid derivative	2.170	0.000
Differentiation of embryonic cells	2.153	0.000
Migration of myeloid cells	2.059	0.000
Response of granulocytes	2.058	0.000
Binding of tumor cell lines	2.057	0.000
Abnormal bone density	-2.021	0.000
Formation of vessel	2.000	0.000
Growth Failure	-3.910	0.000
Growth failure or short stature	-3.910	0.000
Recruitment of phagocytes	3.534	0.000

Table S2. Canonical Pathways (CP), Upstream Regulators (UR) and Desearse and Functions (DF) significantly enriched in patients with NASH clustered against disease progression

C-Diseases and Bio Functions	Moderate NASH VS NAFL	Advanced NASH VS NAFL
Hypoplasia	-3.233	0.000
Aplasia or hypoplasia	-3.233	0.000
Growth of liver tumor	2.572	0.000
Migration of phagocytes	2.536	0.000
Limb defect	-2.436	0.000
Organ Degeneration	-2.356	0.000
Congenital anomaly of digit	-2.300	0.000
Recruitment of neutrophils	2.296	0.000
Allergy	2.260	0.000
Experimentally induced inflammation	2.219	0.000
Hereditary myopathy	-2.211	0.000
Immediate hypersensitivity	2.194	0.000
Abnormal bone density	-2.021	0.000
Benign lesion	-0.938	-2.133
Colon tumor	0.000	-2.178
Apoptosis	-0.462	-2.188
Motor dysfunction or movement disorder	0.000	-2.194
Gastrointestinal tumor	0.000	-2.311
Hereditary connective tissue disorder	-1.484	-2.344
Congenital malformation of genitourinary system	-1.342	-2.553
Lung adenocarcinoma	0.000	-2.630
Non-small cell lung carcinoma	0.000	-2.630
Colorectal disorder	0.021	-2.689
Development of lung tumor	0.000	-2.767
Bleeding	-2.833	-5.526
Organismal death	-6.093	-9.443
Morbidity or mortality	-6.263	-9.494

Table S3 List of genes singificantly modulated in both Het KO (vs. WT) and with same pattern of regulation (WDSW experiment)

		Het vs WT				KO vs WT			
**Ensembl**	**Symbol**	**Log2CPM**	**Log2FC**	**p-value**	**FDR**	**Log2CPM**	**Log2FC**	**p-value**	**FDR**
ENSMUSG00000032561	ACPP	3.64	1.692	0.0000276	0.00198	2.929	2.282	0.000111	0.0224
ENSMUSG00000078686	Mup1 (includes others)	0.671	2.641	0.0000758	0.00341	0.502	2.08	0.000101	0.0211
ENSMUSG00000030364	Clec2e/Clec2h	0.966	1.37	0.0341	0.144	1.324	2.03	0.0052	0.136
ENSMUSG00000035686	THRSP	9.03	1.237	0.000223	0.00692	8.999	1.674	8.53E-07	0.0016
ENSMUSG00000027075	SLC43A1	1.981	1.297	0.00000113	0.000238	1.269	1.619	0.000202	0.0285
ENSMUSG00000018566	SLC2A4	1.189	1.404	0.00000769	0.000848	0.756	1.611	0.000338	0.0374
ENSMUSG00000026839	UPP2	7.363	0.987	0.00411	0.0411	7.107	1.609	0.000238	0.0321
ENSMUSG00000006638	Abhd1	1.94	1.484	0.00293	0.0337	1.922	1.52	0.0164	0.225
ENSMUSG00000018868	PNPLA5	2.529	0.827	0.000453	0.0108	2.514	1.501	0.00000287	0.0034
ENSMUSG00000027533	FABP5	4.822	0.805	0.0469	0.172	4.831	1.477	0.00382	0.115
ENSMUSG00000090622	A930033H14Rik	1.524	1.161	0.00999	0.0705	1.498	1.393	0.00142	0.0681
ENSMUSG00000030256	Bhlhe41	1.557	1.168	0.0441	0.167	1.133	1.356	0.032	0.299
ENSMUSG00000050069	GREM2	2.814	0.67	0.0138	0.0858	2.884	1.326	0.000456	0.0413
ENSMUSG00000078597	CYP4A22	4.98	1.343	0.00146	0.0214	4.723	1.32	0.00578	0.142
ENSMUSG00000074280	Gm6166	1.474	0.639	0.0159	0.0927	1.618	1.273	0.00101	0.0578
ENSMUSG00000115919	Gm31583	3.945	1.465	0.000706	0.0142	3.368	1.251	0.0114	0.193
ENSMUSG00000073835	Mup-ps12	3.655	1.448	0.000394	0.00993	3.375	1.222	0.0143	0.213
ENSMUSG00000084839	Gm14097	1.918	1.321	0.000104	0.00412	1.667	1.2	0.0105	0.184
ENSMUSG00000027346	GPCPD1	6.158	1.029	0.0000472	0.00267	5.773	1.198	0.000617	0.0473
ENSMUSG00000075543	URAD	3.391	0.836	0.000693	0.0141	3.226	1.187	0.000121	0.023
ENSMUSG00000095532	Mup-ps13	8.358	1.464	0.000698	0.0142	8.136	1.177	0.0159	0.222
ENSMUSG00000020593	LPIN1	6.199	0.38	0.03	0.133	6.37	1.164	0.0000014	0.002
ENSMUSG00000042010	ACACB	8.084	0.765	0.00000107	0.00023	7.843	1.137	0.00000701	0.0049
ENSMUSG00000111013	Gm32468	3.08	1.395	0.00233	0.0292	2.939	1.133	0.0331	0.302
ENSMUSG00000020917	ACLY	9.259	0.447	0.00481	0.0449	9.24	1.084	0.0000384	0.0134
ENSMUSG00000027605	ACSS2	7.925	0.645	0.000655	0.0136	7.711	1.084	0.000239	0.0321
ENSMUSG00000094786	Gm14403	1.935	0.609	0.00541	0.0482	2.176	1.049	6.42E-07	0.0015
ENSMUSG00000066477	Gm16551	1.268	0.898	0.00721	0.0583	1.217	1.044	0.00649	0.15
ENSMUSG00000069324	Gm5096	6.763	1.127	0.0000123	0.00117	6.334	1.04	0.0107	0.186
ENSMUSG00000028976	SLC2A5	2.984	0.98	0.0000459	0.00263	2.881	1.029	0.000456	0.0413
ENSMUSG00000093651	Gm5873	2.827	0.406	0.0327	0.14	2.912	1.027	0.0000594	0.0169
ENSMUSG00000074768	BHMT	9.868	1.099	0.0000136	0.00125	9.603	0.996	0.0048	0.131
ENSMUSG00000055866	PER2	4.822	0.66	0.00827	0.0633	4.802	0.98	0.000388	0.0391
ENSMUSG00000030528	BLM	2.301	0.72	0.00668	0.0554	2.195	0.971	0.00146	0.0691
ENSMUSG00000038576	SUSD4	3.437	0.868	0.0016	0.0228	3.317	0.959	0.00313	0.105
ENSMUSG00000024424	TTC39C	7.493	0.73	0.000133	0.00479	7.36	0.959	0.00000917	0.0053
ENSMUSG00000024664	FADS3	5.765	0.632	0.00304	0.0344	5.711	0.956	0.000719	0.0503
ENSMUSG00000025153	FASN	11.06	0.494	0.00879	0.0658	11.168	0.946	0.000352	0.0376
ENSMUSG00000024978	GPAM	8.337	0.555	0.00114	0.0185	8.434	0.931	0.000652	0.0486
ENSMUSG00000015451	C4A/C4B	4.179	0.554	0.0234	0.116	4.258	0.927	0.00108	0.0597
ENSMUSG00000038751	PTK6	0.528	0.761	0.00765	0.0606	0.511	0.918	0.00591	0.142
ENSMUSG00000097673	Gm26608	1.79	0.607	0.00973	0.0696	1.848	0.911	0.00111	0.0604
ENSMUSG00000062181	CES3	7.323	0.838	0.0000582	0.00301	7.338	0.907	0.000206	0.0285
ENSMUSG00000096105	Bhmt-ps1	2.21	1.02	0.000193	0.00618	1.921	0.898	0.0142	0.212
ENSMUSG00000024421	LAMA3	1.021	1.014	0.000435	0.0105	0.978	0.892	0.00632	0.147
ENSMUSG00000003526	LOC102724788/PRODH	7.259	0.781	4.54E-08	0.0000252	7.105	0.858	0.0000794	0.0181
ENSMUSG00000029482	AACS	6.543	0.454	0.0396	0.157	6.796	0.847	0.000328	0.0374
ENSMUSG00000100094	1810008I18Rik	6.513	0.451	0.00288	0.0334	6.66	0.842	0.00000786	0.0051
ENSMUSG00000026471	MR1	3.38	0.725	0.00000573	0.000699	3.304	0.839	0.000741	0.0511
ENSMUSG00000054191	KLF1	0.884	0.682	0.000642	0.0134	0.989	0.838	0.00000952	0.0053
ENSMUSG00000003477	INMT	9.366	0.748	0.0000653	0.00318	9.313	0.836	0.00031	0.037
ENSMUSG00000032500	DCLK3	3.459	0.445	0.0167	0.0956	3.621	0.832	0.0000104	0.0053
ENSMUSG00000020623	MAP2K6	1.659	0.761	0.00499	0.046	1.624	0.832	0.00372	0.115
ENSMUSG00000031767	NUDT7	8.697	0.735	0.00000553	0.000685	8.501	0.831	0.0000186	0.0075
ENSMUSG00000110597	Gm8798	1.222	0.696	0.00192	0.0256	1.157	0.808	0.00323	0.107
ENSMUSG00000042834	NREP	3.523	0.711	0.00297	0.034	3.305	0.806	0.0117	0.195
ENSMUSG00000097908	4933404O12Rik	1.703	0.731	0.0000774	0.00345	1.531	0.8	0.00106	0.059
ENSMUSG00000078716	TMEM8B	1.184	1.203	1.75E-10	3.75E-07	0.925	0.797	0.00325	0.107
ENSMUSG00000032786	ALAS1	8.205	0.602	0.023	0.115	8.316	0.793	0.000982	0.0578
ENSMUSG00000010064	SLC38A3	10.584	0.506	0.0000189	0.00155	10.513	0.793	0.00000716	0.0049
ENSMUSG00000105096	GBP6	1.395	0.65	0.0317	0.138	2.241	0.788	0.033	0.302
ENSMUSG00000065952	C330021F23Rik/Gm5148	1.071	0.52	0.0305	0.135	1.036	0.784	0.0153	0.219
ENSMUSG00000005514	POR	7.98	0.539	0.00151	0.0219	8.212	0.784	0.0000448	0.0144
ENSMUSG00000022679	MPV17L	3.715	0.739	4.67E-07	0.00012	3.519	0.78	0.000197	0.0285
ENSMUSG00000112249	Gm30262	2.697	0.563	0.00289	0.0334	2.701	0.761	0.00133	0.065
ENSMUSG00000079494	Nat8f5	3.014	0.823	0.000368	0.00948	2.575	0.761	0.0255	0.267
ENSMUSG00000024665	FADS2	9.617	0.487	0.0017	0.0236	9.628	0.76	0.00034	0.0374
ENSMUSG00000021260	HHIPL1	1.059	0.656	0.0367	0.15	0.985	0.757	0.0452	0.343
ENSMUSG00000089943	UGT1A4	5.64	0.65	0.00426	0.0421	5.509	0.75	0.0174	0.231
ENSMUSG00000002769	GNMT	9.583	0.514	0.0000428	0.00254	9.576	0.749	0.00000115	0.0019
ENSMUSG00000028637	Ccdc30	1.475	0.859	0.00251	0.0305	1.333	0.747	0.0239	0.26
ENSMUSG00000080738	Mup-ps22	7.322	0.941	0.00516	0.047	7.369	0.737	0.017	0.228
ENSMUSG00000067071	HES6	5.545	0.456	0.000843	0.0156	5.622	0.733	0.0000221	0.0086
ENSMUSG00000021135	SLC10A1	9.16	0.703	0.00109	0.0181	9.156	0.733	0.00335	0.11
ENSMUSG00000090090	Mup-ps16	2.017	0.822	0.00159	0.0227	1.978	0.732	0.0346	0.308

Table S3 List of genes singificantly modulated in both Het KO (vs. WT) and with same pattern of regulation (WDSW experiment)

		Het vs WT				KO vs WT			
Ensembl	Symbol	Log2CPM	Log2FC	p-value	FDR	Log2CPM	Log2FC	p-value	FDR
ENSMUSG00000025271	PFKFB1	5.524	0.549	0.00000199	0.000343	5.495	0.732	0.00000431	0.0043
ENSMUSG00000066441	RDH11	5.856	0.427	0.0435	0.166	5.829	0.727	0.00813	0.163
ENSMUSG00000001665	Gstt3	5.473	0.732	6.21E-07	0.000154	5.223	0.712	0.00382	0.115
ENSMUSG00000079470	UTP14C	3.775	0.502	0.00983	0.0698	3.798	0.712	0.0015	0.0697
ENSMUSG00000116508	AL591952.3	2.404	0.812	6.57E-09	0.0000077	2.228	0.71	0.000292	0.0362
ENSMUSG00000114247	Gm32063	4.308	0.709	0.000065	0.00318	4.102	0.709	0.00237	0.0907
ENSMUSG00000029650	SLC46A3	4.492	0.659	1.63E-07	0.0000526	4.299	0.698	0.0000603	0.0169
ENSMUSG00000027762	SUCNR1	5.405	0.718	0.00000522	0.000659	5.301	0.695	0.00116	0.0615
ENSMUSG00000022389	TEF	7.098	0.44	0.0391	0.156	7.25	0.684	0.000712	0.0503
ENSMUSG00000058207	SERPINA3	12.037	0.679	0.000373	0.00956	12.025	0.678	0.000873	0.0548
ENSMUSG00000027765	P2RY1	3.15	0.602	0.00102	0.0176	2.955	0.672	0.0073	0.154
ENSMUSG00000070644	ETNK2	7.754	0.434	0.000802	0.0151	7.692	0.652	0.0000461	0.0145
ENSMUSG00000081084	Gm5638	1.077	0.596	0.00583	0.0508	0.982	0.651	0.0158	0.222
ENSMUSG00000024378	STARD4	6.799	0.394	0.000995	0.0172	6.79	0.651	0.0000287	0.0103
ENSMUSG00000031645	F11	5.598	0.555	0.0007	0.0142	5.556	0.65	0.00244	0.0923
ENSMUSG00000057880	ABAT	8.668	0.798	1.1E-08	0.00000889	8.514	0.644	0.00161	0.0724
ENSMUSG00000017718	AFMID	5.177	0.656	0.00000214	0.000359	5.042	0.644	0.000889	0.0548
ENSMUSG00000024899	PAPSS2	7.649	0.497	5.72E-07	0.000144	7.63	0.641	0.00000404	0.0043
ENSMUSG00000028600	PODN	2.153	0.626	0.000452	0.0108	1.864	0.64	0.0037	0.115
ENSMUSG00000030088	ALDH1L1	9.938	0.488	1.41E-07	0.0000479	9.889	0.638	0.00000867	0.0053
ENSMUSG00000046541	ZNF526	2.147	0.425	0.00146	0.0214	2.107	0.63	0.00177	0.0753
ENSMUSG00000020538	SREBF1	9.483	0.495	0.00505	0.0463	9.346	0.618	0.0034	0.11
ENSMUSG00000048087	Gm4737	6.462	0.563	8.93E-07	0.000198	6.333	0.617	0.000162	0.027
ENSMUSG00000029445	HPD	10.765	0.616	0.0000882	0.00374	10.678	0.612	0.003	0.102
ENSMUSG00000026495	EFCAB2	2.097	0.541	0.000022	0.00167	1.991	0.611	0.00177	0.0753
ENSMUSG00000026259	NGEF	4.8	0.476	0.00101	0.0174	4.794	0.609	0.000557	0.0454
ENSMUSG00000051041	OLFML1	4.671	0.513	0.0000252	0.00185	4.598	0.589	0.000143	0.0256
ENSMUSG00000023826	PRKN	1.154	0.612	0.000595	0.0128	1.188	0.588	0.00363	0.114
ENSMUSG00000041782	LAD1	3.176	0.732	0.00347	0.0371	3.045	0.584	0.0262	0.271
ENSMUSG00000048538	Gm9826	4.731	0.607	9.43E-09	0.00000813	4.612	0.575	0.00049	0.0435
ENSMUSG00000050445	CYP8B1	8.535	0.626	0.00118	0.0189	8.322	0.573	0.0498	0.357
ENSMUSG00000027597	AHCY	8.388	0.531	0.00000143	0.00028	8.335	0.567	0.000359	0.0378
ENSMUSG00000087404	Gm11752	1.302	0.636	0.00954	0.0688	1.147	0.567	0.0412	0.329
ENSMUSG00000038145	SNRK	5.607	0.458	0.000272	0.00786	5.605	0.565	0.000821	0.0528
ENSMUSG00000044197	GPR146	6.666	0.406	0.00000441	0.000598	6.64	0.564	0.000569	0.046
ENSMUSG00000025317	CA5A	5.768	0.604	8.01E-08	0.0000323	5.689	0.558	0.00116	0.0616
ENSMUSG00000040820	HLCS	4.344	0.508	0.000271	0.00786	4.314	0.555	0.00104	0.0587
ENSMUSG00000113063	Gm34667	2.704	0.729	0.00315	0.035	2.714	0.549	0.0163	0.225
ENSMUSG00000032310	CYP1A2	7.889	0.634	0.00106	0.0178	7.695	0.548	0.0405	0.327
ENSMUSG00000074283	Zfp109	1.05	0.508	0.00552	0.0489	0.959	0.548	0.0107	0.186
ENSMUSG00000021340	GPLD1	7.454	0.524	1.04E-07	0.0000377	7.345	0.544	0.0000687	0.0174
ENSMUSG00000052656	RNF103	6.775	0.389	0.0000603	0.00307	6.814	0.544	0.0000139	0.0062
ENSMUSG00000018727	CPSF4L	2.181	0.69	0.000858	0.0158	2.114	0.542	0.0301	0.289
ENSMUSG00000045094	ARHGEF37	2.799	0.532	0.000471	0.0111	2.65	0.538	0.022	0.253
ENSMUSG00000074052	BC048644	3.336	0.512	0.00222	0.0281	3.293	0.536	0.0123	0.199
ENSMUSG00000038072	GALNT11	4.241	0.488	0.00142	0.0211	4.224	0.529	0.00595	0.142
ENSMUSG00000026723	TRDMT1	1.276	0.499	0.0138	0.0857	1.185	0.521	0.0284	0.281
ENSMUSG00000074071	Fam169b	4.706	0.398	0.000099	0.004	4.77	0.52	6.79E-07	0.0015
ENSMUSG00000042102	DMGDH	8.801	0.507	6.01E-08	0.0000297	8.698	0.512	0.00309	0.104
ENSMUSG00000024292	CYP4F12	7.51	0.468	0.00954	0.0688	7.515	0.504	0.0219	0.252
ENSMUSG00000086010	LOC102631757	2.696	0.438	0.00275	0.0325	2.827	0.501	0.00122	0.0634
ENSMUSG00000035372	C11orf24	6.03	0.493	0.000481	0.0113	5.944	0.5	0.0246	0.264
ENSMUSG00000062410	HSD3B1	6.685	0.544	0.0000189	0.00155	6.568	0.499	0.00102	0.0581
ENSMUSG00000024354	SLC23A1	6.378	0.465	0.0000308	0.00209	6.303	0.499	0.00779	0.158
ENSMUSG00000097203	4732419C18Rik	3.373	0.424	0.00173	0.024	3.302	0.497	0.00173	0.0748
ENSMUSG00000028402	MPDZ	4.808	0.573	1.87E-08	0.0000127	4.69	0.494	0.000182	0.0285
ENSMUSG00000057228	AADAT	6.262	0.501	3.38E-07	0.0000968	6.155	0.491	0.0000521	0.0156
ENSMUSG00000002908	KCNN1	1.629	0.541	0.00162	0.0229	1.545	0.488	0.0188	0.237
ENSMUSG00000038967	PDK2	6.862	0.495	7.77E-09	0.00000813	6.716	0.488	0.000198	0.0285
ENSMUSG00000003123	LIPE	4.173	0.523	0.000293	0.00825	4.064	0.482	0.024	0.26
ENSMUSG00000095362	ZNF442	0.505	0.519	0.00494	0.0457	2.27	0.479	0.0338	0.306
ENSMUSG00000029499	PXMP2	6.281	0.428	0.00000119	0.000244	6.167	0.476	0.000189	0.0285
ENSMUSG00000029269	SULT1B1	4.637	0.623	0.00083	0.0154	4.317	0.474	0.0455	0.343
ENSMUSG00000024140	EPAS1	7.816	0.4	0.00000416	0.000598	7.713	0.472	0.000995	0.0578
ENSMUSG00000038217	TLCD2	6.173	0.556	0.0000102	0.00103	6.161	0.471	0.00347	0.111
ENSMUSG00000020268	LYRM7	0.94	0.552	0.000584	0.0127	0.663	0.47	0.00892	0.17
ENSMUSG00000025089	GFRA1	7.235	0.493	9.47E-09	0.00000813	7.048	0.468	0.000456	0.0413
ENSMUSG00000086628	Gm16157	3.035	0.48	0.00146	0.0214	3.004	0.467	0.00498	0.133
ENSMUSG00000068742	CRY2	5.337	0.394	0.000802	0.0151	5.292	0.465	0.000853	0.0539
ENSMUSG00000025260	HSD17B10	7.185	0.563	0.000013	0.00121	7.074	0.465	0.0241	0.261
ENSMUSG00000071177	SERPINA1	9.337	0.401	0.0019	0.0255	9.26	0.458	0.00481	0.131
ENSMUSG00000087165	2010001A14Rik	1.242	0.535	0.000828	0.0154	1.237	0.454	0.00602	0.142
ENSMUSG00000068686	CD59	1.696	0.608	0.00014	0.00491	1.554	0.453	0.0274	0.277
ENSMUSG00000030055	RAB43	2.133	0.468	0.0124	0.0809	2.194	0.453	0.0431	0.335
ENSMUSG00000030935	ACSM3	6.507	0.605	4.69E-08	0.0000252	6.345	0.452	0.00372	0.115

Table S3 List of genes singificantly modulated in both Het KO (vs. WT) and with same pattern of regulation (WDSW experiment)

		Het vs WT				KO vs WT			
**Ensembl**	**Symbol**	**Log2CPM**	**Log2FC**	**p-value**	**FDR**	**Log2CPM**	**Log2FC**	**p-value**	**FDR**
ENSMUSG00000032788	PDXK	4.733	0.568	7.03E-08	0.0000312	4.589	0.452	0.00617	0.145
ENSMUSG00000039632	CCDC151	1.507	0.727	0.000397	0.00995	1.366	0.45	0.0378	0.318
ENSMUSG00000116718	AC154378.1	2.747	0.613	0.00000467	0.00062	2.571	0.447	0.0195	0.241
ENSMUSG00000031549	IDO2	6.484	0.567	5.44E-08	0.000028	6.261	0.447	0.0011	0.0604
ENSMUSG00000025815	DHTKD1	6.269	0.456	0.0000923	0.00386	6.226	0.443	0.00394	0.117
ENSMUSG00000038641	AKR1D1	7.392	0.431	0.0026	0.0312	7.297	0.439	0.0203	0.245
ENSMUSG00000068463	C730014E05Rik	5.592	0.5	0.00000902	0.000936	5.557	0.439	0.000208	0.0286
ENSMUSG00000035864	SYT1	3.232	0.581	0.0000859	0.0037	3.052	0.436	0.0266	0.272
ENSMUSG00000084129	Hmgb1-ps1	1.125	0.441	0.00741	0.0593	1.013	0.434	0.02	0.244
ENSMUSG00000070305	MPZL3	3.266	0.385	0.000354	0.00937	3.309	0.432	0.000879	0.0548
ENSMUSG00000021589	RHOBTB3	2.595	0.423	0.00578	0.0506	2.585	0.432	0.0159	0.222
ENSMUSG00000029695	AASS	8.286	0.44	0.000218	0.0068	8.154	0.429	0.00379	0.115
ENSMUSG00000025577	CBX2	3.344	0.423	0.00508	0.0465	3.194	0.427	0.042	0.331
ENSMUSG00000020334	SLC22A4	1.242	0.419	0.00141	0.0211	1.251	0.427	0.0209	0.247
ENSMUSG00000035944	TTC38	6.662	0.434	8.13E-07	0.000187	6.608	0.427	0.00104	0.0587
ENSMUSG00000000340	DBT	6.552	0.422	9.34E-07	0.000204	6.356	0.424	0.000825	0.0528
ENSMUSG00000036585	FGF1	6.476	0.557	0.000113	0.00435	6.343	0.424	0.0359	0.312
ENSMUSG00000030737	SLCO2B1	7.588	0.443	4.01E-12	1.54E-08	7.502	0.424	0.000532	0.0447
ENSMUSG00000037458	AZIN1	6.866	0.443	0.0000661	0.00318	6.729	0.422	0.00146	0.0691
ENSMUSG00000037798	MAT1A	11.508	0.394	0.00000353	0.000523	11.453	0.417	0.000315	0.0372
ENSMUSG00000078566	BNIP3	6.136	0.383	0.000973	0.017	6.099	0.415	0.00189	0.0789
ENSMUSG00000040447	SPNS2	5.378	0.481	7.63E-08	0.0000317	5.239	0.415	0.00281	0.0981
ENSMUSG00000022843	CLCN2	5.705	0.506	0.0000631	0.00315	5.659	0.413	0.0169	0.228
ENSMUSG00000030109	SLC6A12	6.14	0.426	4.22E-09	0.00000604	6.055	0.413	0.000125	0.0234
ENSMUSG00000039763	DNAJC28	3.608	0.564	5.32E-10	9.79E-07	3.575	0.411	0.00228	0.0896
ENSMUSG00000116802	LOC102640673	0.776	0.56	0.0000239	0.00178	0.696	0.41	0.00239	0.0912
ENSMUSG00000039853	TRIM14	3.521	0.5	0.00104	0.0178	3.498	0.404	0.0236	0.26
ENSMUSG00000009614	SARDH	9.654	0.478	0.00000023	0.0000704	9.603	0.401	0.00184	0.0776
ENSMUSG00000044250	PCED1B	2.199	0.443	0.000601	0.0129	2.154	0.4	0.00548	0.14
ENSMUSG00000021097	CLMN	7.544	0.395	0.000572	0.0125	7.409	0.398	0.00694	0.153
ENSMUSG00000036904	FZD8	3.305	0.59	0.000822	0.0154	3.252	0.397	0.0417	0.33
ENSMUSG00000028536	C1orf210	3.342	0.408	0.00226	0.0286	3.356	0.395	0.0201	0.244
ENSMUSG00000026669	MCM10	5.817	0.382	0.00934	0.068	5.76	0.394	0.0151	0.216
ENSMUSG00000024135	SRBD1	4.426	0.48	8.73E-08	0.0000331	4.323	0.391	0.00128	0.0641
ENSMUSG00000036687	TMEM184A	4.309	0.476	0.000131	0.00477	4.244	0.389	0.00374	0.115
ENSMUSG00000092545	Gm20319	5.585	0.431	0.00000584	0.000703	5.512	0.385	0.00105	0.0587
ENSMUSG00000020534	SHMT1	7.916	0.38	0.0000107	0.00105	7.811	0.382	0.00993	0.179
ENSMUSG00000019883	ECHDC1	4.706	0.4	1.45E-08	0.000011	4.624	0.38	0.00339	0.11
ENSMUSG00000037808	FAM76B	3.407	-0.412	0.000286	0.0081	3.546	-0.379	0.00692	0.153
ENSMUSG00000021806	NID2	3.221	-0.534	0.00111	0.0183	3.482	-0.379	0.0143	0.212
ENSMUSG00000031875	CMTM3	3.454	-0.486	0.000956	0.0169	3.57	-0.382	0.0458	0.344
ENSMUSG00000032766	GNG11	2.755	-0.503	0.0000219	0.00167	2.981	-0.383	0.0111	0.19
ENSMUSG00000043415	OTUD1	2.686	-0.419	0.0022	0.028	2.827	-0.384	0.0407	0.327
ENSMUSG00000024542	CEP192	2.339	-0.512	0.000345	0.00917	2.539	-0.385	0.0346	0.308
ENSMUSG00000027610	GSS	6.252	-0.513	0.00000753	0.000848	6.515	-0.386	0.0147	0.214
ENSMUSG00000028044	CKS1B	2.333	-0.474	0.00259	0.0312	2.366	-0.399	0.00921	0.171
ENSMUSG00000060438	Rps10-ps1	2.484	-0.388	0.0000851	0.00369	2.644	-0.401	0.00235	0.0903
ENSMUSG00000009207	LNPK	1.739	-0.425	0.00851	0.0644	1.765	-0.404	0.0137	0.209
ENSMUSG00000042712	TCEAL9	3.773	-0.527	0.00027	0.00786	3.853	-0.416	0.0158	0.222
ENSMUSG00000100147	1700047M11Rik	1.004	-0.471	0.0092	0.0674	0.999	-0.418	0.0445	0.34
ENSMUSG00000025372	BAIAP2	4.688	-0.502	0.00664	0.0552	4.732	-0.42	0.047	0.347
ENSMUSG00000056602	FRY	2.028	-0.456	0.00982	0.0698	2.08	-0.42	0.05	0.358
ENSMUSG00000040943	TET2	3.851	-0.567	0.000178	0.00581	3.976	-0.421	0.0236	0.26
ENSMUSG00000034342	CBL	4.035	-0.52	0.00286	0.0333	4.157	-0.427	0.0492	0.356
ENSMUSG00000029174	TBC1D1	3.601	-0.477	0.000329	0.00887	3.645	-0.427	0.0196	0.241
ENSMUSG00000040722	SCAMP5	4.675	-0.382	0.0178	0.0989	4.675	-0.431	0.03	0.288
ENSMUSG00000000552	ZNF385A	4.102	-0.558	0.00017	0.0056	4.256	-0.431	0.0169	0.228
ENSMUSG00000030729	PGM2L1	1.809	-0.451	0.0134	0.0845	1.827	-0.433	0.00323	0.107
ENSMUSG00000036667	TCAF1	2.086	-0.695	0.000414	0.0102	2.385	-0.433	0.035	0.308
ENSMUSG00000025268	MAGED2	3.424	-0.391	0.0211	0.109	3.431	-0.435	0.0191	0.24
ENSMUSG00000030159	CLEC1B	3.662	-0.477	0.00405	0.0407	3.692	-0.436	0.0291	0.285
ENSMUSG00000020231	DIP2A	2.512	-0.463	0.000193	0.00618	2.561	-0.436	0.0015	0.0697
ENSMUSG00000020737	JPT1	3.635	-0.626	0.0000167	0.00145	3.836	-0.437	0.0383	0.32
ENSMUSG00000024143	RHOQ	3.825	-0.412	0.000648	0.0135	3.918	-0.437	0.00229	0.0896
ENSMUSG00000030814	BCL7C	3.381	-0.439	0.000731	0.0145	3.542	-0.439	0.0181	0.234
ENSMUSG00000011305	PLIN5	5.392	-0.383	0.00679	0.056	5.651	-0.439	0.0142	0.212
ENSMUSG00000033705	STARD9	2.531	-0.546	0.00106	0.0178	2.674	-0.441	0.0434	0.336
ENSMUSG00000001473	TUBB6	3.592	-0.692	0.000014	0.00127	3.931	-0.441	0.0451	0.343
ENSMUSG00000042787	EXOG	1.162	-0.4	0.0103	0.0716	1.197	-0.444	0.00777	0.158
ENSMUSG00000019970	SGK1	4.36	-0.467	0.000129	0.00472	4.369	-0.445	0.0026	0.0942
ENSMUSG00000038776	EPHX1	9.148	-0.411	0.00772	0.061	9.269	-0.448	0.0235	0.26
ENSMUSG00000033589	REEP4	3.104	-0.423	0.0000982	0.00399	3.242	-0.449	0.00157	0.0713
ENSMUSG00000053175	BCL3	4.425	-0.562	0.000396	0.00995	4.528	-0.45	0.0354	0.31
ENSMUSG00000034349	SMC4	3.887	-0.46	0.00081	0.0152	4.025	-0.451	0.00381	0.115
ENSMUSG00000000555	ITGA5	5.097	-0.425	0.00191	0.0256	5.13	-0.453	0.00265	0.0951

		Het vs WT				KO vs WT			
**Ensembl**	**Symbol**	**Log2CPM**	**Log2FC**	**p-value**	**FDR**	**Log2CPM**	**Log2FC**	**p-value**	**FDR**
ENSMUSG00000031788	KIFC3	4.056	-0.572	0.000907	0.0164	4.203	-0.453	0.0436	0.337
ENSMUSG00000003873	BAX	4.206	-0.588	1.3E-12	8.37E-09	4.33	-0.456	0.00000674	0.0049
ENSMUSG00000021948	PRKCD	4.753	-0.603	0.000893	0.0162	4.852	-0.456	0.035	0.308
ENSMUSG00000053819	CAMK2D	3.66	-0.404	0.00166	0.0233	3.76	-0.459	0.0017	0.0739
ENSMUSG00000086429	Gt(ROSA)26Sor	2.809	-0.421	0.00264	0.0315	2.987	-0.459	0.00822	0.164
ENSMUSG00000027435	CD93	3.296	-0.402	0.0397	0.158	3.274	-0.464	0.0465	0.347
ENSMUSG00000058135	GSTM5	10.702	-0.398	0.035	0.146	10.851	-0.464	0.0481	0.352
ENSMUSG00000020812	Snhg16	0.958	-0.589	0.000115	0.00442	1.175	-0.464	0.011	0.189
ENSMUSG00000020886	DLG4	0.918	-0.592	0.00127	0.0197	1.015	-0.466	0.0369	0.315
ENSMUSG00000026547	TAGLN2	4.861	-0.733	0.000258	0.00769	5.046	-0.468	0.0353	0.31
ENSMUSG00000027801	TM4SF4	6.577	-0.545	0.00325	0.0356	6.643	-0.472	0.0239	0.26
ENSMUSG00000032902	SLC16A1	6.213	-0.45	0.00167	0.0233	6.291	-0.473	0.0125	0.2
ENSMUSG00000099843	Gm7160	1.031	-0.468	0.00378	0.0389	1.082	-0.478	0.0294	0.286
ENSMUSG00000018920	CXCL16	3.324	-0.562	0.00577	0.0505	3.409	-0.479	0.049	0.355
ENSMUSG00000026640	PLXNA2	4.595	-0.547	0.0000747	0.00341	4.809	-0.484	0.00689	0.153
ENSMUSG00000034329	BRIP1	1.207	-0.384	0.00605	0.052	1.507	-0.485	0.0157	0.222
ENSMUSG00000043391	C3orf70	3.592	-0.414	0.00085	0.0156	3.602	-0.487	0.000191	0.0285
ENSMUSG00000043068	FAM89A	2.456	-0.399	0.0342	0.144	2.424	-0.488	0.0497	0.357
ENSMUSG00000026103	GLS	2.999	-0.516	0.00842	0.064	3.21	-0.489	0.0489	0.354
ENSMUSG00000060771	TSGA10	0.765	-0.382	0.0186	0.102	0.766	-0.489	0.0185	0.236
ENSMUSG00000020773	TRIM47	1.944	-0.843	0.0000984	0.00399	2.171	-0.492	0.0498	0.357
ENSMUSG00000044906	C18orf54	1.96	-0.467	0.00404	0.0407	2.125	-0.497	0.0247	0.264
ENSMUSG00000025395	PRIM1	1.55	-0.556	0.00132	0.0202	1.592	-0.497	0.0377	0.318
ENSMUSG00000027459	FAM110A	1.696	-0.382	0.0185	0.101	1.614	-0.498	0.017	0.228
ENSMUSG00000036501	FAM13B	4.026	-0.41	0.00197	0.026	4.149	-0.502	0.00235	0.0903
ENSMUSG00000050410	TCF19	1.633	-0.551	0.000153	0.0052	1.986	-0.502	0.0311	0.294
ENSMUSG00000041238	RBBP8	3.115	-0.451	0.00097	0.017	3.123	-0.503	0.00125	0.0637
ENSMUSG00000015745	PLEKHO1	2.966	-0.559	0.000727	0.0145	3.062	-0.504	0.018	0.233
ENSMUSG00000002233	RHOC	3.738	-0.932	7.87E-07	0.000184	4.259	-0.504	0.047	0.347
ENSMUSG00000043681	Fam25c	4.36	-0.654	0.0000329	0.00214	4.442	-0.507	0.00345	0.111
ENSMUSG00000046731	KCTD11	1.709	-0.39	0.00488	0.0452	1.781	-0.507	0.00227	0.0896
ENSMUSG00000022948	SETD4	2.601	-0.731	0.000127	0.00472	2.627	-0.508	0.0225	0.253
ENSMUSG00000029177	CENPA	2.461	-0.796	3.81E-08	0.0000239	2.821	-0.509	0.0102	0.182
ENSMUSG00000004098	COL5A3	6.592	-0.438	0.012	0.0787	6.625	-0.527	0.0125	0.2
ENSMUSG00000029484	ANXA3	2.799	-0.799	0.000125	0.00467	2.993	-0.532	0.0393	0.324
ENSMUSG00000057894	ZNF329	2.48	-0.464	0.0108	0.074	2.415	-0.533	0.000992	0.0578
ENSMUSG00000027087	ITGAV	4.821	-0.509	0.000169	0.0056	4.913	-0.536	0.000405	0.0401
ENSMUSG00000046111	CEP295	2.681	-0.42	0.000883	0.0161	2.68	-0.541	0.000648	0.0486
ENSMUSG00000022360	ATAD2	2.368	-0.721	0.000404	0.01	2.549	-0.545	0.0266	0.272
ENSMUSG00000030342	CD9	4.565	-0.664	0.000112	0.00435	4.754	-0.555	0.00657	0.151
ENSMUSG00000036381	P2RY14	1.076	-0.639	0.00951	0.0687	1.205	-0.56	0.0469	0.347
ENSMUSG00000023913	PLA2G7	2.558	-0.616	0.0247	0.119	2.543	-0.562	0.0499	0.357
ENSMUSG00000021701	PLK2	3.961	-0.448	0.0224	0.114	3.852	-0.567	0.0199	0.243
ENSMUSG00000022822	ABCC5	3.118	-0.631	0.000325	0.00883	3.481	-0.582	0.0123	0.2
ENSMUSG00000032348	Gsta4	5.694	-0.62	0.000404	0.01	5.829	-0.586	0.00876	0.169
ENSMUSG00000004951	HSPB1	3.631	-0.459	0.033	0.141	3.577	-0.591	0.0256	0.268
ENSMUSG00000025880	SMAD7	3.584	-0.521	0.00899	0.0665	3.75	-0.594	0.00165	0.0734
ENSMUSG00000030659	NUCB2	1.523	-0.922	0.000032	0.00213	1.63	-0.595	0.0223	0.253
ENSMUSG00000036672	CENPT	1.755	-0.388	0.0275	0.126	1.585	-0.602	0.0103	0.183
ENSMUSG00000019979	APAF1	2.047	-0.567	0.00872	0.0656	2.243	-0.603	0.0237	0.26
ENSMUSG00000049303	SYT12	1.668	-0.812	0.000594	0.0128	1.872	-0.608	0.0238	0.26
ENSMUSG00000037405	ICAM1	4.14	-0.785	0.0000931	0.00387	4.403	-0.61	0.0142	0.212
ENSMUSG00000028312	SMC2	1.844	-0.583	0.00534	0.0479	2.084	-0.61	0.00856	0.167
ENSMUSG00000038147	CD84	2.7	-0.635	0.00354	0.0376	2.798	-0.616	0.0213	0.249
ENSMUSG00000089774	SLC5A3	2.323	-0.593	0.0000993	0.004	2.411	-0.62	0.00563	0.141
ENSMUSG00000007872	ID3	5.531	-0.889	0.0000308	0.00209	5.763	-0.623	0.00429	0.123
ENSMUSG00000030149	Klrk1	0.717	-0.44	0.044	0.167	0.867	-0.625	0.0197	0.242
ENSMUSG00000019194	SCN1B	1.713	-0.805	0.0000714	0.00334	2.1	-0.625	0.034	0.307
ENSMUSG00000029910	MAD2L1	2.106	-0.573	0.0000496	0.00272	2.267	-0.631	0.00189	0.079
ENSMUSG00000005397	NID1	4.904	-0.766	0.0035	0.0373	5.193	-0.636	0.0399	0.325
ENSMUSG00000032997	CHPF	1.378	-0.766	0.000876	0.016	1.659	-0.637	0.0248	0.264
ENSMUSG00000027201	MYEF2	2.693	-0.448	0.000559	0.0123	2.718	-0.638	0.0000802	0.0181
ENSMUSG00000026566	MPZL1	2.128	-0.821	0.0000657	0.00318	2.347	-0.64	0.0151	0.216
ENSMUSG00000066877	NCK2	1.77	-0.724	0.000458	0.0108	1.831	-0.64	0.00982	0.178
ENSMUSG00000025154	ARHGAP19	2.645	-0.661	0.0031	0.0347	2.86	-0.645	0.0132	0.206
ENSMUSG00000053693	MAST1	0.584	-0.997	0.00000853	0.000916	0.935	-0.647	0.00716	0.154
ENSMUSG00000027660	SKIL	3.689	-0.72	0.000914	0.0164	3.926	-0.647	0.0175	0.231
ENSMUSG00000037621	ATOH8	4.059	-0.492	0.0262	0.123	3.972	-0.649	0.00161	0.0724
ENSMUSG00000048997	ATXN7L2	1.315	-0.652	0.00000796	0.000861	1.405	-0.649	0.000205	0.0285
ENSMUSG00000039585	MYO9A	3.241	-0.772	0.00104	0.0178	3.33	-0.652	0.041	0.327
ENSMUSG00000048490	NRIP1	4.041	-0.387	0.0012	0.0191	3.995	-0.655	0.00000497	0.0046
ENSMUSG00000051439	CD14	2.328	-0.802	0.0158	0.0925	2.441	-0.672	0.0369	0.315
ENSMUSG00000054942	MIGA1	0.509	-0.586	0.0329	0.141	0.505	-0.675	0.0395	0.324
ENSMUSG00000032413	RASA2	1.825	-0.517	0.00137	0.0207	1.648	-0.675	0.00000675	0.0049
ENSMUSG00000027111	ITGA6	1.277	-0.689	0.00798	0.0622	1.469	-0.677	0.0375	0.318

Table S3 List of genes singificantly modulated in both Het KO (vs. WT) and with same pattern of regulation (WDSW experiment)

		Het vs WT				KO vs WT			
**Ensembl**	**Symbol**	**Log2CPM**	**Log2FC**	**p-value**	**FDR**	**Log2CPM**	**Log2FC**	**p-value**	**FDR**
ENSMUSG00000028019	PDGFC	2.305	-0.634	0.00002	0.00159	2.354	-0.688	0.000575	0.046
ENSMUSG00000063146	CLIP2	2.539	-0.683	0.000527	0.0119	2.793	-0.692	0.0114	0.192
ENSMUSG00000005125	NDRG1	3.415	-0.53	0.0047	0.0444	3.52	-0.692	0.000452	0.0413
ENSMUSG00000051146	CAMK2N2	0.539	-0.444	0.018	0.0999	0.629	-0.694	0.00502	0.133
ENSMUSG00000032245	CLN6	2.612	-0.932	0.00000419	0.000598	3.263	-0.694	0.00783	0.159
ENSMUSG00000051790	NLGN2	2.005	-0.707	0.0000196	0.00157	2.202	-0.695	0.00282	0.0981
ENSMUSG00000003228	GRK5	1.975	-0.587	0.0000372	0.00234	2.043	-0.696	0.0000532	0.0156
ENSMUSG00000044968	NAPEPLD	0.892	-0.416	0.043	0.165	0.844	-0.697	0.00677	0.152
ENSMUSG00000022528	HES1	4.041	-0.526	0.0165	0.0949	3.968	-0.698	0.00065	0.0486
ENSMUSG00000045287	RTN4RL1	3.648	-0.549	0.000829	0.0154	3.915	-0.7	0.00365	0.114
ENSMUSG00000026622	NEK2	2.607	-0.622	0.0000998	0.00401	2.662	-0.704	0.000307	0.037
ENSMUSG00000023905	TNFRSF12A	3.01	-1.111	0.0000219	0.00167	3.267	-0.707	0.0345	0.308
ENSMUSG00000026074	MAP4K4	4.392	-0.839	3.83E-07	0.000103	4.613	-0.716	0.000809	0.0526
ENSMUSG00000029314	GPAT3	3.195	-0.623	0.0261	0.123	3.223	-0.723	0.0303	0.29
ENSMUSG00000041219	ARHGAP11A	1.938	-1.079	0.00045	0.0107	2.267	-0.737	0.0297	0.287
ENSMUSG00000025396	HSD17B6	6.938	-0.762	0.000261	0.00775	7.385	-0.744	0.0137	0.209
ENSMUSG00000024736	TMEM132A	1.057	-0.922	0.00000129	0.000256	1.24	-0.747	0.00651	0.15
ENSMUSG00000048612	MYOF	2.364	-0.926	0.00372	0.0386	2.839	-0.751	0.0366	0.314
ENSMUSG00000026956	UAP1L1	3.605	-0.927	0.00633	0.0534	3.861	-0.751	0.0452	0.343
ENSMUSG00000042029	NCAPG2	0.803	-0.765	0.0108	0.074	1.017	-0.761	0.037	0.316
ENSMUSG00000040663	CLCF1	1.5	-1.355	0.00000202	0.000343	2.565	-0.77	0.0384	0.32
ENSMUSG00000034731	DGKH	1.286	-0.76	0.00166	0.0233	1.416	-0.778	0.0128	0.203
ENSMUSG00000021556	GOLM1	1.091	-1.093	0.0000601	0.00307	1.413	-0.784	0.0122	0.199
ENSMUSG00000002265	PEG3	3.005	-0.712	0.00541	0.0482	3.107	-0.792	0.0245	0.263
ENSMUSG00000030322	MBD4	0.813	-0.548	0.0272	0.126	0.674	-0.795	0.00258	0.0942
ENSMUSG00000026785	PKN3	1.069	-0.519	0.00455	0.0435	1.104	-0.797	0.000289	0.0362
ENSMUSG00000060923	ACYP2	1.57	-0.597	0.00761	0.0605	1.453	-0.799	0.000339	0.0374
ENSMUSG00000030020	PRICKLE2	0.724	-0.483	0.0271	0.126	0.77	-0.8	0.00206	0.0836
ENSMUSG00000029009	MTHFR	3.982	-0.489	0.0287	0.13	3.98	-0.802	0.000674	0.0491
ENSMUSG00000028832	STMN1	1.471	-1.03	0.0000103	0.00103	2.046	-0.803	0.0069	0.153
ENSMUSG00000002297	DBF4	2.098	-0.847	0.00000152	0.000284	2.27	-0.811	0.00000598	0.0049
ENSMUSG00000042745	ID1	2.219	-1.12	0.00267	0.0318	2.901	-0.816	0.0201	0.244
ENSMUSG00000025758	PLK4	0.82	-0.715	0.00187	0.0252	0.814	-0.816	0.000515	0.0442
ENSMUSG00000056737	CAPG	2.55	-0.937	0.00195	0.0258	2.545	-0.82	0.0254	0.267
ENSMUSG00000037157	IL22RA1	2.264	-0.557	0.0247	0.119	2.386	-0.827	0.00436	0.124
ENSMUSG00000090942	F830016B08Rik	1.116	-0.977	0.000809	0.0152	1.507	-0.832	0.0237	0.26
ENSMUSG00000105315	Gm18635	1.745	-0.528	0.0201	0.107	1.753	-0.871	0.000121	0.023
ENSMUSG00000043263	IFI16	1.772	-1.01	0.0211	0.11	1.589	-0.886	0.00977	0.177
ENSMUSG00000003534	DDR1	1.335	-0.927	0.00481	0.0449	1.285	-0.887	0.0288	0.283
ENSMUSG00000033792	ATP7A	1.064	-0.678	0.0182	0.101	0.986	-0.913	0.00385	0.115
ENSMUSG00000035385	Ccl2	1.265	-0.807	0.0185	0.102	1.363	-0.925	0.0225	0.253
ENSMUSG00000085939	Cd63-ps	0.812	-0.812	0.0135	0.085	0.851	-0.93	0.0152	0.218
ENSMUSG00000038775	VILL	0.789	-0.917	0.00078	0.0149	0.952	-0.94	0.00779	0.158
ENSMUSG00000023505	CDCA3	0.959	-1.424	0.00000433	0.000598	1.521	-0.941	0.00893	0.17
ENSMUSG00000033031	CIP2A	0.617	-0.971	0.000494	0.0114	0.758	-0.945	0.000551	0.0452
ENSMUSG00000019942	CDK1	0.966	-1.777	0.00000152	0.000284	1.578	-0.954	0.0185	0.236
ENSMUSG00000015312	GADD45B	0.707	-1.457	0.0000249	0.00184	1.616	-0.958	0.0278	0.278
ENSMUSG00000082676	Gm11843	0.774	-0.873	0.00255	0.0308	0.759	-0.974	0.0169	0.228
ENSMUSG00000027624	EPB41L1	2.496	-1.608	4.48E-08	0.0000252	3.093	-0.981	0.0124	0.2
ENSMUSG00000037379	SPON2	4.418	-0.9	0.000738	0.0145	4.52	-0.988	0.00515	0.135
ENSMUSG00000025351	CD63	2.728	-1.259	0.0000399	0.00245	2.916	-1.008	0.00454	0.128
ENSMUSG00000045934	MTMR11	1.688	-1.331	0.00104	0.0178	1.865	-1.038	0.0174	0.231
ENSMUSG00000027699	ECT2	0.905	-1.11	0.00073	0.0145	0.817	-1.041	0.00319	0.106
ENSMUSG00000046818	DDIT4L	0.599	-0.971	0.01	0.0705	0.661	-1.065	0.0158	0.222
ENSMUSG00000026628	ATF3	1.955	-1.513	0.000553	0.0122	2.056	-1.094	0.0252	0.267
ENSMUSG00000027068	DHRS9	2.421	-0.814	0.0419	0.163	2.279	-1.111	0.00729	0.154
ENSMUSG00000067656	SLC22A25	2.223	-0.938	0.0316	0.138	2.142	-1.169	0.00674	0.152
ENSMUSG00000044258	Ctla2a/Ctla2b	0.538	-0.982	0.000153	0.00521	0.585	-1.198	0.000403	0.0401
ENSMUSG00000062991	Nrg1	0.647	-1.693	0.00117	0.0188	1.113	-1.261	0.0338	0.306
ENSMUSG00000012443	KIF11	0.599	-1.369	0.000829	0.0154	0.809	-1.27	0.00703	0.153
ENSMUSG00000059060	RAD51B	3.876	-1.436	0.0000732	0.00338	3.957	-1.275	0.0038	0.115
ENSMUSG00000004038	Gstm3	6.655	-1.291	0.0159	0.0925	6.657	-1.291	0.0206	0.246
ENSMUSG00000020914	TOP2A	1.51	-1.596	0.00197	0.026	1.599	-1.325	0.0187	0.237
ENSMUSG00000031004	MKI67	2.091	-1.909	0.000149	0.00512	2.587	-1.339	0.0167	0.228
ENSMUSG00000023067	CDKN1A	3.012	-1.447	0.0000769	0.00344	3.523	-1.361	0.00314	0.105
ENSMUSG00000024298	Zfp871	1.079	-1.225	0.00172	0.0239	1.143	-1.475	0.000151	0.026
ENSMUSG00000074183	GSTA5	0.759	-1.694	0.000417	0.0102	0.667	-1.744	0.000669	0.049
ENSMUSG00000074254	CYP2A6 (includes others)	2.56	-2.088	0.00803	0.0624	2.933	-1.771	0.0273	0.277
ENSMUSG00000040660	Cyp2b13/Cyp2b9	3.395	-1.886	0.0324	0.14	3.652	-2.271	0.0218	0.252

Table S4 List of genes singificantly modulated in both Het KO (vs. WT) and with same pattern of regulation (CCL4 experiment)

		Het vs WT				KO vs WT			
**Ensembl**	**Symbol**	**Log2CPM**	**Log2FC**	**p-value**	**FDR**	**Log2CPM**	**Log2FC**	**p-value**	**FDR**
ENSMUSG00000027577	CHRNA4	2.565	2.768	5.19E-04	4.07E-01	4.47	5.769	2.11E-17	2.42E-14
ENSMUSG00000037071	SCD	11.487	1.989	3.45E-03	7.55E-01	12.884	4.128	1.17E-17	1.46E-14
ENSMUSG00000089943	UGT1A4	3.429	1.824	3.78E-08	5.65E-04	3.916	3.718	5.57E-41	8.31E-37
ENSMUSG00000079507	HLA-A	1.415	1.416	1.35E-02	9.55E-01	2.05	3.098	3.03E-05	2.29E-03
ENSMUSG00000042010	ACACB	5.194	1.33	1.22E-05	4.37E-02	6.3	3.02	9.51E-32	7.09E-28
ENSMUSG00000027605	ACSS2	7.329	1.089	1.28E-04	2.10E-01	8.248	2.988	9.67E-26	4.81E-22
ENSMUSG00000041220	ELOVL6	6.124	0.954	1.75E-02	9.73E-01	7.187	2.84	1.38E-18	1.88E-15
ENSMUSG00000025153	FASN	8.795	1.065	8.39E-03	8.89E-01	9.448	2.779	8.00E-08	1.81E-05
ENSMUSG00000090171	UGT1A3	0.205	1.268	4.90E-02	1.00E+00	1.048	2.773	8.46E-08	1.88E-05
ENSMUSG00000055254	NTRK2	1.31	1.516	8.18E-03	8.89E-01	1.534	2.753	2.92E-03	5.65E-02
ENSMUSG00000020917	ACLY	7.843	0.691	9.74E-03	9.08E-01	8.815	2.604	7.93E-20	1.69E-16
ENSMUSG00000028716	PDZK1IP1	2.152	0.838	2.82E-02	1.00E+00	3.044	2.602	7.49E-19	1.12E-15
ENSMUSG00000038418	EGR1	3.205	1.257	4.47E-02	1.00E+00	3.464	2.413	4.28E-04	1.50E-02
ENSMUSG00000021670	HMGCR	6.138	0.962	1.03E-04	1.92E-01	6.652	2.35	6.49E-06	6.63E-04
ENSMUSG00000023886	SMOC2	4.657	0.876	6.79E-03	8.88E-01	5.235	2.297	6.88E-06	6.94E-04
ENSMUSG00000024978	GPAM	5.524	0.606	1.85E-03	6.29E-01	6.094	2.108	1.89E-11	1.09E-08
ENSMUSG00000104501	Gm37736	1.453	0.657	3.21E-02	1.00E+00	1.602	2.093	1.02E-06	1.45E-04
ENSMUSG00000033105	LSS	6.24	0.8	4.75E-02	1.00E+00	6.474	1.994	3.50E-05	2.55E-03
ENSMUSG00000105837	Gm35986	0.4	1.106	8.28E-03	8.89E-01	1.049	1.975	2.02E-08	5.03E-06
ENSMUSG00000017002	SLPI	1.933	1.516	4.14E-02	1.00E+00	2.481	1.964	8.90E-03	1.14E-01
ENSMUSG00000032561	ACPP	3.246	0.78	2.59E-02	1.00E+00	3.847	1.897	1.11E-11	7.54E-09
ENSMUSG00000025429	PSTPIP2	1.89	0.71	1.37E-02	9.55E-01	2.766	1.844	1.45E-11	9.03E-09
ENSMUSG00000032418	ME1	5.873	0.457	2.44E-02	1.00E+00	6.65	1.817	3.05E-23	1.14E-19
ENSMUSG00000027346	GPCPD1	4.436	0.988	2.10E-02	1.00E+00	5.271	1.749	1.21E-12	9.06E-10
ENSMUSG00000026471	MR1	2.935	0.747	1.62E-02	9.73E-01	3.457	1.726	1.62E-10	7.05E-08
ENSMUSG00000066441	RDH11	5.985	0.694	4.67E-03	7.89E-01	6.187	1.717	9.20E-11	4.58E-08
ENSMUSG00000022883	ROBO1	2.533	0.718	2.04E-02	1.00E+00	2.879	1.708	8.35E-07	1.26E-04
ENSMUSG00000026185	IGFBP5	1.715	2.016	5.24E-03	8.50E-01	1.732	1.68	2.77E-04	1.13E-02
ENSMUSG00000113637	Gm7049	0.96	0.73	3.04E-02	1.00E+00	1.372	1.652	1.74E-06	2.26E-04
ENSMUSG00000022434	FAM118A	1.209	0.534	3.49E-02	1.00E+00	1.428	1.592	1.35E-06	1.85E-04
ENSMUSG00000028457	Atp8b5	0.979	1.371	5.17E-04	4.07E-01	1.085	1.575	1.57E-05	1.37E-03
ENSMUSG00000060548	TNFRSF19	1.898	0.903	1.09E-02	9.12E-01	2.453	1.57	2.23E-09	7.39E-07
ENSMUSG00000042216	SGSM1	1.957	0.722	1.25E-02	9.55E-01	2.271	1.518	1.45E-04	7.10E-03
ENSMUSG00000029482	AACS	5.858	1.267	7.02E-03	8.88E-01	5.437	1.497	3.26E-02	2.30E-01
ENSMUSG00000032349	ELOVL5	8.198	0.697	4.86E-03	8.06E-01	8.421	1.485	2.17E-07	4.37E-05
ENSMUSG00000100094	1810008I18Rik	5.569	0.754	2.43E-03	6.65E-01	5.816	1.45	5.95E-09	1.74E-06
ENSMUSG00000019845	TUBE1	0.313	0.813	4.16E-02	1.00E+00	0.45	1.45	6.34E-04	1.97E-02
ENSMUSG00000079470	UTP14C	2.152	0.884	1.55E-03	5.67E-01	2.188	1.403	4.28E-05	2.92E-03
ENSMUSG00000019853	HEBP2	1.623	0.798	8.87E-03	8.92E-01	1.799	1.378	5.73E-04	1.85E-02
ENSMUSG00000024899	PAPSS2	7.484	0.488	1.72E-02	9.73E-01	8.209	1.365	7.75E-10	2.69E-07
ENSMUSG00000058454	DHCR7	7.014	0.729	1.78E-02	9.73E-01	7.147	1.355	9.03E-05	4.88E-03
ENSMUSG00000023882	Zfp54	1.216	0.91	2.86E-03	7.13E-01	1.357	1.342	3.30E-05	2.46E-03
ENSMUSG00000086040	WIPF3	2.783	0.556	4.27E-02	1.00E+00	3.223	1.296	4.51E-05	3.01E-03
ENSMUSG00000044042	FMN1	2.625	0.631	1.39E-02	9.55E-01	3.093	1.264	4.57E-05	3.04E-03
ENSMUSG00000049164	ZNF518A	1.278	0.642	3.91E-02	1.00E+00	1.688	1.264	1.98E-05	1.64E-03
ENSMUSG00000039145	CAMK1D	5.804	0.426	4.92E-02	1.00E+00	6.278	1.251	1.46E-08	3.77E-06
ENSMUSG00000066554	Gm10167	1.951	0.966	8.48E-04	5.06E-01	2.023	1.242	3.31E-05	2.46E-03
ENSMUSG00000033411	CTDSPL2	0.94	0.887	2.62E-02	1.00E+00	1.18	1.216	1.51E-03	3.63E-02
ENSMUSG00000075511	1700001L05Rik	2.966	0.774	2.47E-03	6.65E-01	3.232	1.18	3.48E-06	4.14E-04
ENSMUSG00000047420	FAM180A	0.489	0.953	3.37E-02	1.00E+00	0.738	1.168	3.29E-03	6.06E-02
ENSMUSG00000032883	ACSL3	4.851	0.579	6.86E-03	8.88E-01	4.893	1.15	1.22E-04	6.19E-03
ENSMUSG00000024981	ACSL5	8.842	0.419	2.53E-02	1.00E+00	9.17	1.136	3.68E-07	6.46E-05
ENSMUSG00000032018	SC5D	8.741	0.49	2.01E-02	1.00E+00	8.936	1.131	4.17E-05	2.87E-03
ENSMUSG00000019990	PDE7B	1.19	1.122	7.81E-03	8.89E-01	1.373	1.119	3.44E-03	6.24E-02
ENSMUSG00000028051	HCN3	3.6	1.284	3.98E-02	1.00E+00	3.828	1.115	4.20E-02	2.62E-01
ENSMUSG00000031451	GAS6	5.221	0.714	1.86E-02	9.79E-01	5.572	1.079	9.06E-06	8.72E-04
ENSMUSG00000024665	FADS2	7.876	0.477	4.67E-02	1.00E+00	8.009	1.037	3.58E-04	1.32E-02
ENSMUSG00000033392	CLASP2	2.77	0.476	3.43E-02	1.00E+00	3.189	1.03	6.23E-06	6.46E-04
ENSMUSG00000022756	SLC7A4	1.251	0.595	3.12E-02	1.00E+00	1.637	1.015	6.07E-04	1.93E-02
ENSMUSG00000044254	PCSK9	5.674	0.853	1.43E-03	5.62E-01	5.695	1.009	6.64E-03	9.48E-02
ENSMUSG00000023805	SYNJ2	2.247	0.451	4.91E-02	1.00E+00	2.566	0.993	2.05E-04	9.33E-03
ENSMUSG00000079164	TLR5	1.647	0.918	1.16E-03	5.13E-01	1.567	0.981	2.34E-03	4.90E-02
ENSMUSG00000031425	PLP1	0.241	1.054	8.85E-03	8.92E-01	0.451	0.981	3.27E-02	2.31E-01
ENSMUSG00000024378	STARD4	6.148	0.653	8.57E-04	5.06E-01	6.316	0.97	1.52E-05	1.34E-03
ENSMUSG00000053846	LIPG	3.164	1.079	4.63E-03	7.89E-01	2.534	0.97	3.91E-02	2.54E-01
ENSMUSG00000018727	CPSF4L	2.099	0.726	1.27E-02	9.55E-01	2.377	0.962	1.59E-03	3.75E-02
ENSMUSG00000034926	DHCR24	9.559	0.514	4.70E-03	7.89E-01	9.798	0.942	1.90E-07	3.95E-05
ENSMUSG00000022679	MPV17L	3.681	0.582	2.58E-02	1.00E+00	3.749	0.915	4.52E-03	7.48E-02
ENSMUSG00000041801	PHLDA3	2.073	0.875	1.07E-03	5.13E-01	2.393	0.915	2.90E-04	1.16E-02

Table S4 List of genes singificantly modulated in both Het KO (vs. WT) and with same pattern of regulation (CCL4 experiment)

		Het vs WT				KO vs WT			
**Ensembl**	**Symbol**	**Log2CPM**	**Log2FC**	**p-value**	**FDR**	**Log2CPM**	**Log2FC**	**p-value**	**FDR**
ENSMUSG00000030309	CAPRIN2	1.415	1.022	5.08E-05	1.08E-01	1.013	0.908	8.12E-03	1.07E-01
ENSMUSG00000038072	GALNT11	3.599	0.59	2.83E-04	3.14E-01	3.863	0.904	1.25E-08	3.34E-06
ENSMUSG00000026773	PFKFB3	3.261	1.013	5.66E-03	8.54E-01	3.147	0.89	2.36E-03	4.93E-02
ENSMUSG00000041237	PKLR	7.439	0.596	2.75E-02	1.00E+00	7.4	0.87	7.32E-04	2.20E-02
ENSMUSG00000035914	CD276	3.088	0.503	4.81E-02	1.00E+00	3.628	0.868	2.92E-03	5.65E-02
ENSMUSG00000030800	PRSS8	3.424	0.533	3.61E-02	1.00E+00	3.512	0.867	5.09E-03	8.07E-02
ENSMUSG00000049233	Apoo-ps	0.779	0.76	3.09E-02	1.00E+00	0.958	0.867	1.95E-02	1.76E-01
ENSMUSG00000010663	FADS1	8.856	0.402	2.98E-02	1.00E+00	8.889	0.846	3.28E-04	1.25E-02
ENSMUSG00000024998	PLCE1	3.285	0.583	8.07E-04	5.06E-01	3.44	0.827	4.93E-05	3.18E-03
ENSMUSG00000006494	PDK1	5.62	0.403	2.07E-02	1.00E+00	5.597	0.809	8.17E-05	4.53E-03
ENSMUSG00000042978	SBK1	4.52	0.593	4.59E-02	1.00E+00	4.486	0.801	1.17E-03	3.08E-02
ENSMUSG00000029664	TFPI2	4.946	0.518	3.61E-03	7.55E-01	5.313	0.798	5.81E-07	9.22E-05
ENSMUSG00000019779	FRK	1.494	0.608	2.60E-02	1.00E+00	1.634	0.797	1.11E-02	1.30E-01
ENSMUSG00000021952	XPO4	2.131	0.545	2.93E-02	1.00E+00	2.352	0.79	3.21E-03	5.97E-02
ENSMUSG00000005580	ADCY9	2.744	0.615	3.96E-03	7.59E-01	2.834	0.774	9.65E-05	5.15E-03
ENSMUSG00000075543	URAD	4.2	0.529	8.93E-03	8.92E-01	4.334	0.765	2.45E-04	1.05E-02
ENSMUSG00000001870	LTBP1	2.061	0.899	2.45E-03	6.65E-01	1.777	0.76	4.77E-02	2.79E-01
ENSMUSG00000036596	CPZ	0.719	0.769	3.81E-02	1.00E+00	0.653	0.76	3.81E-02	2.51E-01
ENSMUSG00000028414	FKTN	2.672	0.514	2.21E-02	1.00E+00	2.88	0.753	1.52E-03	3.64E-02
ENSMUSG00000037458	AZIN1	5.365	0.416	3.98E-02	1.00E+00	5.708	0.751	3.11E-06	3.80E-04
ENSMUSG00000027412	LPIN3	2.609	0.444	1.83E-02	9.79E-01	2.805	0.737	2.66E-04	1.10E-02
ENSMUSG00000079037	PRNP	4.881	0.4	7.32E-03	8.88E-01	5.064	0.735	5.84E-05	3.59E-03
ENSMUSG00000032860	P2RY2	3.564	0.436	2.68E-02	1.00E+00	3.74	0.714	2.25E-04	9.95E-03
ENSMUSG00000032193	LDLR	7.062	0.449	1.86E-02	9.79E-01	7.003	0.71	1.55E-02	1.56E-01
ENSMUSG00000038145	SNRK	3.946	0.431	2.37E-02	1.00E+00	3.848	0.701	6.80E-03	9.59E-02
ENSMUSG00000036155	MGAT5	3.701	0.519	3.74E-02	1.00E+00	3.96	0.695	5.74E-03	8.74E-02
ENSMUSG00000032024	CLMP	1.531	0.563	4.52E-02	1.00E+00	1.57	0.693	4.33E-02	2.66E-01
ENSMUSG00000022894	ADAMTS5	1.514	0.635	2.35E-02	1.00E+00	1.599	0.688	2.43E-02	2.00E-01
ENSMUSG00000090623	Cfhr3	2.095	0.732	2.62E-03	6.85E-01	2.18	0.671	1.92E-02	1.75E-01
ENSMUSG00000032397	TIPIN	2.186	0.559	9.85E-03	9.08E-01	2.182	0.661	1.99E-02	1.78E-01
ENSMUSG00000021611	TERT	3.172	0.522	4.90E-02	1.00E+00	3.221	0.65	1.33E-02	1.42E-01
ENSMUSG00000015085	ENTPD2	1.834	0.555	2.87E-02	1.00E+00	1.665	0.64	3.02E-02	2.22E-01
ENSMUSG00000000056	NARF	4.93	0.471	4.27E-02	1.00E+00	5.177	0.636	1.42E-03	3.50E-02
ENSMUSG00000024135	SRBD1	4.041	0.415	1.65E-03	5.77E-01	4.001	0.583	8.07E-04	2.38E-02
ENSMUSG00000004880	LBR	2.503	0.532	2.87E-02	1.00E+00	2.43	0.581	3.67E-02	2.46E-01
ENSMUSG00000057234	METTL15	1.5	0.519	3.82E-02	1.00E+00	1.427	0.58	4.88E-02	2.82E-01
ENSMUSG00000042213	ZFAND4	4.224	0.447	3.57E-02	1.00E+00	4.17	0.579	3.09E-02	2.24E-01
ENSMUSG00000025384	FAAP100	2.524	0.51	2.01E-02	1.00E+00	2.387	0.572	3.99E-02	2.56E-01
ENSMUSG00000031583	WRN	2.942	0.392	1.36E-02	9.55E-01	2.962	0.553	3.48E-03	6.27E-02
ENSMUSG00000047230	CLDN2	6.614	0.406	2.30E-03	6.65E-01	6.716	0.549	1.13E-04	5.89E-03
ENSMUSG00000035173	CCDC186	2.594	0.531	1.64E-02	9.73E-01	2.391	0.52	4.58E-02	2.73E-01
ENSMUSG00000027165	C11orf74	3.4	0.473	1.07E-02	9.08E-01	3.531	0.515	6.28E-03	9.17E-02
ENSMUSG00000064368	MT-ND6	10.025	0.386	4.16E-02	1.00E+00	10.1	0.496	1.10E-02	1.30E-01
ENSMUSG00000044197	GPR146	6.815	0.386	4.90E-02	1.00E+00	6.899	0.484	1.38E-02	1.45E-01
ENSMUSG00000031371	HAUS7	4.027	0.411	1.66E-03	5.77E-01	3.837	0.432	4.01E-02	2.56E-01
ENSMUSG00000027801	TM4SF4	6.67	-0.398	1.61E-02	9.73E-01	6.747	-0.387	4.34E-02	2.66E-01
ENSMUSG00000027944	HAX1	3.868	-0.416	2.53E-02	1.00E+00	3.879	-0.445	9.43E-03	1.18E-01
ENSMUSG00000036887	C1QA	6.304	-0.386	2.18E-02	1.00E+00	6.306	-0.45	3.36E-02	2.34E-01
ENSMUSG00000089809	RASGEF1B	3.388	-0.929	1.46E-05	4.37E-02	3.869	-0.487	4.56E-02	2.73E-01
ENSMUSG00000053950	ADNP2	2.724	-0.615	1.56E-03	5.67E-01	2.879	-0.542	2.43E-02	1.99E-01
ENSMUSG00000031824	KIAA0513	2.673	-0.599	1.48E-03	5.65E-01	2.807	-0.543	4.98E-02	2.85E-01
ENSMUSG00000040097	FLYWCH1	2.918	-0.446	4.66E-02	1.00E+00	2.954	-0.546	2.43E-02	2.00E-01
ENSMUSG00000062753	AI413582	1.975	-0.545	2.24E-02	1.00E+00	1.972	-0.551	3.73E-02	2.48E-01
ENSMUSG00000026939	TMEM141	4.253	-0.493	9.41E-03	9.02E-01	4.216	-0.557	2.16E-02	1.86E-01
ENSMUSG00000020437	MYO1G	2.04	-0.809	2.09E-03	6.64E-01	2.478	-0.565	2.79E-02	2.14E-01
ENSMUSG00000111446	Gm6581	1.781	-0.585	7.98E-03	8.89E-01	1.918	-0.577	3.07E-02	2.24E-01
ENSMUSG00000003283	HCK	3.363	-0.621	4.60E-03	7.89E-01	3.574	-0.628	2.78E-02	2.13E-01
ENSMUSG00000071714	CSF2RB	2.155	-1.088	1.01E-02	9.08E-01	4.075	-0.638	1.65E-02	1.61E-01
ENSMUSG00000055148	KLF2	2.248	-0.552	8.93E-03	8.92E-01	2.353	-0.642	1.22E-02	1.37E-01
ENSMUSG00000071715	NCF4	1.955	-0.613	1.78E-02	9.73E-01	1.948	-0.654	4.10E-02	2.59E-01
ENSMUSG00000115300	AC126028.1	0.953	-0.597	4.60E-02	1.00E+00	1.177	-0.659	4.22E-02	2.63E-01
ENSMUSG00000049625	TIFAB	2.492	-0.959	9.47E-06	4.37E-02	2.74	-0.664	1.00E-02	1.22E-01
ENSMUSG00000017677	WSB1	4.442	-0.492	3.06E-02	1.00E+00	4.332	-0.666	8.48E-03	1.11E-01
ENSMUSG00000086825	Gm15675	1.647	-0.735	1.07E-02	9.08E-01	1.804	-0.667	3.74E-02	2.48E-01
ENSMUSG00000051495	IRF2BP2	4.781	-0.612	9.43E-03	9.02E-01	4.751	-0.703	2.06E-04	9.33E-03
ENSMUSG00000028495	RPS6	4.387	-0.513	2.70E-05	6.71E-02	4.553	-0.703	4.17E-06	4.83E-04
ENSMUSG00000017670	ELMO2	3.697	-0.603	4.23E-03	7.60E-01	3.751	-0.721	5.42E-03	8.40E-02
ENSMUSG00000019232	ETNPPL	6.21	-0.779	9.83E-04	5.06E-01	6.447	-0.723	1.55E-02	1.56E-01
ENSMUSG00000072620	Slfn2	2.984	-0.748	2.13E-02	1.00E+00	3.182	-0.728	4.57E-02	2.73E-01

Table S4 List of genes singificantly modulated in both Het KO (vs. WT) and with same pattern of regulation (CCL4 experiment)

		Het vs WT				KO vs WT			
**Ensembl**	**Symbol**	**Log2CPM**	**Log2FC**	**p-value**	**FDR**	**Log2CPM**	**Log2FC**	**p-value**	**FDR**
ENSMUSG00000023050	MAP3K12	0.915	-0.763	8.15E-03	8.89E-01	1.025	-0.728	3.14E-02	2.26E-01
ENSMUSG00000049686	ORAI1	3.466	-0.452	3.96E-02	1.00E+00	3.42	-0.734	8.16E-03	1.08E-01
ENSMUSG00000037649	HLA-DMA	2.916	-0.758	1.12E-02	9.19E-01	2.918	-0.767	7.17E-03	9.96E-02
ENSMUSG00000081094	Rpl19-ps11	2.433	-0.68	2.50E-03	6.65E-01	2.53	-0.769	3.04E-03	5.76E-02
ENSMUSG00000063696	Gm8730	1.007	-0.814	1.83E-02	9.79E-01	1.383	-0.784	3.00E-02	2.22E-01
ENSMUSG00000000682	Cd52	3.831	-0.595	1.11E-02	9.19E-01	3.816	-0.793	7.83E-03	1.05E-01
ENSMUSG00000042729	WDR74	1.355	-0.655	2.20E-02	1.00E+00	1.494	-0.801	1.72E-02	1.66E-01
ENSMUSG00000073421	HLA-DQB1	4.869	-0.452	2.40E-02	1.00E+00	4.756	-0.842	2.93E-05	2.24E-03
ENSMUSG00000059089	FCGR3A/FCGR3B	3.399	-0.839	9.95E-03	9.08E-01	3.508	-0.848	3.26E-02	2.30E-01
ENSMUSG00000048498	CD300E	1.129	-1.326	7.42E-04	5.06E-01	1.706	-0.928	2.08E-02	1.82E-01
ENSMUSG00000095687	RNASET2	1.03	-0.605	3.91E-02	1.00E+00	1.149	-0.93	1.19E-02	1.35E-01
ENSMUSG00000044145	1810024B03Rik	1.208	-0.748	9.73E-03	9.08E-01	1.273	-0.931	8.97E-03	1.15E-01
ENSMUSG00000042066	TMCC2	0.96	-0.654	3.86E-02	1.00E+00	1.097	-0.934	9.37E-03	1.18E-01
ENSMUSG00000036864	Proser3	1.916	-0.525	2.36E-02	1.00E+00	1.945	-0.95	3.03E-03	5.76E-02
ENSMUSG00000019564	ARID3A	0.679	-1.306	4.10E-04	3.60E-01	0.873	-0.971	9.49E-03	1.18E-01
ENSMUSG00000048731	Ggnbp1	3.934	-0.391	3.79E-02	1.00E+00	3.726	-1.029	1.44E-05	1.29E-03
ENSMUSG00000082393	Gm15028	1.208	-0.616	1.20E-02	9.36E-01	1.192	-1.039	3.04E-03	5.76E-02
ENSMUSG00000024426	ATAT1	3.695	-0.477	2.98E-03	7.29E-01	3.541	-1.049	1.05E-08	2.89E-06
ENSMUSG00000080316	Spaca6	2.089	-0.798	2.86E-02	1.00E+00	2.007	-1.106	7.42E-03	1.02E-01
ENSMUSG00000079003	SAMD1	3.743	-0.474	2.32E-02	1.00E+00	3.559	-1.111	9.17E-07	1.34E-04
ENSMUSG00000047067	DUSP28	2.63	-0.643	1.35E-02	9.55E-01	2.519	-1.122	2.30E-04	1.01E-02
ENSMUSG00000049184	PURG	0.343	-0.827	1.65E-02	9.73E-01	0.644	-1.125	1.23E-02	1.37E-01
ENSMUSG00000030814	BCL7C	3.608	-0.496	3.45E-02	1.00E+00	3.446	-1.158	2.23E-07	4.43E-05
ENSMUSG00000047904	SSTR2	1.439	-0.702	4.30E-02	1.00E+00	1.538	-1.162	1.50E-03	3.62E-02
ENSMUSG00000030672	MYLPF	0.449	-0.666	3.32E-02	1.00E+00	0.379	-1.171	2.53E-03	5.14E-02
ENSMUSG00000018486	WNT9B	0.459	-1.064	4.02E-02	1.00E+00	0.515	-1.176	4.09E-02	2.59E-01
ENSMUSG00000097571	Jpx	0.705	-0.821	4.17E-03	7.59E-01	0.766	-1.315	4.00E-04	1.44E-02
ENSMUSG00000115431	Gm3219	0.669	-0.795	5.89E-03	8.54E-01	0.806	-1.341	7.64E-04	2.27E-02
ENSMUSG00000078650	G6PC	8.092	-0.636	3.41E-02	1.00E+00	7.764	-1.425	1.14E-06	1.61E-04
ENSMUSG00000025396	HSD17B6	7.514	-0.844	2.27E-03	6.65E-01	7.437	-1.651	1.25E-03	3.21E-02
ENSMUSG00000096688	Mup1 (includes others)	1.03	-2.57	5.59E-03	8.54E-01	2.38	-2.541	9.55E-03	1.18E-01
ENSMUSG00000105987	AI506816	1.81	-0.989	1.27E-03	5.30E-01	0.15	-4.589	2.62E-20	6.52E-17

Table S5 List of Upstream Regulators signficantly enriched and differentially activated/inhibited in the CCl4 model (KO vs WT).

Upstream Regulator	Z-Score	p-value	Target Molecules in Dataset
**SCAP**	5.119	1.41E-23	AACS,ABCA1,ABCA3,ACAA2,ACACA,ACACB,ACLY,ACSL4,ACSL5,ACSS2,ATF5,C5,CYP2B6,DDIT3,DHCR7,EHHADH,ELOVL6,FABP5,FADS2,FASN,FDPS,GPAM,Gstm6,Gstt3,HMGCR,HMGCS1,HMGCS2,LDLR,LSS,PCSK9,PDK1,PMVK,PON1,RDH11,SC5D,SCD,Scd2,SQLE,STARD4,THRSP,TMEM97
**NR1I2**	5.053	2.43E-15	ABCA1,ABCC2,Aldh1a7,CD36,Ces2c,CES3,Cyb5r3,CYP2A6 (includes others),CYP2B6,Cyp2c23,Cyp2d9 (includes others),CYP7A1,DHCR7,EGR1,ELOVL6,ENTPD5,FASN,GLUL,GSTA5,GSTM1,GSTM3,Gstm3,Gstm6,GSTP1,Hamp/Hamp2,HMGCS1,HMGCS2,Hsd3b4 (includes others),HTT,LPCAT3,LTBP1,Mup1 (includes others),NAT8B,NR0B2,NR1I3,PAPSS2,PDIA4,PGD,PGRMC2,SCARB1,SCD,Sult1d1,UGT1A1,UGT1A3,UGT1A4,UGT1A6,Uox
**SREBF1**	4.835	8.69E-24	AACS,ABCA1,ACACA,ACACB,ACADS,ACLY,ACSL4,ACSL5,ACSS2,APOA2,APOA5,ARF4,BHLHE40,Bhlhe41,CAMK1D,CD14,CFI,CXADR,CYP7A1,CYP7B1,DHCR7,EHHADH,ELOVL6,FABP5,FADS1,FADS2,FAS,FASN,FDPS,G6PC,G6PD,GPAM,Gstm6,Gstt3,HMGCR,HMGCS1,HSPA5,IDH1,IL1B,KLK3,LDLR,LSS,MIOX,NFIL3,NPC1,NR0B2,NR1H3,PCK1,PCSK9,PCYT1A,PDK1,PKLR,PLTP,PMVK,PNPLA3,RDH11,RETREG1,RNASET2,RNF14,S100A13,SC5D,SCARB1,SCD,Scd2,SERPINA1,SERPINA3,SLC20A1,SLC22A4,SLC2A2,SQLE,STARD4,THRSP,TMEM97,TNFRSF1B
**SREBF2**	4.671	1.15E-14	AACS,ABCA1,ACAA2,ACACA,ACLY,ACSS2,APOA2,CAMK1D,CYP7B1,DHCR7,ELOVL6,FABP5,FADS2,FASN,FDPS,G6PD,HMGCR,HMGCS1,IDH1,LDLR,LSS,PCSK9,PCYT1A,PMVK,RDH11,SC5D,SCD,SQLE,STARD4,THRSP,TMEM97
**TBX2**	4.487	2.11E-09	ANLN,ASF1B,ATF5,AURKB,BHLHE40,CDCA3,CHAF1B,CHEK1,DBP,DDIT3,FOXM1,HELLS,LIG1,MCM2,MCM5,MCM6,NCAPG2,NFIL3,PCGF6,PKMYT1,PRIM2,RBL1,SMC2,TIPIN
**ERBB2**	4.304	1.49E-10	ABRACL,ACAA2,ACSL4,ACSS2,ADAMTS5,Aldh1a7,ATP6V1A,BACE1,BCL3,BHLHE40,BMP1,BMP7,BTG2,CAVIN2,CBFA2T3,CCND1,CCNE1,CD36,CDC45,CDCA2,CDCA3,CDCA7L,CDCP1,CENPF,CHD1L,CHD4,CHEK1,CHMP2B,CLU,CPS1,DDIT3,EGFR,EGR1,EHHADH,EPHX2,ERBB2,ERBB3,ESR1,FASN,FHIT,FRRS1,GAS6,GLO1,GPAM,GPC1,GPD2,HADHB,HIF1A,HMGCR,HP,HSD17B11,HSPB1,IDH1,IGFBP5,IL1B,ITGA5,JUNB,KLK3,LDHA,LIG1,LSS,MCM2,MCM3,MCM5,MCM6,MGAT5,MKNK2,MPHOSPH9,NCAPG,NDC80,NID1,NOX4,NR1H3,NUCB2,PCNA,PDIA4,PDK1,PFKFB3,PHYH,PMVK,POLA1,POLE,PRIM2,PROM1,PSMB9,QKI,RAD51AP1,RETREG1,RFC4,RPA2,RRM1,RRM2,SCARB1,SERPINA3,SHROOM3,SLPI,SMC2,SNAI1,SPAG1,SQLE,Sult1a1,TERT,THSD1,TIMP3,TJP1,TOP2A,TP53RK,TYMS,UGT1A6,USF2,USP14,WWC1
**ESR1**	3.978	6.57E-10	ABCA3,ABCG5,ABLIM1,ADAMTS5,AGT,AKT1S1,ANK3,ANLN,APOA1,APOE,ARFIP2,ARHGAP18,ARHGEF9,ARNTL,ASS1,ATF5,ATP1B1,ATP6V1A,BCAS3,BCLAF1,C3,C5,CCND1,CCNE1,CENPF,CENPK,CENPM,CENPQ,CEP135,CEP70,CHEK1,CLDN2,CLSPN,CP,CRIM1,CSNK1A1,CXADR,CYFIP2,CYP17A1,CYP2E1,CYP7A1,CYP7B1,DHCR24,DHCR7,DIAPH3,DLG1,DNAJC21,DR1,EGFR,EGR1,ERBB2,ERBB3,ESR1,F3,FAS,FASN,FMN1,FOXM1,FST,G6PD,GABBR2,GAS7,GM2A,GPAM,GSTA5,GUCY1B1,HAX1,HDAC11,HELLS,HIF1A,HMGCR,HMGCS2,HSD17B7,HSP90B1,HSPH1,IGFBP2,IGFBP5,IL1B,ISG20,JUNB,KDM4B,KIF1B,KLHL13,KLK3,LBP,LDLR,LGALS3BP,LIPC,LTBP1,MAP3K12,MAP3K20,MPDZ,MPHOSPH9,MR1,NCAPG,NCAPH,NFAT5,NR0B2,NR1D1,NR1D2,NR1H3,OPHN1,OS9,P2RY2,PBK,PCNA,PCYOX1,PDCD5,PDE4A,PGRMC2,PLCE1,PLS1,PLXNA2,POLA1,PPARD,PPP1R12A,PPP1R3C,PROM1,PTPN4,RAB11FIP4,RAD51B,RBBP4,RBL1,RBP4,RCN2,RDH16,RND1,ROBO1,ROBO2,ROCK1,RTN4RL1,SAMHD1,SCARB1,SCD,Scd2,SERPINB9,SFPQ,SIPA1L1,SLC16A2,SLC2A4,SLC44A1,Slco1a1,SMC2,SNAI1,SORD,SPAG9,SPDL1,SRPK2,STARD3,STAT1,STAT5A,TACC1,TERT,TFRC,TJP1,TMED10,TMEM97,TMPO,TNFSF10,TNS3,TRIB1,TSC22D3,UNC119,WEE1,WNT4,XRN1,YIPF3,YPEL3,ZNF367
**RABL6**	3.500	3.48E-05	AURKB,BIK,BTG2,CENPF,CHEK1,DUT,HMMR,MCM2,MCM5,MELK,NCAPG,NDC80,PBK,POLA1,TMEM97,TOP2A
**SOCS3**	3.226	5.07E-03	ABCA1,CCND1,EGR1,FASN,G6PC,IGFALS,IL1B,IL4R,ISG20,OAS1,PCK1,PCSK9,SCD,STAT5A,TNFRSF1B
**MYB**	3.214	6.74E-03	2900026A02Rik,BHLHE40,BLNK,C3,CCND1,CCNE1,CD14,DNTT,EGFR,FOXM1,GRAP,GSTM1,HSPA5,HSPA8,IGFBP5,MTSS1,PCNA,POLA1,RBL1,SLC11A2,SLC27A2,Slfn2,SNAI1,ZC3H12A
**IL2RG**	3.162	1.03E-02	CRY1,CXXC5,DNTT,ETS1,HLA-A,ILVBL,IRF7,KLF2,NEDD4L,VKORC1
**HGF**	3.078	4.92E-04	AFP,AKAP12,ANK3,ARNTL,AURKB,BMP2K,CCND1,CCNF,CDC14A,CDC45,CELSR1,CENPF,CRY1,CYP17A1,CYP2B6,DDIT3,DDX3X,DTYMK,DYNLL1,EGR1,ETS1,F3,FOXM1,G6PC,GUCY1B1,HELLS,HGF,HMMR,IL1B,ISG20,ITGB3BP,LDHA,LDLR,MCM2,MCM5,MELK,NDC80,NPC1,NR0B2,NR4A1,OCLN,PCK1,PCNA,PDGFA,PKMYT1,PLXNA2,PNRC1,PPARD,RNF103,SLC19A2,SLC20A1,SLC2A2,SMC2,SNAI1,TAGLN2,TGFBR2,TIMP3,TJP1,TMEM97,TRAF2,TRIB1
**CCND1**	3.051	2.23E-10	ATAD2,AZGP1,BCLAF1,CALU,CCND1,CCNE1,CDC45,CDCA2,CDCA7L,CENPF,CENPK,CLSPN,CPEB1,CPNE3,CTNNBIP1,DDIAS,DHCR24,DTL,E2F4,EEF1B2,EGFR,EMSY,ERBB2,ESCO2,FOXM1,GALNT11,GAS2L3,GPALPP1,H2AJ,HJURP,HSPA8,HSPB1,ID3,ITIH2,KLK3,MBLAC2,MELK,MORC4,NCAPH,NT5E,PCNA,PCYT1A,PRIM2,PSRC1,PTBP3,QKI,RAD51,RBL1,RHPN2,RPL10,RPS6,RRM2,SELENBP1,STARD4,TGFBR2,TOR3A,TP53INP1,TP53INP2,TYMP,TYMS,UHRF1,ZNF367
**SH3TC2**	3.051	9.38E-08	AACS,ACLY,DHCR24,DHCR7,EGR1,FDPS,HMGCR,HMGCS1,HSD17B7,LSS,PMVK,SC5D,SQLE
**NRG1**	3.040	1.45E-03	ABCA1,ACAT1,AMOTL2,CASP3,CAVIN2,CCND1,CCNE1,CRIM1,DDIT3,EGR1,ERBB2,ERBB3,FABP5,GAS6,GDA,GUCY2C,HEXB,HIF1A,HMGCR,HMMR,JUNB,KLK3,LPCAT3,NR4A1,PDGFA,PFKFB1,PKLR,PLG,SH2D4A,SHCBP1,SLC2A4,SNAI1,STMN1,TOP2A,USF2,ZFP36
**Pdgf (complex)**	3.015	4.86E-02	CCND1,EGFR,EGR1,F3,HIF1A,ITGA5,JUNB,LDLR,MCM6,NOX4,PCNA,TERT,TIMP3
**NR1H3**	3.003	1.47E-11	ABCA1,ABCG5,ACACA,ACSL3,ANLN,APOA1,APOE,AURKB,C1QA,CD300E,CDCA3,CENPF,CFB,CLSPN,CRP,CYP7A1,CYTIP,FASN,FDPS,FOXM1,G6PC,GAS6,GPAM,GPX1,GSTA5,GSTP1,HELLS,HMGCR,HSD11B1,IL1B,IRF7,KNTC1,LDLR,MELK,MID1IP1,NR0B2,NR1H3,NUSAP1,PFKFB1,PKLR,PLTP,RAD51,SCARB1,SCD,SLC16A2,SLC2A4,SNAI1,TOP2A,TRIB1,UHRF1
**E2f**	2.919	7.16E-04	CCNE1,CDC45,E2F4,HIF1A,ITGA5,KNTC1,LIG1,MCM2,MCM3,MCM5,MCM6,NUSAP1,PCNA,PCYT1A,PFKFB1,POLA1,RAD51,RBL1,RRM2,SKA2,TYMS
**CSF2**	2.893	4.68E-07	ABCA1,ADGRE5,ANLN,BAD,BCL3,C3,CASP3,CCND1,CCNF,CD14,CD276,CD74,CDCA2,CDCA3,CHAF1A,CHAF1B,CHTF18,CSF2RB,DDIT3,EGR1,F3,FAS,FOXM1,GABPA,GCH1,HDC,HGF,HIF1A,HJURP,HLA-DQB1,HMGCR,HSPH1,ID3,IL1B,JUNB,KNTC1,LDHA,MAP3K20,MCM3,MCM5,MCM6,NCAPH,NFE2,NR1H3,NR4A1,NUAK2,NUSAP1,PDE4B,POLE,PPIF,PRNP,QSOX1,RAD51,RBBP4,RINT1,RRM2,SERPINB9,SKA3,SLC11A2,SLC2A4,Slfn2,SMC2,SMPD2,SNTB1,STMN1,TERT,TLR5,TNFRSF1B,TOP2A,UHRF1,ZFP36
**NR1H2**	2.886	3.72E-06	ABCA1,ABCG5,ACACA,AGT,APOE,C3,CRP,CYP7A1,ELOVL6,FASN,FDPS,HMGCR,HSD11B1,IL1B,LDLR,NR1H3,PFKFB1,PLTP,SCARB1,SCD,SLC16A2,SLC2A2,SLC2A4
**MED1**	2.866	3.16E-04	ACACA,ACLY,AURKB,BCL3,BHLHE40,CCND1,CCNE1,CHAF1A,CYP17A1,CYP2B6,CYP7A1,DTYMK,EHHADH,ERBB2,FADS2,FASN,FOXM1,GADD45A,GSTP1,KLK3,LBR,NR1I3,PEX11A,SCP2,SLC2A4,TERT
**E2F1**	2.828	1.08E-06	ACSL5,ATAD2,AURKB,BAD,BCS1L,CALR,CASP3,CASP7,CCND1,CCNE1,CCNF,CD2AP,CDC45,Ces1b/Ces1c,CHEK1,CTNNBIP1,DDIT3,DUT,E2F4,EGR1,EI24,FAS,FHIT,FOXM1,GM2A,GRAP,HELLS,Hmgn2 (includes others),HSP90B1,Hspa1b,HSPA8,ID3,LACTB,MCM2,MCM3,MCM5,MCM6,MDC1,NDC80,NUSAP1,PCNA,PDCD5,PDK1,PKNOX1,POLA1,PPM1G,PRIM2,PSMB9,PTPN4,QKI,RAD51,RAN,RBBP4,RBL1,RFC4,RPA2,RRM1,RRM2,RRP1B,STAM,STMN1,TERT,TOP2A,TP53INP1,TRAF2,TYMS,UGDH,UHRF1,UNG,WEE1,ZFP36
**E2F3**	2.828	8.20E-04	CASP7,CCND1,CCNE1,CDC45,CDCA3,DEK,ECHDC2,FBLN5,FST,ID3,LBR,MCM2,MCM3,MCM5,MCM6,NCAPG2,NUCKS1,PCNA,POLA1,RAD51,RBL1,RRM1,RRM2,TERT,TIMP3,TMPO,XPO4
**Ins1**	2.762	9.09E-10	ACACA,ACADM,AGT,APOA1,APOA5,ASGR2,BAD,BDH1,CCND1,CCNE1,Cyp2d9 (includes others),CYP2E1,CYP7A1,DDIT3,DYNLL1,E2F4,ECI1,ECI2,EGR1,EHHADH,ELOVL6,ERP29,FADS1,FADS2,FASN,G6PC,G6PD,GPD1,GSTP1,HMGCR,HMGCS1,HP,IGFBP2,IGFBP5,IL1B,JUNB,LDHA,LDLR,LGALS3BP,NR1H3,PCK1,PCNA,PKLR,PPARD,PRKCA,RBL1,RETSAT,RRM1,RRM2,SCD,Scd2,SERPINA12,SLC2A2,SLC2A4,STAT5A,THRSP,TIMP3,TRAF2
**ERG**	2.746	2.28E-02	CAMK1D,CD164,DIAPH3,ETS1,GMFB,GUCY1B1,HMMR,KLK3,LAMA5,MYO1D,NFIL3,PCNP,PHACTR2,PREX2,PSTPIP2,PTPN4,RCAN2,RRAS,SIRT5,SNAI1,TACC1,TGFB1I1,TGFBR2,TSC22D3,WDR91
**NR4A1**	2.736	3.00E-05	ABCG5,ACACA,ACLY,ACSL4,APOE,CCND1,CD36,COX6B2,CYP17A1,DDIT3,DLAT,EHHADH,FASN,G0S2,G6PC,G6PD,GPD1,HDC,HIF1A,IL1B,KRT8,LDLR,LIPC,NDUFA1,NOL3,NT5E,NUCB2,OCLN,PCK1,PCNA,PDIA3,PDK1,PHKB,PKLR,PPP1R3C,RBKS,RPIA,RPS6KA1,SCD,SLC2A4,TJP1,TNFSF10
**LONP1**	2.735	7.49E-08	ACADS,ACAT1,CALU,CHAC1,CLPX,ECI1,GFER,GPT2,HSPB1,MARS1,MRPL18,MT-ATP6,MT-CO1,MT-ND1,MT-ND2,MT-ND5,MT-ND6,MTERF2,PDIA3,SARS1,SSBP1,VDAC1
**LRP1**	2.735	3.73E-02	ABCG5,C1R,CD14,DDIT3,IL1B,SERPINF1,SERPING1,SLC2A4
**NCOR-LXR-Oxysterol-RXR-9 cis RA**	2.720	1.17E-06	ABCA1,ABCG5,ACACA,APOA5,APOE,CD36,CYP7A1,FASN,PLTP,SCD,UGT1A3
**LHX1**	2.704	3.49E-02	ASS1,ATP1B1,BHMT2,CLDN2,DPP4,GATM,GSTA5,Hsd3b4 (includes others),PKDCC,PRXL2A,RBM47,Sult1d1,UGT1A3,ZDHHC21
**FOXM1**	2.672	2.59E-03	AURKB,CCND1,CCNE1,CCNF,CDCA2,CENPF,CRP,CYP7A1,ESR1,FOXF1,FOXM1,LDHA,PCNA,PDGFA,PROM1,SFTPD,SNAI1,STMN1,TGFBR2,TJP1,TOP2A
**DBI**	2.646	1.07E-05	ACSS2,ELOVL6,FASN,HMGCR,LSS,PPARD,SQLE
**SIRT2**	2.646	4.15E-04	AACS,DHCR7,HMGCR,LSS,PMVK,SC5D,SQLE
**CASR**	2.592	1.13E-03	ABCC2,ANLN,BMP7,CCND1,CDC45,CHAF1A,CHAF1B,CYP2B6,EGFR,EGR1,GABBR1,GABBR2,GADD45A,Hmgn2 (includes others),IGFBP5,MYD88,NUSAP1,PON1,RAD51AP1,SERPINA1,TYMS,WSB1
**GRP**	2.586	6.39E-03	CCND1,CCNE1,IL33,KLK3,PRKCA,RRM1,RRM2
**MLXIPL**	2.561	1.57E-08	ACACA,ACACB,ACLY,ELOVL6,FASN,G6PC,GPD1,HIF1A,MID1IP1,MIOX,PKLR,SCD,SLC2A2,THRSP
**Srebp**	2.557	1.10E-05	ACLY,ACSS2,FASN,G6PD,IDH1,LDLR,NPC1,PGD
**RPTOR**	2.550	4.52E-05	ACADM,Acp5,CCND1,DHCR24,DHCR7,FDPS,HIF1A,HMGCR,HMGCS1,HSD17B7,IL1B,LBR,LDHA,LSS,MT-CO1,PMVK,RPS6,SC5D,SLC2A4,SQLE
**STAT6**	2.528	1.15E-03	ABLIM1,Acp5,ADAM11,APOE,ASS1,BCL3,CAMK1D,CLDN2,EGR1,EHHADH,FADS1,FAM118B,FAM126A,FASN,FCGR3A/FCGR3B,FNIP2,GATM,GBP5,HCK,HLA-DMA,HMGCR,HMGCS2,HSD3B1,Igtp,IL1B,IL33,IL4R,IRF7,IRF9,Irgm1,ISG20,LACC1,LACTB,LGALS3BP,LMO7,LPIN2,NFIL3,PDGFA,PLS1,PRKCA,PRNP,SAMD4A,Scd2,SELENBP1,SERPINA1,SERPINF1,SLC25A25,STAT2,STAT5A,TMED8,USP2,ZFP36
**TCF7L2**	2.478	3.15E-06	1700047M11Rik,ACSL3,ANLN,ATP6V1G1,BACE1,C5orf51,CCND1,CLDN2,CYP2E1,CYP7A1,DHCR24,DRAM2,EGFR,EHHADH,ENTPD5,ERBB3,FAM91A1,G6PC,GLUL,GSS,HMGCS2,ID3,IDH1,IPO13,KDM4B,ME1,MYRF,NPC1,PCK1,PICALM,PIGA,PLEKHB1,PLEKHG1,PLP1,QKI,RAP2A,RASGEF1B,RNF13,ROCK1,SC5D,SECISBP2L,SLC17A2,SLC27A2,SLC44A1,SUZ12,TAF10,TMEM141,TMEM63A,TPRN,TXNDC16,UTP14C
**MBTPS1**	2.449	2.58E-04	ACACA,FASN,FDPS,HMGCR,LDLR,SCD
**DAP3**	2.449	2.58E-04	MT-ATP6,MT-CO1,MT-ND1,MT-ND2,MT-ND5,MT-ND6
**EWSR1**	2.412	4.85E-02	CCND1,ERBB2,NUSAP1,PBK,RRM2,TOP2A
**MLX**	2.394	9.03E-06	ACACA,ELOVL6,FASN,LGALS3BP,MID1IP1,PKLR,SCD,SLC2A2,THRSP
**PPARGC1B**	2.356	9.81E-11	ABCA1,ACACA,ACACB,ACADM,ACADVL,C3,CCND1,CS,DHCR24,ERBB2,FASN,FDPS,G6PC,GPAM,HMGCR,LSS,MT-ATP6,MT-CO1,MT-ND1,MT-ND2,PCK1,PPARGC1B,SCD,SLC2A4,SQLE
**EP400**	2.345	3.20E-04	AGPAT2,CCNF,CDCA3,CENPF,FOXM1,MCM3,NCAPG2,PCNA,PSRC1,RBL1,UHRF1
**LLGL2**	2.333	3.72E-04	AMOTL2,CRIM1,GAS6,HEXB,HMMR,SH2D4A,SHCBP1,STMN1,TOP2A
**ACKR2**	2.333	5.76E-03	ADAR,Ifi27l2a/Ifi27l2b,Ifi47,IL1B,IRF7,ISG20,OAS1,STAT1,STAT2
**Nr1h**	2.319	9.66E-04	ABCA1,ABCG5,ACACA,ACACB,ACLY,ACSL3,AGT,APOE,ATP1B1,BCL3,C3,CYP7A1,ELOVL6,FASN,G6PC,GPAM,HCK,IL1B,NPC1,PLTP,SCARB1,SCD,SLC2A4,TIMP3
**AR**	2.294	4.24E-07	ABCA1,ACP3,Akr1c19,AQP8,ATAD2,ATP2A2,AVPR1A,AZGP1,C3,CALU,CAST,CCND1,CDO1,CHTF18,CYP17A1,DHCR24,DHCR7,DISP2,DR1,DSC2,EGFR,EGR1,EPPK1,ERBB2,ERBB3,EXTL2,FABP5,GAS6,GPD1,GSTM1,GSTP1,HGF,HMGCR,HSD3B1,HSPA5,HSPH1,IGFBP5,KDM4B,KLK3,KNTC1,LAMA5,MCM2,NPC1,NUSAP1,OCLN,OVOS2,P4HA1,PBK,PDE9A,PDGFA,PIGA,PIGR,PKMYT1,PRKCA,SCAP,SERPINA12,SERPINA3,SLC22A4,SLC43A1,SLC45A3,SLC7A4,SNAI1,SORD,STAT1,Sult5a1,TCF19,TERT,TGFBR2,TIMP3,TJP1,TNFSF10,WEE1
**CNR1**	2.261	3.18E-02	ACACA,APOE,BMP7,CDCA3,CREB3L3,ERBB2,FASN,FOXM1,GLO1,LAMA1,LIPC,LRP4,PCNA,PIK3C2A,RXRG,SCARB1,SLC2A4,TJP1
**E2F2**	2.236	4.49E-03	CCND1,CCNE1,CDC45,MCM2,MCM3,MCM5,MCM6,PCNA,POLA1,RAD51,RBL1,RRM2,TERT
**EIF4G1**	2.236	2.62E-02	CHEK1,GADD45A,HIF1A,RAD51,RFC4

Table S5 List of Upstream Regulators signficantly enriched and differentially activated/inhibited in the CCl4 model (KO vs WT).

Upstream Regulator	Z-Score	p-value	Target Molecules in Dataset
**Vegf**	2.222	7.02E-04	AKAP12,AQP4,ARHGAP18,ARNTL,AURKB,BMP2K,BMP7,CASP3,CCNF,CDC14A,CDC45,CELSR1,CENPF,CRY1,DDIT3,DDX3X,DTYMK,EGR1,ERBB3,ETS1,F3,FAS,FOXM1,FST,GUCY1B1,HDC,HELLS,HIF1A,HMGCS2,HMMR,ISG20,ITGB3BP,LDLR,LPAR1,MAZ,MCM2,MCM5,MELK,MFSD4A,NDC80,NLRP12,NPC1,NR4A1,PABPN1,PDE7B,PDGFA,PKDCC,PKMYT1,PLXNA2,PPARD,SLC19A2,SLC20A1,SMC2,SNRK,Sult1d1,TGFBR2,TJP1,TLR3,TPST1,TRIB1,UGCG
**CCNE1**	2.219	3.32E-03	CCNE1,CDC45,HIF1A,MCM2,PCNA,PCYT1A,RBL1
**TFDP1**	2.213	4.85E-02	CASP3,CASP7,CCND1,CCNE1,RBL1,STMN1
**SPZ1**	2.197	2.16E-02	CCND1,HIF1A,PCNA,PROM1,SNAI1
**ETV1**	2.191	3.71E-02	ACSL3,ERBB2,HSD17B7,TERT,TGFBR2
**PROC**	2.178	2.66E-02	AGT,CALR,CLU,F2,IL1B,NR4A1,PCNA,TJP1
**VDR**	2.171	2.29E-02	ACACA,AGT,ANLN,BMP7,CCND1,CD14,CDC45,CHAF1A,CHAF1B,CYP2B6,EGFR,EGR1,F3,FGG,GADD45A,GPD1,Hmgn2 (includes others),IGFBP5,LDLR,MYD88,NR0B2,NUSAP1,RAD51AP1,SERPINA1,SLC51B,Slco1a1,TERT,WSB1
**AREG**	2.086	1.38E-03	AURKB,C3,CCND1,CCNF,CDC45,CENPF,EGFR,EGR1,FOXM1,HJURP,IQGAP3,PTBP2,RRM2,SAE1,SSBP1,TOP2A
**ZBTB17**	2.000	1.69E-05	ANLN,AURKB,BHLHE40,CCND1,CENPF,DDIT3,EGR1,FOXM1,HMMR,IQGAP3,KIF18B,LDLR,NCAPH,NDC80,NUSAP1,PBK,PSRC1,TOP2A,ZFP36
**LAMTOR1**	2.000	1.16E-02	ACACA,FASN,SCD,SQLE
**SEL1L**	1.982	4.90E-02	DDIT3,DNAJB11,DNAJC3,HSPA5
**YBX3**	1.972	1.67E-05	CCND1,CLDN2,ERBB2,ERBB3,HLA-DQB1,PCNA
**KISS1**	1.970	1.14E-03	Abca8a,EGR1,GSTP1,KLK3,MT-ND2,RABEP2,TMEM144
**MRPL12**	1.969	2.12E-03	MT-CO1,MT-ND1,MT-ND2,MT-ND6
**E2F4**	1.966	1.83E-08	ANLN,ASF1B,AURKB,CCND1,CCNE1,CHEK1,DDX28,DUT,EMC4,FAS,FOXM1,HMMR,HP,HSP90B1,ID3,LPAR1,MCM3,MCM5,MCM6,NDC80,PCNA,POLA1,PRIM2,RAD51,RAD51AP1,RBBP4,RBL1,RFC4,RRM1,RRM2,SFPQ,SKA2,SMC2,STAM,STMN1,SUPT4H1,SUZ12,TERT,TMPO,TOP2A,TP53INP1,TRAF2,UHRF1,UNG
**CD3 group**	1.961	2.55E-03	ACSL3,ACSL4,ACSL5,CCNE1,FASN,IL1B,KLF2,LDHA,LPCAT3,SCD,TFRC
**NKX2-3**	1.961	2.37E-02	ANLN,BCHE,BTG1,CAVIN2,CBS/CBSL,CD36,CXADR,FAM172A,GCA,GCH1,HELZ2,HMMR,KLF2,MYD88,PAPSS2,PSMB9,RPL23,SHFL,STAT1,STAT2,TCTEX1D2,TFPI2,TIPARP,TRIM21,TYMP,UACA
**YAP1**	1.954	3.11E-09	ACACA,ACAT1,ACAT2,ACLY,AMOTL2,ANLN,AURKB,CASP3,CAVIN2,CCND1,CCNF,CD36,CRIM1,DIAPH3,DTYMK,EGFR,EGR1,FASN,FOXM1,GADD45A,HMGCR,HMGCS1,HMGCS2,IL1B,ITGA5,LDHA,LMNB1,MCM3,MCM6,OCLN,POLA1,RAD51,RRM1,RRM2,SYNE3,TOP2A,TYMS,UHRF1,WWC1
**PPARG**	1.951	1.29E-11	ABCA1,ABCA3,ACAA2,ACACA,ACADM,ACADS,ACLY,ACSL4,ACSL5,AGPAT2,AGT,APOA1,APOA2,APOE,ATP2A2,BACE1,BDH1,C3,CAVIN2,CCND1,CD36,CS,DHRS3,DLAT,DR1,EGFR,EHHADH,ELOVL3,ELOVL6,ETS1,FABP5,FASN,FDPS,FST,G6PC,GPAM,GPD1,HADHB,HGF,HMGCR,HMGCS2,HP,HYOU1,IDH1,IGFBP5,IL1B,IL33,LDLR,LOC102724788/PRODH,ME1,MKNK2,MYD88,NOX4,NR1D1,NR1H3,NR4A1,OCLN,OXR1,PCK1,PCNA,PEA15,PEX11A,PFKFB3,PHKB,PLIN4,PLIN5,PMVK,PPARD,PPARGC1B,RBP4,SCARB1,SCD,SCP2,SERPINA1,SLC25A20,SLC2A2,SLC2A4,SLC44A1,TGFBR2,TJP1,TKT,TNFSF10,TSC22D3,UGT1A10 (includes others)
**SFN**	1.937	5.20E-03	AMOT,BTG2,DSC2,ERBB2,F3,KRT8,TJP1
**INHA**	1.930	3.74E-02	CCNE1,CERT1,COL4A5,CYP17A1,EGFR,FGFR4,FST,HSD17B11,KRT8,LTBP1,TBCEL,TJP1,TP53INP1,TUBE1
**TRIM24**	1.909	5.22E-03	BLNK,GLUL,Ifi27l2a/Ifi27l2b,Ifi47,Igtp,IRF7,IRF9,IRGM,Irgm1,ITIH2,LGALS3BP,OAS1,PSMB9,SAMHD1,STAT1,STAT2
**Pkc(s)**	1.908	2.22E-05	ABCA1,ACAT1,APOA1,AQP4,ARNT,BIK,CD36,CYP2A6 (includes others),CYP2E1,DBP,DDIT3,DMD,DNAJC3,EGFR,EGR1,F3,FAS,GADD45A,HIF1A,HMGCR,HSP90B1,HSPA5,IL1B,ITGA5,JUNB,KRT8,MAFK,NOX4,NR1D1,NR4A1,PDIA4,PON1,PRKCA,RBL1,S100A1,SCARB1
**PCGEM1**	1.832	1.33E-03	ACACA,ACLY,CS,DHCR24,FASN,G6PD,IDH1,LDHA,LSS,PGD
**IL10RA**	1.808	6.61E-05	ACSS2,ASS1,C3,CBS/CBSL,CD36,CELA1,CFB,CYFIP2,CYP2C18,ENTPD5,FABP5,FAS,FOXF1,GAS6,GBP5,GDA,GSAP,GSTM1,GSTM2,Gstm3,Gstm6,HGF,HLA-A,HSPA8,Ifi47,IL1B,IRF7,IRGM,Irgm1,KLK3,LRG1,MFSD4A,MLKL,MSRB1,NR1H3,OCLN,OGN,PCK1,PLAAT3,PSMB9,SAMHD1,SELENBP1,SERPINB9,STAT1,Sult1a1
**CG**	1.804	6.17E-06	ACP3,ADH7,Aldh1a7,ARNT,BHLHE40,BLM,BTC,BTG1,BTG2,CBFA2T3,CLU,CREM,CYP17A1,DHCR7,DMD,DTL,EGR1,FABP5,FAS,FST,G0S2,G6PC,GM2A,GPC1,HIF1A,HLA-A,HLA-DQA1,HMGCR,HSD11B1,HSD17B7,HSD3B1,Hsd3b4 (includes others),IGFBP2,IL1B,IL33,IL4R,JUNB,LDLR,LGALS3BP,NR2F6,NR3C2,NR4A1,NUCB2,PCNA,PFKFB3,RGS4,SCARB1,SCUBE1,SLC20A1,SRXN1,TFPI2,TIPIN,UNG,WNT4
**CD24**	1.794	1.09E-02	ATP13A3,BCLAF1,BIK,CHAC1,DLG1,HMMR,HSD17B6,ISG20,KNTC1,N4BP3,OGG1,SFPQ,TFPI2,TMEM135,TOP2A,TP53INP1
**IL15**	1.782	1.06E-02	ACADS,ARNT,BTG1,CASP3,CCND1,CD74,DHRS4,DPP4,ERBB2,ETS1,FAS,FCGR3A/FCGR3B,G6PD,GLDC,GPC1,GPD2,HAT1,HSPA8,JUNB,KLF2,LDHA,MCM5,MLLT3,MYD88,NME4,NR3C2,OGN,PCNA,PDCD5,PDE4B,PDIA3,PDIA4,PDK1,PFKFB3,PGD,PKMYT1,PPARD,PPARGC1B,PSMB9,RPIA,RPS6,TFRC,TGFBR2,THPO,TJP1,TKT,TNFSF10,TOP2A,ZFP36
**CDK4/6**	1.772	9.45E-03	BCLAF1,CALU,CCND1,CDC45,EEF1B2,EMSY,HSPB1,ITIH2,PCYT1A,RHPN2,RPL10,RPS6
**FOXA3**	1.766	8.72E-03	ACADM,Cyp2a12/Cyp2a22,CYP2C18,ELOVL3,G6PC,KLHL13,PCK1,SLC2A2
**HTT**	1.756	6.54E-07	ABCA1,ACADM,ACAT2,ADAMTS5,AGT,AKAP12,APOA1,APOE,ARNTL,ATP2A2,ATP6V0E2,AZIN1,BCL3,BHLHE40,BMP1,C1R,CASP3,CAST,CCND1,CD74,CHKA,COX6B2,CYFIP2,DBP,DDIT3,DHCR7,DNAI1,DNAJB1,DNAJC3,ECI1,EGR1,ETNPPL,FASN,FDPS,GAS7,GCLC,GLO1,GLUL,GPC1,GSS,GSTA5,Hamp/Hamp2,HIF1A,HMGCR,HMGCS1,HRAS,Hspa1b,HSPA5,HSPA8,HTT,IGFBP5,JUNB,KAT2A,KCNC3,LDHA,LDLR,Mup1 (includes others),NDUFA1,NFIL3,NR1D2,NR4A1,NTRK2,PCNA,PDE4B,PDK1,PEA15,PITPNM1,PLOD3,PPARD,PPARGC1B,PROM1,PSMB9,RBP4,RGS4,RXRG,Scd2,SERPINA1,SERPINA3,SERPINF1,SERPING1,SFPQ,SIRT3,SLC25A22,SMC2,STMN1,STX1B,SYVN1,TFRC,THRSP,TIMP3,TUBA4A,UBE2G2,UBE3A,USP2
**ARNTL**	1.749	5.90E-06	ACACA,ARNTL,CRY1,DBP,ELOVL6,FASN,GPAM,HIF1A,HMGCS2,IL1B,NR1D1,PER3,PPARGC1B,SCD,WEE1
**Gcg**	1.732	9.68E-13	ADGRV1,AQP8,ASS1,ATP1B1,CBS/CBSL,CPS1,CYP17A1,ERO1B,ETNPPL,FABP5,GAS2,GSTA5,GSTM1,Hamp/Hamp2,HSD17B6,ITIH5,ME1,MMD2,Mup1 (includes others),PDE4B,PEX11A,SLC38A4,Slco1a1,SULF2,Sult5a1,TFRC,TNFRSF19
**SGPP2**	1.698	1.42E-02	DNAJC3,Hspa1b,HSPA5,HSPA8,HSPH1,IL1B
**SLC9A3R1**	1.698	2.62E-02	ABCC2,EGFR,ERBB2,ERBB3,SCARB1
**BARX2**	1.698	4.90E-02	ANLN,DYNLL1,ESR1,TIMP3
**IGF1**	1.674	4.51E-06	ABCA1,ACACA,ACSS2,AQP4,ASGR2,BAD,BHLHE40,BTG2,C1QA,CASP3,CAVIN2,CCND1,CCNE1,CLU,CYFIP2,CYP17A1,DAXX,DDIT3,DMD,EGR1,ESR1,F3,FASN,GADD45A,GPD1,GPD2,HDC,HGF,HIF1A,HMGCR,HSD11B1,HSD17B11,HSD3B1,HSPA5,IGFBP2,IGFBP5,IL1B,IL4R,ITGA5,JUNB,KAT2A,LDHA,NFE2,NOX4,NR4A1,NTRK2,OCLN,PBK,PCNA,PLP1,PRNP,PSMB9,SCD,SLC20A1,SLC23A2,SLC25A25,SLC2A4,SLPI,SQLE,THRSP,TJP1,TMED1,UGCG,WEE1,WNT4,YPEL3,ZFP36
**GCK**	1.673	3.72E-04	ACADM,ACLY,FASN,G6PC,PCK1,PKLR,SCD,Scd2,SLC2A2
**ZNF281**	1.673	2.80E-02	BLM,Ccl9,GADD45A,MDC1,PROM1,XRCC4
**PPARD**	1.657	4.57E-14	ACAA2,ACACA,ACACB,ACADVL,APOA1,APOA2,APOE,C11orf86,C1QA,CCND1,CCNE1,CD36,CYP2B6,DET1,ECH1,ECI2,FAM241B,FASN,GAS6,GPAM,GPD1,GPD2,GSTA5,HMGCS2,IGFBP2,IL1B,INHBE,Irgm1,KYAT3,LDHA,LGALS4,LIPG,LPCAT3,LPIN2,MMD2,OCEL1,PCNA,Pcp4l1,PCSK9,PCYOX1,PER3,PKLR,PLP1,PPARD,PPARGC1B,SCD,SIRT5,SLC25A20,SLC27A2,SLC2A2,SLC2A4,SORD,STAT1,TLR5
**EGF**	1.637	1.81E-05	ACSL4,AFP,APOA1,ATP2A2,AURKB,BTG2,CASP3,CAVIN2,CCND1,CCNE1,CLDN2,CLU,CREM,CYP2B6,DDIT3,DNAI1,DPP4,EGFR,EGR1,ERBB2,ESR1,ETS1,F3,FASN,FST,GADD45A,GSTP1,HGF,HIF1A,HMGCS1,HRAS,IDH1,IGFALS,IGFBP2,IGFBP5,IL1B,JUNB,KLK3,LDHA,LPCAT3,MAP3K20,NPR2,NR4A1,OCLN,PBK,PCNA,PFKFB1,PLAGL1,PPARD,PROM1,RPS6KA1,RRM1,RRM2,SCD,Scd2,SERPINA1,SERPINA3,SLC2A4,SMAD1,SNAI1,STAT1,TERT,TFPI2,TGFBR2,TIMP3,TJP1,TJP3,TRAF2,ZFP36
**PDGF BB**	1.630	1.09E-07	ACAT2,AKAP12,AKR7A2,ASAH2,ATP2A2,AVPR1A,BCL3,BHLHE40,C3,CCND1,CCNE1,CDO1,CP,DDIT3,EGFR,EGR1,ETS1,F3,FASN,FBLN5,GADD45A,GDA,GLUL,GSS,GUCY1B1,HMGCR,HMGCS1,HRAS,ID3,IL1B,ITGA5,JUNB,LBP,LDLR,MAFK,MCM6,NFIL3,NOX4,NR4A1,NXF1,PCNA,PDGFA,PDLIM1,PPARD,RPS6,RXRG,SCD,Scd2,SERPINA3,SNAI1,THPO,TRA2B,TRIB1,UGDH,ZFP36
**PRDM16**	1.628	4.85E-02	AGT,ELOVL3,HGF,IRF7,STAT1,STAT2
**WT1**	1.610	1.95E-05	ADGRE5,BTG2,CBS/CBSL,CCND1,CCNE1,CD2AP,CDC45,CHAF1B,CMPK1,CUL4B,DDX3X,EGFR,EGR1,ERBB2,ESR1,ETS1,FDPS,HSP90B1,HSPG2,IL1B,JUNB,LSS,MED13,NQO2,NTRK2,OS9,PDGFA,PDIA4,RAB40C,ROCK1,Scd2,SLC20A1,SQLE,TERT,TIMP3,TRIM21,WDR74,WNT4
**PAX7**	1.603	4.49E-03	ABCA1,ASS1,CAMK1D,DHX40,FRK,GCH1,ID3,IL13RA1,MYO1D,PEG3,PLAGL1,PTGR1,SLC44A1
**PXR ligand-PXR-Retinoic acid-RXRα**	1.602	4.04E-05	ABCC2,Aldh1a7,CES3,CYP2A6 (includes others),CYP2B6,CYP7A1,GSTM1,GSTM2,PAPSS2,SCD,UGT1A1,UGT1A10 (includes others)
**PSEN1**	1.589	1.81E-03	ANKS4B,APOE,ARF4,ATP1B1,ATP2A2,ATP6V1A,BACE1,C1QA,C1R,C3,CASP3,CCND1,CD74,CS,CYFIP2,CYTIP,DBP,EGFR,EGR1,EXTL2,Fus,GAS5,GPANK1,GSTP1,H2AJ,HSPA5,HSPA8,IL1B,IRF7,Meg3,MT-CO1,OCEL1,Pcp4l1,PEA15,PER3,QDPR,SCARB1,SLC11A2,SRD5A3,STIM2,STIP1,STMN1,STX1B,TGFBR2,TIPARP,TUBA4A,UEVLD,VDAC1
**NCOR1**	1.572	5.45E-07	ACP3,AFP,AZGP1,BCL3,CCND1,CREM,CYP7A1,DHCR24,ELOVL3,FASN,G6PC,HP,IL1B,KLK3,ME1,MRPL49,NR1D1,PDE9A,PFKFB1,SCAP,SERPINF1,SORD,THRSP
**TP73**	1.554	9.14E-06	AFP,ARNTL,BHLHE40,BMP7,BTG2,C1R,C3,CASP3,Ccl9,CCNF,CLMN,DBP,DDIT3,DHRS3,DLG1,DTYMK,EGFR,EGR1,FAS,FASN,FOXF1,FST,G0S2,G6PD,GADD45A,HIF1A,IGFBP5,IL1B,IL4R,ITGA5,LDHA,LIG1,LRG1,LTBP1,MCM6,Meg3,MPZL2,P2RY2,PDGFA,PDK1,PNRC1,PPL,PSRC1,RAN,RB1CC1,RTN4,SERPINA1,SERPINA3,SERPINF1,SERPING1,SNAI1,SNRK,SPHK2,STMN1,TERT,TIMP3,TNFRSF1B,WNT4
**MYOD1**	1.553	2.45E-06	ACACA,ACACB,ACSL5,AGT,ARID3A,ARNT,ASS1,CCNE1,CDCA3,CDO1,CENPF,DMD,EGFR,ESR1,FOXM1,GADD45A,GREM2,HDAC11,HJURP,IGFBP5,JUNB,KIF18B,MYLPF,NCAPG,NCAPH,NDC80,NUSAP1,PBK,PDGFA,RRAS,RRM2,SBK1,SHCBP1,SLC2A4,SPC24,STAM,STAT1,TJP1,TKT,TOP2A,TRIM21,TROAP
**Jnk**	1.540	1.23E-02	ABCA1,AGT,ANXA5,BTG1,CASP3,CCND1,CCNE1,CLDN2,DDIT3,E2F4,EGR1,ETS1,F3,FAS,GADD45A,HSPA5,IL1B,NOX4,NR4A1,OCLN,PLG,PPARD,RAD51,SESN2,TERT,TGFBR2,ZFP36
**CCNK**	1.539	2.31E-03	ATR,DDIT3,MDC1,PLTP,RFC4,RPA2,SNAPC5,TAF10
**PRKCI**	1.532	7.77E-03	CCND1,FASN,GMFB,PLS1,RFC4,SLC2A2,SLC2A4
**MITF**	1.519	1.28E-02	Acp5,APEX2,APOE,AURKB,CAPN3,CCNF,CDCA3,CENPF,CENPM,CHAF1A,CHKA,CHTF18,CREM,FRMD4B,GM2A,HIF1A,LIG1,MCM2,MCM5,MCM6,MDC1,QDPR,SCARB1,SERPINF1,SLC19A2,SNAI1,SPC24,TERT,TMCC2
**FSHR**	1.510	6.13E-04	ACP3,FABP5,G6PC,GDE1,HSD3B1,Hsd3b4 (includes others),PCK1,PDGFA,PPP1CB,SLC7A4,TJP1,ZNF519
**AKT1**	1.509	6.75E-06	ACAT2,ACLY,ASS1,AURKB,BAD,CASP3,Ccl9,CCND1,CCNE1,CD74,Ces1b/Ces1c,CLU,CST3,CYP7A1,DHCR7,DIAPH3,EGR1,ESR1,FABP5,FAS,FASN,G6PC,GATM,GCLC,GSS,GSTA5,HIF1A,HMGCR,HMGCS1,IGFBP5,LAMA1,LSS,ME1,NDC80,NOX4,PCK1,PEX11A,PLG,PLP1,PROM1,RFC4,SCD,SERPINF1,SNAI1,TNFSF10,TYMS
**ERBB3**	1.469	9.58E-06	AMOTL2,CCND1,CLU,EGFR,EGR1,ERBB2,ERBB3,F3,GAS5,GAS6,HEXB,HIF1A,HMMR,HP,IGFBP5,NID1,RRAS,SERPINA3,SLPI,SNAI1,STMN1,TIMP3,TOP2A,USP14
**TMBIM6**	1.467	1.41E-02	DDIT3,ERO1B,G6PC,HSPA5,MAP1LC3A
**NR1I3**	1.461	4.09E-09	ABCC2,Aldh1a7,APOA1,Ces2c,CYP2A6 (includes others),CYP2B6,CYP7A1,CYP7B1,EI24,FASN,G6PC,GSTA5,GSTM1,Gstm3,GSTM4,GSTP1,LEAP2,LRG1,PAPSS2,PCK1,PGD,RPA2,SCD,Sult1a1,Sult1d1,THRSP,TUBA4A,UGT1A1,UGT1A3
**POU2F1**	1.452	1.93E-04	Aldh1a7,AOX1,CCND1,CDO1,CRP,CYP2A6 (includes others),CYP2B6,ESR1,FRRS1,GADD45A,GAS5,GAS6,GSTM1,GSTM3,GSTM4,IDH1,IGKC,KLK3,PDE9A,SERPING1,SESN2,TIMP3

Table S5 List of Upstream Regulators signficantly enriched and differentially activated/inhibited in the CCl4 model (KO vs WT).

Upstream Regulator	Z-Score	p-value	Target Molecules in Dataset
**IL4**	1.445	1.66E-02	ABCA1,ABLIM1,Acp5,ACSL4,APOE,ASS1,BCL3,BHLHE40,C3,CAMK1D,CASP3,CCND1,CCNE1,CD14,CD276,CD36,CD74,CHD4,CLDN2,CPEB3,CSF2RB,CYP2E1,DPP4,EHHADH,F3,FADS1,FAM118B,FAM126A,FAS,FASN,FCGR3A/FCGR3B,FNIP2,GATM,GBP5,GCAT,GSTT2/GSTT2B,HCK,HGF,HLA-DMA,HMGCR,HSD11B1,HSD3B1,ID3,Igtp,IL13RA1,IL1B,IL33,IL4R,IRF7,IRF9,Irgm1,ISG20,JUNB,KLK3,LACC1,LACTB,LGALS3BP,LIPG,LMO7,LPIN2,MCM5,MPHOSPH9,NCBP1,NFIL3,PDE4B,PDK1,PIGR,PKNOX1,PLS1,PMVK,POLA1,PPARGC1B,PPM1G,PRKCA,PRNP,PRSS8,PTBP2,RIDA,RPS6,Rrbp1,SAMD4A,SCARB1,Scd2,SDF2L1,SLC23A1,SLC25A25,SPHK2,STAT1,STAT2,STAT5A,TFRC,TGFBR2,TIMP3,TLN2,TMED5,TMED8,USP2,ZFP36
**USF1**	1.434	1.40E-05	ABCA1,AGT,AKAP12,APOA2,APOA5,CBS/CBSL,Ces2c,FASN,G6PC,GPAM,Hamp/Hamp2,HGF,LDLR,P4HA1,PCK1,PIGR,PKLR,TERT,USF2
**SP1**	1.422	1.81E-05	ABCA1,AKAP12,APOA1,APOE,ASS1,ATP2A2,BACE1,BMP7,CASP3,CBS/CBSL,CCND1,Ces2c,CREM,CSNK2A1,CYP17A1,CYP2B6,Cyp2d9 (includes others),CYP7B1,DHCR24,DIAPH3,DMD,EGFR,EGR1,ENTPD5,ESR1,F3,FAS,FASN,FGFR4,FOXM1,GSS,HADHB,HDC,HGF,HIF1A,HMGCR,HSD17B7,HSD3B1,Hspa1b,HSPA5,IL1B,IL4R,LAMA1,LCAT,LDLR,MAT2B,MAZ,NOX4,OGG1,PCK1,PCYT1A,PDGFA,PDK1,PPARD,PRKCA,PRNP,PROM1,RBL1,RRAS,SCARB1,SLC11A2,SLC22A4,SNRPN,STAT1,TERT,TFPI2,TGFBR2,TIMP3,TNFSF10,TRIB1,TYMS,UGCG,UGDH,UGT1A10 (includes others)
**Insulin**	1.414	1.93E-10	ABCA1,ACACA,ACADM,ACAT1,ACLY,AGT,APOA1,APOE,APPL1,BACE1,CCND1,CCNE1,CD36,CYP17A1,CYP7A1,EGFR,EGR1,ELOVL3,ELOVL6,FADS1,FADS2,FASN,FGB,FGFR4,FKBP2,G6PC,G6PD,GCLC,GSTP1,GUCY2C,HIF1A,HMGCR,HSD11B1,HSP90B1,HSPA5,HSPA8,IDH1,IGFALS,IGFBP2,IRF7,KLK3,LDHA,LGALS3BP,MCM6,ME1,MT-ND1,NDUFA1,NFIL3,NR1H3,NR4A1,NUCB2,OCLN,Orm1 (includes others),PCK1,PCNA,PCSK9,PDK1,PFKFB3,PLAAT3,PLIN3,PPARD,PPARGC1B,PRKCA,RBP4,RETSAT,RPS6,SCARB1,SCD,Scd2,SLC2A2,SLC2A4,SLC2A5,SLC38A3,SQLE,STAT1,STAT2,THRSP,TJP1,WNT4
**NR5A2**	1.414	1.01E-07	ABCG5,AFP,APOA1,C3,C5,CCND1,CELA1,CRP,CYP17A1,CYP7A1,CYP7B1,FASN,FGB,GATM,GPT2,HMGCR,HP,Hspa1b,IL1B,JUNB,KLK3,LDLR,NR0B2,RGS4,SCARB1,TNFRSF1B
**LIF**	1.408	7.21E-03	ACAA2,ACACA,AFP,AHSG,BMP7,CCND1,CCNE1,CRP,CYP17A1,EGR1,FGB,FGG,FOXF1,GPCPD1,HGF,HIF1A,IL1B,JUNB,LBP,LIMS1,MT-ATP6,MT-CO1,MT-ND1,MT-ND2,MT-ND5,MT-ND6,RGS4,SERPINA3
**MYC**	1.407	1.38E-04	ABCA1,ABCD1,ACACA,ACAT1,ACSL4,AFP,AKAP12,ANKRD17,ANXA5,ASS1,AURKB,Cald1,CASP3,CAST,CCND1,CCNE1,CD19,CHEK1,CHKA,CLU,COL15A1,CYFIP2,DDIT3,DDX3X,DEK,DNTT,EGR1,ERBB2,F2,F3,FABP5,FADS2,FAS,FASN,FBLN5,FCGRT,FOXF1,FOXM1,G6PC,G6PD,GADD45A,GCLC,GFER,GLUL,GM2A,GPC1,H3-3A/H3-3B,HIF1A,HLA-A,Hmgn2 (includes others),HRAS,Hspa1b,HSPB1,HSPH1,ID3,IDH1,IREB2,IRF7,IRF9,ISG20,KAT2A,LDHA,LIMS1,LOC102724788/PRODH,MCM5,MCM6,MGST3,MOGS,MTHFR,MYD88,MYLPF,NFAT5,OAS1,PCK1,PCNA,PDK1,PEG3,PFKFB1,PFKFB3,PHF21A,PKLR,PLP1,PPARD,PPL,PRDX3,PRKCA,PROM1,RAD51,RBBP4,ROCK1,RPL10,RPL23,RPS6,RRM2,RRP1B,SAE1,SCARB1,Scd2,SERPINA1,SLC22A4,SLC2A2,SMAD1,SNRPN,STAT1,STMN1,SUZ12,TAGLN2,TERT,TFRC,TGFBR2,TKT,TLE4,TMEM97,TNFRSF19,TNFSF10,TNS3,TYMS,UGT1A1,UGT1A3,UGT1A6,UNC119,VHL,WRN,XPO1,ZFP36
**RXRA**	1.399	3.67E-09	ABCA1,ABCC2,ACACA,ACACB,ACADM,ACSL3,ACSL5,APOA1,APOA2,APOA5,APOE,C1QA,C3,Ccl9,CCND1,CD36,CDO1,CLMN,CTSH,CYP2B6,CYP7A1,DR1,EHHADH,FABP5,FADS2,FASN,FGG,GAS6,GCLC,GPD1,GPX1,HADHB,HCK,HIF1A,HMGCS2,IL1B,MID1IP1,NR0B2,NR1D1,NR1H3,Orm1 (includes others),PCK1,PDGFA,PKLR,PLTP,PPARD,RXRG,SCD,SLC16A2,SLC2A2,SLC2A4,SLC51B,THRSP,UGT1A10 (includes others),WEE1
**EGR2**	1.397	8.42E-03	ASS1,BHLHE40,CAST,CHKA,DHCR7,EGR1,ERBB2,GTF2A1,HMGCR,HSPA5,ID3,IL1B,MR1,ROBO1,S100A1,SCD,SMAD1,SQLE,STAT5A,TFRC,TNFSF10,ZNF519
**F7**	1.396	4.03E-02	CASP7,CCND1,EGR1,F3,GADD45A,IL1B,ZFP36
**NFIL3**	1.387	2.39E-02	ACACA,FAS,FASN,GADD45A,SCD,TNFSF10
**NCOA6**	1.342	9.54E-04	ABCG5,CYP7A1,FASN,HSPA5,NR1I3
**26s Proteasome**	1.336	3.18E-02	ARNT,ATF5,BCL3,CTSF,DDIT3,EGFR,ESR1,GAS5,GCH1,HSD3B1,JUNB,POLA1,PRKCA,PRNP,SNAI1,STAT1,STAT5A,TNFSF10,TOP2A
**ROCK**	1.294	2.23E-02	CCND1,DMD,ETS1,F3,MAFK,MYLPF,NOP16,PPP1R12A,TJP1
**TFE3**	1.265	2.78E-04	CCNE1,ELOVL3,FASN,KLF2,MT-ATP6,MT-CO1,MT-ND1,MT-ND2,MT-ND5,PPARGC1B,SCD,THRSP
**SERPINA1**	1.264	2.66E-02	CD36,CYP7A1,DDIT3,HSP90B1,HSPA5,LDLR,PDIA3,SCARB1
**VCAN**	1.258	9.69E-03	ADAMTS9,APOE,ASS1,C1R,C3,CASP3,CLU,EGFR,F3,FABP5,FAS,FBLN5,FST,GUCY1B1,IL1B,ITGA5,LBP,OGN,ROCK1,SERPING1,STAT1,WNT4
**NCOA2**	1.253	1.07E-07	ADH4,CCND1,CYP17A1,CYP2B6,CYP7A1,EGR1,ELOVL6,FASN,FGB,FST,G6PC,GLO1,GPD2,HMGCR,HMGCS1,IL1B,KLK3,LDLR,LIPC,LIPG,NR1D1,PCK1,PGM3,PLTP,PPP1R3C,SLC2A2,TSC22D3
**STAT3**	1.247	8.60E-06	AGT,AHSG,BCL3,C5,CASP3,CASP7,CCND1,CCNE1,CD74,CEP70,CFB,CHEK1,CRP,DDIT3,DPP4,EGR1,ESR1,F3,FAS,FASN,FGA,FGB,FGG,FST,G6PC,GADD45A,GBP5,HGF,HIF1A,HLA-A,HLA-DMA,HLA-DQA1,HLA-DRB5,HP,IGFBP5,Igtp,IL1B,IL4R,IRF7,IRF9,Irgm1,ISG20,JUNB,KLK3,LBP,LCAT,LDHA,LGR4,MGAT5,MT-ATP6,MT-CO1,MT-ND1,MT-ND2,MT-ND5,MT-ND6,MYD88,OAS1,PCK1,PCSK9,PDIA4,PDK1,PROM1,PSMB9,RRAS2,SERPINA1,SERPINA3,SERPINB9,SLFN13,Slfn2,SNAI1,STAT1,STAT2,TERT,TFPI2,TLR3,TNFRSF1B,TNFSF10,ZFP36
**LPIN1**	1.237	6.51E-05	ACACA,ACADM,ACADVL,APOE,FASN,FDPS,HMGCR,IL1B,LPIN2,SCD
**INS**	1.233	1.89E-09	ACACA,ACADM,ACADVL,ACLY,AGT,APOA1,CBS/CBSL,CCND1,CD36,CRP,CYP17A1,DDIT3,EGR1,ERBB3,F3,FAS,FASN,G6PC,HGF,HIF1A,HMGCR,HSPA5,IGFBP2,IGFBP5,IGKC,JUNB,NOX4,NR1H3,NR4A1,PCK1,RDH16,SCD,SLC2A2,SLC2A4,SMPD2,SNAI1,UGCG
**IGFBP2**	1.215	4.59E-02	CASP3,CCND1,EGFR,ELOVL6,ESCO2,ESR1,IGFBP2,ITGA5
**PTP4A1**	1.213	5.81E-03	ACLY,ANXA5,CDPF1,COL15A1,CSNK2A1,DNAJB1,FASN,GSTP1,HIF1A,HRAS,PCNA,PPP1R12A,RAN,RCN2,ROCK1,STMN1,TAGLN2,TKT
**GPER1**	1.194	1.99E-02	BHLHE40,CCND1,DDIT3,EGR1,ESR1,FASN,HSPA5,RND1,ZFP36
**CREB1**	1.190	1.89E-04	ABCA1,Abca8a,APOE,ATF5,BCAP29,BHLHE40,BTG2,CALN1,CAMK1D,CCND1,CIART,CLMP,CPEB1,CREM,CRIM1,CYFIP2,DXO,EGR1,FASN,FRAT1,G6PC,GABPA,GADD45A,GDA,HECA,HIF1A,HLA-A,HLA-DQB1,HMGCR,HMGCS1,HSD11B1,HSPA5,IL1B,IRF7,JUNB,LDLR,LSM11,LSS,MCM5,MCM6,MKNK2,MRPL49,MTSS1,NARF,NECTIN3,NEURL1B,NFIL3,NR4A1,PCK1,PCNA,PLXNA2,RASGEF1B,RBP4,ROBO1,RRAS2,SCD,SLC2A4,SMPD3,SPTY2D1,SRPK2,SRXN1,SSR4,STMN1,SYNJ2,TAGLN2,TFRC,TIPARP,TP53INP2,ZFP36,Zfp948
**PLAGL1**	1.154	2.64E-02	LDHA,LOC102724788/PRODH,SLC2A4,SORD
**MECP2**	1.147	1.36E-02	APOE,DOCK8,DPP4,EGR1,ESR1,GABBR2,GSTO1,GSTP1,HSPA5,JUNB,Meg3,NR4A1,PLAAT3,PLP1,PROM1,TFPI2,UBE3A
**ASXL2**	1.134	6.39E-03	G0S2,GPCPD1,GPD2,HSD11B1,PPARGC1B,SCD,SLC2A4
**HOXC6**	1.134	4.52E-02	BMP7,CHRNA2,GAS2L3,RFX7,SENP8,SIPA1L1,SLC44A1
**CAMKK2**	1.131	1.57E-02	ACACA,CCND1,FASN,G6PC
**SIRT1**	1.130	2.36E-07	ABCA1,ABCC2,ACADM,ADAMTS5,ADAR,AFP,AGT,AURKB,BCLAF1,CCND1,CD36,CD74,CELA1,CYP2B6,DBP,EPDR1,ESR1,FAS,FASN,G6PC,GADD45A,GSTM1,GSTM3,HCK,HELZ2,HGF,HIF1A,HJURP,HLA-A,HLA-DQA1,HLA-DQB1,HLA-DRB5,HMGCR,Hsd3b4 (includes others),Ifi47,Igtp,IL1B,IRF7,IRGM,Irgm1,KLF2,LDLR,LGALS3BP,MPHOSPH9,MT-CO1,NAT1,NEDD4L,NR1D1,NTRK2,OAS1,PCK1,PHKB,PNPLA3,PSMB9,ROCK1,Scd2,SIRT3,Slfn2,STAT1,TERT,TIMP3,TRAF2,Wasl
**IGF2**	1.127	4.31E-08	APOA2,ARL4A,CCND1,CD36,CDC45,CRY1,DDIT3,DHCR7,EGR1,ELOVL6,GADD45A,GCH1,GPANK1,HELLS,HIF1A,IDH1,IGFBP2,IGFBP5,ITGA5,JUNB,KIFAP3,MCM2,MCM5,OGN,RAD51,RAD51AP1,RNF103,RNF13,RRM1,RRM2,SLC20A1,SLC2A4,UHRF1,XRN1,ZFAND5,Zfp35
**PTGER2**	1.127	3.74E-02	CENPF,CKAP2L,EGR1,HDC,IL1B,KIF18B,MASTL,MELK,NUSAP1,PABPN1,PBK,PILRB,TIMP3,TROAP
**TSC2**	1.126	7.39E-03	ACSS2,ATP6V1A,ATP6V1G1,CCND1,CP,DDIT3,EGR1,HIF1A,HMGCS2,HSPA5,HSPB1,IGFBP5,OGG1,PHLPP2,PRKCA,SCD,STAT1,THRSP,ZFP36
**IFN alpha/beta**	1.121	4.31E-03	DAXX,GAS7,HLA-A,Ifi27l2a/Ifi27l2b,IRF7,Irgm1,SERPINB9,SLPI,STAT1,STAT2,TLR3,TLR5,TNFSF10,TRIM21
**SOST**	1.118	1.12E-02	ACACA,ACADS,ACADVL,CD36,FASN,IL1B,NOX4
**MAPK9**	1.115	5.16E-04	ACACA,ACLY,BMP1,DMD,EGR1,FAS,FASN,GADD45A,GSTM1,HDC,HIF1A,IGFBP2,IL1B,IRF9,ISG20,JUNB,LGALS3BP,LMO7,NR1H3,NR3C2,PDIA3,RBL1,SCD,SERPINA1,TLR3,ZFP36
**HFE**	1.114	1.14E-03	DDIT3,Hamp/Hamp2,HSP90B1,HSPA5,SLC2A2,TFR2,TFRC
**HNF4α dimer**	1.114	2.06E-02	CYP7A1,PKLR,PON1,SLC2A2
**BAG1**	1.103	2.60E-03	CCND1,EGFR,FRK,GADD45A,Hspa1b,KLK3,NEDD4L
**HDAC4**	1.103	2.02E-02	CAMTA2,CYFIP2,DMD,ETNPPL,FST,G6PC,GADD45A,HIF1A,HMGCR,KLF2,LDHA,MMD2,NR4A1,Pcp4l1,PRKCA,RGS4,SLC2A4,TERT
**EGR1**	1.100	1.78E-04	APOA1,ATP2A2,BACE1,CASP3,CASP7,Ccl9,CCND1,CLU,CREM,CYP17A1,EGFR,EGR1,ENPEP,F3,FAS,GADD45A,HIF1A,HMGCR,IL1B,JUNB,LDLR,ME1,NR4A1,PDGFA,SNAI1,SQLE,TGFBR2,TNFSF10,UFSP1,WNT4
**EP300**	1.078	1.44E-02	ACACB,ACADM,AGT,AKAP12,BCLAF1,CCND1,CCNE1,CD19,CD36,CHKA,CLASP2,CRIM1,CYP7A1,DHRS3,EGR1,ERBB2,FASN,FRMD4B,G6PC,GM2A,HDAC11,HMGCR,HMGCS2,HSD11B1,ID3,KIF1B,KLK3,LAMA3,LDHA,LGALS3BP,MCM3,MFHAS1,NR4A1,PCK1,PCYT1A,PDGFA,PITPNM1,PKLR,RAD51,RMND5A,RPS6KA1,SLC43A1,SMPD3,TERT,TGFBR2,TIPARP,TLE2,TM2D2
**CLPP**	1.069	4.73E-04	ACADM,ACADS,ACADVL,CS,DLAT,ECH1,ECI1,ECI2,ETFDH,HADHB,PRDX3,SAMM50,SLC25A20,TKT
**CX3CR1**	1.067	2.03E-02	ANXA5,CD14,CD36,IL1B,MYD88,SLC2A2
**PIAS3**	1.067	2.06E-02	FGG,HP,SERPINA3,SNAI1
**MUC1**	1.033	1.12E-02	BAD,CCND1,CHEK1,FASN,GCLC,HGF,HIF1A,PDGFA,SNAI1,STAT1,TERT
**EIF4EBP1**	1.019	4.27E-02	CCND1,CYP2A6 (includes others),EGR1,IRF7,NFIL3,PHLPP2
**CEBPA**	1.014	2.75E-05	ABCA3,ACACA,ACLY,ADH7,AGPAT2,AGT,AKAP12,ASS1,BIK,BTG1,BTG2,C3,Ccl9,CCND1,CD14,CD19,CPS1,CYP2A6 (includes others),CYP2B6,CYP7A1,DHCR7,DYNLT3,EXTL2,FASN,FOXM1,G0S2,G6PC,GABPA,GADD45A,GCH1,GSTP1,Hamp/Hamp2,HGF,HMGCR,HP,HSD11B1,HSPA5,JUNB,MT-CO1,Mup1 (includes others),NFIL3,Orm1 (includes others),PCK1,PCNA,PGD,PPARD,PPL,PPP1R3C,PUM1,QKI,RBL1,SCAP,SCD,SERPINF1,SFTPD,SLC2A4,TFR2,TNFRSF19,TNFSF10,TRIB1,TSC22D3
**LEPR**	1.012	3.74E-05	ABCG5,ACACA,ANXA5,APOA1,APOA2,APOE,ATP2A2,BTG1,CYP2E1,EMSY,FASN,GPAM,GTF2A2,HIF1A,HMGCR,LDLR,LIPC,Mup1 (includes others),NR3C2,PLP1,PPARD,RNASET2,SCARB1,SLC2A2,SLC2A4,STAT2,TGFBR2,TIMP3,TP53INP1,TSC22D3
**CCNC**	1.011	4.18E-03	ACACA,CD36,FAS,FASN,SCD,SLC2A4,TYMS
**CREM**	1.007	5.00E-03	ABCA1,APOE,ATP2A2,BHLHE40,BTG2,CCND1,CIART,CREM,EGR1,GPD2,HMGCR,HMGCS1,JUNB,LDLR,LSS,MKNK2,NFIL3,NR4A1,PDP2,SPTY2D1,TIPARP,Zfp948
**AGTR1**	1.000	4.13E-03	AGT,CASP3,CCND1,CP,EGFR,GSTA5,Gstm6,GSTP1,HGF
**Mmp**	1.000	1.57E-02	FAS,OCLN,PLG,TJP1
**Ap2**	1.000	2.11E-02	AFP,BACE1,C3,ERBB2,ERBB3,PCK1,TERT
**ROR1**	0.990	1.12E-02	AMOTL2,GAS6,HEXB,SNAI1,STMN1,TJP1,TOP2A
**IL5**	0.976	1.81E-04	ACAA2,ASS1,ATP1B1,BCL3,CCNE1,CRELD2,CSF2RB,EGR1,FAS,GADD45A,GCAT,GPAM,GSTT2/GSTT2B,HIF1A,HMGCR,HMMR,HSP90B1,HSPA5,HSPH1,IL13RA1,IL4R,IRF7,LMNB1,LMO7,MANF,MAPK6,NFE2,NFIL3,P4HA1,PDE4B,PDIA6,PDK1,PDLIM1,PPIF,QSOX1,RRAS2,Scd2,SDF2L1,SFPQ,SNTB1,TMEM97
**MAPK1**	0.973	9.61E-03	ADAR,APOE,ATP1B1,CCND1,CCNF,CFB,DXO,EGR1,ERP29,ESR1,FAM20A,GBP5,Gstt3,HDC,HLA-DMA,IL1B,IRF7,IRF9,ISG20,JUNB,LGALS3BP,LIG1,MAP3K20,MYRF,NR4A1,NT5E,OAS1,OGFR,OSBPL3,PSMB9,QKI,SAMHD1,SHCBP1,SNAI1,STAT1,STAT2,TJP1,TLR3,TNFRSF1B,TNFSF10,TRIM21
**RHO**	0.956	2.31E-02	ACADM,C1QA,CASP3,CASP7,CD74,DDIT3,GNAT1,NID1,NT5E,SERPING1
**TWIST1**	0.949	1.11E-02	C3,CASP3,CBX2,CELSR1,CLU,ESR1,ETS1,FAS,FBXL14,GPC1,IGFBP5,IL1B,ITGA8,LRG1,MGST3,NT5E,OSGIN1,PROM1,PXDN,SNAI1,TGFBR2,TIMP3
**REL**	0.933	7.28E-03	ACSL4,ACSS2,ANLN,BCL3,CCND1,CDS2,CMPK1,CREM,DUT,ESR1,F3,FADS2,FAS,FRMD4B,GPT2,H2AJ,HLA-A,MLLT3,NFE2,NR4A1,PCBP4,PLAAT3,RAB40C,RAP2A,RBL1,SNAI1,Snord104,TNFSF10,TWNK,ZHX3
**STK11**	0.933	2.16E-02	ASS1,BCL6B,CCND1,CPS1,CS,CYP2B6,DUT,FGG,GADD45A,HIF1A,IGFBP5,IL33,ITGB3BP,JUNB,LDHA,MAFK,NDUFB11,NPEPL1,NR1D1,NT5E,PHC2,PRIM2,RND2,RPIA,RPS6,RRM1,RRM2,SNAI1,TKT,TNFSF10,TOP2A,TUBE1,TYMS

Table S5 List of Upstream Regulators signfficantly enriched and differentially activated/inhibited in the CCl4 model (KO vs WT).

Upstream Regulator	Z-Score	p-value	Target Molecules in Dataset
**MTORC1**	0.921	2.17E-03	CCND1,CYP17A1,FASN,FDPS,HMGCR,LDHA,NFIL3,NOX4,PLP1,SCD,SLC2A4
**Mek**	0.915	2.36E-03	ACAT1,APOE,CCND1,CD36,CDCP1,CLDN2,CLU,EGR1,ERBB2,ERBB3,ESR1,ETS1,FASN,GDA,HIF1A,HSPB1,Ifi47,IL1B,JUNB,MANSC1,NOL3,NOX4,NR1H3,NR4A1,PUS7,RETSAT,ROCK1,RPL23,SLC20A1,TOP2A,UNG
**ESR2**	0.896	1.84E-03	ABCA1,ABLIM1,ADAMTS5,ADH7,AGT,AKAP12,APOE,ARL4A,ATF5,C3,CBFA2T3,CBS/CBSL,CCND1,CLU,CRIM1,CYP17A1,CYP2A6 (includes others),DHCR7,EGFR,EGR1,ERBB2,ESR1,FASN,FOXM1,FST,GABBR2,GCLC,GM2A,GSTA5,GSTP1,HRAS,HSD17B7,IL1B,JUNB,KLK3,KRT8,LTBP1,MIOX,MPP7,OCLN,PCYOX1,PDGFA,PGRMC2,PPP1R12A,PPP1R3C,PROM1,RBP4,ROCK1,SCARB1,SCUBE1,SMC2,SORD,SRXN1,SYTL4,TERT,TGFBR2,THRSP,TMIE,TNS3,TRIB1
**MUC4**	0.896	2.70E-03	CCND1,EGFR,ERBB2,ERBB3,SNAI1,VTN
**JINK1/2**	0.896	7.63E-03	CYP7A1,FAS,FASN,PLG,SCD,SLC2A4
**CXCR4**	0.895	4.55E-02	C5,CCND1,DPP4,EGFR,EGR1,F3,JUNB,RRAS,TNFSF10
**PTGER4**	0.894	3.94E-03	APOA5,CP,CREB3L3,DAXX,EGR1,Fnbp1l,HGF,Ifi47,Igtp,IL1B,IRF7,IRGM,Irgm1,ISG20,MASTL,SLFN13,TNFSF10,TOR3A,TP53INP1,TRIM21,YPEL3,ZDHHC14
**MTOR**	0.875	7.43E-05	ACACA,ACACB,ACADM,ACLY,ARNT,ATP2A2,CASP3,CCND1,CCNE1,CHKA,CS,CYP17A1,DDIT3,DMD,EGFR,FABP5,FASN,FDPS,FGB,FST,HADHB,Hamp/Hamp2,HIF1A,HMGCS2,HSD11B1,HSP90B1,HSPB1,IL1B,IL4R,KLK3,LDHA,MT-CO1,Mug1/Mug2,NEURL1B,NOX4,OAS1,PCNA,PDE4A,PHLPP2,PLP1,PPARD,PPARGC1B,RPL23,SERPINA1,SGCB,SLC2A4,STAT1,TLR5,TNFSF10
**PLAU**	0.859	7.65E-04	CCND1,EGFR,F3,HGF,HLA-DMA,HSPA5,IGFBP2,IL1B,OAS1,PLG,PON1,SLFN13,SLPI,SNAI1
**PKM**	0.845	3.41E-02	ACLY,CCND1,EGR1,IL1B,LDHA,LSS,MT-ATP6,MT-CO1,PDK1,TP53RK
**PIK3CA**	0.816	4.08E-02	BAD,CCND1,CD14,FASN,FOXM1,GSS,HIF1A,PDK1,SNAI1,TNFSF10
**TGFA**	0.800	2.53E-02	CCND1,DDX3X,EGFR,ESR1,GSTP1,HGF,JUNB,PLAGL1,PRNP,SERPINA1,SLPI,TLR5
**PLIN5**	0.788	1.12E-03	ACAA2,ACADM,ACADVL,GCLC,GSS,GSTA5,MAFK,PPARGC1B
**RELA**	0.786	3.30E-03	ADAMTS5,AFP,AGT,APOE,BACE1,BCL3,BHLHE40,BTG2,C3,CASP3,CBS/CBSL,CCND1,CD14,CERT1,CFB,CRP,CYP17A1,CYP2B6,DDIT3,EGR1,ERBB2,F3,FAS,FCGRT,GCH1,Gstm3,HIF1A,HSP90B1,IGFBP2,IGKC,IL1B,IRF7,JUNB,KRT8,L3MBTL2,NR1I3,NR4A1,ORAI1,P2RY2,PCK1,PDE4B,PSMB9,SLC2A4,SLC2A5,SMOC2,SNAI1,STAT5A,TERT,TFPI2,UGT1A1,VHL
**FGF21**	0.785	5.03E-05	ACACA,ACADM,ACLY,BDH1,EGR1,FASN,G6PC,HMGCL,HMGCS2,LDLR,ME1,PCK1,PPARGC1B
**JAK2**	0.779	1.50E-02	AKAP12,BLNK,BMP7,Ccl9,CCND1,CD19,CD36,DHX40,EGR1,ESR1,GCNT2,IL1B,MYL12A,NR4A1,PCBP4,PHLDA3,PSMB9,STAT1,THPO,TP53INP1
**TP63**	0.767	1.72E-03	ADH7,AKT1S1,BAD,BCL3,BHLHE40,BLM,BMP7,CASP3,CAST,CAVIN2,CCND1,CCNE1,CYP2A6 (includes others),EGFR,EI24,F3,FAS,FASN,FDPS,FST,G6PD,GADD45A,HRAS,HSPA8,ID3,IGFBP2,IL1B,JUNB,KLK3,MDC1,MPZL2,NT5E,P2RY2,PCNA,PHC2,PKLR,PPP1R13L,PRNP,RAD51,RAP2A,RRM2,SERPINF1,SNAI1,STMN1,TERT,TGFBR2,TIMP3,TIPIN,TNFSF10,TOP2A,TRAF2,UGT1A1,UGT1A10 (includes others),UHRF1,WEE1,WNT4
**PCK1**	0.765	7.04E-05	ACADM,ACADVL,EHHADH,G6PC,IL1B,PCK1,SLC2A4
**SMAD4**	0.765	4.28E-02	AFP,APOA1,APOA2,BMP7,CCND1,CCNE1,CYP17A1,DAXX,EGFR,ERBB2,FST,GADD45A,HP,HSD3B1,IL1B,LOC102724788/PRODH,LPAR1,NPR2,PGRMC2,SAE1,SCD,SLC23A2,SNAI1,SSTR2,TIMP3,UNC119,ZFP36
**mGluR**	0.762	8.23E-03	EGFR,ERBB2,NTRK2,OPHN1
**SQSTM1**	0.761	4.15E-02	CYP2A6 (includes others),CYP2B6,GCLC,IL1B,MAP1LC3A,RTN4,UGDH,USP12
**MAP2K1**	0.748	1.08E-02	ALDH1L1,APOE,ASGR2,BAD,CAPN3,CCND1,CLDN2,CRIM1,CRP,CYP17A1,DPP4,F3,FASN,HDC,HIF1A,HSPA5,ITGA5,LDLR,PCNA,PLP1,RRM2,SCARB1,SERPINA1,TFPI2,TNFSF10
**GH1**	0.747	4.86E-03	CD19,CLU,DDIT3,EGR1,ERBB3,FASN,GADD45A,Hsd3b4 (includes others),ID3,IGFALS,IL4R,JUNB,LDLR,RAD23A,Slco1a1,SNAI1,TKT,ZFP36
**RGCC**	0.739	2.06E-02	FASN,IL1B,SCD,SNAI1
**TGFB1**	0.733	1.13E-06	ABCA1,ABCC2,ABCD1,ACAA2,ACLY,ACP3,Acp5,ACSL3,ADCY9,AFP,Akr1c19,AMOTL2,APOE,AQP8,ARF4,ARL4A,ASGR2,ASS1,ATP13A3,BACE1,BAD,BCL3,BDH1,BHLHE40,BMP1,BMP7,BTG1,C1QA,C1R,C3,C5,C730027H18Rik,CASP3,CAVIN2,CBFA2T3,CCND1,CCNE1,CD14,CD36,CENPF,Ces2c,CFB,CFI,CHD4,CLK2,CLU,CREB3,CRP,CST3,CTSH,CXADR,CYP17A1,CYP7A1,DAXX,DBP,DISP2,DSC2,DYNLL1,EGR1,ETS1,F2,F3,FABP5,FAS,FASN,FBLN5,FCGR3A/FCGR3B,FETUB,FGA,FGB,FGG,G6PC,GABBR1,GADD45A,GAS7,GATM,GCLC,GPR108,GPR146,GSDME,GSTA5,GUCY2C,Hamp/Hamp2,HAT1,HEXB,HGF,HIF1A,HLA-DMA,HLA-DQA1,HLA-DQB1,HLA-DRB5,HNMT,HSD17B6,HSPA5,HSPB1,HSPG2,ID3,IGFBP2,IGFBP5,IL1B,IL33,IL4R,ITGA5,ITIH3,ITIH5,JUNB,KLF2,KLK3,KRT8,LBR,LCAT,LDHA,LDLR,LIMS1,LIN37,LOC102724788/PRODH,LPAR1,LPCAT3,LRBA,LTBP1,MAPK6,MARCHF7,MBNL2,MCM2,MGAT5,MPHOSPH9,MPP6,MPV17L,MS4A8,MYD88,MYL12A,MYLPF,NAA80,NAB1,NCAPG,NDC80,NFAT5,NOX4,NR1H3,NR4A1,NT5E,NUAK1,NUCB2,OCLN,P2RY1,P4HA1,PCNA,PDGFA,PDZK1IP1,PGRMC2,PITPNM1,PLAGL1,PPARD,PPP1R3C,PRKCA,PROM1,QKI,RAD51,RAD51AP1,RBL1,RDH11,RFC4,RND1,RPA2,SAE1,SAMHD1,SCARB1,SCD,SELENBP1,SERPINA1,SERPINA3,SERPINF1,SLC20A1,SLC23A2,SLC51B,SMC2,SMOC2,SNAI1,SSTR2,STAT1,STAT5A,STK16,TERT,TFRC,TGFB1I1,TGFBR2,THPO,TIMP3,TJP1,TLE4,TOP2A,TSC22D3,TYMP,TYMS,UBE3A,UNC119,WNT4,XRCC4,ZFP36,ZNF354A
**KEAP1**	0.715	3.52E-03	Acp5,GCLC,GSTP1,HIF1A,IL1B,MAP1LC3A,TOP2A,UGT1A1
**EPO**	0.706	6.36E-03	ATOH8,BCL3,BLM,BTG1,CA1,CASP3,CD36,DALRD3,DDIT3,DHCR24,EGR1,FABP5,FAS,G6PC,GLUL,Hamp/Hamp2,Ifi27l2a/Ifi27l2b,JUNB,LDLR,MID1IP1,NFE2,NMNAT1,OSBPL1A,PCNA,PRKCA,RCC2,ROCK1,RPS6,SLC11A2,TFRC,TJP1,TNFSF10,TSC22D3,UGCG,ZFHX2,Zfp35
**PDX1**	0.677	8.34E-05	AKR7A2,ATF5,ATP2A2,CCND1,CDO1,CRELD2,CREM,CROT,Cyb5r3,ECH1,EGR1,ELAPOR1,ERO1B,F3,G6PC,GCH1,HSPA5,ID3,IDH1,IL1B,INTS5,JUNB,KRT8,MT-ND1,PAPSS2,PCNA,PDE4B,PDIA4,PDIA6,PDZK1IP1,SLC2A2
**Tgf beta**	0.657	1.46E-02	ABCA1,Acp5,ADGRE5,AKT1S1,ASGR2,BACH1,BHLHE40,CCND1,CCNE1,CLU,FGG,FST,GCLC,HSPG2,ID3,IGFBP5,IL1B,ITGA5,JUNB,LAMA3,LCAT,MAFK,NFAT5,NOX4,PDGFA,PPP1R13L,PROM1,QKI,SNAI1,TERT,TFRC,TIMP3,TNFSF10,VTN
**p70 S6k**	0.651	1.71E-02	CCND1,E2F4,FASN,NOX4,SCD,SNAI1
**EDN3**	0.651	2.16E-02	CCND1,CCNE1,EGR1,ITGA5,LAMA1
**Aldose Reductase**	0.640	2.16E-02	CCND1,CCNE1,G6PD,IL1B,PCNA
**FST**	0.638	2.36E-03	APOA2,BMP7,CD36,CPS1,ELOVL3,FST,HP,Mup1 (includes others),PLG,PPARGC1B,SERPINA1,SLC2A4,THRSP
**LXR ligand-LXR-Retinoic acid-RXRα**	0.628	7.98E-05	ABCA1,ABCG5,APOE,CYP7A1,PLTP,SCARB1
**TNFSF11**	0.620	2.10E-03	Acp5,Ccl9,CCND1,CCNE1,CD14,ESR1,ETS1,F3,FAS,FOXM1,FRRS1,GABPA,GCH1,GCLC,HCK,IL13RA1,IL1B,ITGA5,JUNB,MT-CO1,NFIL3,NR1H3,PDGFA,PHLDA3,RRAS,SENP2,SERPINB9,SIGMAR1,SLC11A2,SLC20A1,Slfn2,SRXN1,STAT1,STMN1,TNFRSF1B
**AMPK**	0.616	5.13E-04	AACS,ACACA,CS,CYP2B6,EGR1,FASN,G6PC,HIF1A,MT-ATP6,MT-CO1,MT-ND1,NOX4,NR0B2,ORAI1,PCK1,PFKFB3,PKLR,SLC2A4,SNAI1,STAT1,THRSP
**NFE2L2**	0.592	9.35E-09	ABCC2,Acp5,ACSL5,ADH7,AGT,AKR7A2,AOX1,C5,CD36,CELA1,CS,Cyp2a12/Cyp2a22,DDIT3,DHCR7,DNAJB11,DNAJB5,DNAJC3,DYNLL1,ENTPD5,ERP29,G6PD,GAS2,GCLC,GPX1,GSS,GSTA5,GSTM1,Gstm3,GSTM4,Gstm6,GSTO1,GSTP1,GSTT2/GSTT2B,HAX1,HSD3B1,HSP90B1,HYOU1,IDH1,IL1B,KLK3,MAP1LC3A,ME1,MGST3,MOGS,NAT8B,NR0B2,NR1I3,NUCB2,OSGIN1,PDIA3,PDIA4,PDIA6,PGD,RAN,S100A13,SCARB1,SERPINA3,SLC16A2,SLC35B1,SLC38A3,SRXN1,STIP1,THRSP,TKT,UGDH,UGT1A1,UGT1A10 (includes others),UGT1A6,Uox,USP14
**RETN**	0.592	4.15E-02	ACACA,APOA1,FABP5,FASN,G6PC,LDLR,SCD,SLC2A4
**CLDN7**	0.580	8.19E-03	ABLIM1,AGT,APOE,C3,CBX2,F3,HELZ2,LGR4,PKMYT1,PLAAT3,PRNP,PRSS8,RNASET2,RRAS2,SLC25A22,SLC35B1,TJP1,TSPAN12
**TWIST2**	0.579	8.72E-03	CCND1,FBLN5,IL1B,MGST3,OSGIN1,RNF43,SNAI1,TERT
**TFAP2A**	0.578	3.25E-02	ABCA1,APOE,BLCAP,EGFR,ERBB2,ESR1,FGFR4,G3BP2,GLO1,MCM5,PLAAT3,PPARD,TERT,XRN1
**RETNLB**	0.577	2.32E-02	BMP1,CASP3,Ccl9,IL13RA1,IL1B,IL4R,LAMA1,LMNB1,Mup1 (includes others),NR4A1,SMAD1,TERT
**BRD4**	0.564	2.64E-02	ABLIM1,ACSL5,AURKB,BCL3,BDH1,BHLHE40,CCND1,FADS1,GTF3C6,ITGA5,KAT2A,MCM3,MYD88,PDGFA,RAI14,RRM2,SORD,TOP2A
**SMARCA2**	0.555	5.92E-03	CCNE1,CD36,CP,CYP7A1,G6PC,IGFBP5,KLK3,PCK1,RBL1,RIC8B,RRM2,TYMS
**ZBTB20**	0.535	3.63E-05	ACSS2,ADH4,AFP,CYP2B6,Cyp2c54 (includes others),FASN,G6PC,GAS6,GCGR,GSTM4,IGFALS,IGFBP2,PCK1,UGDH
**FGF10**	0.529	1.03E-02	CTSH,FOXF1,HSD3B1,LDLR,LMO7,LSS,S100A13,SCD,SNAI1,TIMP3
**RORA**	0.492	1.95E-19	ABCA1,ABCG5,ACACB,ACSL4,AKR1D1,Aox3,APOA5,APOE,AQP8,ARNTL,ATP1B1,Cald1,CD36,CYP2A6 (includes others),CYP2B6,Cyp2c54 (includes others),CYP2E1,CYP7B1,DHCR24,ELOVL3,ELOVL6,FASN,G6PC,GPX1,GSTA5,GSTM2,GSTM4,GSTP1,Gstt3,HMGCR,HSD17B7,HSD3B1,Hsd3b4 (includes others),IL1B,LPIN2,LRTM1,MGST3,Mup1 (includes others),NR1D1,NR1D2,NR1I3,NTRK2,PCK1,PPP1R3C,RDH16,SCD,Scd2,SDR9C7,SELENBP1,SLC2A4,SLC30A10,Slco1a1,SULF2,Sult1a1,Sult1d1,Sult5a1,TLR3,UGGT2
**CD3**	0.492	5.94E-04	ACADS,ACP3,BTG1,BZW1,C1R,CASP3,CCND1,CCNE1,CD74,CENPF,CREM,CS,DHRS4,DPP4,E2F4,ETS1,FAS,FDPS,FKBP2,G6PD,GLDC,GTF2IRD1,GUCY1B1,HAT1,HAX1,HELLS,HIF1A,HLA-DQB1,HSP90B1,HSPA5,HSPA8,ID3,IGFBP5,IL1B,IL4R,IRF9,JUNB,LTBP1,MCM5,MT-CO1,NFAT5,NLRP1,NME4,NR3C2,NR4A1,OGN,ORAI1,ORAI3,P2RY2,PCNA,PCYOX1,PDCD5,PDE4A,PDE4B,PDIA4,PKLR,PKMYT1,RNF103,SDF2,SLC23A2,SLPI,SNRPN,STAT1,STMN1,TFPI2,TFRC,TMPO,TNFSF10,TOP2A,TPST1,TRAF2,VPS4B,WEE1,ZFP36
**IL13**	0.470	6.31E-03	ABCA1,ACADVL,Acp5,AVPI1,BCL3,C3,CASP3,CASP7,CD14,CD36,CFI,CHCHD7,CLDN2,CTSH,DHCR24,DNASE2,DYNLT3,EGFR,EGR1,F3,FADS1,FADS2,FAS,G0S2,G6PD,GAS6,GAS7,HDC,HSD11B1,HSD3B1,IFFO1,IL13RA1,IL1B,LTBP1,MTSS1,NCAPH,NFE2,NID1,PDGFA,PRSS8,QSOX1,SERPINA1,SERPINF1,SLPI,TFRC,TNFRSF1B
**SHH**	0.463	8.27E-03	ATF5,Ccl9,CCND1,CCNE1,CHEK1,EGFR,FOXF1,GPC1,HELLS,IGFBP5,MCM2,MCM3,MCM5,MCM6,NR4A1,OCLN,PCNA,RAD51,RPA2,SMAD1,TJP1,TSC22D3,TYMS,UNG
**Map3k7**	0.460	1.73E-02	AFP,BHLHE40,CASP3,FASN,IL1B,ISG20,MLLT3,PROM1,SCD,SNAI1,TLR3
**RARA**	0.459	1.15E-02	ABCA1,ABCC2,ABLIM1,ACACA,ACACB,APOA1,ATF5,C5,CCND1,CD14,CENPF,CENPM,CLMN,CP,CPSF4L,CYP7A1,DHRS3,DNAJC21,EGR1,FGFR4,HELLS,KLK3,LGALS3BP,NCAPG,NCAPH,NCOA5,NR2C1,OAS1,PBK,PCK1,PLXNA2,Rps27/Rps27rt,SAMHD1,SFPQ,SIPA1L1,SLC16A2,SLC2A2,STAT1,TERT,TLE4,ZNF367
**NONO**	0.447	3.60E-03	ACACA,CYP17A1,FASN,PDE4A,PDE4B,PDE7B
**JUNB**	0.437	9.38E-03	ACAT2,ACLY,BCL3,C3,C5,CCND1,CLU,FASN,FBLN5,JUNB,LPAR1,MYL12A,PTBP2,ROCK1,SCD,SFTPD,SNAI1,TIMP3,UGT1A3
**CSF1**	0.435	1.09E-04	Acp5,ADGRE5,APOE,BAD,CCND1,CCNE1,CYP17A1,DHCR24,DHCR7,EGR1,FAS,FCGR3A/FCGR3B,FDPS,HMGCR,HMGCS1,HSD17B7,HSP90B1,HSPA5,IL1B,IRF7,ITGA5,JUNB,LBR,LDHA,LSS,PCNA,PFKFB3,PMVK,SC5D,SQLE,STAT1,TLR5,TNFRSF1B,TRAF2
**P38 MAPK**	0.429	2.14E-02	ABCA1,ANXA5,AQP4,ASS1,CCND1,CCNE1,CCNF,CD36,CYP4F3,DDIT3,EGR1,ETS1,F3,FAS,FST,GCLC,HIF1A,HP,HSPA5,ID3,IGFBP5,IL1B,IRF7,JUNB,LAMA3,MYLPF,NFAT5,NR4A1,OCLN,PCK1,PCNA,RAD51,RGS4,SNAI1,STAT1,TERT,TIMP3,TNFSF10,TOP2A,TRIB1,ZFP36
**Gsk3**	0.399	1.39E-03	BACE1,CCND1,DDIT3,Dleu2,EGFR,ESR1,FASN,HIF1A,HMGCR,IL1B,LDLR,NR4A1,PMS2,SNAI1,STAT1
**HIF1A**	0.391	7.64E-05	AFP,AGT,AKAP12,APOE,AQP4,ATP2A2,BACE1,BHLHE40,CCND1,CD164,CD36,CDCP1,CHKA,CYP4F3,EGFR,ERBB2,ETS1,Hamp/Hamp2,HIF1A,HMGCL,HP,HSPA5,HSPB1,IGFBP2,IGFBP5,IL1B,ITGA5,KDM4B,KIAA1217,LDHA,MANF,MT-ND1,NOX4,NR4A1,NT5E,NTRK2,NUCKS1,P4HA1,PDGFA,PDK1,PFKFB3,PHLPP2,PRKCA,PROM1,QKI,R3HCC1L,RAB11FIP4,SESN2,SLC2A4,SNAI1,SSBP1,SUZ12,TERT,TFRC,TMEM19,TP53RK,TRIM21
**Mapk**	0.390	2.14E-02	APOA1,CCND1,DBP,EGR1,ESR1,F3,HGF,IL1B,JUNB,NR1I3,PIGR,PPARD,SNAI1,STAT1,UGT1A1
**THRSP**	0.372	5.96E-06	ACACA,ACLY,FASN,PCK1,PKLR,THRSP
**OSM**	0.367	1.90E-10	ABCA1,ACSL3,ACSL5,ADAMTS5,AFP,ARL4A,ATP13A3,BCL3,BHLHE40,BMP10,BTC,C1R,CCND1,CLPX,CRP,CRY1,CTSH,CXADR,CYP4F3,DHCR24,DHRS3,DNAJC3,DSC2,EGR1,EPHA1,ERBB3,F3,FASN,FETUB,FGA,FGB,GAS7,GCA,GLUL,GRHPR,Hamp/Hamp2,HGF,HIF1A,HLA-A,HMG20B,HP,HSD11B1,HSPA5,IL13RA1,IL1B,IL33,IL4R,IRF7,IRF9,ISG20,JUNB,LBP,LDLR,LRG1,MKNK2,MPZL2,MYD88,NEDD4L,NR1I3,NUAK1,OAS1,PCNA,PDGFA,PDZK1IP1,PFKFB3,PGGT1B,PIGR,PLLP,PSMB9,QKI,QSOX1,RAD23A,RAP2A,SERPINA1,SERPINA3,SERPING1,SLPI,SORD,STAT1,TDP2,TERT,TIMP3,TLR3,TOP2A,TUBGCP2,TYMP,UGT1A1,ZFP36
**AGT**	0.362	7.32E-07	ACAT2,ADAMTS5,AGT,ANK3,ARHGEF9,ASAH2,ATP1B1,BACH1,CALR,CASP3,CCND1,CCNE1,CD36,CP,CREM,CRP,CYP17A1,Cyp2c23,CYP2E1,EGFR,EGR1,ETS1,F3,FASN,GABBR1,GAS2,GCH1,GSS,GSTA5,Gstm6,GSTP1,HGF,HIF1A,HMGCR,HMGCS1,HSD3B1,Hspa1b,HSPB1,HSPG2,ID3,IDH1,IGFBP5,IL1B,ITGA5,LDLR,LSS,MAP1LC3A,NOX4,NR4A1,OCLN,PDGFA,PNKD,PPP1R3C,PTBP2,RBL1,RRAS,SCARB1,SPTSSA,TFRC,TGFBR2,TIMM9,TJP1,TLN2,TNFRSF1B,TUT4,UGCG,WNK4,ZFP36

Table S5 List of Upstream Regulators signficantly enriched and differentially activated/inhibited in the CCl4 model (KO vs WT).

Upstream Regulator	Z-Score	p-value	Target Molecules in Dataset
**LGR4**	0.359	4.13E-03	CD14,CYP2E1,ESR1,GLUL,IL1B,NR3C2,RDH16,SNAI1,WNT4
**KITLG**	0.351	2.89E-03	BIK,BMP1,BTG1,CASP3,CCNE1,CD14,DALRD3,DHCR24,EGR1,FABP5,FRK,GAS6,HCK,Ifi27l2a/Ifi27l2b,IL1B,LDLR,MID1IP1,NMNAT1,OGG1,ORAI1,OSBPL1A,PRKCA,RCC2,RPS6,TERT,TSC22D3,UGCG,USF2,WEE1,XRCC4,Zfp35
**T3-TR-RXR**	0.341	9.88E-06	ACACA,APOA5,CYP7A1,FASN,FGA,G6PC,HP,LDLR,ME1,PCK1,RCAN2,SCARB1,THRSP
**FZR1**	0.339	1.57E-02	CCND1,CCNE1,PFKFB3,VHL
**PEBP1**	0.302	3.20E-04	ACACA,ACLY,Aldh1a7,BACH1,ELOVL6,FASN,PCK1,PON1,RBP4,SCD,SNAI1
**ELANE**	0.298	2.43E-02	CD14,DDIT3,EGFR,HSPA5,IL1B,SERPINA1,SLPI
**PGR**	0.257	5.00E-05	ARNT,ATP1B1,BTG1,CCND1,CD36,DYNLL1,EGFR,EPHA1,ERBB2,ESR1,F3,G6PC,GAS6,GLUL,GSTM3,HIF1A,IGFBP5,LIG1,MTSS1,NOP16,NPC1,NQO2,OCEL1,OGG1,P2RY2,PAPSS2,PCNA,PDGFA,PDLIM1,PFKFB3,PHF21A,PPIF,RAP2C,SERPINA1,SLC31A2,STAT1,STAT5A,TIPARP,TSC22D3,UGCG,WNT4,ZNF354A
**CTNNB1**	0.248	1.73E-03	ACACA,ACAT1,ACAT2,ACLY,ADGRV1,AFP,AQP4,ARL4A,BACE1,BHLHE40,BMP1,BMP7,C6,CASP7,CCND1,CCNE1,CD36,Cd52,CELSR1,CENPM,CLDN2,CLU,COL4A5,CTSF,CYFIP2,CYP2A6 (includes others),Cyp2d9 (includes others),CYP2E1,CYP7B1,DDIT3,DIAPH3,EGFR,EGR1,ELOVL3,ERBB3,FAM126A,FAS,FASN,FST,GCH1,GLUL,GPAM,HMG20B,HMGCR,HMGCS1,HMGCS2,Hsd3b4 (includes others),ID3,IGFBP2,IGFBP5,IL1B,KLK3,ME1,MPZL2,Mup1 (includes others),NFAT5,NR4A1,OGG1,OGN,OSBPL1A,PCNA,PDE4B,PEG3,PROM1,RAD23A,RAI14,RBP4,SCAP,SCD,SERPINA1,SERPINA3,SLC25A46,Slco1a1,SNAI1,STAT5A,SUZ12,TERT,TIMP3,TJP1,TLE4,TNFRSF19,TNFSF10,UBQLN4,UGCG,WNT4,YPEL3,Zfp94,ZNF778
**IRF2**	0.240	6.09E-05	CCND1,CD14,CFB,CLDN2,FGA,FGB,FGG,IDH1,Ifi47,IL1B,IRF7,LBP,MAPK6,OAS1,PFKFB3,PSMB9,PTGR1,SLC51B,SRXN1,TLR3,TLR5,TMEM256,TNFSF10,TRIM21
**CCR5**	0.227	4.52E-02	CASP3,F3,FAS,IL1B,SERPINA1,SLPI,TNFRSF1B
**KLF15**	0.226	6.41E-05	ACADM,ACADS,ACADVL,ACAT1,CD36,EHHADH,FABP5,FASN,GPAM,HADHB,SCD,SLC25A20,SLC2A4
**ARRB2**	0.218	4.85E-02	ATP2A2,EGR1,F3,HIF1A,IL1B,JUNB
**ELAVL1**	0.215	1.84E-02	ABCA1,CCNE1,Gm4951,GSS,H2-K2/H2-Q9,HLA-A,HLA-DRB5,IRF9,Irgm1,LGALS3BP,NCAPG,NXF1,OAS1,PBK,PIGR,PROM1,RPS6,Slfn2,STAT1,TJP1,TOP2A,TP53INP1,TSC22D3,WEE1,ZFP36
**MAP4K4**	0.203	4.59E-05	ACAA2,ACACA,ACACB,ACADS,ACADVL,ACLY,ACSL5,DHRS4,DLAT,FASN,GLUL,GRHPR,HADHB,HMGCL,ITGA5,KYAT3,PAPSS2,PEX11A,PHYH,PNPLA3,SCD,SCP2,SLC2A4
**CSF1R**	0.186	4.52E-02	Acp5,CCND1,CCNE1,IL4R,JUNB,NR1H3,PCNA
**NR1D1**	0.167	2.27E-05	ACACA,ACLY,ARNTL,CRY1,EGR1,EHHADH,FADS2,FASN,G6PC,ME1,NR1D1,STAT1
**ADCYAP1**	0.165	3.43E-02	ACAT1,AZIN1,B4GALT3,BTG2,CCBE1,EGR1,FRRS1,FST,GREM2,Hmgn2 (includes others),ID3,KRT8,LAMA1,MCM2,MREG,NTRK2,P2RY2,PLAGL1,QKI,SCARB1,SELENOT,SERPINA3,SERPINF1,SMAD1,TFPT,TMPO,UGT1A6
**BMP6**	0.165	4.23E-02	ACADM,ADAMTS5,ARHGAP18,Cald1,CAVIN2,FRMD4B,Hamp/Hamp2,ID3,ITGA5,SLC20A1,SNAI1,SORD,TEF
**HOXA-AS2**	0.157	8.23E-03	DDIT3,EGFR,KLF2,TNFSF10
**AEBP1**	0.152	5.58E-03	ABCA1,APOE,CD36,NR1H3
**EIF2AK4**	0.151	3.73E-02	AQP4,CHAC1,DDIT3,EGR1,IL1B,PLP1
**BAP1**	0.150	1.53E-03	ASXL2,CDC14A,DDIT3,HSPA5,MCM3
**Growth hormone**	0.147	1.83E-09	ACACA,AGT,APOE,BMP7,C3,CCND1,CD36,CLU,CROT,CYB5B,Cyp2a12/Cyp2a22,CYP2E1,CYP7A1,DDIT3,EGFR,EGR1,FASN,FDPS,FGG,G6PC,G6PD,GCLC,GSTM1,GSTP1,HADHB,IGFALS,IGFBP2,IGFBP5,JUNB,LDLR,MT-ND5,NOX4,NR1D1,NR2F6,PCK1,PCNA,PFKFB3,PLAAT3,PON1,PRDX3,Pzp,SCARB1,STAT5A,TGFBR2,TKT,TNFSF10,UGDH
**BRCA1**	0.144	4.40E-04	ATP11C,BACH1,CCND1,CCNE1,CTSF,DDIT3,EGFR,EGR1,ESR1,FAS,FHIT,GADD45A,H3-3A/H3-3B,HSPB1,IRF7,KLK3,LAMA3,PCNA,PLTP,RAD51,RBL1,ROBO1,SFPQ,SNAI1,STAT1,STAT5A,TERT,WEE1
**NFYA**	0.140	8.66E-09	ACLY,BIK,CALR,CBS/CBSL,COL5A3,EGR1,FASN,G6PC,GADD45A,GPAM,GUCY1B1,HLA-DQB1,HSD17B7,HSPA5,JUNB,OGG1,PCK1,POLA1,RGS4,RRM2,SCD,Scd2,TGFBR2,THRSP,TOP2A
**ITGA5**	0.133	3.73E-02	APOE,CCND1,CHRD,ERBB2,IGFBP2,IL1B,JUNB,TGFBR2
**PPP3R1**	0.128	3.73E-02	ABCA3,ATP2A2,C5,PON1,SCD,Scd2
**PDPK1**	0.122	4.27E-02	CCND1,CST3,IL1B,KLF2,PDGFA,SNAI1
**NRIP1**	0.113	8.29E-04	ACAA2,ACACA,ACACB,APOA1,CCND1,CCNE1,FASN,HADHB,IL1B,NR2C1,SCD,SLC25A20,THRSP
**CXCL12**	0.099	8.52E-03	AFP,BAD,BCL3,BMP1,C5,CCND1,CD14,CD36,CYP2A6 (includes others),CYP2C18,DPP4,EGFR,EGR1,ERBB2,FAS,GAS2,IL1B,JUNB,MAFK,NR2F6,NR4A1,ROCK1,RRAS,SSBP1,TNFRSF1B,TNFSF10
**FXR ligand-FXR-Retinoic acid-RXRα**	0.070	7.63E-05	ABCC2,ABCG5,APOE,FASN,FETUB,LIPC,NR0B2,PLTP,SCARB1
**HOXA11**	0.064	3.30E-02	EGFR,ITGA8,KLF2,PDGFA
**INSR**	0.060	2.14E-07	ACAA2,ACADM,ACADS,ACADVL,ACSL4,ACSL5,ARL4A,C1QA,CCND1,CD36,CDC45,CRY1,CS,DEK,DHCR7,ECI1,EGR1,ETFDH,FASN,FDPS,GADD45A,GCH1,GPANK1,HADHB,HELLS,HMGCR,HMGCS1,IDH1,IL1B,ITGA5,JUNB,KIFAP3,LSS,MCM2,MCM5,Meg3,MT-CO1,NDUFA1,NR1H3,OGN,PDE4B,PKLR,PLAGL1,PPARGC1B,RAD51,RAD51AP1,RNF103,RNF13,RRM1,RRM2,SC5D,SCARB1,SCD,SCP2,SLC20A1,SLC25A20,SLC2A4,SQLE,UHRF1,XRN1,ZFAND5,Zfp35
**ADIPOQ**	0.060	9.38E-05	ACACA,ACACB,ACLY,ACSL5,BAD,BCL3,CCND1,CD36,CYP7A1,EGR1,F3,FASN,G6PC,GPD1,HMGCS2,IL4R,LDLR,NCF4,NOX4,NR1H3,PCK1,PCNA,PFKFB1,PPARD,PRDX3,ROCK1,SCD,SLC27A2,SLC2A4
**USF2**	0.049	3.37E-04	ABCA1,AGT,APOA2,APOA5,FASN,Hamp/Hamp2,HGF,KLK3,LGALS3BP,P4HA1,PIGR,PKLR,TERT,THRSP
**BMI1**	0.032	1.54E-03	BTG2,CCNE1,CDC14A,CYFIP2,DENND10,FAS,KIF1B,MCM3,NID1,P4HA1,RRM1,TERT,USF2
**LRPPRC**	0.000	1.60E-06	MT-ATP6,MT-CO1,MT-ND1,MT-ND2,MT-ND5,MT-ND6
**RBL2**	0.000	1.31E-02	AURKB,CCND1,CCNE1,CCNF,CHKA,E2F4,FAS,PCNA,PPM1G,PRIM2,RBL1,RRM2,STMN1,TERT,TYMS
**AKT3**	0.000	1.42E-02	AKT1S1,ERBB2,ERBB3,ETS1,MT-CO1,RPS6
**MDGA2**	0.000	1.57E-02	ATR,EI24,FAS,SESN2
**SP110**	0.000	2.99E-02	ANXA5,BCL3,CD74,CLU,DMD,ETS1,F3,IL4R,IRF9,MTHFR,NFIL3,OAS1,POLE,PRKCA,SERPINA1,STAT1,STAT5A,TAB3
**YY1**	-0.026	1.18E-10	ARID3A,AURKB,BIK,BLNK,BTG2,CALR,CASP7,CDCA3,CLU,COL15A1,DMD,DNTT,EEF1B2,EGFR,EGR1,ERBB2,FAS,FDPS,FOXM1,GFER,GTF2A1,HELLS,HMGCR,HSPA5,ID3,IGFBP5,IL1B,KLF2,LAMA1,LDLR,LMNB1,MCM5,MCM6,MYLPF,PCNA,PDGFA,PEG3,PLAGL1,POLA1,PSRC1,PTBP3,RAD51,RAD51AP1,RBBP4,RRM1,SCARB1,SFPQ,SLC23A2,SNRPN,TLN2,TOP2A,TP53INP1,UHRF1,UNG,USP14,WSB1
**PTK2**	-0.049	3.77E-02	CCND1,ITGA5,KRT8,LDLR,OCLN,P4HA1,SNAI1,TIMP3,TJP1
**NR2C2**	-0.068	4.34E-02	APOE,CCND1,CD36,DR1,THRSP
**PIAS1**	-0.072	4.52E-04	ACACA,CCND1,CYP17A1,FASN,GADD45A,KLF2,NR0B2,SCD,SLC51B,STAT1
**GNRH1**	-0.104	1.32E-02	CHRNA4,CREM,CYP17A1,EGR1,FST,HSD11B1,HSD3B1,IGFBP5,IL1B,JUNB
**NFKBIA**	-0.109	1.60E-06	ADAMTS5,ADGRE5,APOA1,AURKB,AZGP1,BCL3,BTG2,C3,Ccl9,CCND1,CDC45,CLU,CP,CREB3L3,CTSF,CYP7B1,DDIT3,DDX3X,ERBB2,F3,FAS,FCGRT,FGFR4,FOXM1,GADD45A,GAS5,GCH1,GM2A,HIF1A,HLA-A,HSPA5,HSPA8,IL1B,IL33,JUNB,KRT8,LAMA5,MR1,NID1,NINJ1,NLRP12,NR1I3,NR4A1,OGN,PCNA,PDGFA,PDLIM1,PEA15,PICALM,RABEP2,RGS4,ROCK1,RRM1,Rtp3,SLC16A2,Slfn2,TFRC,TGFB1I1,TIMP3,TMPO,TNFRSF1B,TNFSF10,TRAF2,UBE3A
**PER1**	-0.115	1.21E-04	ARNTL,CCND1,CCNE1,CRY1,DBP,KLK3,PER3,WNK4
**ARNT2**	-0.131	2.28E-02	AOX1,APOA5,APOE,AQP4,Cd52,EGFR,EGR1,FHIT,GLUL,GUCY2C,H2-K2/H2-Q9,HLA-A,ICMT,ITIH3,KLK3,LCAT,NDUFA1,NFE2,PEA15,PLP1,SELENBP1,TSC22D3,UGT1A6,USP2,ZDHHC14
**SIM1**	-0.131	3.10E-02	AOX1,APOA5,APOE,AQP4,Cd52,EGFR,EGR1,FHIT,GLUL,GUCY2C,H2-K2/H2-Q9,HLA-A,ICMT,ITIH3,KLK3,LCAT,NDUFA1,NFE2,PEA15,PLP1,SELENBP1,TSC22D3,UGT1A6,USP2,ZDHHC14
**PRNP**	-0.135	1.09E-02	APOE,CASP3,CCND1,DDIT3,EGR1,FST,HSP90B1,HSPA5,HSPA8,HSPB1,IL1B,PDIA3,PRNP
**estrogen receptor**	-0.137	4.28E-02	ABCA1,BMP7,C3,CCND1,COL4A5,CYP2B6,DSC2,EGFR,EGR1,ERBB2,ERBB3,ETS1,FGFR4,GSTA5,HRAS,KRT8,LDLR,NT5E,OCLN,PDGFA,SMAD1,SNAI1,TERT,TGFBR2,TIMP3,TJP3,TSC22D3
**CIP2A**	-0.149	3.73E-02	CFB,COL15A1,DSC2,ELAPOR1,GADD45A,LAMA1,LRP4,PDK1,SLC22A4,TUBA4A
**FOXO1**	-0.150	7.08E-07	ACACA,ACLY,ACSL5,ANLN,APOA5,BLNK,C1QA,CCND1,CCNF,CD36,CENPF,CS,CYP7A1,EGR1,ELOVL5,ELOVL6,ENPEP,FABP5,FAS,FASN,FDPS,FOXM1,FOXP4,FST,G6PC,GADD45A,GPAM,GPD1,GPD2,GRHPR,HIF1A,HMGCR,HSPA5,HUS1,HYOU1,IL1B,JUNB,KLF2,KLK3,LPIN2,MCM5,ME1,MRPL34,NCAPG,NPC1,NUSAP1,PBK,PCK1,PKLR,PPARGC1B,RPIA,SCARB1,SCD,SLC25A20,SLC25A22,SLC25A25,SLC25A30,SLC2A2,SLC2A4,SQLE,STAT2,TKT,TNFSF10,TRAF2,WNT4
**Nuclear factor 1**	-0.152	4.88E-04	CYP2A6 (includes others),DDIT3,GSS,GUCY1B1,HGF,HSD17B7,PKLR,SERPINA3,SLC2A4
**Complement**	-0.152	2.32E-03	BAD,C5,CLU,F3,PDGFA
**EBF1**	-0.158	3.59E-02	ACACB,Ccl9,CD19,ESR1,FASN,G6PC,IGKC,IRF7,MKNK2,MYD88,NFIL3,PPP1CB,PPP1R3C,SCD,SLC2A4,STAT1,TLR3
**NFATC2**	-0.172	2.28E-02	ABCA1,ACP3,CASP3,CCNE1,CCNF,DAXX,FAS,FOXM1,HELZ2,IL1B,IRF7,IRGM,ISG20,LDHA,NAA80,NOX4,NR4A1,PDCD5,SNAI1,STAT1,STAT2,STAT5A,TERT,TLE4,TLR3
**TLR3**	-0.183	2.91E-02	ABCA1,C3,CAST,CD300E,CFB,CLU,FAS,GCH1,Hamp/Hamp2,Ifi47,IL1B,IL33,IRF7,ISG20,JUNB,LIPG,LMNB1,LPIN2,MYD88,NR4A1,OAS1,PFKFB3,SERPINB9,SLPI,STAT1,TLR3,TNFRSF1B,TNFSF10,TSPAN33,UBE3A
**IKBKB**	-0.188	3.55E-05	ABCA1,Acp5,AFP,AURKB,C3,Cald1,Ccl9,CCND1,CLU,CP,CTSF,CYP7B1,EGR1,ETS1,F3,FAS,FASN,FOXM1,GADD45A,GCH1,GM2A,HGF,HIF1A,HLA-A,IGFBP2,IL1B,JUNB,KRT8,LMNB1,MYO1D,NID1,OGN,RABEP2,Rtp3,SLC16A2,Slfn2,SNAI1,TGFBR2,TIMP3,TLR3,TNFRSF1B,TRAF2,UACA
**RARB**	-0.194	4.05E-03	ACADM,APOA1,CASP7,CCNE1,CPSF4L,DHRS3,DLG1,EGFR,EGR1,ETS1,GADD45A,ID3,IL1B,KLK3,RPL15,Rps27/Rps27rt,SLC16A2,SSR4,STAT1,TJP1
**HDL**	-0.201	3.73E-02	DDIT3,HIF1A,HSPA5,IL1B,KLF2,LDLR,MYD88,PON1
**HNF1B**	-0.213	2.30E-03	ACADVL,AFP,BHLHE40,FGB,ID3,KIFAP3,MPZL2,OCLN,PCSK9,PDZK1IP1,PSTPIP2,SERPINA1,SGK2,SLC2A2,Slco1a1,SNAI1,Sult1d1,TM4SF4,UGT1A10 (includes others)
**EIF2AK3**	-0.214	1.09E-02	ARNTL,ATF5,BTG2,CCND1,DDIT3,DNAJC3,EGR1,ERO1B,GADD45A,GCH1,HSPA5,IL1B,MTHFR,NFIL3,SARS1,ZFP36
**COLQ**	-0.216	4.45E-02	ADAMTS5,BMP1,DMD,FBLN5,GPC1,HSPG2,LRP4,LTBP1,NID1,WNT4
**CLOCK**	-0.229	2.50E-08	ACSL4,AVPR1A,BHLHE40,CA1,CCND1,CTPS2,DBP,DDIT3,DHTKD1,DR1,EGFR,ELOVL6,FASN,GADD45A,GALNT11,HLA-DQB1,HMGCS2,Hspa1b,HSPH1,IGFBP5,JUNB,MID1IP1,MKNK2,NFIL3,NR0B2,NR1D1,NR1D2,NR2C1,PDP2,PER3,PICALM,PIGA,PPIF,PRDX3,QSER1,RAD23A,RAP2A,RCN2,RNF11,SCD,SLC20A1,Smim11,TEF,TMED3,TPST1,USP2,WEE1,WNK4
**TSH**	-0.229	3.41E-02	CCND1,CCNE1,EGR1,ERP29,GCH1,IGFBP5,JUNB,NOX4,PPARD,SLC2A4
**PRKAA1**	-0.238	1.40E-02	BTG1,CENPF,CLU,CYP2B6,DDIT3,FOXM1,G6PC,GPX1,HSPA5,ITIH5,LDHA,MT-CO1,NPEPL1,OCLN,OPHN1,PKLR,SIRT3,THRSP,TJP1,TNS3
**ERBB4**	-0.242	1.91E-02	BTG2,CCND1,DDIT3,EGFR,EGR1,GADD45A,GPC1,HIF1A,IGFBP5,NID1,SERPINA3,SLPI,TIMP3
**CREBBP**	-0.253	4.42E-02	AGT,AKAP12,BMP7,CCND1,CLASP2,CRIM1,CYP7A1,DHRS3,EGR1,EHHADH,FDPS,FRMD4B,G6PC,GM2A,HDAC11,HMGCS1,HMGCS2,HSD11B1,KIF1B,KLF2,KLK3,LDLR,LGALS3BP,MCM3,MFHAS1,NR4A1,PCK1,PCNA,PITPNM1,PKLR,PSMB9,RMND5A,RPS6KA1,SERPINA3,SLC43A1,SMPD3,THRSP,TIPARP,TLE2,TM2D2,TNFSF10
**GAB1**	-0.254	4.90E-02	CASP3,EGR1,HRAS,IL1B
**ANGPT2**	-0.260	2.41E-04	C1R,CALR,CASP3,CFB,DDIT3,EEF1B2,EGFR,EPHX2,ETS1,FAS,FKBP2,HIF1A,HMGCS1,HSPA5,IL1B,LARP4,NOX4,NR3C2,PDIA3,PDIA6,PLG,POLA1,RFC4,RPA2,RPS6,SERPING1,SNAI1,SRXN1,TGFBR2,TLR3,TOR1AIP2,VCPIP1
**IKBKG**	-0.266	1.24E-03	ATP2A2,C3,Ccl9,CLU,CP,CTSF,CYP7B1,GCH1,GM2A,HIF1A,HLA-A,IL1B,IRF7,JUNB,NID1,OGN,RABEP2,Rtp3,SLC16A2,Slfn2,TNFRSF1B,TNFSF10
**MAP2K1/2**	-0.267	8.85E-03	C3,CCND1,CYP7A1,DDIT3,EGR1,GCLC,HSPA5,HSPB1,IL1B,PCNA,PDGFA,RRM1,SCARB1
**SMAD3**	-0.269	6.87E-03	APOA1,APOA2,ARID3A,C3,CCND1,CCNE1,CRP,CYP7A1,DDIT3,EGR1,ESR1,FST,G6PC,Hamp/Hamp2,HP,HSP90B1,HSPA5,ID3,IL1B,JUNB,KLK3,LPAR1,Orm1 (includes others),PCNA,PPARD,SLC51B,SMAD1,SNAI1,SSTR2,TERT,TIMP3,ZFP36
**HDAC5**	-0.269	1.44E-02	ACADM,ETNPPL,G6PC,HMGCL,HMGCR,HMGCS2,KLF2,MMD2,NDUFA1,NR4A1,SCD,SLC2A4

Table S5 List of Upstream Regulators signficantly enriched and differentially activated/inhibited in the CCl4 model (KO vs WT).

Upstream Regulator	Z-Score	p-value	Target Molecules in Dataset
**S-nitroso-N-acetylpenicillamine**	-0.271	4.85E-02	CCNE1,DDIAS,DDIT3,FAS,HIF1A,LDLR
**ATR**	-0.277	1.41E-02	ATR,FAS,PCNA,RRM2,WRN
**ETV5**	-0.277	2.21E-02	CAVIN2,CCND1,FASN,FOXM1,ITGA5,LMO7,MID1IP1,QKI,SAMHD1,SCARB1,SCD,TIMP3,TJP1,TJP3
**Creb**	-0.301	1.57E-02	ANK3,ARHGEF9,ATP2A2,BACE1,CCND1,CD14,CLMN,CREM,CYP17A1,DDX28,EGR1,FAS,G6PC,GABBR1,GDA,GIMAP8,Gimap9,GSTM3,GSTM4,GTF2H3,HTT,IL1B,JUNB,LDLR,MCM3,MT-ND5,NR4A1,OSBPL3,PCK1,PCNA,PDE4B,PLIN3,POLA1,POLE,PRKCA,SSTR2,TCTEX1D2
**RAF1**	-0.305	2.21E-04	ACAT2,AKAP12,BTG1,CCND1,CD74,CRP,CTSH,DHRS3,EGR1,ERBB2,ESR1,FAS,HDC,HIF1A,HSPB1,ID3,IL1B,LAMA3,LDHA,LPGAT1,MGAT5,NINJ1,OCLN,PCK1,PPP1R10,RAN,RRM2,SELENBP1,SMAGP,SQLE,TERT,TFPI2,TUBA4A
**ESRRA**	-0.312	1.85E-02	ACADM,ACADVL,ATP2A2,CD36,CHKA,CYP17A1,DEK,ERBB2,ETFDH,HMGCR,HMGCS1,LDHA,NDUFA1,NDUFAF5,NR0B2,NR1D1,PPARGC1B,Scd2,SIRT3,SLC25A20,STARD3,TOP2A,TRA2B
**IL1B**	-0.336	1.11E-09	ABCC2,ACP3,ADAMTS5,APOE,AQP4,ASAH2,ASS1,ATF5,ATP2A2,AVPR1A,BACE1,BCL3,BTG2,C1R,C3,CASP3,Ccl9,CD14,CD74,CFB,CP,CREM,CRP,CSF2RB,CTSF,Cyb5r3,CYP2B6,CYP2E1,CYP7A1,CYTIP,DBP,DDIAS,DDIT3,DPP4,DR1,EGR1,ERBB2,ESR1,F3,FABP5,FAS,FGB,FGG,FST,G0S2,G6PD,GADD45A,GAS5,GAS6,GCH1,GCLC,GM2A,Hamp/Hamp2,HELZ2,HEXB,HGF,HIF1A,HLA-A,Hmgn2 (includes others),HP,HSD11B1,HSPA5,HSPB1,HSPG2,ID3,Ifi47,IGFALS,IGFBP5,IL1B,IL33,IRF7,ISG20,ITGA5,JUNB,LAMA3,LBP,LDHA,LDLR,MYD88,NFAT5,NFIL3,NR0B2,NR1D1,NR1H3,NR1I3,NR4A1,OCLN,PCNA,PDE4B,PDK1,PIGR,PPARGC1B,PSMB9,RAN,SAA2-SAA4,SCARB1,SCUBE1,SERPINA3,SERPINB9,SIRT3,SLC11A2,SLC20A1,SLC22A4,SLC25A25,SLC2A2,SLC2A4,Slco1a1,Slfn2,SSTR2,STAT1,STAT5A,SYVN1,TFPI2,TGFBR2,THRSP,TIMP3,TJP1,TLR3,TNFRSF1B,TNFSF10,TRAF2,TSC22D3,TYMP,UGCG,UGDH,UGT1A1,ZC3H12A,ZFP36
**IGF1R**	-0.341	1.22E-02	ACADM,ACADS,AQP4,BAD,CALU,CD36,CLU,DDIT3,EGR1,ESR1,FASN,HIF1A,HSPA5,IGFBP2,IGFBP5,IL1B,Meg3,MT-ATP6,MT-CO1,NDUFA1,NR4A1,PROM1,RPS26,Scd2,SCP2,SLC16A2,SLC25A20,SLC2A2,SNAI1,TIMP3,TJP1,TNFSF10,TYMS
**IL3**	-0.346	3.05E-03	ADGRE5,ATF5,BTG2,C1QA,CALR,CASP3,CCND1,CD14,Ces1b/Ces1c,CREM,CSNK1A1,DDIT3,ECH1,EGR1,FAS,FASN,FGFR4,GADD45A,GPX1,HGF,HLA-A,HSP90B1,HSPA5,HUS1,IL1B,IL33,JUNB,KIF1B,MCM5,PPM1G,RAN,RIDA,RPL10,RPS3,RPS6,SERPINA3,SSBP1,TERT,TNFSF10,USF2,YPEL3,ZFAND5
**GPS2**	-0.351	4.55E-02	ABCA1,BLNK,CCND1,CD14,CYP7A1,DHRS3,HP,IDH1,SLC44A1
**MDM2**	-0.359	3.07E-02	CCND1,ETS1,FCGRT,HIF1A,NDUFA1,NR0B2,RABAC1,RPL23,TERT,TNFSF10,TOP2A
**PPARA**	-0.365	1.76E-38	ABCA1,ACAA2,ACACA,ACADM,ACADS,ACADVL,ACAT1,ACAT2,Acot6,ACSL5,ACSS2,APOA1,APOA2,APOA5,APOE,AQP8,ASS1,C1QA,C1R,C3,C5,C6,C8A,C9,CCND1,CCNE1,CD36,CDS2,CES3,CFI,CHAF1B,CHKA,CHRNA2,COX6B2,CPS1,CROT,CS,CYB5B,CYP2B6,Cyp2c54 (includes others),CYP7A1,CYP7B1,DHCR7,DR1,DYNLL1,ECH1,ECI1,ECI2,EHHADH,ELOVL3,ELOVL5,ELOVL6,FABP5,FADS1,FADS2,FASN,FDPS,FGA,FGB,FGG,G0S2,G6PC,G6PD,GLUL,GPAM,GPD1,GPD2,GSTM3,GSTP1,GSTT2/GSTT2B,HADHB,HGF,HIF1A,HLA-DQA1,HMGCR,HMGCS1,HMGCS2,Hmgn2 (includes others),HSD17B11,HSD3B1,IGFBP2,IGFBP5,IL1B,KRT8,KYAT3,LGALS4,LIPG,LPCAT3,LSS,MGST3,Mup1 (includes others),NR1D1,NR1H3,OCLN,OGG1,Orm1 (includes others),PCK1,PCNA,PEX11A,PKLR,PLIN4,PLIN5,PLTP,PMVK,PON1,PPARD,PTGR1,RAD51B,RBP4,RETSAT,RTN4,SC5D,SCARB1,SCD,SCP2,SELENBP1,SIRT5,SLC25A20,SLC27A2,SLC2A4,SMC2,SORD,SQLE,TERT,TJP1,TLR5,TMEM98,TOP2A,UGT1A10 (includes others)
**TFAP2C**	-0.368	5.69E-03	ANLN,Ccl9,EGFR,ERBB2,ESR1,FMN1,FST,GPX1,IGFBP5,KRT8,MAGI3,SFTPD,TACC1
**COL18A1**	-0.376	2.27E-02	BAD,CCND1,EGFR,ETS1,F3,HGF,HIF1A,ID3,JUNB,LDHA,PDCD5,STAT1,VDAC1
**RUVBL1**	-0.378	8.85E-04	CYP4F3,ETS1,KDM4B,MCM3,NUCKS1,PCNA,QKI,RBL1,SESN2,TMEM19
**Rxr**	-0.391	1.52E-03	ABCA1,ABCC2,APOE,BMP7,BTG2,C1QA,CD36,CYP2B6,CYP7A1,GADD45A,GAS2,IL1B,NR0B2,NR1H3,PCK1,PDGFA,PLTP,SERPINF1,TERT
**SP3**	-0.402	1.23E-02	ABCA1,AKAP12,CBS/CBSL,CCND1,CD2AP,Ces2c,CYP17A1,CYP7B1,DMD,EGFR,EGR1,ESR1,F3,FAS,HGF,HSPA5,LAMA1,LCAT,LDLR,MAT2B,PCYT1A,RBL1,RRAS,SCARB1,STAT1,TERT,TGFBR2
**FOXO4**	-0.407	1.05E-03	ACACA,ACLY,C1QA,CCND1,DHCR24,ELOVL5,ELOVL6,ENPEP,FABP5,FASN,GADD45A,HIF1A,HMGCR,HMGCS1,SCD,Scd2,ZFAND5
**Esrra**	-0.440	1.80E-03	ACADM,ACAT1,ACSL5,AGT,BDH1,DLAT,ECH1,ERBB2,GABPA,GCGR,HGF,HSD3B1,PCK1,PDK1,Scd2,SIRT3,SIRT5,STARD3
**IgG**	-0.451	3.26E-02	APOE,ATP1B1,C3,C5,CALR,DDIT3,DHCR7,EGR1,FABP5,FST,GPX1,GUK1,HSPA5,HSPB1,IL1B,LDLR,PEA15,PPIF,PPL,PRSS8,RND2,SERPINF1,TLR3,UGCG,ZFP36
**S100A9**	-0.470	4.62E-02	ADAMTS5,AKAP12,BACE1,BCHE,BCL3,BHLHE40,C3,CCND1,CP,CSF2RB,ENTPD2,FAS,IL1B,IL33,LOC102724788/PRODH,LTBP1,ROBO2,RXRG,SCUBE1,UGT1A6
**NR3C1**	-0.479	8.16E-07	ABCA1,ACAT1,AGT,APOA2,APOE,ATP1B1,BHLHE40,BTG1,C1R,CASP7,CAVIN2,CCND1,CLK2,CPS1,CYP2A6 (includes others),CYP7A1,DAXX,DEAF1,DUT,DYNLL1,EGR1,ELMO2,ELMOD3,ETNK2,F3,G6PC,G6PD,GADD45A,GLUL,GPX1,GSTP1,HMGCS2,HSD11B1,HSD17B12,Hsd3b4 (includes others),IDH1,IFT122,IGFALS,IL1B,IL33,JADE1,KLK3,LIPC,MAGI3,MAP3K20,MKNK2,MSRB1,MYD88,NFIL3,NOL3,NR0B2,NR1I3,NSMCE3,OCLN,PCK1,PDE4B,PDGFA,PEA15,PFKFB3,PIGR,PLAGL1,POGK,PPP1CB,PPP1R13L,PTGR1,RNF11,ROCK1,RTN4,SCAP,SCARB1,SERPINA1,SERPINB9,SLC19A2,SLC25A33,SLC2A5,SLC43A1,SMAD1,STAT5A,TCF19,TIMP3,TIPARP,TLE3,TLR5,TM2D2,TNFRSF1B,TSC22D3,TUBA4A,UGCG,UNC13B,Uox,WDR12,ZNF48
**NFkB (complex)**	-0.479	1.12E-03	ADAMTS9,AGT,APOE,ATP2A2,BACE1,BAD,BCL3,C1R,C3,CASP7,CCND1,CD36,CD74,CFB,CLU,CREB3,CREM,CRP,CSNK2A1,Cyb5r3,DAXX,DBP,DDIT3,EGFR,EGR1,ERBB2,ETS1,F3,FABP5,FAS,FGG,FOXF1,FST,G0S2,GADD45A,GAS6,GCH1,GCLC,HIF1A,HLA-A,HSPB1,IGFBP2,IGKC,IL1B,IRF7,JUNB,MFHAS1,MTSS1,NLRP12,NR1D1,NUAK2,P2RY2,PEA15,PPIF,PSMB9,RRAS,RRAS2,SENP2,SERPINA3,SLC11A2,SLC22A4,SLC2A2,SLC2A4,SLC2A5,SLPI,STAT5A,TERT,TFPI2,TFRC,TNFSF10,TRAF2,ZC3H12A,ZFP36
**HNF1A**	-0.486	3.78E-13	ABCC2,ABCC9,ACAT2,ADH4,AFP,AGT,AHSG,Akr1c19,ANKS4B,AP3M1,APOA2,APOH,ASAH2,ASGR2,BCL6B,BMP2K,C5,C8A,C8B,C8G,C9,CBS/CBSL,CCND1,CCNE1,CDC45,CDO1,CLDN2,CRP,CTDSPL2,CYB5B,CYP2E1,CYP7A1,DPP4,DTL,ENPEP,FAS,FGA,FGB,FRK,G0S2,G6PC,GATM,GC,GLUL,GRHPR,HMGCR,HNMT,HSD11B1,HSPA5,ITIH4,KYAT3,LBP,LCAT,LDLR,MAPK15,MID1IP1,NOP16,NR0B2,NR1D1,PCK1,PCNP,PCSK9,PDK1,PIGR,PKLR,PLG,PROC,SERPINA1,SERPING1,SGK2,SLC17A2,SLC2A2,SLC38A4,Slco1a1,SLPI,SPHK2,Sult1d1,TEF,TFRC,TM4SF4,TMED10,UGT1A1,UGT1A10 (includes others),VTN,WDR12,WNK4,YPEL3
**TICAM1**	-0.505	1.92E-02	BCL3,CFB,EGR1,FAS,Ifi47,Igtp,IL1B,IRF7,IRGM,Irgm1,ISG20,LPAR1,MCM2,PDE4B,PSTPIP2,SAMHD1,TLR3,TNFSF10,TOR1AIP2
**JAK1**	-0.543	2.70E-04	BCL3,CD14,HIF1A,HLA-A,IL1B,IL4R,IRF7,IRF9,JUNB,LRG1,Orm1 (includes others),PSMB9,SNAI1,STAT1,STAT2
**PPARGC1A**	-0.552	8.12E-09	ACACA,ACACB,ACADM,ACADVL,ACAT1,ADAMTS5,AGT,BDH1,C3,CD36,CS,CYP2A6 (includes others),CYP2B6,CYP7A1,DLAT,DR1,EGFR,ERBB2,FAM20A,FASN,FDPS,G6PC,GABPA,GAS6,GPAM,GPX1,HMGCL,HMGCS2,IGFBP5,IL4R,KYAT3,LDHA,LDLR,LIPC,MCM3,MCM5,MCM6,ME1,MT-CO1,NR1H3,NR1I3,OGN,PCK1,PDGFA,PHYH,PLIN5,PPARGC1B,PRDX3,RBP4,SCARB1,SCD,SERPINA3,SESN2,SIRT3,SIRT5,SLC25A20,SLC2A4,TIMP3,TNFSF10,UGT1A1
**IL6**	-0.555	1.87E-07	ABCA1,ABCC2,Acp5,ADAMTS5,ADGRE5,AFP,AGT,APOA1,APOE,AQP4,ATP2A2,BCL3,BTC,BTG2,C3,CASP3,CASP7,CCND1,CCNE1,CD14,CD36,CD74,CDC45,CHEK1,CLDN2,CLU,CP,CRP,CSF2RB,CST3,CYP2B6,CYP2E1,CYTIP,E2F4,EGFR,EGR1,F3,FAS,FCGRT,FGA,FGB,FGG,FOXM1,FRRS1,G6PC,GADD45A,GCH1,Hamp/Hamp2,HGF,HIF1A,HLA-A,HLA-DMA,HLA-DQA1,HLA-DRB5,HP,HSPA5,IGFBP5,IL4R,IRF9,JUNB,KLK3,KRT8,LBP,LCAT,LDLR,LGR4,LIG1,LRG1,MARCHF7,MCM3,MYD88,NUCB2,Orm1 (includes others),OSBPL1A,PCNA,PDGFA,PLG,PON1,PRNP,PROM1,PSMB9,RRM2,SC5D,SENP2,SERPINA1,SERPINA3,SLC2A2,SLC2A4,Slco1a1,SNAI1,STAT1,TERT,TFR2,TFRC,THPO,THRSP,TLR3,TLR5,TNFRSF1B,TNFSF10,TOP2A,WEE1,WNT4
**PER2**	-0.555	3.38E-03	ARNTL,CCND1,CCNE1,DBP,PER3
**calpain**	-0.555	4.34E-02	AQP4,CCND1,DDIT3,HSPA5,NTRK2
**RB1**	-0.570	3.30E-03	ASF1B,ATAD2,ATP1B1,AURKB,BLM,CASP3,CASP7,CCND1,CCNE1,CDCA7L,CELSR1,CENPK,CERT1,CHAF1A,CNOT6L,DCK,DDIT3,DEK,DLAT,EGR1,EI24,F3,FAS,FST,GPT2,HRAS,LACTB,LIG1,MCM2,MCM3,MCM5,MCM6,MELK,PCNA,PGD,PPARGC1B,PROM1,PTPN4,RAD51,RAD51AP1,RBL1,RFC4,RRM1,RRM2,SUZ12,TCF19,TERT,TMEM97,TMPO,TYMS,VDAC1
**Sik1**	-0.579	5.57E-04	ACACA,CREM,FASN,GPD1,THRSP
**Akt**	-0.596	7.03E-04	AMOT,BAD,BHLHE40,BTG2,CCND1,CD36,CP,E2F4,EGFR,ERBB3,ESR1,ETS1,F3,FAS,FHIT,FOXM1,GSS,HIF1A,IGFBP2,IL1B,MT-CO1,NFIL3,NOX4,NR0B2,PCK1,PCNA,PEA15,PLG,PPARD,RAD51,RRM2,SCARB1,SLC2A4,SNAI1,TERT,TIPARP,TJP1,TMED10,TNFSF10
**FOXA2**	-0.602	2.63E-02	ABCA3,AFP,APOA1,C3,C5,CCND1,CFAP126,CHRD,Cyp2a12/Cyp2a22,CYP2A6 (includes others),FABP5,FGB,G6PC,GSTM3,HMGCS2,IGFBP5,IL33,PCK1,PON1,PRNP,SCD,SERPINA1,SFTPD,SLC2A2,SLC2A4,SNAI1
**HMGA1**	-0.611	2.65E-02	ARL4A,CCND1,CD19,CLCN6,CTSH,CYP7A1,DDIT3,DHCR7,EGR1,ESR1,HCK,HMGCR,ID3,IGFALS,JUNB,KLF2,LAMA1,RAN,SLC27A2
**HRG**	-0.640	3.60E-03	C1QA,CD74,FCGR3A/FCGR3B,HLA-DQA1,SNAI1,TIMP3
**ATF6**	-0.647	1.62E-09	ATP2A2,CALR,CHKA,DDIT3,DNAJB11,DNAJC3,FDPS,G6PC,HMMR,HSP90B1,HSPA5,MANF,NR0B2,NUCB2,PCK1,PDIA4,PRNP,RAN,SLC35B1,SYVN1,TROAP,UNC13B
**FADD**	-0.659	4.09E-03	CASP3,CASP7,CCNE1,EGR1,FAS,FOXM1,IRF7,JUNB,NAT1,NFIL3,RNPC3,STAT1,STAT2,TIPARP,TLR3
**GATA1**	-0.681	1.02E-02	BTG2,CA1,CALR,CBFA2T3,CCND1,CD164,CD36,CREM,DNTT,DUT,EGR1,ETS1,FAM118A,GUCY1B1,HDAC11,JUNB,MCM3,MCM5,NFE2,NFIL3,PCNA,PLXNA2,PSTPIP2,RBL1,RFC4,RRM1,STAT5A,TFR2,TFRC,TGFB1I1,TOP2A,UHRF1,UNG,ZBTB42,ZFPM1
**NR3C2**	-0.687	1.09E-02	ABCC9,AGT,APOE,AQP4,ATP1B1,CYFIP2,ECHDC2,EGR1,GPX1,HLA-DQB1,IL1B,JUNB,MSRB1,NOX4,NSMCE3,PLIN4,SERPINA3,TSC22D3,TUBA4A
**IL33**	-0.706	3.05E-02	ABCA1,ACAT1,APOE,BCL3,CCND1,CD36,Cd52,EPHX2,FAS,FBXL21P,G0S2,HCK,HDC,HIF1A,IL1B,ITGA5,ITIH1,JUNB,LAMA5,NCF4,PAPSS2,PEA15,RAI14,SCARB1,STAT5A,TNFRSF1B,TNFSF10,ZC3H12A
**HDL-cholesterol**	-0.707	7.38E-03	CHAC1,CREM,DHCR7,EGR1,GPT2,HMGCS1,SLC2A2,SQLE
**AHR**	-0.718	1.34E-11	1810008I18Rik,ACACA,ADAMTS5,ARNT,ATOH8,BTG2,CCND1,CCNE1,CCNF,CD14,CD36,CELA1,Ces2c,CRP,CYP17A1,CYP2A6 (includes others),CYP2B6,DCLK3,DNAJB1,ENHO,ENPEP,ESR1,FAS,FBLN5,GADD45A,GAS6,GPAM,GSTA5,HIF1A,HMGCR,HP,Hsd3b4 (includes others),HSPA5,HSPH1,IL1B,ITGA5,JUNB,KLF2,LBP,LDHA,LTBP1,MMD2,MT-CO1,NID1,NRG4,OLFML1,PCK1,Pcp4l1,PDE4B,PKLR,PLXNA2,POLA1,RBL1,RDH16,SCD,SDR9C7,SERPINA12,SHB,SLC16A5,SLC17A2,SNAI1,TGFBR2,TIMP3,TIPARP,TNFRSF19,UGT1A1,UGT1A10 (includes others),UGT1A6
**CD44**	-0.724	4.04E-02	ABCA5,CALR,CCND1,CD36,CELA1,CLU,CXADR,Cyb5r3,DDIT3,ERBB2,FAS,FASN,GADD45A,HMMR,IL1B,ITGA5,LIMS2,LTBP1,NOX4,SBK1,SERPINA1,SMAD1,SMARCD1,STAT2
**EGFR**	-0.731	4.61E-03	AHCTF1,BCL7C,BTC,BTG1,CASP3,CCND1,CDCP1,CHKA,CLDN2,CST3,DDIT3,DSC2,EGFR,EGR1,ERBB2,ERBB3,ESR1,F3,FASN,GADD45A,GAS5,GPC1,HDC,HIF1A,HP,HSP90B1,HSPA5,HSPB1,IGFBP5,IL1B,IL33,JUNB,KLK3,MTHFR,PCNA,PDK1,PROM1,RAI14,RRAS,SDF2L1,SERPINA3,SLPI,TCERG1,TIMM9,TPST1,TUBA4A,WEE1,WNT4
**NR0B2**	-0.735	3.08E-06	ABCA1,ACACA,ACADM,ACLY,AGT,APOA1,CD36,Ces2c,CYP7A1,CYP7B1,EGR1,FASN,G6PC,HDC,HMGCR,NR0B2,PCK1,PDIA3,PDIA4,SCARB1,SCD
**NKX2-1**	-0.735	2.55E-04	AGT,BDH1,C5,CD14,CD74,CLU,DCK,DPP4,FGFR4,HCK,HELLS,HLA-A,LRG1,MREG,NECTIN3,PGM3,PLP1,PON1,QKI,QSOX1,RAD51,RRM2,SCD,SERPINB9,SFTPD,SNAI1,SSBP1,WNT4,ZNF519

Table S5 List of Upstream Regulators signficantly enriched and differentially activated/inhibited in the CCl4 model (KO vs WT).

Upstream Regulator	Z-Score	p-value	Target Molecules in Dataset
**TP53**	-0.747	1.16E-24	ABCC2,ACAA2,ACACB,ACADM,ACADS,ACADVL,ACAT1,ACLY,ACOT11,ACSL3,ACSS2,ADGRE5,AFP,AHSA1,AKAP12,AMOTL2,ANLN,APOA1,APOE,ARHGAP5,ASF1B,ASS1,ATAD2,ATL3,AURKB,BAD,BCAS3,BCL3,BCL6B,BHLHE40,BIK,BMP1,BTG1,BTG2,C9,Cald1,CALU,CASP3,CCND1,CCNE1,CCNF,CD36,CELSR1,CENPF,Ces1b/Ces1c,CHEK1,CHMP4C,CLU,CP,CREB3,CS,CTSF,CTSH,Cyb5r3,CYFIP2,DBP,DCK,DDIAS,DDIT3,DDX3X,DEK,DHCR24,DHCR7,DLAT,DLG1,DNAJB5,DPP4,DTYMK,DUT,E2F4,ECH1,EGFR,EGR1,EI24,ENTPD5,ERBB2,ESR1,F3,F8A1 (includes others),FABP5,FAS,FASN,FDPS,FOXM1,G0S2,G6PC,G6PD,GABPA,GADD45A,GAS6,GAS7,GATM,GC,GDA,GLUL,GPD1,GPX1,GSTM1,GSTP1,HADHB,HDC,HGF,HIF1A,HJURP,HLA-DQA1,HMGCR,HMGCS1,Hmgn2 (includes others),HMMR,HRAS,HSPA8,HSPB1,HSPG2,HSPH1,HTT,ID3,IDH1,IFFO1,IGFBP2,IGFBP5,IL1B,IL4R,IREB2,IRF7,IRF9,ISCU,JUNB,KLK3,KNTC1,KRT8,LAMA5,LBR,LDHA,LDLR,LOC102724788/PRODH,LRRC3,LSS,LTBP1,MAP1LC3A,MAPK6,MBNL2,MCM2,MCM3,MCM5,MCM6,ME1,MELK,MIS18A,MPDZ,MPV17L,MPZL2,MRPL18,MRPL34,MRPL38,MSRB1,MT-ND5,Mup1 (includes others),NAB1,NCAPG,NCAPH,NDC80,NECTIN3,NFE2,NINJ1,NLRP12,NOL3,NOX4,NR0B2,NR2C1,NUSAP1,OAS1,OSGIN1,P4HA1,PANK1,PBK,PCBP4,PCK1,PCNA,PDE4B,PDGFA,PDIA6,PDK1,PDLIM1,PDSS2,PEA15,PEG3,PFKFB1,PFKFB3,PGD,PGM3,PHLDA3,PLAAT3,PLTP,PMS2,POLA1,PPARD,PPARGC1B,PPP1R13L,PRDX3,PRKCA,PRNP,PROM1,PSMB9,PSRC1,PSTPIP2,QDPR,RAD23A,RAD51,RAD51AP1,RAN,RAP2A,RB1CC1,RBBP4,RBL1,RFC4,ROBO1,RPL10,RPS26,RPS3,RPS6KA1,RRM1,RRM2,RRP1B,SAMM50,SCD,SCP2,SERPINA3,SERPINB9,SERPING1,SESN2,SFPQ,SHROOM3,SIRT3,SIRT5,SLC19A2,SLC2A4,SLPI,SMC2,SNAI1,SNRK,SPDL1,SPHK2,SQLE,STARD4,STAT1,STIP1,STMN1,SULF2,TAGLN2,TDP2,TERT,TFPI2,TFRC,TGFB1I1,TGFBR2,TIMM9,TIMP3,TJP1,TKT,TLR3,TMED7,TMEM97,TNFRSF1B,TNFSF10,TOP2A,TP53INP1,TRIAP1,TSC22D3,TYMS,UGDH,UHRF1,UNG,USP14,VDAC1,WEE1,XPO1,YPEL3,ZC3H12A,ZFP36
**THRA**	-0.760	1.76E-02	ACACA,APOA1,APOA5,ATP2A2,CCND1,CSNK1A1,CTSH,CYP7A1,DR1,EHHADH,FASN,GPD2,LDLR,PCK1,PROM1,RCAN2,SLC2A4,THRSP
**Ifn gamma**	-0.762	1.01E-02	ADAR,AGT,CASP3,ESR1,FAS,IGFBP5,IL1B,IL33,LAMA1,MYD88,PCK1,STAT1,TERT,TLR3,TNFSF10
**PTEN**	-0.771	3.28E-04	ACAA2,ACACA,ARF4,ARHGAP5,AURKB,BCL3,BTG1,BTG2,C3,CASP7,CCND1,CCNE1,CELSR1,CHEK1,CPEB3,CS,DLAT,ECI2,EGFR,EHHADH,ELOVL6,ERBB2,ERBB3,ESR1,ETS1,F3,FAS,FASN,FGFR4,FOXM1,G0S2,GCAT,GPR146,GSS,GTF2A1,HADHB,HEXB,HIF1A,HLA-DMA,HMGCR,HMGCS1,HSD17B7,IGFBP2,IGFBP5,IL1B,IL4R,KAT2A,KIF1B,LDLR,LIMS1,LSS,MAFK,ME1,MT-ATP6,MT-ND1,NFIL3,PFKFB3,PKLR,RAD51,RAD51B,RPS6,RRM1,SCD,SDHAF1,SFTPD,SLC20A1,SLC30A10,SMAD1,SNAI1,SORD,STAT5A,SULF2,TAF10,TGFBR2,TLE2,TNFSF10,TSC22D3,VHL,YPEL3
**H1-6**	-0.775	2.92E-03	ATP6V0E2,BCL3,CBFA2T3,CD36,COPS7B,FDPS,GLUL,IRGM,MARS1,MCM6,MRPL38,PCBP4,RRM1,SGF29,USF2
**H1f1**	-0.775	2.92E-03	ATP6V0E2,BCL3,CBFA2T3,CD36,COPS7B,FDPS,GLUL,IRGM,MARS1,MCM6,MRPL38,PCBP4,RRM1,SGF29,USF2
**VHL**	-0.775	9.38E-03	BHLHE40,CCND1,CDCP1,EGFR,F3,GLO1,GPX1,HIF1A,ITGA5,JADE1,NFE2,NOX4,PCNA,PFKFB3,RPS6,TFRC,TMED3,TNFSF10,TYMS
**ADRB**	-0.786	5.91E-07	AACS,ACAT2,ANXA5,ATF5,BAD,BHLHE40,CASP3,CAVIN2,CCND1,CCNE1,CREM,DDIT3,E2F4,FAS,FDPS,GADD45A,GSTA5,HMGCS1,HSD17B7,IDH1,JUNB,LSS,LTBP1,NR4A1,PCNA,RAD51,RAN,SRXN1,WEE1,YPEL3
**TFAM**	-0.816	3.60E-03	ACADM,ACADS,ECH1,FABP5,IL1B,SLC25A20
**B2M**	-0.816	6.39E-03	FCGRT,Hamp/Hamp2,HLA-A,HLA-DQA1,HLA-DQB1,HLA-DRB5,IL1B
**SFRP1**	-0.817	4.58E-05	ACACA,ACLY,BACE1,BIK,CASP3,CCND1,ELOVL6,FASN,G6PC,NR1H3,PCK1,SCD,SLC2A2,SLC2A4
**LRPAP1**	-0.831	6.04E-03	CYP17A1,DDIT3,F3,IL1B,LDLR,SERPINA3
**SASH1**	-0.832	3.50E-03	ACP3,DAXX,HDC,HELZ2,IRF7,IRGM,ISG20,RBL1,STAT1,STAT2,TLR3,TRIM21,USP12
**IL24**	-0.844	3.73E-02	CASP3,CCND1,DDIT3,FAS,GADD45A,HSP90B1,HSPA5,XRCC4
**BTG2**	-0.861	1.17E-02	BTG2,CCND1,CCNE1,DIAPH3,IL1B,NOX4
**THRB**	-0.863	4.30E-05	ACACA,APOA1,APOA2,APOA5,ATP2A2,BTG2,CCND1,CCNE1,CDCA7L,CSNK1A1,CTSH,CYP7A1,DR1,EGFR,EHHADH,ERBB3,FASN,FDPS,FRAT1,G6PC,GCH1,HIF1A,IGFBP2,LDLR,ME1,NR1H3,NR4A1,PCK1,PGD,PHYH,PROM1,SLC2A4,THRSP,TLE2,WNT4
**MED13**	-0.879	1.01E-08	AACS,ACACA,CD36,CLSTN3,CYP2A6 (includes others),ELOVL6,FASN,G6PC,GPD1,GPD2,HMGCR,NTRK2,PCSK9,PKLR,SCD,SLC2A4,THRSP,TKT
**JUN**	-0.885	4.83E-03	ACAT2,ADH7,AFP,AKAP12,APOE,BCL3,BTG1,C3,C5,CCND1,CD14,CD164,CLDN2,CLU,CYP17A1,CYP7A1,EGFR,EPHX2,F3,FAS,FBLN5,GADD45A,GAS6,GCLC,GSTA5,GSTP1,IGFBP2,IL1B,JUNB,LBP,LPAR1,LTBP1,MOGS,MTHFR,MYLPF,NR0B2,NR4A1,PDK1,PEA15,PLAGL1,PTBP2,PXDN,SCD,SERPINB9,SFTPD,SNRPN,STAT1,STMN1,SULF2,TIMP3,UGT1A3,WNT4,ZFP36,ZNF385A
**FOS**	-0.897	2.18E-08	ABCG5,ACACA,Acp5,AGT,AKR1D1,AMIGO1,ANK3,ARID3A,ARNT,ATF7IP,C3,C5,CALU,CASP3,CAST,CCND1,CCNE1,CD14,CD164,CLDN2,CLU,CTSH,CYP17A1,CYP7A1,DDX3X,DLG1,EGR1,ELOVL3,ELOVL6,EPHX2,F3,FASN,FETUB,FGA,FMN1,G6PC,GAS2,GAS5,GM2A,GSTA5,GSTP1,HMMR,HSD3B1,HSP90B1,HSPA5,IGFBP5,JUNB,KIF1B,KRT8,LAMA3,LGALS3BP,LGALS4,LPCAT3,LTBP1,MAFK,MAP3K20,MAPK6,MCM5,MPZL2,NAA80,NFIL3,NR1H3,NR3C2,NR4A1,PLAGL1,PRCC,QKI,RBBP4,RGS4,RPS6,SCD,SELENBP1,SERPINB9,SFTPD,SLPI,SNAI1,STMN1,SULF2,TIPARP,Uba52,UGCG,WEE1,WNT4
**KLF11**	-0.905	6.27E-03	ACSL5,AGT,BMP7,CHRNA2,CHRNA4,ELOVL3,HADHB,HGF,IL1B,INHBE,LTBP1,PDGFA,PLG,SERPINA1,SLC25A20,SNAI1,STAT1,TGFBR2,TIMP3
**CTNNBIP1**	-0.905	1.16E-02	CCND1,HMGCR,HMGCS1,KLK3
**NCOA1**	-0.906	1.39E-03	ABCA1,ACSL4,BTG2,C3,CASP7,CCND1,CYP2B6,CYP7A1,DBP,DDX28,EGR1,ERBB3,HIF1A,JUNB,KLK3,NR0B2,NR1I3,PCK1,PKLR,PPARD,SLC2A2,THPO,THRSP
**LDL**	-0.913	1.65E-04	ABCA1,APOE,ATP2A2,BAD,C3,CASP3,CASP7,CBS/CBSL,CCND1,CD36,CREM,DDIT3,EGR1,ERBB2,F3,FAS,FDPS,G0S2,GAS6,GCLC,GPX1,HIF1A,HMGCR,HSP90B1,HSPA5,HYOU1,IGFBP2,IL1B,IRF7,LDLR,LIMS1,NPC1,NR0B2,NR1H3,NR4A1,SCARB1,TGFBR2,TNFSF10
**ANGPTL4**	-0.919	1.16E-02	AGPAT2,CD36,FASN,STAT1
**PRKAA**	-0.921	1.38E-02	FASN,G6PC,HSD11B1,HYOU1,OGG1,PCK1,SNAI1,STAT1
**FOXO3**	-0.925	1.23E-02	ACLY,AQP4,BTG1,C1QA,CCND1,CDC45,DDIT3,EGR1,ELOVL5,ENPEP,ESR1,FABP5,FASN,FOXM1,GADD45A,GCLC,GLUL,GPX1,HIF1A,HMGCR,HMGCS2,JUNB,MAP1LC3A,MCM2,NCAPG2,NOL3,NT5E,PBK,PCNA,PRDX3,PRNP,RTN4,SKA2,SNAI1,TIPIN,TNFRSF1B,TNFSF10,TP53INP1
**MAPK8**	-0.934	4.71E-03	ABCA1,ACSL4,APOA1,APOE,CASP3,CCND1,CRP,CYP7A1,DDIT3,EGR1,ETS1,G6PC,GADD45A,GSTM1,IL1B,MTHFR,NOX4,PPARD,PPARGC1B,RGS4,SESN2,TSC22D3,WNT4,ZFP36
**FSH**	-0.938	3.15E-03	ACP3,ADCY9,AKAP12,AMOTL2,ATP2A2,BAD,BTC,BTG2,CCND1,COL15A1,CREM,CSNK1A1,CYFIP2,CYP17A1,DHCR7,DHRS3,EGFR,ESR1,FST,GCLC,GDE1,HSD11B1,HSD3B1,ISG20,ITGA5,JUNB,LDLR,MAPK6,MYRF,NOL3,NR4A1,PCK1,PCNA,PLIN3,PPP1CB,PRNP,RGS4,SCARB1,SMAD1,SMARCD1,SSTR2,STAT1,STIP1,TFPI2,TFRC,TGFBR2,TRIB1,ZNF519
**IRF1**	-0.939	2.20E-02	C1R,CASP3,CASP7,CCND1,CFB,HELZ2,Ifi47,IL1B,IRF7,IRF9,NFE2,OAS1,PCNA,PIGR,PLAAT3,PSMB9,SLPI,STAT1,STAT2,TERT,TLR3,TNFSF10,TRIM21
**JAK1/2**	-0.954	3.50E-03	CD74,FAM81A,GBP5,GDA,GLDC,HLA-DQA1,HLA-DRB5,Ifi47,IRF7,LMO7,PSMB9,THPO,WIPF3
**DUSP1**	-0.954	4.05E-02	AACS,CFB,CYP17A1,EGFR,ELOVL3,HELZ2,IL1B,ISG20,PDGFA,PEX11A,PNRC1,RETSAT,SERPINA12,STAT5A,ZFP36
**CEBPD**	-0.957	1.20E-02	AGT,C3,CCND1,CD14,CLU,CYP2A6 (includes others),CYP2E1,DDIT3,HGF,HIF1A,HP,IGFBP5,IL1B,PDGFA,SFTPD
**ONECUT2**	-0.975	2.12E-03	EGFR,KLK3,NAT1,SYTL4
**CYP3A**	-1.000	5.17E-04	CYP7A1,CYP7B1,HMGCS1,SQLE
**Pro-inflammatory Cytokine**	-1.000	6.68E-03	ABCC2,APOE,ASS1,CLU,F3,GCLC,GLUL,HP,HSPA5,LIPG,NR4A1,S100A1
**Irgm1**	-1.000	7.85E-03	AURKB,CDCA3,DTL,FAS,ID3,Ifi27l2a/Ifi27l2b,IRF7,NCAPG,RRM2
**CERS2**	-1.000	8.23E-03	DDIT3,GSTA5,Gstm3,SMPD3
**NELFB**	-1.000	1.16E-02	GADD45A,Ifi27l2a/Ifi27l2b,MKNK2,RPS3
**48s**	-1.000	2.64E-02	ATF5,CCND1,DDIT3,HSPA5
**OMA1**	-1.000	3.30E-02	ACADVL,FASN,PPARGC1B,SCD
**HCAR2**	-1.000	4.90E-02	IL1B,IQGAP3,ROPN1L,WEE1
**TNF**	-1.035	2.72E-12	ABCA1,ABCC2,ACACA,ACADM,ACADS,ACADVL,Acp5,ADAMTS5,AGT,AKAP12,Aldh1a7,ANK3,APOA1,APOE,ARF4,ARHGAP18,ASS1,ATP2A2,AVPR1A,BACE1,BCL3,BHLHE40,BIK,BTG1,BTG2,C3,C5,CALR,CASP3,CASP7,Ccl9,CCND1,CCNE1,CD14,CD36,CDO1,CERT1,CFB,CLU,COL15A1,CP,CREB3,CREB3L3,CREM,CRP,CRY1,CSF2RB,CTSF,CYP17A1,CYP2E1,CYP7A1,CYP7B1,CYTIP,DAXX,DDIAS,DDIT3,DHRS3,DMD,DPP4,DPYS,DSC2,ECH1,EGFR,EGR1,ENTPD5,ERBB2,ESR1,ETS1,F3,FABP5,FADS1,FAS,FASN,FCGRT,FDPS,FGG,FOXF1,FST,G0S2,GADD45A,GAS5,GCH1,GCLC,GM2A,GPAM,GPD1,GPD2,GPX1,GSTM2,GSTP1,Hamp/Hamp2,HCN3,HDC,HEXB,HGF,HIF1A,HLA-A,HMGCR,Hmgn2 (includes others),HP,HSD11B1,HSD17B7,HSP90B1,HSPA5,HSPA8,HSPG2,HUS1,ID3,IGFBP2,IGFBP5,IGKC,IL1B,IL33,IL4R,IRF7,ITGA5,JUNB,KLF2,KLK3,KRT8,LAMA3,LBP,LCAT,LDHA,LDLR,LRG1,LSS,MAP3K20,MCM3,MFHAS1,MYD88,NID1,NINJ1,NLRP12,NOX4,NR0B2,NR1H3,NR1I3,NR4A1,NUAK1,NUAK2,NUCB2,OAS1,OCLN,OGN,Orm1 (includes others),PCK1,PCNA,PCSK9,PCYT1A,PDE4B,PDE7B,PDGFA,PDIA4,PEX11A,PIGR,PILRB,PKMYT1,PLAAT3,PLIN4,PLP1,PPARD,PPARGC1B,PPIF,PPP1R3C,PRKCA,PRNP,PSMB9,QKI,RABEP2,RCAN2,RGL3,RGS4,RND1,ROBO1,RPS3,RRM1,RRM2,Rtp3,SAMD4A,SCARB1,SCD,SCUBE1,SERPINA3,SERPINB9,SERPINF1,SIRT3,SLC11A2,SLC16A2,SLC16A5,SLC20A1,SLC22A4,SLC2A2,SLC2A4,Slco1a1,Slfn2,SLPI,SMAD1,SMPD2,SNAI1,SNRK,SQLE,STARD3,STAT1,STAT5A,STMN1,SUPT4H1,SYVN1,TERT,TFPI2,TFRC,TGFBR2,THRSP,TIMP3,TJP1,TLR3,TLR5,TNFRSF1B,TNFSF10,TNS3,TP53INP1,TPST1,TRAF2,TSC22D3,TYMP,UACA,UGCG,USP2,ZC3H12A,ZFP36
**NR1H4**	-1.071	2.66E-08	ABCC2,ABCG5,ACACA,APOA1,APOA5,C3,CCNE1,CYP2B6,CYP7A1,FABP5,FAS,FASN,FGB,FOXM1,G6PC,GPX1,HSPA5,IL1B,LCAT,LDHA,LIPC,NFAT5,NR0B2,NR1I3,Orm1 (includes others),PCK1,PKLR,PLTP,PNPLA3,PON1,PPARGC1B,SCARB1,SLC51B,Slco1a1,Sult1a1,UGT1A1
**CAV1**	-1.072	7.58E-06	AFP,CCND1,CCNE1,CD14,CD276,CD36,EGR1,ESR1,FAS,FGB,GIPC2,HGF,HSD17B12,HSPA8,KRT8,NEDD4L,NR3C2,NR4A1,NTRK2,OCLN,PCNA,PROM1,SLC20A1,SNAI1,SPAG9,TGFB1I1,TJP1,TRAF2
**LMNA**	-1.103	9.12E-03	9130221H12Rik,AFP,AHCTF1,CAMK1D,DIS3L2,FNIP1,GCLC,GRAP,GSTT2/GSTT2B,H3-3A/H3-3B,ID3,Irgm1,ISG20,LMNB1,LRP4,LYSMD3,MBIP,NPC1,NPR2,PCGF6,PDP2,PPARGC1B,RHPN2,SLC25A42,SNRK,STAT1,TBC1D23,TIMP3,TMPO,ZFP36,ZNF519
**CREB3**	-1.103	3.30E-02	DDIT3,HSPA5,KLK3,MBTPS2
**CEBPB**	-1.111	1.10E-03	ADH7,AGT,ATR,BLNK,C3,CCND1,CCNE1,CD14,CDC45,CDO1,CP,CRP,CYP17A1,CYP2A6 (includes others),Cyp2d9 (includes others),CYP2E1,DDIT3,DHCR7,ENTPD2,FAS,G6PC,GADD45A,Hamp/Hamp2,HDC,HGF,HLA-A,HP,HSD11B1,HSPA8,IGKC,IL1B,IRF9,LBP,LDLR,MCM3,NR1H3,Orm1 (includes others),PCK1,PCNA,PEA15,PLG,PPARD,PRSS8,RGS4,RIC8B,SCARB1,SCD,SERPINA1,SFTPD,TNFRSF19,UGT1A6,USP33
**EPAS1**	-1.117	3.30E-05	ACACA,ACLY,AKAP12,BHLHE40,C1QA,CCND1,CDCP1,CHKA,CHMP2B,DDIT3,EGFR,FASN,GPX1,HIF1A,HMGCS1,HSPA5,IGFBP5,ITIH5,KDM4B,LDHA,LDLR,MANF,NFIL3,NT5E,P2RY1,PFKFB3,PRKCA,RB1CC1,SCAP,SFTPD,SLC11A2,SLC2A4,TERT,TGFBR2,TRIM21,UNG
**MYOC**	-1.131	2.09E-02	DDIT3,DIS3L2,DLG1,ENPEP,HSPA5,IGFBP5,INHBE,MAFK,NOL3,PLP1,PXDN,QKI,SESN2
**ATG5**	-1.134	8.62E-04	CASP3,CASP7,CYP2B6,HSPA5,HTT,PDIA3,RTN4,SCARB1
**ASXL1**	-1.134	1.59E-02	G0S2,GPCPD1,GPD2,HSD11B1,MCM2,PPARGC1B,SCD,SLC2A4
**ONECUT1**	-1.149	2.15E-06	ABCA8,ABCC2,ACACB,ACP3,ACSS3,AFP,APOH,C14orf119,C8G,CD36,CFI,CLDN2,COL5A3,Cyp2a12/Cyp2a22,CYP7A1,DHRS4,FABP5,FAS,FASN,G3BP2,G6PC,GABPA,GSS,GUF1,HMGCR,HSPH1,ITIH1,MGST3,NR0B2,NUAK2,OGFR,PCK1,PFKFB1,PON1,PPARD,SAMHD1,SCD,SERPINA1,SH3BGRL,SLC2A2,TM4SF4,TNFSF10,UGT1A1,WDR12,YPEL3,ZBTB45
**NPPB**	-1.153	3.52E-03	ACAT2,HMGCR,HMGCS1,HSD3B1,IL1B,LDLR,LSS,SCARB1
**MACROH2A1**	-1.155	1.80E-03	ABCA1,CD36,EGR1,ERBB2,ETS1,FASN,G6PC,GADD45A,HUS1,IRF7,SLC2A2,SLC2A4,SNAI1,TERT,THRSP
**DOCK8**	-1.155	7.52E-03	DAXX,HDC,HELZ2,IRF7,IRGM,ISG20,RBL1,STAT1,STAT2,TLR3,TRIM21,USP12
**CREB3L3**	-1.159	1.41E-02	APOA5,CRP,CYP2B6,G6PC,PCK1
**IL1A**	-1.184	4.28E-02	ABCC2,Acp5,ADAMTS5,BCL3,C3,CYP17A1,CYP2B6,CYP2E1,DPP4,FAS,GCH1,Hamp/Hamp2,HDC,HSD11B1,HSD3B1,HSPG2,IGFBP5,IL1B,ITGA5,JUNB,LDHA,PDGFA,PDZK1IP1,SERPINA1,SERPINA3,SSTR2,ZC3H12A
**TAF4**	-1.195	7.59E-03	CARMIL1,CCND1,EGFR,ETS1,GAS2,GAS5,GAS6,GTF2A1,MAFK,OAS1,PBK,PDGFA,SCUBE1,TAF10,WWC1
**RET**	-1.195	3.31E-02	CCND1,CLU,DNAJC3,EGR1,HSPA8,HSPH1,IL1B,KLK3,PER3,PITPNM1,PRNP,RNF11,RTN4,STIP1,TIMP3
**EIF3E**	-1.195	3.58E-02	CCND1,CCNF,CD36,LIG1,SMC2,SNAI1,TOP3A
**TCR**	-1.202	3.23E-02	ABCD1,ABLIM1,APOE,BCL3,CCND1,CCNE1,CS,CYTIP,ECH1,EGR1,FAS,HIF1A,HSP90B1,HSPA5,ID3,IL4R,IRF7,ISG20,JUNB,KLF2,LBR,LMNB1,LPIN2,MYD88,NR4A1,OAS1,PDIA4,RPL13A,RPL15,RPL18A,RPS3,SERPINA3,STAT1,STAT5A,TLE3,TLR3,TNFRSF1B,TRAF2,TSC22D3,USP21

Table S5 List of Upstream Regulators signficantly enriched and differentially activated/inhibited in the CCl4 model (KO vs WT).

Upstream Regulator	Z-Score	p-value	Target Molecules in Dataset
**PIK3R1**	-1.219	2.92E-03	ATF5,CCND1,CD36,DDIT3,DNAJC3,F3,GCH1,HIF1A,HSP90B1,HSPA5,IL1B,IRF7,LDHA,PCK1,PDK1
**HOXA10**	-1.225	4.54E-03	ADH4,APOE,ARL4A,BCHE,COL15A1,CYP2E1,DBP,DPP4,EGFR,FST,GAS6,HLA-DQA1,HLA-DQB1,HMGCR,IGFBP5,ME1,NR4A1,PEG3,PIGR,PPP1R13L,PROM1,SCD,Scd2,THRSP,WNT4
**IFNB1**	-1.237	3.27E-03	Acp5,ANXA5,BHLHE40,CASP3,CD14,CDKL2,CREM,CYP2E1,DAXX,GBP5,Hamp/Hamp2,HLA-A,HMGCR,HMGCS1,Ifi27l2a/Ifi27l2b,Ifi47,Igtp,IL1B,IRF7,IRF9,IRGM,Irgm1,ISG20,MCM6,MYD88,OAS1,OGN,RAD51,SLFN13,SQLE,STARD4,STAT1,STAT2,TLR3,TNFSF10,TRIM21,ZDHHC14
**MAP3K1**	-1.237	2.49E-02	CCND1,CYP7A1,EGR1,FAS,HSPB1,LDLR,PPARD,RGS4,TOP2A
**ATF4**	-1.251	1.97E-04	ABCA1,ABCG5,APOE,ATF5,CALR,CHAC1,CYP7A1,DDIT3,FASN,GADD45A,GCH1,HSP90B1,HSPA5,HYOU1,IGFBP2,IGFBP5,MARS1,MID1IP1,NOX4,NR1H3,PCK1,PEG3,SARS1,SERPINF1,SIGMAR1,SLC38A3,TKT
**CHUK**	-1.262	1.03E-02	Acp5,C3,Ccl9,CCND1,CLU,CP,CTSF,CYP7B1,F3,FAS,GCH1,GM2A,HLA-A,IL1B,JUNB,NID1,OGN,PCNA,PPARGC1B,RABEP2,Rtp3,SLC16A2,Slfn2,SMOC2,TIMP3,TLR3,TNFRSF1B
**NGFR**	-1.268	3.24E-02	CCND1,CCNE1,DMD,LDLR,NR0B2,PCNA
**TCF4**	-1.284	2.69E-05	ADAMTS9,ASF1B,BHLHE40,BMP7,C15orf39,CASP3,CBFA2T3,CCND1,CDCA3,CELA1,CRELD2,CREM,DCK,DUT,ELAPOR1,ESCO2,FAS,FBXO4,HIF1A,Hmgn2 (includes others),HMMR,HSD11B1,HSP90B1,HYOU1,ID3,IGFBP2,IGFBP5,MANF,MPV17L,NCAPG,NCBP1,NDC80,NUFIP1,OSBPL1A,PDE7B,PDIA4,PDIA6,PPIF,RPS6KA1,RPS6KC1,RRM2,SDF2L1,SERPINF1,SLC35B1,SMAD1,SMAGP,SPC24,STAT2,TERT,THPO,TNFSF10,TNS3,UBE3A,USP12,VHL,WDR12
**DUSP5**	-1.342	1.76E-02	EGR1,SLPI,SRXN1,SSTR2,ZFP36
**RIPK2**	-1.353	3.19E-04	ABCA1,ABCG5,ACACA,ACADM,CD36,CYP7A1,FASN,HMGCR,IL33,LDLR,NR1H3,PDE4B,PPARD,SCAP,SCD
**UTS2R**	-1.387	2.98E-04	ABCA1,ACAT1,NR1H3,PPARD,SCARB1
**PML**	-1.389	4.27E-10	ACACA,ACACB,ACADM,ACADS,ACSL4,AKR7A2,APOA1,APOE,CCND1,CCNE1,EGFR,ELOVL6,FAS,FASN,GADD45A,GPAM,HADHB,HMGCR,HMGCS1,HSD17B7,HSPB1,IL1B,IRF7,ISG20,LDLR,LIPC,MCM6,ME1,OAS1,PIK3C2A,PRKCA,PSMB9,RRAS2,SCD,SLC25A20,SNAI1,STAT1,STIP1,STMN1
**TOB1**	-1.395	2.54E-06	AMOTL2,ATR,CCND1,CHEK1,HJURP,KAT8,MBNL2,MIS18A,PCBP4,PHLDA3,SNRK,SPDL1,TMED7,TP53RK,UBA6
**NFIX**	-1.400	2.62E-02	CCND1,HGF,PDGFA,PKLR,SERPINA3
**Ifnar**	-1.467	1.05E-03	C3,CD74,DDIT3,HLA-A,IL1B,IRF7,IRF9,Irgm1,ISG20,MYD88,OAS1,PSMB9,STAT1,STAT2,TLR3,TNFSF10,TRIM21
**UXT**	-1.492	3.73E-02	ATR,CHEK1,HUS1,KLK3,SORD,TLR3
**SIK1/SIK1B**	-1.501	5.57E-04	ACACA,FASN,GPD1,SCD,THRSP
**E2F6**	-1.508	2.82E-04	CAMTA2,CCNE1,CDC45,LIG1,MCM2,MCM3,MCM5,RAD51,RAD51AP1,RBBP4,RFC4,RPA2,RRM2,UNG
**STAT1**	-1.518	1.02E-04	ABCA1,AGT,APOE,BAD,BTG1,C1R,C3,CASP3,CBFA2T3,CCND1,CCNE1,CD14,CFB,CREM,Cyp2d9 (includes others),CYP2E1,DPP4,EGR1,FAS,GBP5,HIF1A,HLA-DQA1,HLA-DRB5,Ifi47,Igtp,IL1B,IRF7,IRF9,Irgm1,NFE2,NOX4,OAS1,PDGFA,PPARGC1B,PSMB9,SAMHD1,SERPINA3,SERPING1,SLC2A2,SLC51B,SLFN13,Slfn2,SMAGP,STAT1,STAT2,TLR3,TNFSF10,TRAF2,TRIM21,TYMP
**HDAC1**	-1.525	6.85E-03	Acp5,AKAP12,APOA1,CAVIN2,CCND1,CCNE1,EGFR,EGR1,ESR1,FAS,FOXM1,GLUL,GSTP1,KLK3,LIG1,MCM3,MCM5,PCK1,PCYT1A,PDGFA,PFKFB3,PRIM2,RBL1,RRM2,SNAI1,TERT,TGFBR2,TNFRSF19,TOP2A,TSC22D3,TYMS,UHRF1,VHL
**HSF1**	-1.556	7.21E-03	ACACA,ACLY,AHSA1,BMP7,CCNE1,CD36,CELSR1,CLU,DNAJB1,FASN,FOXM1,Hspa1b,HSPA8,HSPB1,HSPH1,IL1B,ITGB3BP,KNTC1,LDHA,LDLR,MRPL18,PMS2,RAD51B,SCD,SPHK2,SSBP1,STIP1,TRA2B
**SAMSN1**	-1.604	1.21E-02	DAXX,HDC,HELZ2,IRF7,IRGM,ISG20,RBL1,STAT1,STAT2,STAT5A,TLR3,TRIM21,USP12,ZC3H12A
**Interferon alpha**	-1.619	3.47E-02	ADAR,AFP,ANXA5,BCL3,BTG2,C3,CASP3,CCND1,CELSR1,CMTR1,CSF2RB,CYP2E1,DAXX,E2F4,EGFR,F3,FAS,FASN,GBP5,HELZ2,HLA-A,Ifi47,IL1B,IL4R,IRF7,IRF9,ISG20,MT-CO1,MYD88,NFIL3,NT5E,OAS1,PSMB9,RNF103,SERPINB9,SHFL,SLFN13,Slfn2,STAT1,STAT2,TBC1D10A,TERT,TLR3,TNFSF10,TRIM21,TYMP,TYMS
**RBL1**	-1.646	2.97E-04	AURKB,CCND1,CCNE1,E2F4,F3,FAS,MCM2,MCM3,MCM5,MCM6,PCNA,RBL1,RRM1,RRM2,TERT,TYMS
**CNTF**	-1.664	3.00E-02	BDH1,CASP3,CRP,EGR1,FASN,JUNB,LBP,NR2C1,SCD,SERPINA3,STAT1,ZFP36
**SYK**	-1.670	6.38E-03	AKAP12,Btbd8,CCND1,CREM,FST,GADD45A,IL1B,KLF2,OAS1,PCMTD1,PLEKHG1,ROCK1,TFPI2,THPO,TNFSF10,TP53INP1,TSC22D3
**FGF19**	-1.673	5.05E-20	ACACA,ACACB,ACLY,AFP,APOE,AQP4,AQP8,CCND1,CD36,CYP17A1,Cyp2d9 (includes others),CYP7A1,CYP7B1,EGR1,ELOVL3,FABP5,FASN,FDPS,G0S2,GADD45A,GAS2,HLA-DQB1,HMGCR,NR0B2,PEG3,PKLR,PON1,PPARGC1B,PROM1,SCD,Scd2,SERPINA1,SERPINA3,SLC2A2,SLC2A5,SLPI,SQLE,THRSP
**PRL**	-1.675	1.79E-03	ADAR,ANXA5,ATP2A2,CCND1,CLU,CST3,CTSH,EGFR,EGR1,ERBB2,ERBB3,ESR1,GSTM1,HELZ2,HSD17B7,Hsd3b4 (includes others),ID3,IGFALS,IGFBP5,IRF7,IRF9,MARS1,Meg3,MLKL,OAS1,OCLN,PCNA,PDIA4,RABAC1,SAMHD1,SCARB1,SCP2,SERPINA3,SHFL,STAT1,STAT2,STAT5A
**GCG**	-1.697	4.44E-07	ACADM,ACADS,AQP8,BTG2,CASP3,CBS/CBSL,ETNPPL,FASN,FDPS,FST,G6PC,GCGR,GREM2,GSTP1,HMGCR,PCK1,Pde4d,PPARGC1B,PPP1R3C,SCARB1,SLC2A4
**ADAM12**	-1.706	1.44E-02	Ccl9,COL4A5,COL5A3,EGFR,GAS6,HGF,IGFBP5,IL33,JUNB,LAMA5,OGN,S100A1
**APP**	-1.724	4.87E-06	ABCA1,ANKS4B,ANXA5,APOE,ARF4,ARNTL,ATP1B1,ATP6V1A,BACE1,BAD,BIK,C1R,C3,CALR,CASP3,CAST,CCND1,CCNE1,CD74,CDC14A,CLU,CP,CS,CSF2RB,CSNK2A1,CYFIP2,DAXX,ECH1,EGFR,EGR1,ELMOD3,ERBB2,ESR1,Fnbp1l,Fus,GABBR2,GPANK1,GPC1,GSTM3,GSTP1,H2AJ,HDAC11,HGF,HMGCR,HSPA5,HSPA8,HSPB1,HSPG2,Ifi47,IGFBP2,IGFBP5,Igtp,IL1B,IRF7,IRGM,Irgm1,ISG20,JUNB,KAT2A,KLK3,LBP,LCAT,LDHA,LDLR,MASTL,MT-ATP6,MT-CO1,MT-ND1,MT-ND2,MT-ND5,MT-ND6,MYD88,NDUFB11,NR1D1,OCLN,OGN,Pcp4l1,PDE4B,PDGFA,PDK1,PEA15,PFKFB3,PPARD,PRKCA,PRNP,QDPR,RAN,RBL1,RFC4,ROCK1,RPS6,SCARB1,SERPINF1,SIGMAR1,SLC11A2,SLFN13,STIP1,STMN1,STX1B,TERT,TGFBR2,TJP1,TLR3,TLR5,TNFSF10,TOP2A,TOR3A,TP53INP1,TRIM21,TUBA4A,VDAC1,YPEL3,ZDHHC14
**MEF2D**	-1.772	1.38E-03	CCND1,CCNE1,GCLC,JUNB,KLF2,MCM3,MCM5,MCM6,MT-ND6,NR4A1,PCNA,SLC2A4,SRXN1,ZFP36
**CDK19**	-1.796	2.04E-02	BTG2,CLU,CYFIP2,DHCR7,FADS2,FAS,GADD45A,HMGCS1,HRAS,LSS,NINJ1,OSGIN1,PDGFA,PHLDA3
**IFI16**	-1.803	1.59E-02	BAD,CCND1,ESR1,GADD45A,GPX1,HSD3B1,IL1B,LDLR,OAS1,PCNA,SFTPD,STAT2
**ERN1**	-1.849	3.64E-05	ACACA,ARF4,ATP2A2,CYP2E1,DDIT3,DNAJB11,DNAJC3,ERO1B,ETS1,FAS,FASN,G6PC,HSP90B1,HSPA5,IL1B,MPZL2,NOX4,NR1H3,PDIA6,PIGA,SCD,SDF2L1,SLC20A1,SLC35B1,SYVN1,TMED3
**TLR4**	-1.925	1.92E-03	ACP3,C3,CASP7,CAST,CD14,CERT1,CFB,CREB3L3,DAXX,DDIT3,F3,FAS,HDC,HELZ2,HLA-DQA1,HSPA5,Ifi47,Igtp,IL1B,IRF7,IRGM,Irgm1,ISG20,MT-ND6,MYD88,NAA80,NLRP12,NR1I3,NR4A1,PLAGL1,RBL1,SCARB1,SIRT3,SMPD2,STAT1,STAT2,STAT5A,TFRC,TLR3,TNFSF10,TOR1AIP2,TRIM21,TSPAN33,UGCG,UGT1A1,USP12,ZC3H12A
**CIITA**	-1.931	1.38E-02	CCND1,CD74,GCNT2,HLA-A,HLA-DMA,HLA-DQA1,HLA-DQB1,HLA-DRB5
**BNIP3L**	-1.941	7.02E-04	BLNK,CD19,CENPF,CHEK1,CST3,Dleu2,GADD45A,IL1B,PFKFB3,RBL1,RRM2,TFRC,TOP2A
**FLCN**	-1.949	1.56E-02	ELOVL3,MT-ATP6,MT-CO1,MT-ND1,MT-ND2,MT-ND5,PPARGC1B
**N-cor**	-1.958	1.02E-06	ABCA1,ACACA,ACACB,ARNTL,C1QA,CCND1,CD36,CRP,DHRS3,ELOVL5,ELOVL6,ESR1,FADS2,FASN,G6PD,GSTP1,IDH1,IL1B,PCK1,PDGFA,SCD,Scd2,SLC2A4,SLC44A1
**LMNB1**	-1.970	6.02E-03	DHCR7,FASN,HMGCR,HMGCS1,LDLR,RAD51,RCN2,S100A13,SCD,SQLE
**CFB**	-1.982	4.06E-02	C3,C5,DDIT3,HSPA5
**NUPR1**	-1.990	3.84E-07	ABHD15,ADCY9,AKAP12,ANK3,ATR,AVPI1,B3GAT3,BLM,BTG1,CARMIL1,CASTOR2,CCNF,CDCA2,CDCA3,CHD4,CKAP2L,CROT,CTPS2,CXADR,CYB5B,DDIT3,DHCR24,DHTKD1,DLG1,ETS1,EXTL2,GABBR1,GADD45A,GCH1,GCNT2,GPCPD1,HJURP,IL13RA1,IRF2BP2,LARS2,LBR,LMNB1,MRS2,MYD88,NFIL3,NR1D1,NR1D2,NUCKS1,PCYOX1,PDK1,PEA15,PER3,PFKFB3,PRNP,RAD51,RCN2,RILPL2,SAMD4A,SAMHD1,SESN2,SHCBP1,SHROOM3,SIGMAR1,SKA2,SLC25A20,SPC24,SPDL1,SPTSSA,TANGO6,TERT,TMEM19,TMPO,TNS3,TRIB1,UEVLD,WDR91,ZPR1,ZSWIM6
**Stat3-Stat3**	-1.998	1.12E-02	CRP,FGA,FGB,FGG,HIF1A,LBP,SERPINA3
**MMP2**	-2.000	6.21E-03	CCND1,CSF2RB,EGFR,IL4R,IRF7,OCLN,PLG,TJP1
**MYCN**	-2.029	2.76E-02	ABCA1,ABCA3,ABCB7,ABCD1,AMOTL2,CCND1,CLU,CRIM1,EGFR,HLA-A,KLK3,LDHA,MYL12A,PDIA4,PDK1,PGRMC2,RBBP4,RNF11,RPL10,RPL13A,RPL18A,RPL23,RPL28,RPS26,RPS28,RPS3,RPS6,RPS8,SORD,SUZ12,TERT,ZFAND5
**IRF7**	-2.037	1.64E-02	ADAR,C5,DAXX,GBP5,Hamp/Hamp2,HELZ2,Ifi47,Igtp,IL33,IRF7,IRF9,IRGM,Irgm1,ISG20,OAS1,PSMB9,STAT1,STAT2,TMPO,TNFSF10,TPST1,TRIM21
**IFT88**	-2.177	2.86E-03	ACAT1,ACAT2,ACLY,FDPS,HMGCR,HMGCS1,LSS,TIMP3
**KDM5B**	-2.209	7.32E-06	CCND1,CCNE1,CDC14A,CDCA3,DDIT3,DHCR24,EGR1,ERBB2,ERBB3,ESR1,FABP5,FST,GADD45A,GCA,HMMR,IGFBP2,LGALS3BP,MCM2,MCM3,NCAPH,NDC80,NOL3,PBK,RNASET2,SPTSSA,STAT5A,TOP2A
**OSMR**	-2.219	9.14E-05	BCL3,FASN,FGB,GBP5,Ifi47,Igtp,Irgm1,JUNB,LBP,LRG1,OAS1,PSMB9,SCD,SERPINA1,ZFP36
**Hmgn3**	-2.219	2.31E-03	AHSG,APOA1,APOA2,AZGP1,FGA,Mup1 (includes others),SERPINA1,SLC2A2
**CDKN1A**	-2.256	1.99E-06	ANLN,ATAD2,AURKB,CASP3,CCND1,CCNE1,CENPF,CHAF1B,CHEK1,DTL,FAS,FOXM1,HJURP,HSPA5,IL1B,KNTC1,LBR,LGALS3BP,MBNL2,MCM2,MCM3,MCM5,MCM6,NUSAP1,P4HA1,PBK,PCNA,RAD51,RBL1,RFC4,RRM1,SMC2,SPDL1,STMN1,TERT,TOP2A,TYMS,UHRF1,WNT4
**HNF4A**	-2.290	2.08E-26	ABCC2,ABCG5,ACAA2,ACAD8,ACAT1,ACCS,ACLY,ACMSD,Acnat1/Acnat2,ACSS2,ACSS3,AFP,AGT,AHSA1,AHSG,ALDH1L1,ANXA5,AP3M1,APOA1,APOA2,APOE,APOH,AQP8,ARFIP2,ASGR2,ATAT1,AVPI1,BACE1,BAD,BCLAF1,BCS1L,BDH1,BMP2K,BMP7,BTG1,C14orf119,C3,C6,C8G,CBS/CBSL,CCDC115,CCND1,CCNE1,CDC14A,CDC45,CDCA7L,CDK2AP2,CDKAL1,CEP83,Ces2c,CHD1L,CLCN6,CLDN2,CLPX,COMMD9,COPS7B,CP,CROT,CRP,CRY1,CS,CSNK2A1,CTDSPL2,CUL4B,CWC25,CXADR,Cyp2a12/Cyp2a22,CYP2A6 (includes others),CYP2B6,CYP2E1,CYP4F3,CYP7A1,CYP7B1,DBP,DCK,DDX28,DDX49,DHRS4,DISP2,DNAL1,DSC2,DTL,DYNC2LI1,E2F4,ECI2,EGR1,ELMOD3,EPPK1,ERG28,ETFDH,ETNK2,ETNPPL,FAS,FDPS,FETUB,FGA,FGB,FHIT,FRK,G0S2,G3BP2,G6PC,G6PD,GABPA,GDAP2,GFER,GIPC2,GOLIM4,GPALPP1,GPANK1,GPR137,GPR146,GPX1,GRHPR,GSS,GSTA5,GSTM4,GSTO1,GTF2H3,GUCY2C,GUF1,HADHB,Hamp/Hamp2,HIF1A,HLA-A,HSD11B1,Hsd3b4 (includes others),HSP90B1,HSPA5,HSPA8,HSPH1,IFT122,IL22RA1,INHBC,INHBE,IPO13,ITGB3BP,ITIH3,ITIH4,JUNB,KCNC3,KIF1B,KRT8,LARP4,LARS2,LBHD1,LBP,LCMT2,LDHA,LDLR,LIMS1,LIPC,LOC102724788/PRODH,LPCAT3,LPGAT1,LRRC40,MCM3,MGST3,MID1IP1,MPZL2,MREG,MRPL18,MRPL34,MRPL4,MRPL49,MS4A8,MSRB1,NCBP1,NDC80,NDUFA1,NOP16,NOX4,NR0B2,NR1D1,NR1H3,NR1I3,NTRK2,NUAK1,NUAK2,NUFIP1,OCLN,OGFR,P2RY2,PABPN1,PALMD,PANK1,PCK1,PCNA,PCNP,PCYT1A,PDSS2,PGGT1B,PGM3,PHKB,PKLR,PLA2G12B,PLG,PNKP,PNPLA3,PNPLA6,POC1A,POLR3F,PON1,PPARD,PPIP5K2,PPL,PPP1R10,PPP1R3C,PRCC,PRODH2,PROM1,PRR14,PRRG2,PTPN4,PTPRG,PURG,RABAC1,RABEPK,RABGEF1,RAD51,RAP2C,RBBP4,RBKS,RBL1,RDH11,RHPN2,RIOK2,ROCK1,RPA2,RPL10,RPL18A,RPS6,RPS6KA1,RPS6KC1,RRM1,SAMHD1,SAMM50,SAP30L,SCARB1,SCD,SCP2,SDF2,SEPSECS,SERPINA1,SERPINA3,SESN2,SGCE,SGK2,SHFL,SLC17A2,SLC17A5,SLC25A20,SLC27A2,SLC2A2,SLC35B1,SLC38A4,SLC43A1,SLC44A1,Slco1a1,SLPI,SMC2,SPTSSA,SSBP1,STAM,STAT1,STOML1,SUGCT,SUGT1,SYTL4,TAGLN2,TBC1D10A,TBC1D23,TCF19,TDP2,TEF,TFPT,TFR2,TFRC,THPO,TIMM9,TIMP3,TLE3,TM4SF4,TMEM30A,TMEM63A,TMEM87B,TP53RK,TUBE1,TYMS,UBE2D3,UGT1A1,UGT1A10 (includes others),UGT1A6,UROS,USP2,UTP14C,VDAC1,VKORC1,VTN,WDR12,WNT4,WRNIP1,XPO1,XRCC4,YPEL3,ZBTB45,ZC3H10,ZFP36,ZFYVE19

Table S5 List of Upstream Regulators signficantly enriched and differentially activated/inhibited in the CCl4 model (KO vs WT).

Upstream Regulator	Z-Score	p-value	Target Molecules in Dataset
LEP	-2.347	2.50E-11	ABCG5,ACACA,ACADM,ACADVL,ACAT1,ACLY,ACSL3,ACSL5,APOA1,APOA2,APOH,APPL1,ASS1,ATP2A2,BAD,CASP3,CASP7,CCND1,CD14,CD36,CPS1,CROT,CRP,CYP17A1,Cyp2a12/Cyp2a22,CYP2C18,Cyp2c54 (includes others),Cyp2d9 (includes others),CYP2E1,CYP7A1,ECH1,EGR1,EHHADH,ELOVL5,ELOVL6,EPHX2,ERBB2,ESR1,ETFDH,F3,FADS2,FAS,FASN,G6PC,GADD45A,GCLC,GPAM,GPX1,Gstm3,HMGCR,HSPA5,IDH1,IGFBP2,IL1B,Irgm1,JUNB,LDLR,LIPC,MT-ND1,NPR2,PCK1,PCNA,PLP1,PPARGC1B,SC5D,SCARB1,SCD,Scd2,SCP2,SLC2A2,SLC2A4,SMPD2,SORD,TERT,THRSP,TIMP3,Tmem18,TNFSF10,ZFP36
THBS4	-2.387	3.24E-02	CALR,CRELD2,HSPA5,HYOU1,MANF,SDF2L1
TXNIP	-2.442	2.32E-03	CCND1,CD36,DHCR7,FASN,G0S2,IL1B,NOX4,PCNA,STARD4,THRSP
SPARC	-2.598	1.20E-05	AGK,BHLHE40,BLM,C17orf78,CABYR,CHAF1A,CHAF1B,CHRNA2,CIART,CTSF,ELMOD3,FANCB,GPD1,HAT1,MCM6,P2RY2,POLE,RFC4,RRM1,SNAI1,UBE2U,USP2,WNK4
NRG4	-2.599	1.07E-05	ABCA1,ACACA,EHHADH,ELOVL5,FASN,ME1,SCD
IRF3	-2.638	4.82E-03	ABCC2,ADAR,DAXX,FAS,FST,GBP5,Hamp/Hamp2,HELZ2,Ifi27l2a/Ifi27l2b,Ifi47,Igtp,IL1B,IRF7,IRGM,Irgm1,ISG20,JUNB,MARCHF6,OAS1,PRNP,STAT1,STAT2,TIMP3,TLR3,TMPO,TNFSF10,TPST1
EIF2AK2	-2.747	4.54E-02	BHLHE40,CCND1,DDIT3,EGR1,FAS,GABBR1,Hspa1b,IL1B,ISG20,LGALS3BP,OAS1,SAMHD1,STAT1
TCF3	-2.765	2.39E-05	ACACB,ANLN,ARSA,ASF1B,AZGP1,BIK,BLNK,C15orf39,CASP3,CBFA2T3,CCNE1,CDC45,CDCA3,CELA1,CRELD2,DCK,DNTT,DUT,E2F4,ESCO2,GADD45A,GM2A,GPAM,Hmgn2 (includes others),HMMR,HSD11B1,HSP90B1,HYOU1,ID3,IL1B,LRBA,MANF,NCAPG,NDC80,NFIL3,NR0B2,NTRK2,NUSAP1,PDIA4,PDIA6,PMS2,RRM2,SDF2L1,SLC35B1,SMAGP,SPC24,TOP2A,TYMS,ZC3H12A
IFNG	-2.983	1.79E-04	ABCA1,ACLY,ADAMTS9,AGT,ALDH1L1,ASS1,ATP1B1,ATP2A2,AVPR1A,AZGP1,BACE1,BACH1,BCL3,BLNK,BTG1,C1QA,C1R,C3,CAPN3,CASP3,CASP7,Ccl9,CCND1,CD14,CD276,CD36,CD74,CELSR1,CENPJ,CENPM,CFB,CHAC1,CP,CREM,CSF2RB,CTSH,CXADR,Cyb5r3,CYP2E1,DAXX,DBP,DDIAS,DDIT3,DPP4,E2F4,EGR1,ENTPD2,ERBB2,F3,FABP5,FAS,FASN,FCGR3A/FCGR3B,FGG,GAS5,GAS6,GBP5,GCH1,GLUL,GPR108,GPR146,GSTP1,HCK,HIF1A,HLA-A,HLA-DMA,HLA-DQA1,HLA-DQB1,HLA-DRB5,HMGCR,HMGCS1,HSPA5,HSPA8,HSPB1,HSPG2,Ifi47,Igtp,IL1B,IL22RA1,IL4R,IREB2,IRF7,IRF9,IRGM,Irgm1,ISG20,ITGA5,JUNB,KLF2,LDHA,LGALS3BP,MLKL,MTSS1,MYD88,NOX4,NR1D1,NTRK2,OAS1,OCLN,OGFR,P2RY1,PBK,PCK1,PDGFA,PEA15,PFKFB3,PIGR,PLAAT3,PPARD,PPARGC1B,PRKCA,PRNP,PRSS8,PSMB9,RFXANK,SAMHD1,SCARB1,SCUBE1,SERPINA1,SERPINB9,SERPING1,SHB,SLC11A2,SLC2A2,SLC2A4,Slfn2,SLPI,SMAD1,SMAGP,SQLE,SSBP1,STAT1,STAT2,TBC1D10A,TERT,TFRC,TGFBR2,THPO,TIMP3,TJP1,TLR3,TLR5,TNFRSF1B,TNFSF10,TRAF2,TRIM21,TSC22D3,TSPAN33,TYMP,ZFP36,ZPR1
KLF3	-3.000	3.54E-02	ACOT11,ANXA5,APOE,BCAP29,BCL3,Cyb5r3,ELOF1,FAM126A,FCGRT,GIMAP8,GPD2,GPR137,GSTP1,HDAC11,HEXB,HLCS,IDH1,LENG9,MBLAC2,MRPL34,PCYOX1,PDLIM1,PKNOX1,PPM1G,Proser3,RAP2C,SCP2,SGCB,SNRK,TATDN2,TMPO,TRABD,UGCG,WSB1,ZDHHC14,ZNF367
XBP1	-3.004	3.15E-07	ACACA,ACACB,AFP,ALG12,APOA1,ATP2A2,BFAR,CALR,CREB3,CYP2E1,DDIT3,DNAJB11,DNAJC3,EDEM2,ERO1B,ERP29,ESR1,ETS1,FAS,FASN,FKBP2,GCC1,HSP90B1,HSPA5,HYOU1,MOGS,NR1H3,NUCB2,PCYT1A,PDIA3,PDIA4,PDIA6,PGM3,PIGA,PRNP,RABAC1,Rrbp1,SCD,SDF2L1,SERPINA1,SSR4,SYVN1
INSIG2	-3.092	1.97E-08	ACACA,ACLY,ELOVL6,FASN,FDPS,G6PD,GPAM,HMGCR,LDLR,SCD
Rb	-3.106	3.02E-03	AURKB,CCND1,CCNE1,FOXM1,MCM3,MCM5,PCNA,PCYT1A,RBL1,RFC4,RPA2,TERT,TOP2A,TYMS
STAT5B	-3.330	2.07E-10	Acnat1/Acnat2,ACOT11,ADGRV1,AGPAT2,ANKRD13B,Aox3,ARNT,ATP2A2,C8B,C9,CASP3,CCDC141,CCND1,CCNE1,CD276,CDO1,CHEK1,CYP17A1,Cyp2a12/Cyp2a22,CYP2A6 (includes others),CYP2B6,Cyp2d9 (includes others),DDIT3,EGFR,ELOVL3,ESR1,FAM126A,FAS,G0S2,GADD45A,GPD1,GSTP1,Gstt3,Hamp/Hamp2,Hsd3b4 (includes others),IGFALS,LAMA3,ME1,Mup1 (includes others),NOL3,NOX4,NT5E,Pcp4l1,PLTP,PPP1R3C,PROM1,RAD51B,SCD,SLC16A5,SLC51B,Slco1a1,STAT5A,THRSP,TMEM98,TRAF2,ZFP36
SMARCB1	-3.430	3.34E-05	ACAT1,APOA5,ATP1B1,BTG1,CCND1,CCNE1,CRP,Cyp2c54 (includes others),Cyp2d26,Cyp2d9 (includes others),CYP2E1,DSC2,ERBB2,FAS,G6PC,GADD45A,HP,IGFBP2,LBP,MCM2,MCM3,MCM5,Mup1 (includes others),OAS1,PCK1,PFKFB1,POLA1,PPP1R3C,RAD51AP1,S100A1
CDKN2A	-3.681	8.03E-05	ASF1B,ATAD2,AURKB,BLM,BTG2,C3,CASP3,Ccl9,CCND1,CCNE1,CDCA7L,CENPK,CHAF1A,CNOT6L,CRP,DCK,DEK,EGR1,FST,GAS7,IGFBP5,IL1B,KLK3,MCM5,MELK,PCNA,PDGFA,PEG3,PLAGL1,PSMB9,RAD51AP1,RBL1,RFC4,RRM1,RRM2,SAE1,SUZ12,TCF19,TMEM97,TMPO,TNFRSF1B,TP53INP1,ZNF385A
INSIG1	-4.218	3.86E-14	AACS,ABCA1,ACACA,ACACB,ACLY,ACSL4,ACSS2,APOE,DHCR24,DHCR7,ELOVL6,FABP5,FADS1,FADS2,FASN,FDPS,G6PD,GPAM,HLA-DQA1,HMGCR,HMGCS1,HMGCS2,LDLR,LIPG,LPCAT3,LSS,PMVK,SCAP,SCARB1,SCD,Scd2,SLC2A2,SQLE,STARD4,STAT5A

Table S6 List of Upstream Regulators predicted by IPA to be activated/inhibited with the same pattern of regulation in both Het KO (vs. WT) and both CCL4 and WDSW experiments.

	WDSW			CCL4		
**Upstream Regulators**	**WDSW VS. LFD**	**HET VS. WT**	**KO VS. WT**	**CCL4 VS. OO**	**HET VS. WT**	**KO VS. WT**
SCAP	-3.79	3.27	5.54	-0.96	3.81	5.12
SREBF1	-1.46	2.38	5.06	1.71	3.66	4.84
SREBF2	-3.81	1.87	4.94	0.02	3.89	4.67
ATP7B	-3.71	3.32	4.36	-2.71	2.65	2.18
INSR	-1.72	0.51	3.07	-0.24	2.38	0.06
PPARGC1B	-2.02	1.82	3.04	-0.88	2.05	2.36
PPARG	1.74	1.52	2.69	-1.12	2.22	1.95
NR1H3	0.56	1.25	2.67	2.63	2.25	3.00
INS1	0.84	0.24	2.49	2.11	3.24	2.76
NR1H2	0.49	1.99	2.45	1.73	2.36	2.89
ACSS2	1.13	2.65	2.45	-1.89	2.45	1.90
FASN	0.72	0.91	2.45	0.13	2.14	1.53
NCOA2	-0.65	0.22	2.45	0.51	2.22	1.25
MLXIPL	0.24	1.50	2.43	0.00	2.07	2.56
MAPK9	0.86	1.08	2.42	0.21	2.21	1.12
PPARD	1.93	0.15	2.21	-0.11	0.43	1.66
SCD	-0.10	1.16	2.03	1.12	1.18	1.54
FGF21	0.63	2.19	1.96	0.00	1.70	0.79
SOCS3	0.67	2.40	1.93	-2.57	1.57	3.23
ARNTL	0.30	2.03	1.75	0.00	1.17	1.75
TSC2	-0.91	3.24	1.59	-4.14	2.57	1.13
RXRA	0.31	2.68	1.27	-0.27	0.22	1.40
HSPA5	1.93	1.66	1.15	0.00	2.19	0.78
ADIPOQ	1.03	2.09	1.04	-2.18	1.98	0.06
ZBTB20	-0.85	1.94	0.71	-2.18	1.34	0.54
FMO3	1.63	2.00	0.45	0.00	1.00	1.63
APOE	-0.17	3.95	0.24	-3.31	2.34	1.64
APOA1	-1.14	2.18	0.24	-2.07	1.09	1.73
CEBPB	1.23	-2.81	-0.22	2.14	-0.80	-1.11
NOS2	0.74	-2.85	-0.59	2.79	-2.36	-0.98
FOS	-0.75	-2.34	-0.84	1.47	-1.12	-0.90
APP	2.61	-2.38	-1.04	3.90	-0.43	-1.72
N-COR	-2.58	-0.49	-1.16	-1.36	-2.36	-1.96
PML	0.78	-1.23	-1.68	0.24	-2.15	-1.39
STAT1	3.60	-4.10	-1.72	5.63	-2.04	-1.52
IL17A	2.12	-3.05	-1.79	3.45	-1.01	-1.78
SMAD3	1.44	-3.78	-1.80	2.37	-1.58	-0.27
TNF	1.54	-6.90	-1.85	5.52	-1.20	-1.04
IL1B	1.74	-5.72	-1.97	5.29	-1.12	-0.34
HMGA1	1.29	-0.64	-2.13	1.62	-1.22	-0.61
MAP3K1	1.92	-3.11	-2.20	3.14	-1.49	-1.24
TLR4	1.50	-4.73	-2.21	3.54	-1.85	-1.93
IFNG	2.95	-6.83	-2.31	6.64	-3.57	-2.98
MAPK14	1.40	-3.09	-2.38	3.49	-3.09	-2.51
IFNA	2.94	-3.91	-2.38	5.43	-2.72	-1.62
IRF1	0.00	-2.04	-2.40	2.90	-1.97	-0.94
ACACB	1.03	-1.43	-2.43	-0.69	-1.98	-2.43
RIPK2	1.75	-2.55	-2.73	1.16	-2.00	-1.35
FGF19	-0.32	-2.60	-2.78	0.49	-1.71	-1.67
ELOVL5	-1.21	-2.20	-2.79	0.00	-2.05	-1.89
LEP	1.60	-3.01	-2.92	2.62	-2.28	-2.35
INSIG2	0.68	-1.97	-2.93	0.00	-2.59	-3.09
OGA	3.03	-3.05	-3.03	2.83	-2.88	-2.70
LDL	1.86	-4.39	-3.07	4.45	-2.69	-0.91
MFSD2A	1.94	-1.39	-3.30	0.45	-2.83	-3.46
FOXO1	1.34	-3.21	-3.50	3.40	-2.14	-0.15
POR	2.07	-0.30	-3.86	-1.32	-2.55	-1.52

Table S7 List of genes differentially expressed in both both CCL4 and WDSW experiments at the comparison KO vs. WT.

		WDSW			CCL4		
Ensembl(A1)	Symbol	Log Ratio	p-value	FDR	Log Ratio	p-value	FDR
ENSMUSG00000027577	CHRNA4	1.722	1.69E-04	2.76E-02	5.769	2.11E-17	2.42E-14
ENSMUSG00000041653	PNPLA3	1.059	1.20E-03	6.27E-02	4.913	2.96E-07	5.38E-05
ENSMUSG00000025014	DNTT	0.754	7.65E-03	1.57E-01	4.32	2.98E-19	5.55E-16
ENSMUSG00000089943	UGT1A4	0.75	1.74E-02	2.31E-01	3.718	5.57E-41	8.31E-37
ENSMUSG00000042010	ACACB	1.137	7.01E-06	4.85E-03	3.02	9.51E-32	7.09E-28
ENSMUSG00000027605	ACSS2	1.084	2.39E-04	3.21E-02	2.988	9.67E-26	4.81E-22
ENSMUSG00000035686	THRSP	1.674	8.53E-07	1.57E-03	2.961	2.64E-04	1.10E-02
ENSMUSG00000041220	ELOVL6	0.741	1.19E-02	1.96E-01	2.84	1.38E-18	1.88E-15
ENSMUSG00000025153	FASN	0.946	3.52E-04	3.76E-02	2.779	8.00E-08	1.81E-05
ENSMUSG00000020917	ACLY	1.084	3.84E-05	1.34E-02	2.604	7.93E-20	1.69E-16
ENSMUSG00000028716	PDZK1IP1	0.756	3.37E-02	3.06E-01	2.602	7.49E-19	1.12E-15
ENSMUSG00000027533	FABP5	1.477	3.82E-03	1.15E-01	2.564	1.39E-21	4.14E-18
ENSMUSG00000018566	SLC2A4	1.611	3.38E-04	3.74E-02	2.561	1.92E-05	1.60E-03
ENSMUSG00000021670	HMGCR	1.08	7.44E-05	1.74E-02	2.35	6.49E-06	6.63E-04
ENSMUSG00000003555	CYP17A1	0.744	2.16E-02	2.51E-01	2.263	4.03E-05	2.81E-03
ENSMUSG00000024978	GPAM	0.931	6.52E-04	4.86E-02	2.108	1.89E-11	1.09E-08
ENSMUSG00000104501	Gm37736	1.454	1.39E-03	6.71E-02	2.093	1.02E-06	1.45E-04
ENSMUSG00000033105	LSS	0.652	2.54E-03	9.39E-02	1.994	3.50E-05	2.55E-03
ENSMUSG00000032561	ACPP	2.282	1.11E-04	2.24E-02	1.897	1.11E-11	7.54E-09
ENSMUSG00000026839	UPP2	1.609	2.38E-04	3.21E-02	1.891	5.63E-09	1.68E-06
ENSMUSG00000030256	Bhlhe41	1.356	3.20E-02	2.99E-01	1.858	4.14E-07	7.10E-05
ENSMUSG00000025429	PSTPIP2	0.588	6.14E-03	1.44E-01	1.844	1.45E-11	9.03E-09
ENSMUSG00000038550	CIART	1.517	1.13E-02	1.91E-01	1.836	3.43E-02	2.37E-01
ENSMUSG00000001665	Gstt3	0.712	3.82E-03	1.15E-01	1.801	1.36E-11	8.84E-09
ENSMUSG00000027346	GPCPD1	1.198	6.17E-04	4.73E-02	1.749	1.21E-12	9.06E-10
ENSMUSG00000028957	PER3	1.224	9.60E-04	5.78E-02	1.746	7.77E-07	1.18E-04
ENSMUSG00000059824	DBP	0.954	2.44E-02	2.63E-01	1.735	2.17E-03	4.63E-02
ENSMUSG00000026471	MR1	0.839	7.41E-04	5.11E-02	1.726	1.62E-10	7.05E-08
ENSMUSG00000066441	RDH11	0.727	8.13E-03	1.63E-01	1.717	9.20E-11	4.58E-08
ENSMUSG00000059743	FDPS	1.028	7.45E-05	1.74E-02	1.625	1.12E-02	1.30E-01
ENSMUSG00000022351	SQLE	1.058	2.90E-02	2.84E-01	1.531	9.45E-03	1.18E-01
ENSMUSG00000042216	SGSM1	0.793	7.62E-03	1.57E-01	1.518	1.45E-04	7.10E-03
ENSMUSG00000027952	PMVK	0.542	2.35E-02	2.60E-01	1.517	2.29E-03	4.82E-02
ENSMUSG00000029482	AACS	0.847	3.28E-04	3.74E-02	1.497	3.26E-02	2.30E-01
ENSMUSG00000032010	USP2	1.274	9.64E-03	1.76E-01	1.494	3.46E-02	2.38E-01
ENSMUSG00000093651	Gm5873	1.027	5.94E-05	1.69E-02	1.474	1.40E-02	1.46E-01
ENSMUSG00000100094	1810008I18Rik	0.842	7.86E-06	5.06E-03	1.45	5.95E-09	1.74E-06
ENSMUSG00000021747	C3orf67	0.767	4.07E-02	3.27E-01	1.414	6.13E-03	9.08E-02
ENSMUSG00000079470	UTP14C	0.712	1.50E-03	6.97E-02	1.403	4.28E-05	2.92E-03
ENSMUSG00000026692	FMO4	0.45	2.42E-02	2.61E-01	1.392	1.61E-07	3.48E-05
ENSMUSG00000113859	Gm7979	0.894	3.56E-03	1.13E-01	1.383	2.48E-03	5.07E-02
ENSMUSG00000024899	PAPSS2	0.641	4.04E-06	4.27E-03	1.365	7.75E-10	2.69E-07
ENSMUSG00000058454	DHCR7	0.64	7.75E-04	5.17E-02	1.355	9.03E-05	4.88E-03
ENSMUSG00000090610	Gm3571	0.91	2.52E-04	3.31E-02	1.341	6.50E-03	9.39E-02
ENSMUSG00000034837	GNAT1	0.924	1.43E-04	2.56E-02	1.328	7.59E-09	2.18E-06
ENSMUSG00000069601	ANK3	0.643	1.10E-02	1.89E-01	1.317	8.00E-16	7.96E-13
ENSMUSG00000028976	SLC2A5	1.029	4.56E-04	4.13E-02	1.283	1.42E-05	1.28E-03
ENSMUSG00000026723	TRDMT1	0.521	2.84E-02	2.81E-01	1.269	1.15E-03	3.02E-02
ENSMUSG00000006711	KIAA0319	0.489	4.85E-02	3.53E-01	1.165	2.50E-05	1.99E-03
ENSMUSG00000050069	GREM2	1.326	4.56E-04	4.13E-02	1.163	5.33E-07	8.65E-05
ENSMUSG00000032883	ACSL3	0.448	4.33E-03	1.24E-01	1.15	1.22E-04	6.19E-03
ENSMUSG00000032018	SC5D	0.443	8.70E-05	1.90E-02	1.131	4.17E-05	2.87E-03
ENSMUSG00000084839	Gm14097	1.2	1.05E-02	1.84E-01	1.119	4.15E-02	2.61E-01
ENSMUSG00000028051	HCN3	0.69	1.91E-02	2.40E-01	1.115	4.20E-02	2.62E-01
ENSMUSG00000030528	BLM	0.971	1.46E-03	6.91E-02	1.104	2.27E-04	1.00E-02
ENSMUSG00000090622	A930033H14Rik	1.393	1.42E-03	6.81E-02	1.075	4.54E-02	2.73E-01
ENSMUSG00000026675	HSD17B7	0.459	1.51E-02	2.16E-01	1.045	1.18E-03	3.10E-02
ENSMUSG00000024665	FADS2	0.76	3.40E-04	3.74E-02	1.037	3.58E-04	1.32E-02
ENSMUSG00000008035	MID1IP1	0.469	7.01E-03	1.53E-01	1.013	5.18E-03	8.16E-02
ENSMUSG00000021589	RHOBTB3	0.432	1.59E-02	2.22E-01	1.01	1.51E-04	7.33E-03
ENSMUSG00000044254	PCSK9	0.577	1.40E-03	6.74E-02	1.009	6.64E-03	9.48E-02
ENSMUSG00000044250	PCED1B	0.4	5.48E-03	1.40E-01	1.008	2.56E-03	5.16E-02
ENSMUSG00000027765	P2RY1	0.672	7.30E-03	1.54E-01	0.986	4.38E-03	7.33E-02
ENSMUSG00000024378	STARD4	0.651	2.87E-05	1.03E-02	0.97	1.52E-05	1.34E-03
ENSMUSG00000018727	CPSF4L	0.542	3.01E-02	2.89E-01	0.962	1.59E-03	3.75E-02
ENSMUSG00000003526	LOC102724788/PRODH	0.858	7.94E-05	1.81E-02	0.952	3.78E-05	2.70E-03

ENSMUSG00000020334	SLC22A4	0.427	2.09E-02	2.47E-01	0.931	5.99E-03	8.93E-02
ENSMUSG00000024354	SLC23A1	0.499	7.79E-03	1.58E-01	0.926	4.36E-04	1.51E-02
ENSMUSG00000022679	MPV17L	0.78	1.97E-04	2.85E-02	0.915	4.52E-03	7.48E-02
ENSMUSG00000038072	GALNT11	0.529	5.95E-03	1.42E-01	0.904	1.25E-08	3.34E-06
ENSMUSG00000052302	TBC1D30	0.641	5.84E-03	1.42E-01	0.887	3.67E-03	6.54E-02
ENSMUSG00000041237	PKLR	0.508	2.48E-02	2.64E-01	0.87	7.32E-04	2.20E-02
ENSMUSG00000019883	ECHDC1	0.38	3.39E-03	1.10E-01	0.864	9.97E-04	2.79E-02
ENSMUSG00000015806	QDPR	0.39	1.04E-03	5.87E-02	0.86	3.15E-04	1.24E-02
ENSMUSG00000025271	PFKFB1	0.732	4.31E-06	4.27E-03	0.855	7.39E-04	2.22E-02
ENSMUSG00000010663	FADS1	0.435	1.19E-03	6.24E-02	0.846	3.28E-04	1.25E-02
ENSMUSG00000046687	Gm5424	0.543	1.44E-02	2.13E-01	0.819	2.67E-04	1.10E-02
ENSMUSG00000025991	CPS1	0.632	3.45E-03	1.11E-01	0.797	2.45E-04	1.05E-02
ENSMUSG00000076441	ASS1	0.542	1.66E-02	2.26E-01	0.775	4.36E-04	1.51E-02
ENSMUSG00000019437	TLCD1	0.425	4.10E-04	4.03E-02	0.77	2.90E-04	1.16E-02
ENSMUSG00000075543	URAD	1.187	1.21E-04	2.30E-02	0.765	2.45E-04	1.05E-02
ENSMUSG00000074052	BC048644	0.536	1.23E-02	1.99E-01	0.753	2.62E-03	5.25E-02
ENSMUSG00000037458	AZIN1	0.422	1.46E-03	6.91E-02	0.751	3.11E-06	3.80E-04
ENSMUSG00000030711	Sult1a1	0.418	6.99E-04	5.01E-02	0.751	6.32E-07	9.93E-05
ENSMUSG00000051041	OLFML1	0.589	1.43E-04	2.56E-02	0.731	1.99E-03	4.33E-02
ENSMUSG00000070960	Gm19680	0.522	8.28E-04	5.28E-02	0.73	2.17E-02	1.86E-01
ENSMUSG00000022389	TEF	0.684	7.12E-04	5.03E-02	0.72	6.53E-03	9.39E-02
ENSMUSG00000032193	LDLR	0.471	1.13E-03	6.07E-02	0.71	1.55E-02	1.56E-01
ENSMUSG00000052656	RNF103	0.544	1.39E-05	6.18E-03	0.709	1.74E-06	2.26E-04
ENSMUSG00000032311	NRG4	0.615	2.53E-03	9.39E-02	0.704	1.18E-02	1.34E-01
ENSMUSG00000038145	SNRK	0.565	8.21E-04	5.28E-02	0.701	6.80E-03	9.59E-02
ENSMUSG00000046541	ZNF526	0.63	1.77E-03	7.53E-02	0.683	2.41E-02	1.99E-01
ENSMUSG00000037847	NMRK1	0.499	2.10E-03	8.49E-02	0.661	1.19E-03	3.10E-02
ENSMUSG00000030088	ALDH1L1	0.638	8.67E-06	5.29E-03	0.649	1.56E-02	1.56E-01
ENSMUSG00000025815	DHTKD1	0.443	3.94E-03	1.17E-01	0.64	4.51E-03	7.47E-02
ENSMUSG00000070305	MPZL3	0.432	8.79E-04	5.48E-02	0.637	2.72E-03	5.36E-02
ENSMUSG00000000056	NARF	0.524	7.58E-04	5.13E-02	0.636	1.42E-03	3.50E-02
ENSMUSG00000022353	MTSS1	0.597	1.77E-04	2.85E-02	0.631	1.98E-03	4.32E-02
ENSMUSG00000024052	LPIN2	0.43	1.71E-02	2.28E-01	0.625	2.18E-02	1.87E-01
ENSMUSG00000020190	MKNK2	0.595	2.86E-03	9.87E-02	0.623	7.15E-03	9.95E-02
ENSMUSG00000032500	DCLK3	0.832	1.04E-05	5.34E-03	0.617	2.55E-02	2.04E-01
ENSMUSG00000027075	SLC43A1	1.619	2.02E-04	2.85E-02	0.61	1.45E-02	1.49E-01
ENSMUSG00000029269	SULT1B1	0.474	4.55E-02	3.43E-01	0.609	7.78E-04	2.30E-02
ENSMUSG00000010064	SLC38A3	0.793	7.16E-06	4.85E-03	0.598	9.64E-04	2.72E-02
ENSMUSG00000030064	FRMD4B	0.413	7.00E-03	1.53E-01	0.595	1.55E-02	1.56E-01
ENSMUSG00000037278	TMEM97	0.455	1.74E-03	7.48E-02	0.593	1.65E-03	3.84E-02
ENSMUSG00000024135	SRBD1	0.391	1.28E-03	6.41E-02	0.583	8.07E-04	2.38E-02
ENSMUSG00000004768	RAB23	0.43	8.67E-03	1.68E-01	0.58	2.68E-02	2.09E-01
ENSMUSG00000047767	ATG16L2	0.636	6.58E-05	1.74E-02	0.572	1.55E-02	1.56E-01
ENSMUSG00000032092	MPZL2	0.379	9.45E-03	1.74E-01	0.564	1.31E-02	1.41E-01
ENSMUSG00000028402	MPDZ	0.494	1.82E-04	2.85E-02	0.556	1.25E-02	1.38E-01
ENSMUSG00000051339	2900026A02Rik	0.388	1.74E-03	7.48E-02	0.551	1.79E-03	4.06E-02
ENSMUSG00000116718	AC154378.1	0.447	1.95E-02	2.41E-01	0.551	2.11E-02	1.83E-01
ENSMUSG00000032485	SCAP	0.482	1.05E-03	5.87E-02	0.549	1.06E-03	2.88E-02
ENSMUSG00000042118	BHMT2	0.449	1.55E-05	6.44E-03	0.527	1.82E-03	4.09E-02
ENSMUSG00000018042	Cyb5r3	0.391	1.56E-03	7.13E-02	0.525	1.20E-02	1.35E-01
ENSMUSG00000038641	AKR1D1	0.439	2.03E-02	2.45E-01	0.515	3.41E-02	2.37E-01
ENSMUSG00000027165	C11orf74	0.462	1.49E-08	9.63E-05	0.515	6.28E-03	9.17E-02
ENSMUSG00000070644	ETNK2	0.652	4.61E-05	1.45E-02	0.505	1.64E-02	1.61E-01
ENSMUSG00000044197	GPR146	0.564	5.69E-04	4.60E-02	0.484	1.38E-02	1.45E-01
ENSMUSG00000018821	AVPI1	0.4	1.18E-04	2.30E-02	0.477	3.29E-02	2.32E-01
ENSMUSG00000028186	Uox	0.395	1.79E-03	7.57E-02	0.446	9.51E-03	1.18E-01
ENSMUSG00000025577	CBX2	0.427	4.20E-02	3.31E-01	0.442	4.97E-02	2.85E-01
ENSMUSG00000022464	SLC38A4	0.433	2.77E-03	9.79E-02	0.406	7.57E-03	1.03E-01
ENSMUSG00000040820	HLCS	0.555	1.04E-03	5.87E-02	0.392	4.44E-02	2.69E-01
ENSMUSG00000021097	CLMN	0.398	6.94E-03	1.53E-01	0.38	3.26E-02	2.30E-01
ENSMUSG00000027801	TM4SF4	-0.472	2.39E-02	2.60E-01	-0.387	4.34E-02	2.66E-01
ENSMUSG00000029233	SRD5A3	-0.38	3.43E-04	3.74E-02	-0.392	7.55E-03	1.03E-01
ENSMUSG00000032300	C15orf39	-0.464	2.12E-02	2.49E-01	-0.434	3.05E-02	2.23E-01
ENSMUSG00000026491	AHCTF1	-0.392	4.82E-02	3.52E-01	-0.448	2.67E-02	2.09E-01
ENSMUSG00000053175	BCL3	-0.45	3.54E-02	3.10E-01	-0.466	1.80E-02	1.70E-01
ENSMUSG00000023079	GTF2IRD1	-0.407	7.06E-03	1.53E-01	-0.47	2.60E-02	2.06E-01
ENSMUSG00000027610	GSS	-0.386	1.47E-02	2.14E-01	-0.498	1.55E-03	3.70E-02
ENSMUSG00000063558	AOX1	-0.412	1.92E-02	2.40E-01	-0.549	2.66E-02	2.08E-01

ENSMUSG00000016520	LNX2	-0.402	2.68E-03	9.57E-02	-0.552	1.43E-03	3.51E-02
ENSMUSG00000067199	FRAT1	-0.517	1.07E-02	1.86E-01	-0.577	3.35E-02	2.34E-01
ENSMUSG00000023018	SMARCD1	-0.443	5.54E-03	1.40E-01	-0.592	3.42E-02	2.37E-01
ENSMUSG00000000555	ITGA5	-0.453	2.65E-03	9.51E-02	-0.607	1.79E-07	3.82E-05
ENSMUSG00000011305	PLIN5	-0.439	1.42E-02	2.12E-01	-0.613	4.30E-02	2.66E-01
ENSMUSG00000060519	TOR3A	-0.604	6.04E-04	4.72E-02	-0.617	1.39E-02	1.46E-01
ENSMUSG00000036501	FAM13B	-0.502	2.35E-03	9.03E-02	-0.62	9.08E-03	1.16E-01
ENSMUSG00000032350	GCLC	-0.495	1.52E-02	2.18E-01	-0.632	6.42E-03	9.30E-02
ENSMUSG00000031286	Glt28d2	-0.478	3.99E-02	3.25E-01	-0.651	3.31E-02	2.32E-01
ENSMUSG00000024843	CHKA	-0.823	2.52E-03	9.39E-02	-0.681	3.94E-02	2.54E-01
ENSMUSG00000040128	PNRC1	-0.457	1.59E-02	2.22E-01	-0.685	4.88E-04	1.64E-02
ENSMUSG00000000552	ZNF385A	-0.431	1.69E-02	2.28E-01	-0.699	8.57E-04	2.48E-02
ENSMUSG00000038126	MPHOSPH9	-0.467	1.69E-03	7.39E-02	-0.711	1.50E-02	1.53E-01
ENSMUSG00000039395	MREG	-0.449	1.87E-02	2.37E-01	-0.756	1.10E-03	2.95E-02
ENSMUSG00000100147	1700047M11Rik	-0.418	4.45E-02	3.40E-01	-0.771	2.99E-02	2.22E-01
ENSMUSG00000003623	CROT	-0.454	2.72E-03	9.66E-02	-0.78	2.33E-03	4.87E-02
ENSMUSG00000007872	ID3	-0.623	4.29E-03	1.23E-01	-0.822	2.43E-05	1.95E-03
ENSMUSG00000058486	WDR91	-0.418	6.79E-03	1.52E-01	-0.824	1.86E-10	7.67E-08
ENSMUSG00000044847	LSM11	-0.486	7.99E-04	5.22E-02	-0.83	1.00E-02	1.22E-01
ENSMUSG00000015357	CLPX	-0.56	3.97E-03	1.17E-01	-0.889	1.69E-03	3.90E-02
ENSMUSG00000026640	PLXNA2	-0.484	6.89E-03	1.53E-01	-0.909	1.62E-03	3.79E-02
ENSMUSG00000051439	CD14	-0.672	3.69E-02	3.15E-01	-0.957	1.19E-02	1.35E-01
ENSMUSG00000036957	LRFN3	-0.717	4.65E-03	1.30E-01	-1.017	4.76E-05	3.11E-03
ENSMUSG00000029009	MTHFR	-0.802	6.74E-04	4.91E-02	-1.017	8.59E-05	4.71E-03
ENSMUSG00000002265	PEG3	-0.792	2.45E-02	2.63E-01	-1.029	3.04E-02	2.23E-01
ENSMUSG00000017639	RAB11FIP4	-0.74	2.41E-02	2.61E-01	-1.075	1.41E-04	6.97E-03
ENSMUSG00000004098	COL5A3	-0.527	1.25E-02	2.00E-01	-1.157	2.97E-06	3.66E-04
ENSMUSG00000030814	BCL7C	-0.439	1.81E-02	2.34E-01	-1.158	2.23E-07	4.43E-05
ENSMUSG00000030659	NUCB2	-0.595	2.23E-02	2.53E-01	-1.207	1.98E-03	4.32E-02
ENSMUSG00000037157	IL22RA1	-0.827	4.36E-03	1.24E-01	-1.217	1.40E-02	1.46E-01
ENSMUSG00000045287	RTN4RL1	-0.7	3.65E-03	1.14E-01	-1.263	4.72E-05	3.10E-03
ENSMUSG00000028982	SLC25A33	-0.416	9.85E-03	1.78E-01	-1.371	4.43E-08	1.02E-05
ENSMUSG00000004951	HSPB1	-0.591	2.56E-02	2.68E-01	-1.391	5.93E-05	3.61E-03
ENSMUSG00000037621	ATOH8	-0.649	1.61E-03	7.24E-02	-1.426	4.96E-06	5.29E-04
ENSMUSG00000022769	SDF2L1	-0.487	2.39E-02	2.60E-01	-1.519	1.14E-04	5.92E-03
ENSMUSG00000009633	G0S2	-0.609	2.23E-02	2.53E-01	-1.542	4.32E-03	7.27E-02
ENSMUSG00000002831	PLIN4	-1.067	3.52E-03	1.12E-01	-1.554	3.00E-03	5.72E-02
ENSMUSG00000055116	ARNTL	-1.015	1.35E-02	2.08E-01	-1.584	8.72E-05	4.77E-03
ENSMUSG00000025396	HSD17B6	-0.744	1.37E-02	2.09E-01	-1.651	1.25E-03	3.21E-02
ENSMUSG00000059060	RAD51B	-1.275	3.80E-03	1.15E-01	-2.006	5.24E-05	3.33E-03
ENSMUSG00000056749	NFIL3	-0.521	3.39E-02	3.06E-01	-2.132	1.40E-13	1.16E-10

Table S8. Fatty acyl chain composition of complex lipids as confirmed by collision-induced dissociation and tandem mass spectrometry.

Lipid annotation	Fatty acid composition	*m/z*	CID MS/MS	Diagnostic ions
		**PC**		
PC(34:1)	16:0/18:1	760.5856	Yes	496.3, 504.3
PC(34:2)	16:0/18:2	758.5707	Yes	496.3, 502.3
PC(36:4)	16:0/20:4	780.5695	Yes	496.3, 524.3
PC(38:4)	18:0/20:4	810.6018	Yes	506.3, 524.4
PC(38:6)	16:2/22:6	806.5713	Yes	496.4, 550.3
		**TG**		
TG(46:2)	12:0/16:1/18:1 and 14:0/16:1/16:1	792.7073	Yes	493.4, 521.5, 547.5, 575.5
TG(48:1)	16:0/16:0/16:1	822.7561	No	
TG(48:2)	16:0/16:1/16:1 and 14:0/16:1/18:1	820.7412	Yes	521.5, 547.5, 549.5, 575.5
TG(48:3)	16:1/16:1/16:1	818.7248	Yes	547.5
TG(50:1)	16:0/16:0/18:1	850.7855	No	
TG(50:2)	16:0/16:1/18:1	848.7724	Yes	549.5, 575.5, 577.5
TG(50:3)	16:1/16:1/18:1	846.7567	Yes	547.5, 575.5
TG(51:2)	16:0/17:1/18:1 and 15:0/18:1/18:1	862.7861	Yes	563.5, 603.5, 577.5, 589.5
TG(52:1)	16:0/18:0/18:1	878.8170	Yes	577.5, 579.5, 605.5
TG(52:2)	16:0/18:1/18:1	876.8000	Yes	577.5, 603.5
TG(52:3)	16:1/18:1/18:1	874.7862	Yes	575.5, 603.5
TG(52:4)	16:1/18:1/18:2	872.7706	Yes	573.5, 575.5, 601.5
TG(54:1)	18:0/18:0/18:1 and 16:0/18:1/20:0	906.8424	Yes	577.5, 605.6, 607.6, 633.6
TG(54:2)	18:0/18:1/18:1	904.8337	Yes	603.6, 605.6
TG(54:3)	18:1/18:1/18:1	902.8196	Yes	603.5
TG(54:4)	18:1/18:1/18:2	900.8027	Yes	601.6, 603.6
TG(56:2)	18:1/18:1/20:0	932.8570	Yes	603.6, 633.6
TG(56:3)	18:1/18:1/20:1	930.8470	Yes	603.6, 631.6
TG(56:5)	18:1/18:3/20:1	926.8179	Yes	603.5, 627.5
TG(58:2)	18:1/18:1/22:0 and 18:1/20:1/20:0	960.8949	Yes	661.6, 631.6, 603.5, 633.5
TG(58:3)	18:1/18:1/22:1 and 18:1/20:1/20:1	958.8775	Yes	603.5, 631.6, 659.6
